# *PARTICLE* triplexes cluster in the tumor suppressor *WWOX* and may extend throughout the human genome

**DOI:** 10.1038/s41598-017-07295-5

**Published:** 2017-08-02

**Authors:** Valerie Bríd O’Leary, Jan Smida, Fabian Andreas Buske, Laura Garcia Carrascosa, Omid Azimzadeh, Doris Maugg, Sarah Hain, Soile Tapio, Wolfgang Heidenreich, James Kerr, Matt Trau, Saak Victor Ovsepian, Michael John Atkinson

**Affiliations:** 1 Institute of Radiation Biology, Helmholtz Zentrum Munich - German Research Center for Environmental Health, Ingolstaedter Landstrasse 1, 85764 Neuherberg, Germany; 2 Kinghorn Cancer Centre, Garvan Institute of Medical Research, 384 Victoria St., Darlinghurst, Sydney, NSW 2010 Australia; 3 St. Vincent’s Clinical School, University of New South Wales Australia, 390, Victoria Street, Darlinghurst, Sydney, NSW 2010 Australia; 4 Centre for Personalized Nanomedicine, Australian Institute for Bio-engineering and Nanotechnology, The University of Queensland, Corner of College and Cooper Roads, 4072 Brisbane, Queensland Australia; 5 Department of Pediatrics and Children’s Cancer Research Center, Technical University Munich, Munich, Germany; 6 Department of Translational Dermatoinfectiology, Westfaelische Wilhelms University Muenster, Faculty of Medicine, Clinical University Muenster, Rontgenstrasse 21, D48149 Muenster, Germany; 7 School of Chemistry and Molecular Biosciences, The University of Queensland, Cooper Road, Brisbane, QLD 4072 Queensland, Australia; 8 Institute of Biological and Medical Imaging, Helmholtz Zentrum Munich - German Research Center for Environmental Health, Ingolstaedter Landstrasse 1, 85764 Neuherberg, Germany; 9 Faculty for Electrical Engineering and Information Technology, Technical University Munich, Munich, Germany; 10 Chair of Radiation Biology, Technical University Munich, Munich, Germany

## Abstract

The long non-coding RNA *PARTICLE* (Gene *PARTICL*- ‘*P*romoter of *MAT2A*-*A*ntisense *R*adia*T*ion *I*nduced *C*irculating *L*ncRNA) partakes in triple helix (triplex) formation, is transiently elevated following low dose irradiation and regulates transcription of its neighbouring gene - *Methionine adenosyltransferase 2A*. It now emerges that *PARTICLE* triplex sites are predicted in many different genes across all human chromosomes. *In silico* analysis identified additional regions for *PARTICLE* triplexes at >1600 genomic locations. Multiple *PARTICLE* triplexes are clustered predominantly within the human and mouse tumor suppressor *WW Domain Containing Oxidoreductase* (*WWOX*) *gene*. Surface plasmon resonance diffraction and electrophoretic mobility shift assays were consistent with *PARTICLE* triplex formation within human *WWOX* with high resolution imaging demonstrating its enrichment at this locus on chromosome 16. *PARTICLE* knockdown and over-expression resulted in inverse changes in *WWOX* transcripts levels with siRNA interference eliminating *PARTICLEs* elevated transcription to irradiation. The evidence for a second functional site of *PARTICLE* triplex formation at *WWOX* suggests that *PARTICLE* may form triplex-mediated interactions at multiple positions in the human genome including remote loci. These findings provide a mechanistic explanation for the ability of lncRNAs to regulate the expression of numerous genes distributed across the genome.

## Introduction

The relevance of long non-coding RNA (lncRNA) is growing following the discovery of significant roles for members of this abundant family of human transcripts. Although these RNAs may hold some coding capacity for micro peptides^1^, their broad functionality is suggested to be derived from their ability to adopt elaborate structures^2^. The lack of conservation of primary sequences has contributed to the challenge of determining the biological significance of newly discovered lncRNA transcripts. An explanation for the lack of sequence conservation may be the evolutionary conservation of important secondary structural modules^3–5^.

Decades have passed since the observation of RNA triplex formation^6^, but the spotlight has returned to this feature following the most recent mechanistic characterization of lncRNAs^7–9^. Sequence specific interaction between lncRNA and short stretches of genomic DNA leads to the formation of RNA: DNA: DNA triplexes^7–9^. This occurs through the recognition of short homopolypurine/homopolypyrimidine sequences of the duplex DNA by the single-stranded RNA that inserts into the major groove in a parallel or antiparallel orientation^10, 11^. The triplex is stabilised by the formation of Hoogsteen hydrogen bonding between the RNA and the target duplex DNA^12^. Bioinformatics approaches have suggested that multiple triplex target sequences are distributed across the human genome^13^. The concentration of potential triplex sites within regulatory regions of transcribed genes suggests a role for triplexes in controlling gene expression^13^.

Triplex formation has been observed for a number of different lncRNAs such as *Fendrr* (FOXF1 adjacent non-coding developmental regulatory RNA^14^), *MEG3* (maternally expressed 3^9^), *Khps1* (*sphingosine kinase 1a* antisense transcript)^8^ and *PARTICLE* (promoter of *MAT2A* antisense radiation-induced circulating long non-coding RNA^7^). *PARTICLE* triplex formation has been demonstrated *in vitro* in the ‘shore’ region of a CpG island in the *MAT2A* (methionine adenosyltransferase 2 A) promoter^7^. *PARTICLE* binding is part of an active feedback silencing mechanism for *MAT2A*, limiting the increased *MAT2A* transcription that follows exposure to low dose radiation^7^. In contrast to lncRNAs, whose triplex binding has only been investigated at a specific gene (usually in *cis*), we now report that *PARTICLE* triple helix sites are predicted in many different genes across all human chromosomes. A cluster of *PARTICLE* binding sites lies within the tumor suppressor *WWOX* (tryptophan domain containing oxidoreductase). This enrichment is worthy of further study as both *WWOX* and *PARTICLE* are shown to be responsive to environmental stressors^15^. Indeed, we now provide evidence for an interaction between *PARTICLE* and the *WWOX* gene that is consistent with triplex formation. This may be a more general phenomenon and the role of the 1600 plus other potential *PARTICLE* sites should be investigated.

## Results

### Human and mouse *PARTICLE* have different sequences but comparable secondary structure

**Figure 1:**
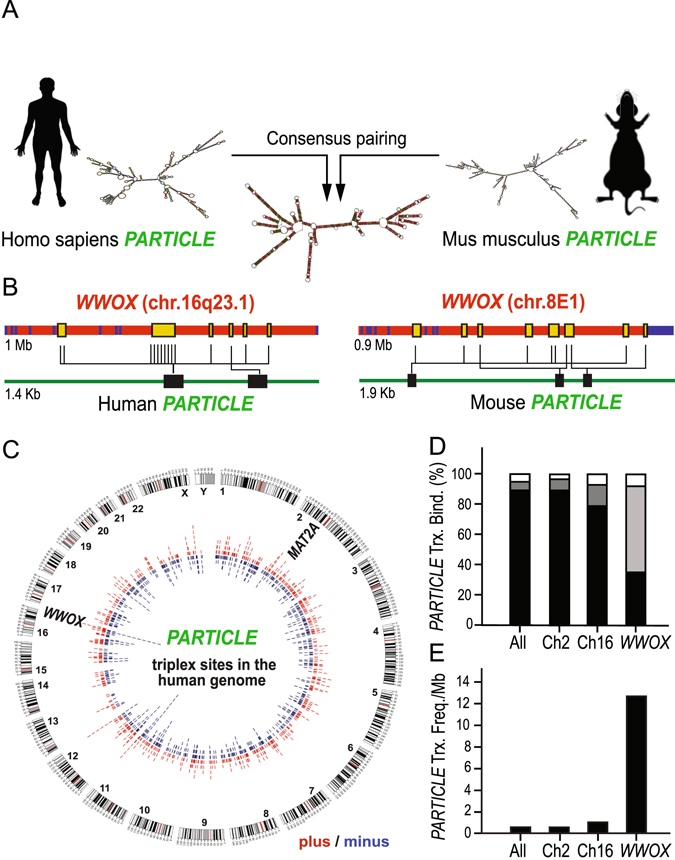
Predicted *PARTICLE* consensus structure and triplex formation with one local enrichment at the *WWOX* tumor suppressor locus on chromosome 16. (**A**) Secondary structure minimum free energy (MFE) prediction of *PARTICLE* in human (left) and mouse (right) with LocARNA consensus pairing (centre; compatible base pairs are colored). (**B**) Schematics of the *WWOX* locus on human chromosome 16 and mouse chromosome 8 (upper, exons in blue boxes) and predicted triplex binding sites (yellow boxes) with *PARTICLE* (vertical lines, lower). (**C**) GENCODE hg19 assembly with *PARTICLE* triplex sites on plus (red) and minus (blue) genomic strands as identified by Triplexator v1.3.2. The genomic location of tumor suppressors *MAT2A* and *WWOX* are indicated. (**D**) Percentage of triplex binding sites (involving *PARTICLE* regions 1098–1114 nucleotides (white), 629–646 nucleotides (grey) and 624–644 nucleotides (black)) and (**E**) *PARTICLE* triplex frequency per megabase on all human chromosomes combined (All), chromosome 2 (Ch2) and 16 (Ch16) and within the *WWOX* locus. Human silhouette (id:47607240, Dreamstime.com).

The presence of important secondary structural elements or functional ‘modules’ interspersed within longer and less conserved stretches of nucleotide sequences within lncRNAs has been proposed^3^. The secondary structure of *PARTICLE* (1432 nucleotides) in human was previously reported^7^. Mouse *PARTICLE* (gene synonym 4930414L22RiK, 1933 nucleotides) was identified from a reference genome assembly^16^. Comparison of human and mouse *PARTICLE* using RNAcentral (RNAcentral.org) revealed 55.7% coverage (797/1432 nucleotides) with 59.2% identity between their nucleotide sequences with the likelihood of finding this alignment by chance highly improbable (E-value = 1.00e^−51^). The optimal secondary structure for human or mouse *PARTICLE* was predicted using the minimum free energy fold algorithm from the RNAfold webserver (http://rna.tbi.univie.ac.at/)^17^ (Fig. 1A). A consensus RNAalifold structure for human and mouse *PARTICLE* transcripts was generated via LocARNA (http://rna.informatik.uni-freiburg.de)^18^ from an alignment pairing of human and mouse *PARTICLE* sequences (Fig. 1A). This enabled a consensus structure to be engineered based on the conservation of *PARTICLE* base pairing between these two species (Fig. 1A).

### *PARTICLE* triplex formation is predicted throughout the human and mouse genomes

A region (chromosome 2: 85765239–85765251) upstream of a *MAT2A* CpG island promoter was identified with Triplexator (v1.3.2) and confirmed by surface plasmon resonance diffraction to be a site of *PARTICLE* triplex formation^7^. *In silico* investigation of *PARTICLE* triplex target sites within the mouse and human whole genomes was undertaken using Triplex Domain Finder^19^. Predicted *PARTICLE* triplex binding sites amounted to approximately 16,900 and 7,200 loci in the mouse and human genomes respectively (Table S1: doi:10.20348/storedb/1048/1082). Sequence elements predicted to be *PARTICLE* triplex target sites occur on chromosome 16 at fourteen positions in human *WWOX* (chromosome 16: 78099412–79212666; GRCh37) (Fig. 1B, Table S2) and at ten positions in mouse *WWOX* (chromosome 8: 114549301–115290873; GRCm38.p4) (Fig. 1B, Table S3).

### *In silico* determination of *PARTICLE* triplex formation within human genes and promoters

A Triplexator v1.3.2 search revealed 1652 target sites for *PARTICLE* triplex formation with human genomic loci (genes and promoters) distributed throughout all chromosomes (Fig. 1C). Domains within *PARTICLE* were investigated (including 1098 to 1114 nucleotides and 624 to 646 nucleotides) to assess triplex binding capability in the broadest context. The prevalence of these *PARTICLE* triplex sites varied considerably between chromosomes 2 and 16 in comparison to genome wide occurrence (Fig. 1D). Chromosome 2 (harboring the *MAT2A* and *PARTICLE* transcription sites) has a similar density of *PARTICLE* triplex locations as determined for the entire genome (f = 0.52 versus f = 0.53 respectively) (Fig. 1E). Chromosome 16 (harbouring *WWOX*) in comparison to other chromosomes has the highest frequency of *PARTICLE* triplex loci per megabase (f = 0.98) (Fig. 1E). Predicted *PARTICLE* triplex loci per megabase exposed enrichment within human *WWOX* (f = 12.3) (Fig. 1E). These *in silico* observations suggest that *PARTICLE* triplexes potentially occur extensively within the human genome and predominantly within the tumor suppressor *WWOX*.

### *PARTICLE* and *WWOX* interaction is consistent with triplex formation

**Figure 2:**
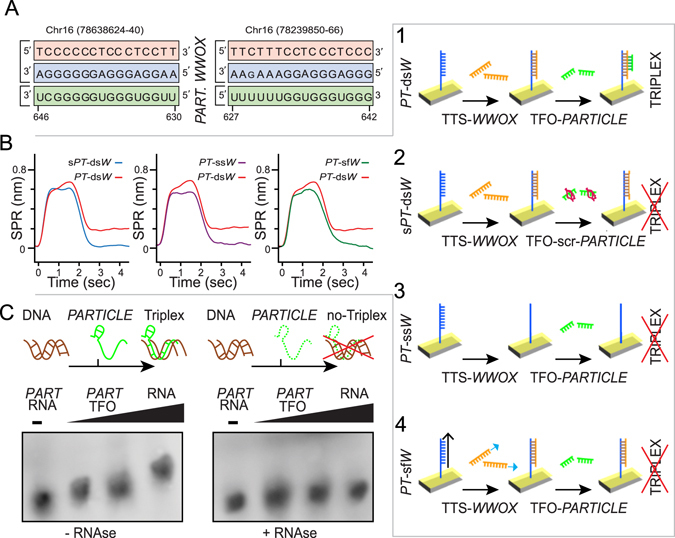
*PARTICLE* forms a triplex with *WWOX*. (**A**) Triplex-forming oligonucleotide motifs within *PARTICLE* (green) identified using Triplexator software to target triplex sites in *WWOX* (blue and red) to form a triple helix. Subscript base indicates a mismatch in the sequence. (**B**) Representative surface plasmon resonance (SPR) sensorgrams illustrating spectral shift following *PARTICLE* triplex formation with the duplex *WWOX* region (*PT*-ds*W*, red line, schematic B1) in comparison to controls (scrambled *PARTICLE* on duplex *WWOX* region; s*PT*-ds*W*, blue line, left, schematic B2); *PARTICLE* on single stranded *WWOX* target (*PT*-ss*W*, purple, middle, schematic B3); *PARTICLE* on duplex shuffled *WWOX* region (*PT*-sf*W*, green, right, schematic B4)), n > 3. (**C**) Electrophoretic mobility shift assays (EMSA) with gels (cropped) showing triplex formation (T) with incremental concentrations (0, 0.2, 2, 20 nM horizontal black triangle) of TFO: *PARTICLE* 627-646 RNA with duplex *WWOX* target region and absence with single *WWOX* (TTS_hit_1) oligo or following treatment with RNase H.

A sequence domain (627–646 nucleotides) identified *in silico* as a site of triplex formation (Fig. 2A) within human *PARTICLE* was shown by surface plasmon resonance (SPR) to interact with *WWOX* (Fig. 2B). Addition of the *PARTICLE*-derived triplex forming oligo (TFO_*PART*_627–646; 400 nM; Table S4) directly to the predicted duplex DNA target from *WWOX* provided a spectral shift of 0.19 ± 0.04 nm (Fig. 2B). Negligible signals (0.02 ± 0.01 nm, p > 0.05) were obtained when this candidate *PARTICLE* sequence interacted with single stranded DNA from the *WWOX* target or a duplex of a shuffled *WWOX* target sequence (Fig. 2B). Addition of a scrambled version of the candidate *PARTICLE* sequence to the predicted duplex *WWOX* target also provided a negligible signal (0.02 ± 0.02 nm, p > 0.05), further confirming the triplex-binding specificity (Fig. 2B, Table S4). The involvement of *PARTICLE* RNA in this triplex formation was also demonstrated through the electrophoretic mobility shifts of the DNA target in the presence of *PARTICLE* and eliminated when the triplex was treated with RNase (Fig. 2C). These findings provide evidence for an interaction that is consistent with triplex formation between *PARTICLE* and *WWOX* at another sequence domain than previously reported for this lncRNA and *MAT2A* ^7^.

### *PARTICLE* influences *WWOX* transcript levels

**Figure 3:**
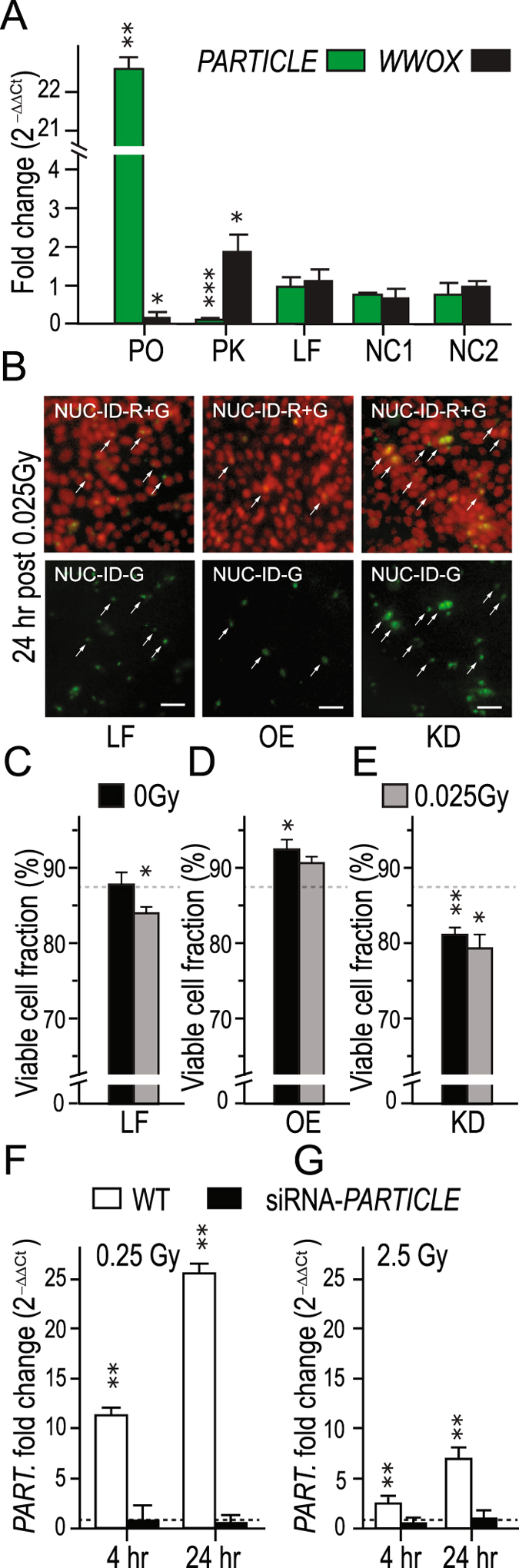
*PARTICLE* influences *WWOX* transcript levels. (**A**) Expression of *PARTICLE* (*green*) or *WWOX* (*black*) in U2OS control cells (transfected with lipofectamine only (LF), *in vitro* control transcript (NC1) or negative siRNA control (NC2)), *PARTICLE* over-expression (PO) or *PARTICLE* knockdown (PK). Values were normalized with the TATA-binding protein (TBP) encoding endogenous gene with relative expression comparison to relevant control. *PARTICLE* over-expression enhances cancer cell line viability. (**B**) Representative epifluorescence micrographs of MDA-MB-361 lipofectamine treated (LF), *PARTICLE* over-expressing (OE) or knockdown (KD) 24 hr post 0.025 Gy identifying viable (red) or non-viable cells (green; lower) using NUCLEAR: ID (NUC-ID; Enzo). Merged images of red and green (R + G) stained nuclei (upper). Scale bar 25 μm. (**C**–**E**) Summary plots illustrating percentage viable cell fraction in LF, OE and KD 24 hr post sham irradiated or 0.025 Gy. Data are represented as mean ± SEM (n = 3) with significance represented by asterisks (p < 0.05) where appropriate. *PARTICLE* response to irradiation exposure is removed after siRNA interference. (**F**,**G**) Expression of *PARTICLE* 4 and 24 hr following 0.25 (**F**) or 2.5 Gy (**G**) irradiation in MDA-MB-361 (negative siRNA (NC2) control transfected (WT: white bars)) or *PARTICLE* siRNA transfected MDA-MB-361 (siRNA-*PART*.: black bars)). Values were normalized with the TBP encoding endogenous gene with relative expression comparison to sham-irradiated (0 Gy) cells (dashed lines).

Upon *PARTICLE* knockdown (97 ± 2% reduction with siRNA n307629, p = 0.0002; 96 ± 1% reduction with siRNA n307634, p = 0.0003) significantly elevated *WWOX* expression (0.7 ± 0.1 fold increase compared to negative siRNA control (NC2), p = 0.03; Fig. 3A; 0.8 ± 0.1 fold increase compared to wild type (WT), p = 0.02; Fig. S2) was noted. Overexpression of *PARTICLE* lead to a 21.2 ± 0.8 fold increase in this lncRNA compared to lipofectamine only or negative transcript control (NC1) (p = 0.002, p = 0.001 respectively; Fig. 3A) with concomitant reduction in *WWOX* expression (p = 0.03, Fig. 3A). The inter-relationship between *PARTICLE* and *WWOX* is revealed here notwithstanding the intronic positioning of triplex binding sites. This suggests that *PARTICLE* may provide a docking platform for modifier proteins that may be far from the gene promoter when considered linearly yet in the realistic coiled genomic setting may be in fact spatially close to the gene promoter.

### *PARTICLE* over-expression enhanced cancer cell line viability

Exposure to very low irradiation (ie. 0.025 Gy) did not activate endogenous *PARTICLE* expression in the breast cancer cell line MDA-MB-361 (Fig. S1) enabling the effects of controlled artificial levels of *PARTICLE* to be evaluated. While the viable cellular fraction was reduced in MDA-MB-361 24 hr post 0.025 Gy exposure (p = 0.046), increased survival (p = 0.02) was noted in sham-irradiated *PARTICLE* over-expressing (OE) cells when compared to lipofectamine-only transfected controls (Fig. 3B–E). In contrast, the viable cell fraction was very significantly compromised upon *PARTICLE* knockdown (p = 0.004) plus 0.025 Gy exposure (p = 0.01) (Fig. 3B–E, Fig. S2). This phenotypic evidence suggests that this breast cancer cell line is influenced by *PARTICLE* transcript levels.

### *PARTICLE* response to irradiation exposure is removed after siRNA interference

Profiling of *PARTICLE* post irradiation of MDA-MB-361 demonstrated a non-linear dose response to radiation extending over the range 0.025 Gy to 5 Gy (Fig. S1). Elimination of increased *PARTICLE* expression 4 hr (p = 0.0018) and 24 hr (p = 0.0036) post low dose (0.25 Gy) irradiation exposure was noted in siRNA transfected MDA-MB-361 (Fig. 3F). Following exposure of these cells to medium dose (2.5 Gy) a similar effect was noted (4hr, p = 0.002; 24hr, p = 0.0024; n = 3; Fig. 3G).

### Increased *PARTICLE* expression post-irradiation with concentration on chromosome 16 harbouring the *WWOX* locus

**Figure 4:**
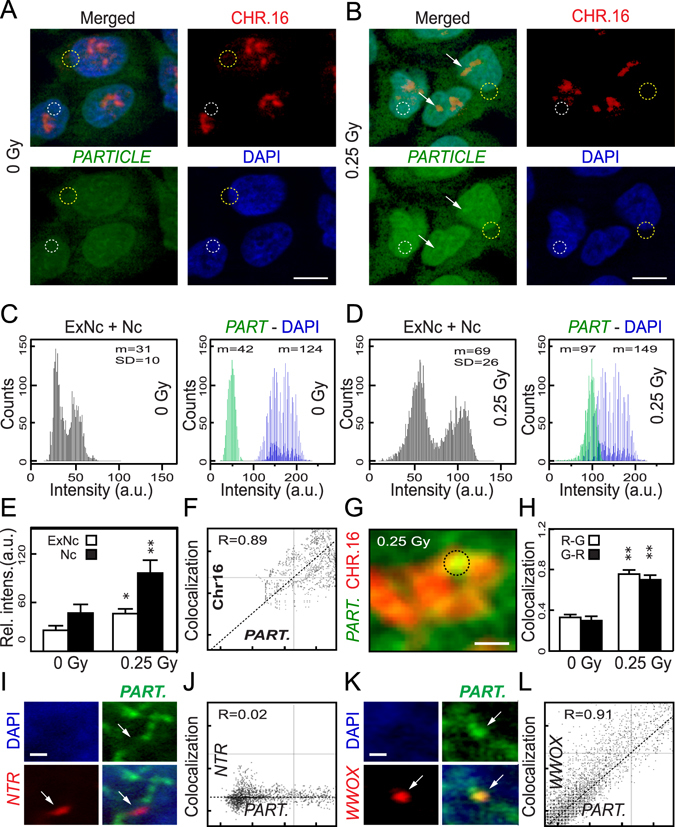
*PARTICLE* transcripts are predominantly nuclear following irradiation and associated with chromosome 16 harbouring *WWOX*. (**A**,**B**) Representative epifluorescence microscopic images of U2OS sham irradiated (left) or 24 hr post 0.25 Gy (right) labelled with chromosome 16 paint (red: upper) and RNA *in-situ* hybridisation probes specific for *PARTICLE* (green: lower). Nuclei stained with DAPI (blue; lower) with merged images (upper). Mean fluorescence intensity analysis determined from regions-of-interest (ROI) indicated by circular dashed lines. Increased intensity over chromosome 16 indicated by arrows. (**C**,**D**) Plots of arbitrary units (AU) of overall *PARTICLE* (*PART*.) fluorescence intensity in the extra-nuclear (ExNc) plus nuclear (Nc) compartments (left) and intracellular distribution (right; green and blue lines represent *PARTICLE* and nuclear fluorescence respectively) in sham-irradiated (**C**) or 24 hr post 0.25 Gy (**D**). (**E**) Summary plots illustrating average relative fluorescence intensities pooled from the selected ROIs from the various experimental groups. (**F**) Scatterplot of arbitrary units (AU) of *PARTICLE* and chromosome 16 co-localization from a ROI (dashed circle), on (**G**) a representative high resolution epifluorescence micrograph after chromosomal spreading. (**H**) Summary co-localization plots for chromosome 16 (ChR = red channel) and *PARTICLE* (ChG = green channel) in ROIs taken from sham irradiated (0 Gy) or 24 hr post 0.25 Gy. Data are represented as mean ± SEM. (**I**) Representative epifluorescence microscopic images of U2OS labelled with RNA *in-situ* hybridisation probes specific for *PARTICLE* (*PART*: pseudo green: upper right) and dual labelled probe specific for a predicted Non-Triplex Region (NTR: pseudo red: lower left). Nuclei stained with DAPI (blue; upper left) with merged images (lower right). Arrow indicates absence of *PARTICLE* at NTR. (**J**) Scatterplot of arbitrary units (AU) of *PARTICLE* (*PART*.) and NTR indicating no co-localization. (**K**) Representative epifluorescence microscopic images of U2OS labelled with RNA *in-situ* hybridisation probes specific for *PARTICLE* (*PART*: pseudo green: upper right) and dual-labelled probe-specific for *WWOX* locus (pseudo red: lower left). Nuclei stained with DAPI (blue; upper left) with merged images (lower right). Arrow indicates presence of *PARTICLE* at *WWOX* locus. (**L**) Scatterplot of arbitrary units (AU) of *PARTICLE* (*PART*.) and *WWOX* indicating strong co-localization.

Our previous study (utilising *in situ* hybridization and confocal microscopy) revealed elevated *PARTICLE* in the nucleus and cytoplasm of the breast cancer cell line MDA-MB-361, 24 hr after low-dose irradiation^7^. Under low dose irradiation conditions in U2OS, we now use intensity profiling analysis of Stellaris probes to reveal an increase in *PARTICLE* expression, with a pre-dominance in the nucleus in comparison to sham irradiated controls (p < 0.05, Fig. 4A,B,D,E, Figs S3 and S4). In the sham-irradiated osteosarcoma cell line U2OS, *PARTICLE* was found in both the extra nuclear and nuclear compartments (mean intensity units = 31 ± 10; Fig. 4C). Nevertheless, a 58 ± 3% augmentation in the level of cytosolic *PARTICLE* transcripts in U2OS was also noted at this time post 0.25 Gy exposure (Fig. 4E). Following irradiation there was an indication that increased *PARTICLE* might localise to chromosome 16 as noted in the nuclei of U2OS cells stained with a pan-chromosome 16 paint (MetaSystems, Fig. 4B) and with fluorescently labeled complementary FAM probes to *PARTICLE*. Chromosome spreading enabled high resolution confocal imaging of *PARTICLE* on chromosome 16 (Fig. 4G). A strong co-localization signal between *PARTICLE* and chromosome 16 was detected, particularly after low dose irradiation exposure compared to sham-irradiated controls (p < 0.005; Fig. 4F–H). Direct interaction was confirmed on chromosome spreads from low dose exposed U2OS (24 hr post 0.25 Gy irradiation) using dual labelled fluorescence intronic probes specific for *WWOX* (R = 0.91; for sequence see methods) in contrast with chr.11: 108106358–108106457 (a genomic region not predicted to be involved in *PARTICLE* binding, R = 0.018) (Fig. 4I–L). This suggests that *PARTICLE* might be associated with *WWOX* through triplex binding as part of the cellular response to low dose irradiation exposure.

### Predicted *PARTICLE* triplex binding sites predominate within the extracellular signal-regulated protein kinase (ERK) pathway

**Figure 5:**
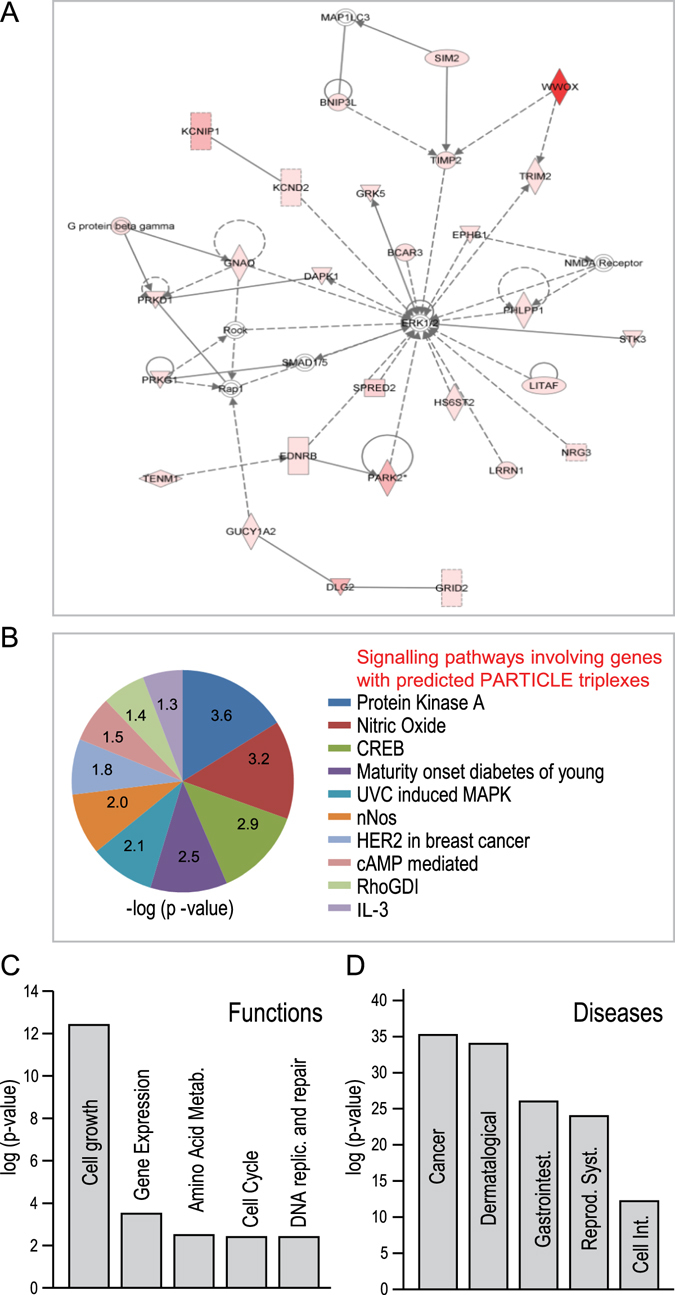
Ingenuity® Analysis™ software (www.ingenuity.com) used in the analysis of genes with predicted *PARTICLE* triplex binding sites (Fig. S1 Triplex Domain Finder) associated with signaling pathways, molecular functions and diseases. (**A**) Graphical representation of genes with predicted *PARTICLE* triplex binding sites (pink) associated with the ERK 1/2 regulatory network. The predominance of triplexes within *WWOX* is shown in red. (**B**–**D**) Significantly associated signaling pathways (**B**), molecular functions (**C**) and diseases (**D**) involving genes with predicted *PARTICLE* triplexes. Pie chart (**B**) or histograms (**C**,**D**) of associated –log of the calculated p-value whereby a significant p value of p < 0.05 is equivalent to −log = 1.3.

Genomic *PARTICLE* triplex binding sites obtained from Triplex Domain Finder (Table S1) were analysed via INGENUITY (http://www.INGENUITY.com) in the context of direct plus indirect biological pathway association and functional interaction. The ERK 1/2 pathway was found to be the predominant enriched network amongst the set of genes containing predicted *PARTICLE* triplexes with *WWOX* operating as the principal influence (Fig. 5A). Further signaling pathways included the contribution of protein kinase A, nitric oxide and CREB etc. (Fig. 5B).

### Molecular functions and associated diseases

Many of the genes with predicted *PARTICLE* triplex sites were associated with cell growth, gene expression, amino acid metabolism, cell cycle and DNA replication and repair (Fig. 5C). The analysis showed cancer to be the predominant significantly associated disease (Fig. 5D).

## Discussion

LncRNAs are comprised of motifs (eg. polypurine bases) that appear important for chromatin interaction^20^ and sequences possibly acting as spacers that link functional modules^3^. While sequence conservation is limited between orthologous lncRNAs^21^, we show that human *PARTICLE* shares considerable secondary structural features with its positional equivalent mouse ortholog. Moreover, we detect common *PARTICLE* binding site enrichment for mouse and human at the *WWOX* locus. This study also reveals the predicted widespread formation of *PARTICLE* triple helixes across every chromosome in both human and mouse genomes.

After exposure to low dose irradiation, we demonstrate that human *PARTICLE* predominates in the nucleus in U2OS with an increase in chromosome 16 association after low dose irradiation. Co-localization of *PARTICLE* with the *WWOX* locus in irradiated cells is illustrated. *PARTICLE* knockdown resulted in increased *WWOX* transcript levels in the breast cancer cell line MDA-MB-361. Others have shown reduced levels of full-length *WWOX* or alternative transcript isoforms for this gene in breast cancer cell lines^22^. In cancer cells, WWOX is decreased or absent in most cases compared to a normal cell state, suggesting the functions of WWOX are hampered to permit the onset of malignant trans-formations^23, 24^. Here we report increased survival of MDA-MB-361 in *PARTICLE* over-expressing (OE) cells while the viable cell fraction was very significantly compromised upon *PARTICLE* knockdown. As the physiological role of WWOX remains to be better defined, a ‘guilt by association’ principle has been employed by others to identify signaling pathways associated with WWOX expression^25, 26^. The WWOX protein contains two functional domains predicted to carry out NADP(H)-dependent dehydrogenase reactions and oxidoreductase activity^25^ with the latter represented in the set of proteins deregulated by irradiation (Fig. S5).

INGENUITY analysis of human genes with predicted *PARTICLE* triplex binding sites indicated the predominance of the ERK 1/2 signaling pathway operating via WWOX. Ionizing radiation induces activation of MAPK family members, including ERK 1/2^27, 28^ whose activation has been shown to play an important role in promoting cell survival^29, 30^. The rapid activation of HER2 receptors following ionizing radiation contributes to ERK 1/2 signaling activation in cancer cells of the breast^31^. While the exact mechanisms responsible for the activation of ERK 1/2 signaling by radiation has not yet been clearly elucidated, it is tempting to speculate that *PARTICLE* triplex formation has an influence on several signaling mechanisms such as those involving CREB and MAP kinase proposed to be involved in this activation.

Remarkable conservation in *WWOX* intronic sequences has been speculated to reflect the significant evolutionary selection pressure existing to preserve the non-coding regions of the *WWOX* locus^32^. MicroRNAs have already been implicated in this phenomenon^33^ with lncRNA involvement no doubt probable given the prevalence of *PARTICLE* triplex sites within *WWOX* introns and indeed potentially genome wide. While direct NMR structural evidence remains to be obtained for definitive proof, *PARTICLE* triplex formation was previously suggested to be a singular event, serving to reign in the activity of its neighboring gene *MAT2A* ^7^. Our results now reveal that *PARTICLE* may cast its triplex net across every human chromosome. In keeping with the recognized role of lncRNAs in genomic architectural regulation^34^ and given the interaction between *PARTICLE* and SUZ12^7^, it is tempting to speculate that *PARTICLE* binding provides an epigenetic modifying platform to control chromatin structure on chromosome 16 at the *WWOX* locus *etc*. Others have revealed five potential triple helix forming domains within *HOTAIR* ^35^ which are predicted to be involved in recruitment of Polycomb^20^. It is now recognized that PRC2 targets RNA through recognition of short repeats of consecutive guanines^36^. The natural occurrence of this motif has been suggested to explain promiscuous RNA binding by PRC2^36^. While it was proposed over 20 years ago that RNA sequences are not tolerated in purine motif triple helices^37^, other authors more recently have demonstrated that the lncRNA *MEG3* ^9^ and microRNAs^38^ are capable of triple helical formation with purine motifs. We propose that *PARTICLE* serves as an lncRNA-directed component of a genomic ‘zip code’ potentially guiding modifiers to pertinent chromatin locations for gene regulation. Given the enrichment of *PARTICLE* binding sites within the tumor suppressor *WWOX*, time has come to understand the disease implications of triplex formation between emerging lncRNAs and the human genome.

## Materials and Methods

### Secondary structure prediction and consensus pairing of *PARTICLE* in human and mouse

The *PARTICLE* nucleotide sequences (human *PARTICLE*: NR_038942.1; mouse *PARTICLE*: 4930414L22Rik) were uploaded in FASTA format onto the RNAfold webserver (http://rna.tbi.univie.ac.at/). The minimum free energy (MFE) prediction was calculated for each species as previously reported^39^ and color annotated secondary structure drawings generated using equilibrium base pairing probability measurements. A local consensus RNalifold structure was generated from aligned human *PARTICLE*: NR_038942.1 and mouse *PARTICLE*: 4930414L22Rik sequences using LocARNA (http://rna.informatik.uni-freiburg.de)^18^. Compatible base pairs are colored in accordance with the LocARNA online color index. The Expect (E) value parameter was utilized to determine significance *ie*. E value = 1 assigned to a hit can be interpreted as an expectation to see 1 match with a similar score simply by chance. The lower the E-value, or the closer it is to zero, the more significant the match^40^.

### Triplexator and Triplex Domain Finder

Triplexator and Triplex Domain Finder are computational frameworks^41^ for the *in silico* prediction of triplex structures. Triplex (nucleic acid triple helices) formation is governed by sequence-specific binding rules. The triplex-forming oligonucleotides (TFO) are located in the region of the single stranded nucleotide (RNA) capable of forming Hoogsteen (or reverse) bonds with the duplex^42^. The triplex target site (TTS) is defined as the polypurine-polypyrimidine tract of a duplex (DNA) capable of accommodating the TFO. An *in silico* triplex search was performed using Triplexator v1.3.2 with the *PARTICLE* RNA and selected regions of the human genome (UCSC assembly hg19; promoter and gene associated regions) as single stranded and double stranded inputs, respectively, employing parameters: a maximal error rate = 10, number of consecutive matches in a feature = 10, a minimum triplex length of 15 bp, a maximum triplex length of 30 bp, tolerated number of consecutive errors = 1. Triplexator software can be accessed using the virtual machine (http://bioinformatics.org.au/tools/triplexator/inspector/vm_guide.html). Assessment of the triplex target sites number/gene was determined followed by abundance sorting.

Triplex Domain Finder (TDF) was utilized for a more extensive search to determine whether *PARTICLE* could form triplexes extensively throughout the mouse (GRCm38.p4) and human (GRCh37/hg19) genomes. The parameters utilized were similar to those for Triplexator but also included the following: cut off value for RNA accessibility = 500.

### Surface plasmon resonance (SPR) assay

SPR sensor chips were fabricated by thermal deposition of titanium (5 nm) and gold (50 nm) onto a glass chip. Sensor chips were rinsed sequentially with hot acetone, ethanol (100%), dH_2_O and dried under a flow of nitrogen gas. Subsequently, chips were dipped in solution (70% H_2_SO_4_–30% H_2_O_2_), rinsed with dH_2_0 and dried under flow of nitrogen gas. Following this step, the chips were placed for 1 hr in a solution containing thiol-modified DNA receptor (TTS_*WWOX*_a_2 or TTS_WWOX_a_shuffled_1 (2 μM); Table S4) and mercaptohexanol (MCH (400 nM)) in SSC (5 X) buffer (NaCl (750 mM), Na citrate (75 mM, pH 7). The thiol on the DNA receptor linker enables this DNA molecule to anchor to the gold surface, while the MCH is employed as a lateral spacer to improve target accessibility. Detection of sample was done at a constant flow (13 μl/min) of running buffer (HEPES (50 mM), MgCl_2_ (10 mM)) by initially injecting duplex forming oligo (TTS_*WWOX*_a_1 or TTS_*WWOX*_a_shuffled_2 (200 nM), Table S4) prepared in SSC (5 X) buffer followed by triplex forming oligo candidate (TFO_*PARTICLE*_627-646_DNA or scrambled_*PART*. (400 nM), Table S4) prepared in HEPES (50 mM) and MgCl_2_ (10 mM). Specificity of triplex detection was verified by injecting TFO_*PARTICLE*_627-646 (400 nM) in the absence of previous duplex formation and by injecting a scrambled *PARTICLE* sequence (scrambled_*PART*; 400 nM, Table S4) over the duplex target or shuffled sequence from *WWOX*. All sensorgrams were subjected to zeroing. All experiments were performed six times.

### Electrophoretic mobility shift assay (EMSA) for *PARTICLE*: *WWOX* triplex determination

EMSA oligos (TTS_hit_1: DNA, TTS_hit_2: DNA and TFO_*PART*_627-646_RNA; Table S4) were commercially synthesized (Eurofins, Germany). The procedure was carried out in accordance with instructions in the LightShift Chemiluminescent EMSA kit (ThermoScientific, cat # 20148). Binding reactions containing incremental amounts (0 nM, 0.2 nM, 2 nM or 20 nM) of TFO_*PART*_627-646_RNA were denatured at 70 °C for 10 min and slowly cooled to room temperature. To determine the dependency of triplex formation on the added RNA, control reactions were run in parallel that were subsequently treated with RNase H (0.5 U, Thermo Scientific cat # EN0201) at 37 °C for 1 hr. High-density TBE loading sample buffer (5 X, Thermo Scientific, cat # LC6678) was added to samples with electrophoresis through pre-run 6% DNA retardation gels (Thermo Scientific, cat # EC6365BOX) with transfer onto Biodyne positively charged nylon membrane (Thermo Scientific, cat # QK225019C) and chemiluminescent detection using reagents supplied with the LightShift EMSA kit (Thermo Scientific, cat # 20148) and image acquisition using an Alpha Innotech gel imager (Fluor Chem HD2, BioZym).

### Propagation and maintenance of cell lines

MDA-MB-361 (American Type Culture Collection (ATCC)) was cultivated as previously described^43^. U2OS (ATCC) was grown under similar conditions except Roswell Park Memorial Institute (RPMI) 1640 media (GibcoTM cat # 21875-034) and FBS (10%) was utilized. The identity of all cell lines was established by microsatellite analysis (Forensik GmbH, Germany). All cultures were routinely checked for mycoplasma contamination using a MycoAlert mycoplasma detection kit (Lonza, cat. # LT07-218). In general, cells were grown to 80% confluency prior to removal from the dish using trypsin (0.25%)/EDTA (0.02%) for sub-culturing or harvesting.

### Irradiation

All irradiations were performed using a closed HWM-D 2000 Cesium^137^ source (Wälischmiller Engineering GmbH, Markdorf, DE; 10 cm height, 33 cm diameter) at a dose rate of 0.0082 Gy/sec. For very low dose irradiation exposures the tissue culture dishes were placed into a lead box within the irradiation chamber causing a 10 fold reduction in the dosage rate. Cells were exposed to 0.0025, 0.025, 0.25, 2.5 or 5 Gy. Sham irradiation of controls involved only transport to the irradiation facility. Annual calibration was performed by the Helmholtz Zentrum Munich, DE with reference to standards established by the National Physical Laboratory (U.K).

### RNA interference targeting the *PARTICLE* triplex

*PARTICLE* knockdown was undertaken with Silencer Select siRNA interference technology (siRNA id: n307629; Part # 4390771 or siRNA id: n307634; Part # 4390771). MDA-MB-361 cells were grown to 60% confluence and transfected with these siRNAs (10 nM) using lipofectamine as per manufacturer instructions. After 72 hr, cells were irradiated at 0.25 Gy, 2.5 Gy or sham-irradiated (0 Gy). Control conditions included sham irradiation plus transfection with lipofectamine and/or negative siRNA (NC2; cat # AM4615 no.3, Thermo Scientific). RNA extraction was performed 4 hr and 24 hr post irradiation (or sham irradiation).

### *PARTICLE* overexpression

*PARTICLE* was cloned into the pGEM - T vector (p.*PART*) (GenScript) and transformed into Top10 bacteria. A colony was grown in ampicillin (100μg/ml) overnight and plasmid midiprep (Promega) performed. Plasmid concentration and purity was assessed (NanoDrop 1000, Thermo Scientific) with A260/280 ratio determination with automated sequence validation (GenScript). Plasmid linearization was carried out using 1μg plasmid DNA and S*ac*I overnight digestion at 37 °C. *PARTICLE* (1432 nucleotides) was *in vitro* transcribed from a pGEM - T vector (GenScript) using the TranscriptAid T7 High Yield transcription kit (Thermo Scientific, cat # K0441). Transcripts were treated with RNase-free DNase 1 (Thermo Scientific) and purified using an RNeasy mini-elute clean-up kit (Qiagen, cat # 74204) and verified by TBE-agarose (1.8%) electrophoresis. Prior (24 hr) to transfection, MDA-MB-361 or U2OS were seeded (10^5^ cells/35mm dish) in growth media (described above) in the absence of antibiotic/anti-mycotic to ~70% confluence at the time of transfection. The control template included in the Transcript T7 High Yield Transcription kit (Thermo Scientific, cat # K0441) as utilized for the production of a 2223 nucleotide ‘run off’ transcript serving as a negative control (NC1) for over-expression studies. Cells were transfected with lipofectamine and *PARTICLE* (4 μg) or negative control (4 μg) as per standard conditions with incubation for 72 hr prior to irradiation exposure.

### Cell viability

MDA-MB-361 were grown to ~70% confluence/well on 6-well plates and transfected with *PARTICLE* transcript for overexpression or with siRNA for *PARTICLE* knockdown or relevant controls (NC1 or NC2) as outlined above. Growth medium was removed and replaced with RPMI-Medium (cat. # E15-848, PAA). Cell viability analysis was performed using the Nuclear ID Blue/Green cell viability reagent (cat. # ENZ-53006-C100, Euro Life Science International) added to the growth medium (1:1000 dilution). This is a mixture of a blue fluorescent cell-permeable nucleic acid staining dye and a green fluorescent cell-impermeable nucleic acid staining dye for identifying dead nuclei. Following incubation for 30 min in the tissue culture incubator with protection from light, the staining solution was removed. Cell survival/viability was visualized using a Keyence confocal microscope (#BZ-9000, Biorevo) with associated BZ II viewer software.

### RNA isolation and cDNA synthesis

Total RNA was isolated from cell lines and purified using TriFast peqGOLD (Peqlab, cat # 30–2010) and a Maxwell 16 LEV Blood DNA kit (Promega cat # AS1290) with solution substitution (ie. isopropanol replacement by 100% ethanol in cartridge number 1) and Maxwell 16 machine (Promega). Final elution was in ultra-pure water with concentration and purity assessment using O.D. 260/280 ratio determination (NanoDrop 1000, Thermo Scientific). Total RNA was stored at −80 °C. Total RNA (1μg) from sham-irradiated and irradiated cells was converted into first strand cDNA using standard protocol procedures (with the inclusion of random hexamers and oligo dT primers) and reagents from Life Technologies, Germany.

### Real time PCR quantification

*PARTICLE* and *TBP* primers and probes^7^ or pre-designed *WWOX* (Hs00249590_ml: assay spanning the exon 1–2 boundary) single Taqman gene expression assay (ThermoFisher cat # 4331182) were utilized for gene expression determination. The reaction conditions for single gene assays were as such: cDNA (50–100 ng), 1x Taqman universal PCR master mix (no AmpErase UNG; Life Technologies, cat. # 4324018), forward and reverse primers (10 pmol), specific fluorescent probe (5 pmol), nuclease-free water up to 25 μl. For pre-designed assays similar conditions were used except for assay mix (1X) instead of individual primers and probes. Cycle threshold values were extracted and fold changes in gene expression determined by 2^(−∆∆Ct)^ ^44^. Negative controls were normalized to a value = 1 with test samples relatively compared.

### *PARTICLE in situ* hybridization, chromosome painting and microscopic analysis

Procedures were followed as previously reported^7^ and in accordance with Stellaris fluorescence *in situ* hybridization (FISH) (Biosearch Technologies) website information (www.biocat.com). Using the online probe designer tool (www.biosearchtech.com/stellarisdesigner/), 40 specific probes were selected from an input sequence (*PARTICLE* NR_038942.1) for optimal binding properties to the target RNA sequence. Search parameters were selected that included a masking level (3–5), maximum probe coverage = 40 and minimum 2 nucleotides spacing level. The probe fluorophore 5′carboxyfluorescein FAM (Excitation (Ex): 495 nm; Emission (Em): 520 nm) was used for *PARTICLE* detection. Chromosome 16 was visualized using XCyting whole chromosome paints (MetaSystems, cat # D-0316-100-OR) labelled with Texus Red in accordance with manufacturer’s instructions. Cells were mounted in VECTASHIELD™ HardSet™ containing the nuclear counterstain DAPI (Vector lab, cat # H-1500). For greater resolution, chromosomes were isolated from U2OS according to the online protocol^45^.

A dual labelled probe was designed for a 97 bp *WWOX* specific intronic sequence (chr 16: 78638458–78638554) directly upstream of a predicted *PARTICLE*-binding rich region as follows: FAM 5′GTATCAATTTTAAAGTATTTCTTTTAGGATTCTATTATTTACCCTTTTTCTTCAATGTTAAATACCATTTCCTTTAATAAAGATTTAAAAAAGATTC3′ C3-Fluorescein. A dual labelled probe was designed for a 100 bp negative control region not predicted to contain *PARTICLE* triplex binding sites *ie*. ‘Non-Triplex predicted Region (NTR) (chr11: 108106358–108106457) as follows: FAM 5CAAATTTATGTTTTTCTTTATTTGTTTATTTTGAAATAGGAGCACCTAGGCTAAAATGTCAAGAACTCTTAAATTATATCATGGATACAGTGAAAGATTC3′ C3 Fluorescein. The dual labelled probes (50 nmol synthesis scale, HPLC purity) were re-suspended in 200 μl nuclease-free dH_2_O. The *in situ* hybridization protocol (as indicated above) was utilized for detection of specific genomic regions on isolated chromosomes.

Epifluorescence microscopic imaging was performed on an Axiovision microscope equipped with green fluorescent protein (GFP; Ex: 488 nm; Em: 509 nm) and tetramethylrhodamine isothiocyanate (TRITC; Ex: 550 nm; Em: 570 nm) filter sets for visualization of labelled *PARTICLE* and chromosome 16 respectively. Emitted fluorescence signals were sampled at a resolution of 30 nm/pixel with a dwell time of 1.5 μs. Co-localization of FAM and C3 fluorescein labelled probes was defined by the presence of these two labels in the same pixel in the digitally acquired images from 10 regions of interest, using a co-localization algorithm (Zen 2008, Carl Zeiss). Separation of emission spectra was ensured with appropriate cut-off filters (green 492–590 nm; red 585–734 nm). Cells with fluorescence signal intensity exceeding three times the average intensity of the background were considered suitable for inclusion in the analysis^43^. Mean numerical values of fluorescence intensity were normalized over regions of interest (ROI) pooled from at least 25 frames, collected from 20–30 cells for each experimental condition. These were tabulated and analyzed using Zen 2008 and Excel analytical software.

### Statistical Analysis

Values in the text are expressed as the mean ± S.E.M., and n refers to the number of independent biological replicated data. Triplex t statistic was tested using hypothesis parameters (HA: μT–μC > 0) and Origin 7 software. Triplex Frequency distribution was evaluated by Microsoft Excel. Differences between means were tested using the Student’s t-test with p values < 0.05 taken to indicate statistical significance. Co-localization testing was assessed using FIGI software (NIH).

### PANTHER bioinformatic meta-analysis of radiation studies

To examine the proportion of oxidoreductase related proteins altered by low to medium dose irradiation in proteomics data, we performed enrichment analysis on the significantly deregulated proteins in our previously published studies using the PANTHER bioinformatics tool (http://www.pantherdb.org). Data sets were searched using the following Gene Ontology (GO) terms: molecular function and oxidoreductase activity (GO Accession: 0016491). The percentage of proteins with oxidoreductase activity was calculated taking account of all significantly deregulated proteins applied to the PANTHER software.

### INGENUITY pathway analysis (IPA)

IPA was utilized (http://www.ingenuity.com) to analyze the dataset related to TDF predicted *PARTICLE* triplex binding sites in the human genome (Table S1). IPA enabled the visualization of changed molecular functions and disease risk with associated significance expressed as −log of the calculated p-value (p < 0.05 equivalent to −log = 1.3).

## Electronic supplementary material

Supplementary information

Supplementary Dataset 1
