# Supplementary material for: PARTICLE triplexes cluster in the tumor suppressor WWOX and may extend throughout the human genome
Source: Sci Rep. 2017 Aug 2;7:7163. doi: 10.1038/s41598-017-07295-5 (PMC5541130; doi:10.1038/s41598-017-07295-5)
Supplement: Supplementary file 1 — Supplementary information [file 41598_2017_7295_MOESM1_ESM.pdf]

Supplemental Information

PARTICLE triplexes cluster in the tumor suppressor *WWOX* and may extend throughout the human genome.

Valerie Bríd O'Leary, Jan Smida, Fabian Andreas Buske, Laura Garcia Carrascosa, Omid Azimzadeh, Doris Maugg, Sarah Hain, Soile Tapio, Wolfgang Heidenreich, James Kerr, Matt Trau, Saak Victor Ovsepian, Michael John Atkinson.

Table S1 is relevant to Fig. 1. http://www.rbstore.eu/ id: 1048 and supplementary dataset file. Nucleotide sequence location for triplex binding sites between *PARTICLE* and the human genome (hg19) as predicted by Triplex Domain Finder software (www.regulatory-genomics.org/tdf) [1]. Regions with a second associated gene highlighted in blue.

<table>
<tr><th>PARTICLE Triplex* formation site in WWOX</th><th>Intragenic Region</th><th>PARTICLE Triplex region</th><th>Triplex RNA Strand</th><th>Triplex sequence along Hoogsteen face</th><th>PARTICLE Orientation</th></tr>
<tr><td>Chr.16 : 78912951 - 67</td><td>Intron 8</td><td>1098 - 1114</td><td>plus</td><td>TFO: 3'- AGGGuGGAAAGGAGGA -5'
||||*|||||||||||
TTS: 5'- AGGGAGGAAAGGAGGA -3'
3'- TCCCTCCTTTCCTCCT -5'</td><td>AP</td></tr>
<tr><td>Chr.16 : 78638579 - 95</td><td>Intron 8</td><td>630 - 646</td><td>minus</td><td>5'- TCCCCCCTCCCTCCTT -3'
TTS: 3'- AGGGGGGAGGGAGGAA -5'
|*||||||||||||||
TFO: 3'- UcGGGGGUGGGUGGUU -5'</td><td>P</td></tr>
<tr><td>Chr.16 : 78638594 - 610</td><td>Intron 8</td><td>630 - 646</td><td>minus</td><td>5'- TCCCCCCTCCCTCCTT -3'
TTS: 3'- AGGGGGGAGGGAGGAA -5'
|*||||||||||||||
TFO: 3'- UcGGGGGUGGGUGGUU -5'</td><td>P</td></tr>
<tr><td>Chr.16 : 78638609 - 25</td><td>Intron 8</td><td>630 - 646</td><td>minus</td><td>5'- TCCCCCCTCCCTCCTT -3'
TTS: 3'- AGGGGGGAGGGAGGAA -5'
|*||||||||||||||
TFO: 3'- UcGGGGGUGGGUGGUU -5'</td><td>P</td></tr>
<tr><td>Chr.16 : 78638624 - 40</td><td>Intron 8</td><td>630 - 646</td><td>minus</td><td>5'- TCCCCCCTCCCTCCTT -3'
TTS: 3'- AGGGGGGAGGGAGGAA -5'
|*||||||||||||||
TFO: 3'- UcGGGGGUGGGUGGUU -5'</td><td>P</td></tr>
<tr><td>Chr.16 : 78638639 - 55</td><td>Intron 8</td><td>630 - 646</td><td>minus</td><td>5'- TCCCCCCTCCCTCCTT -3'
TTS: 3'- AGGGGGGAGGGAGGAA -5'
|*||||||||||||||
TFO: 3'- UcGGGGGUGGGUGGUU -5'</td><td>P</td></tr>
<tr><td>Chr.16 : 78638654 - 70</td><td>Intron 8</td><td>630 - 646</td><td>minus</td><td>5'- TCCCCCCTCCCTCCTT -3'
TTS: 3'- AGGGGGGAGGGAGGAA -5'
|*||||||||||||||
TFO: 3'- UcGGGGGUGGGUGGUU -5'</td><td>P</td></tr>
<tr><td>Chr.16 : 78638669 - 85</td><td>Intron 8</td><td>630 - 646</td><td>minus</td><td>5'- TCCCCCCTCCCTCCTT -3'
TTS: 3'- AGGGGGGAGGGAGGAA -5'
|*||||||||||||||
TFO: 3'- UcGGGGGUGGGUGGUU -5'</td><td>P</td></tr>
<tr><td>Chr.16 : 78638684 - 700</td><td>Intron 8</td><td>630 - 646</td><td>minus</td><td>5'- TCCCCCCTCCCTCCTT -3'
TTS: 3'- AGGGGGGAGGGAGGAA -5'
|*||||||||||||||
TFO: 3'- UcGGGGGUGGGUGGUU -5'</td><td>P</td></tr>
<tr><td>Chr.16 : 78239850 - 66</td><td>Intron 5</td><td>630 - 646</td><td>minus</td><td>5'- TTCTTTCCTCCCTCCC -3'
TTS: 3'- AAGAAAGGAGGGAGGG -5'
||*|||||||||||||
TFO: 5'- UUUUUUGGUGGGUGGG -3'</td><td>P</td></tr>
<tr><td>Chr.16 : 78239867 - 83</td><td>Intron 5</td><td>627 - 642</td><td>minus</td><td>5'- TTCTTTCCTCCCTCCC -3'
TTS: 3'- AAGAAAGGAGGGAGGG -5'
||*|||||||||||||
TFO: 5'- UUUUUUGGUGGGUGGG -3'</td><td>AP</td></tr>
<tr><td>Chr.16 : 78832453 - 69</td><td>Intron 5</td><td>628 - 644</td><td>minus</td><td>5'- TATTCCTCCCTCCCCC -3'
TTS: 3'- AtAAGGAGGGAGGGGG -5'
|*||||||||||||||
TFO: 5'- UUUUGGUGGGUGGGGG -3'</td><td>AP</td></tr>
<tr><td>Chr.16 : 79031539 - 55</td><td>Intron 8</td><td>627 - 642</td><td>minus</td><td>5'- TTATTTCCTCCCTCCC -3'
TTS: 3'- AAtAAAGGAGGGAGGG -5'
||*|||||||||||||
TFO: 5'- UUUUUUGGUGGGUGGG -3'</td><td>AP</td></tr>
<tr><td>Chr.16 : 79156490 - 506</td><td>Intron 8</td><td>627 - 642</td><td>minus</td><td>5'- TTCTTTCCTCCCTCCC -3'
TTS: 3'- AAGAAAGGAGGGAGGG -5'
||*|||||||||||||
TFO: 5'- UUUUUUGGUGGGUGGG -3'</td><td>AP</td></tr>
</table>

Table S2 is relevant to Fig. 1. Genomic nucleotide sequence location for triplex formation between *PARTICLE* and human *WWOX* as predicted by Triplexator v1.3.2. *Fraction of mismatches in the triplex = 0.062. AP = Anti-parallel; P = parallel, GRCh37/hg19 assembly.

<table>
<tr><th>Mouse PARTICLE Triplex* formation site in WWOX</th><th>Intragenic Region</th><th>Mouse PARTICLE Triplex region</th><th>Triplex RNA Strand</th><th>Triplex sequence along Hoogsteen face</th><th>Mouse PARTICLE Orientation</th></tr>
<tr><td>Chr. 8 : 114549301 – 17</td><td>Intron 5</td><td>1590 – 1606</td><td>minus</td><td>5'- CCTCCCCCCTCCCTTCC -3'
TTS: 3'- GGAGGGGGGAGGGAAGG -5
|||||||||||*|||||
TFO: 5'- CCUCCCCCCUCGCUUCC -3'</td><td>P</td></tr>
<tr><td>Chr. 8 : 114778642 – 58</td><td>Intron 8</td><td>1590 - 1606</td><td>plus</td><td>TFO: 5'- GCCUCCCCUCCCCCCU -3'
||||||||||||||||
TTS: 5'- GGGAGGGGAGGGGGGT -3'
3'- CCCTCCCCTCCCCCCA -5'</td><td>P</td></tr>
<tr><td>Chr. 8 : 114854006 – 26</td><td>Intron 8</td><td>625 – 645</td><td>minus</td><td>5'- CCCTCCTCCCTTCTCTCCTCC -3'
TTS: 3'- GGGAGGAGGGAAGAGAGGAGG -5'
|||||||*|||||*|||||||
TFO: 5'- CCCUCCGCCCUUCCCUCCUCC -3'</td><td>P</td></tr>
<tr><td>Chr. 8 : 115003654 – 70</td><td>Intron 8</td><td>1590 – 1606</td><td>minus</td><td>5'- CCTCCTCCCTCCCCTCC -3'
TTS: 3'- GGAGGAGGGAGGGGAGG -5'
|||||*|*|*|||||||
TFO: 5'- CCUCCCCUCCCCCCUCC -3'</td><td>P</td></tr>
<tr><td>Chr. 8 : 115048864 – 80</td><td>Intron 8</td><td>1590 – 1606</td><td>minus</td><td>5'- CCTCTCCCCTCCCCTCC -3'
TTS: 3'- GGAGAGGGGAGGGGAGG -5'
||||*||*|*|||||||
TFO: 5'- CCUCCCCUCCCCCCUCC -3'</td><td>P</td></tr>
<tr><td>Chr. 8 : 115048889 - 05</td><td>Intron 8</td><td>1590 – 1606</td><td>minus</td><td>5'- CCTCTCCCCTCCCCTCC -3'
TTS: 3'- GGAGAGGGGAGGGGAGG -5'
||||*||*|*|||||||
TFO: 5'- CCUCCCCUCCCCCCUCC-3'</td><td>P</td></tr>
<tr><td>Chr. 8 : 115064168 – 86</td><td>Intron 8</td><td>509 – 527</td><td>plus</td><td>TFO: 3'- AAGGGGGAGGGGAGCGGG -5'
|*||||||||||||*||*
TTS: 5'- AGGGGGGAGGGGAGGGGA -3'
3'- TCCCCCCTCCCCTCCCCT -5</td><td>A</td></tr>
<tr><td>Chr.8 : 115067629 – 46</td><td>Intron 8</td><td>628 – 645</td><td>plus</td><td>TFO: 5'- TCCGCCCUUCCCUCCUCC -3'
||||||||||||||||
TTS: 5'- GGGAGGGAAGGGAGGAGG -3'
3'- CCCTCCCTTCCCTCCTCC -5'</td><td>P</td></tr>
<tr><td>Chr.8 : 115179334 – 50</td><td>Intron 8</td><td>1590 – 1606</td><td>plus</td><td>TFO: 5'- GCCUCCCCUCCCCCCUC -3'
|||||||||||||||
TTS: 5'- GGGAGGGGAGGGGAGAG -3'
3'- CCCTCCCCTCCCCTCTC -5'</td><td>P</td></tr>
<tr><td>Chr.8 : 115290859 – 75</td><td>Intron 8</td><td>504 – 520</td><td>plus</td><td>TFO: 3'- GGGAAAGGGGGAGGGAG -5'
|||**||||||||*|||
TTS: 5'- GGGGGAGGGGGAGAGAG -3'
3'- CCCCCTCCCCCTCTCTC -5'</td><td>A</td></tr>
</table>

Table S3 is relevant to Fig. 1. Genomic nucleotide sequence location for triplex formation between *PARTICLE* and mouse *WWOX* as predicted by Triplexator v1.3.2. *Maximum total error = 3. AP = Anti-parallel; P = parallel, GRCm38.p4 assembly.

Short Name	Description	Sequence
TTS_*WWOX*_a_2	SPR thiol modified receptor oligo matching the antiparallel triplex target site of *WWOX*.	SH-5’-TTTTTTTTTTTTTTTTTCTTTCCTCCCTCCC-3’
TTS_hit_2	EMSA oligo matching the antiparallel triplex target site of *WWOX.*	5'- TTCTTTCCTCCCTCCCCCGA-3'
TTS_*WWOX*_a_1	SPR duplex forming oligo complementary to TTS_*WWOX*_a_2.	5’-GGGAGGGAGGAAAGAA-3’
TTS_hit_1	EMSA duplex forming oligo complementary to TTS_hit_2.	5'- TCGGGGGAGGGAGGAAAGAA -3'
TFO_*PART*_627_646_DNA	SPR oligo matching the triplex target site 627 – 646 bp in *PARTICLE.*	5’-TTTTTTGGTGGGTGGGGGCT-3’
TFO_*PART*_627-646_RNA	EMSA oligo matching the triplex target site 627 – 646 bp in *PARTICLE.*	5'- UUUUUUGGUGGGUGGGGGCU -3'
Scrambled_*PART.*	SPR oligo representing a scrambled version of the TFO from *PARTICLE* used for control purposes.	5’-GGTGTTGTGCGTTGTTGTGG-3’
TTS_*WWOX*_a_shuffled_1	SPR oligo with sequence shuffled for control purposes.	SH-5'-TTTTTTTTTTTTTTTTCTCTCCTCTTCCCCT -3'
TTS_*WWOX*_a_shuffled_2	SPR duplex forming oligo complementary to TTS_*WWOX*_a_shuffled_1 used for control purposes.	5'- AGGGGAAGAGGAGAGA -3'

Table S4 is relevant to Fig. 2. Sequence and description of oligos used for surface plasmon resonance or EMSA triplex detection between *PARTICLE* and *WWOX*.

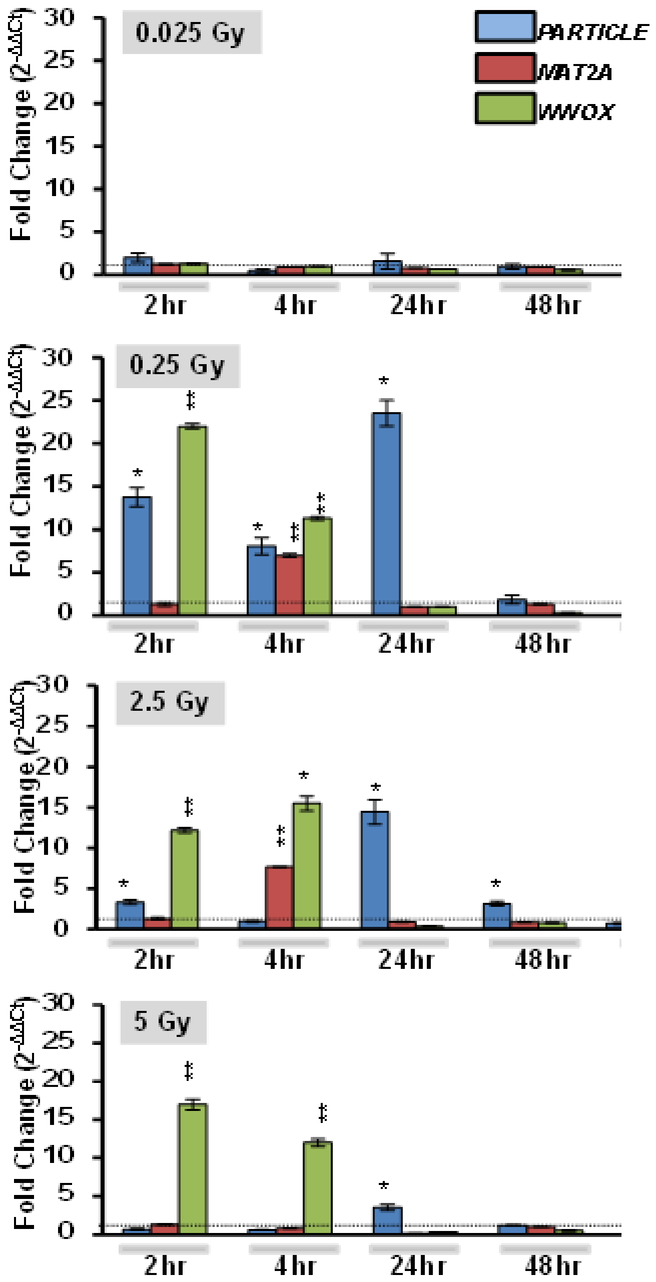

Fig. S1 is relevant to Fig. 3: Irradiation dose response of *PARTICLE*, *MAT2A* and *WWOX* in the breast cancer cell line MDA-MB-361. Relative gene expression was determined using the *TBP* endogenous control with comparison to sham irradiated controls normalized to a value of 1.

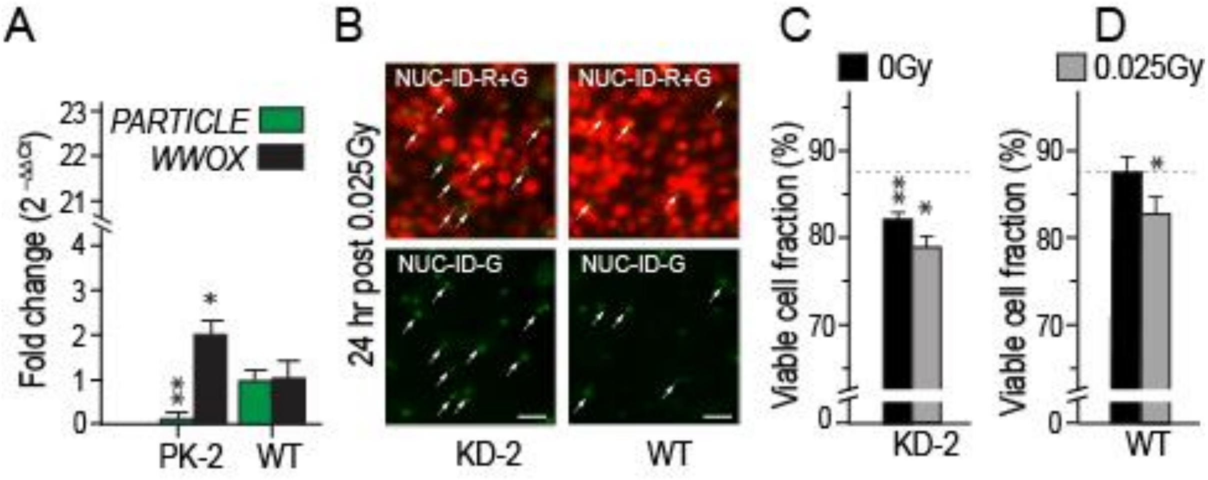

Fig. S2 relevant to Fig.3. PARTICLE knockdown by siRNA interference diminishes cellular viability. These experiments were conducted with a second siRNA (Thermo Fisher Scientific cat # n307634, Entrez gene id: 100630918) for confirmatory purposes. *(A)* Expression of *PARTICLE (green)* or *WWOX (black)* in U2OS cells (wild type; WT), or *PARTICLE* knockdown with the siRNA indicated above (PK-2). Values were normalized with the TATA-binding protein (TBP) encoding endogenous gene with relative expression comparison to relevant control. *(B)* Representative epifluorescence micrographs of MDA-MB-361 (WT) or *PARTICLE* knockdown (KD-2) 24 hr post 0.025 Gy identifying viable (red) or non-viable cells (green; lower) using NUCLEAR: ID (NUC-ID; Enzo). Merged images of red and green (R + G) stained nuclei (upper). Scale bar 25 µm. *(C - E)* Summary plots illustrating percentage viable cell fraction in KD-2 or WT 24 hr post sham irradiated or 0.025 Gy. Data are represented as mean ± SEM (n = 3) with significance represented by asterisks ($p < 0.05$) where appropriate.

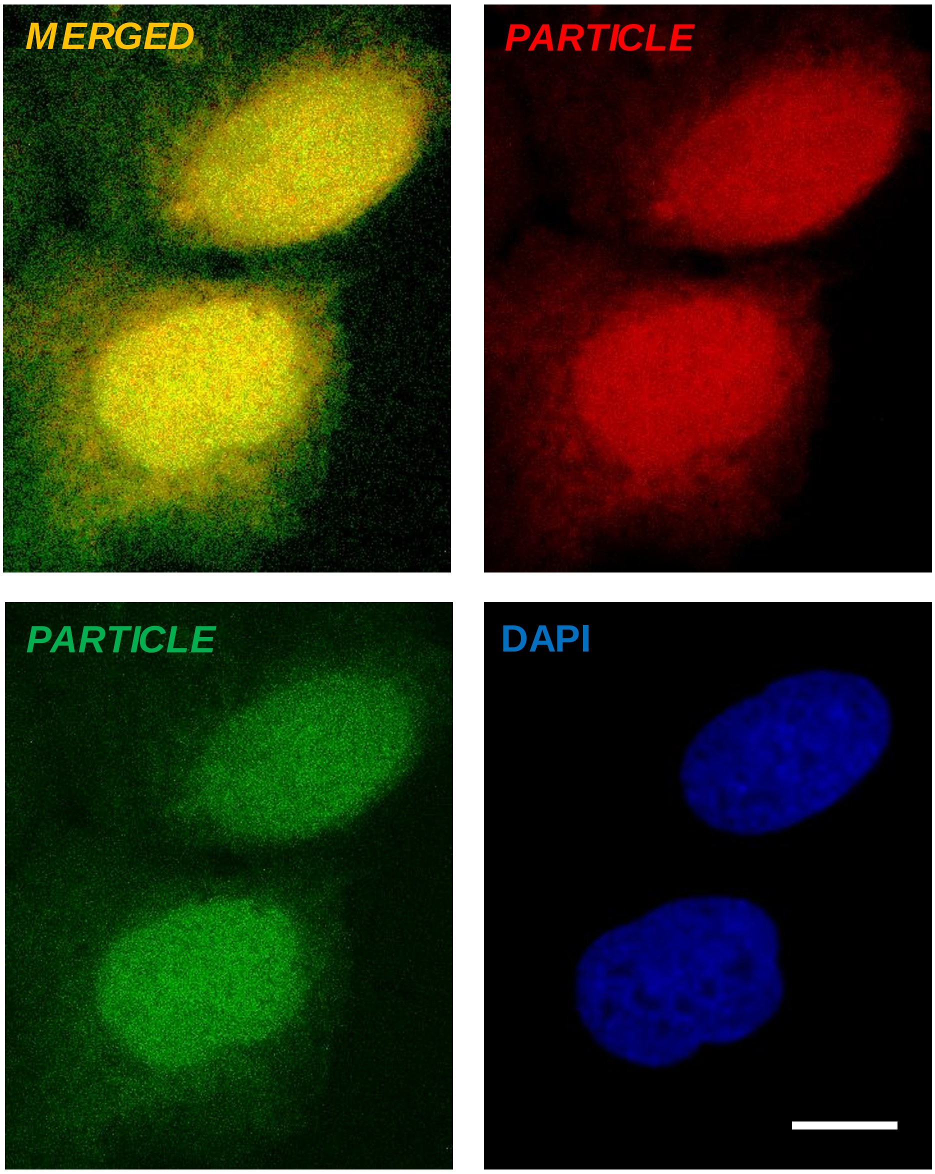

Fig. S3 relevant to Fig.4. Representative epifluorescence microscopic images of U2OS 24 hr post 0.25 Gy labelled with two independent sets of RNA *in situ* hybridisation probes (Stellaris two color system; carboxyfluorscein FAM (green) or Quasar 570 (red) specific for *PARTICLE*. Scale bar 20µm.

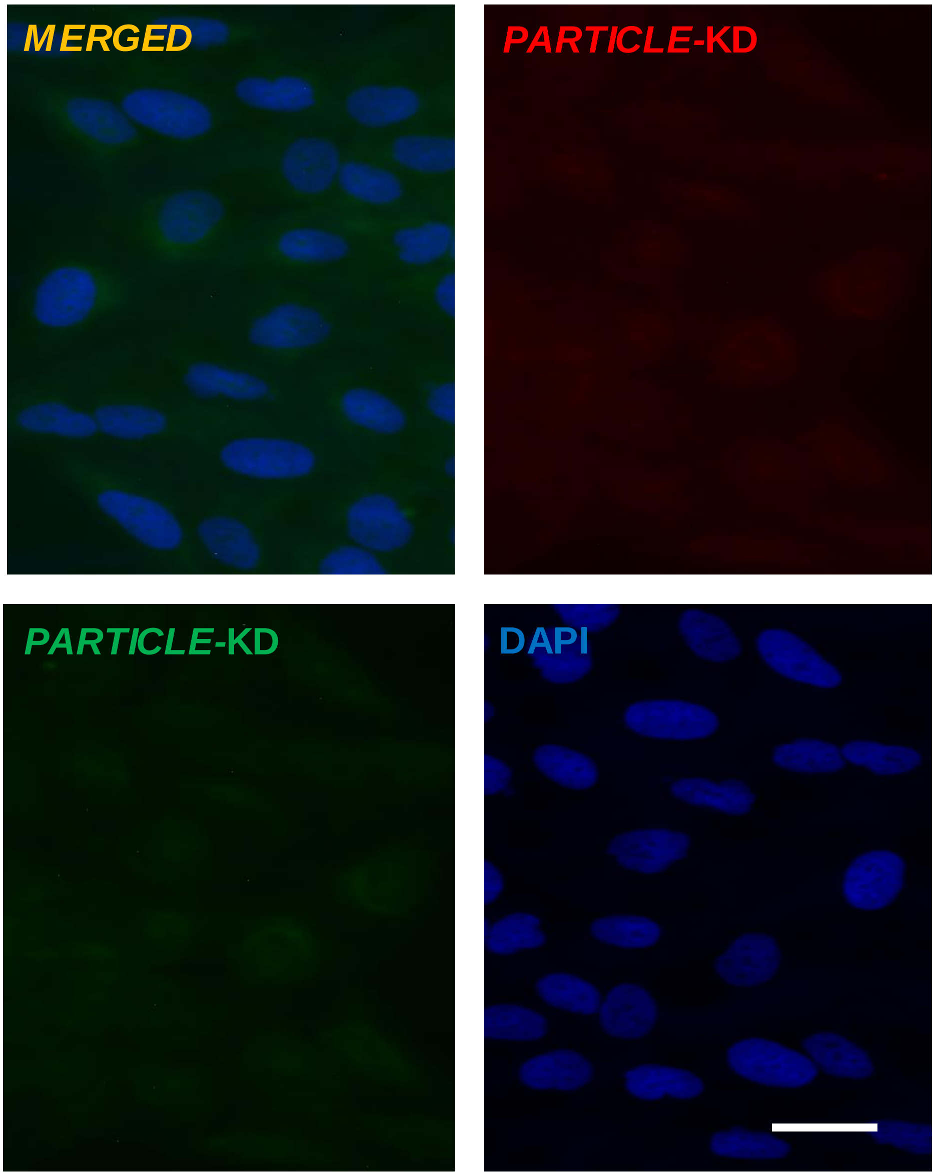

Fig. S4 relevant to Fig.4. Representative epifluorescence microscopic images of U2OS transfected for 72 hr with siRNA targeting *PARTICLE* (*PARTICLE* knockdown (KD) with Ambion id: n307629, part # 4390771) plus 24 hr post 0.25 Gy. These cells were then labelled with two independent sets of RNA *in situ* hybridisation probes (Stellaris two color system; carboxyfluorscein FAM (green) or Quasar 570 (red) specific for *PARTICLE*. Scale bar 5μm.

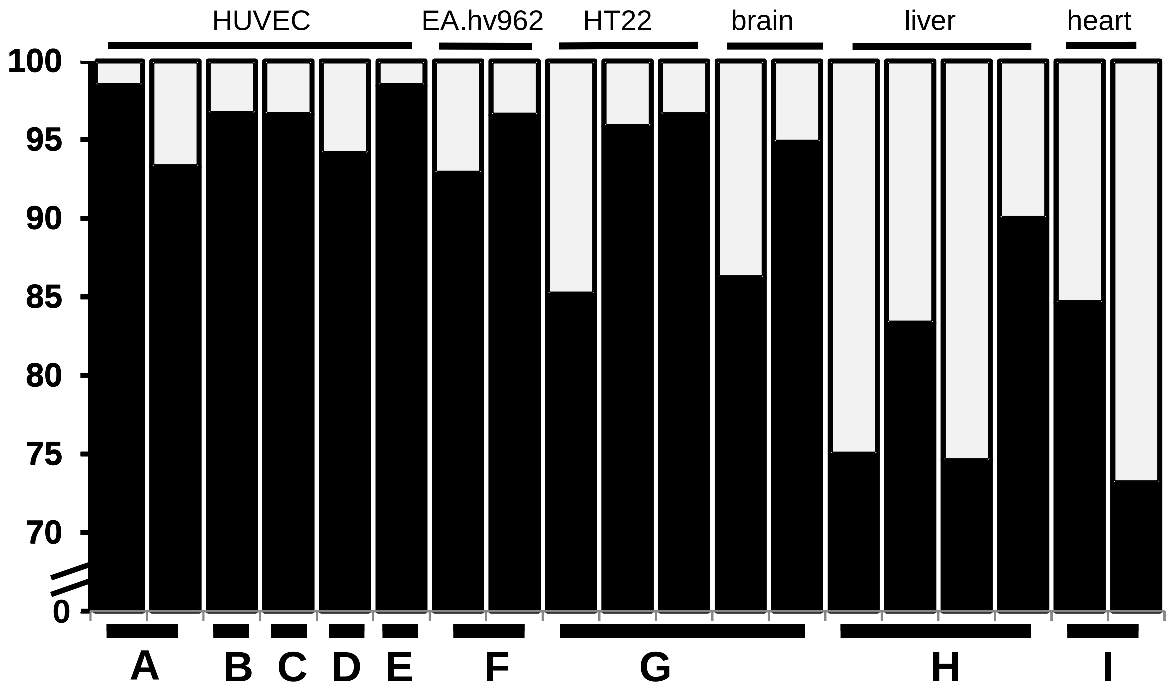

Published studies:

A: Yentrapali *et al.,* Plos One 2013 [2].

B: Yentrapali *et al.,* Proteomics 2013 [3].

C: Yentrapali *et al.,* Proteomics 2013 [3].

D: Yentrapali *et al.,* Proteomics 2015 [4].

E: Yentrapali *et al.,* Proteomics 2013 [3].

F: Sriharshan *et al.,* Journal of Proteomics 2012 [5].

G: Kempf *et al.,* Plos One 2014 [6].

H: Bakshi *et al.,* Journal of Proteomic Research 2013 [7].

I: Azimzadeh *et al.,* Proteomics 2011 [8].

Fig. S5 is relevant to Fig. 5. Metanalysis of previous published studies showing the percentage of oxidoreductase related proteins (white bars) deregulated in various cell lines/tissues (depicted at the top of the corresponding histogram) following irradiation exposure (0.05 Gy to 4 Gy within 24 hr).

References

1 Hanzelmann Sonja, K. C.-C., Kalwa Marie, Wagner Wolfgang, Costa Ivan G. Triplex Domain Finder: Detection of Triple Helix Binding Domains in Long Non-Coding RNAs. (2015).

2 Yentrapalli, R. *et al.* The PI3K/Akt/mTOR pathway is implicated in the premature senescence of primary human endothelial cells exposed to chronic radiation. *PLoS One* **8**, e70024, doi:10.1371/journal.pone.0070024 (2013).

3 Yentrapalli, R. *et al.* Quantitative proteomic analysis reveals induction of premature senescence in human umbilical vein endothelial cells exposed to chronic low-dose rate gamma radiation. *Proteomics* **13**, 1096-1107, doi:10.1002/pmic.201200463 (2013).

4 Yentrapalli, R. *et al.* Quantitative and integrated proteome and microRNA analysis of endothelial replicative senescence. *J Proteomics* **126**, 12-23, doi:10.1016/j.jprot.2015.05.023 (2015).

5 Sriharshan, A. *et al.* Proteomic analysis by SILAC and 2D-DIGE reveals radiation-induced endothelial response: four key pathways. *J Proteomics* **75**, 2319-2330, doi:10.1016/j.jprot.2012.02.009 (2012).

6 Kempf, S. J. *et al.* Ionising radiation immediately impairs synaptic plasticity-associated cytoskeletal signalling pathways in HT22 cells and in mouse brain: an in vitro/in vivo comparison study. *PLoS One* **9**, e110464, doi:10.1371/journal.pone.0110464 (2014).

7 Bakshi, M. V. *et al.* Long-term effects of acute low-dose ionizing radiation on the neonatal mouse heart: a proteomic study. *Radiat Environ Biophys* **52**, 451-461, doi:10.1007/s00411-013-0483-8 (2013).

8 Azimzadeh, O. *et al.* Rapid proteomic remodeling of cardiac tissue caused by total body ionizing radiation. *Proteomics* **11**, 3299-3311, doi:10.1002/pmic.201100178 (2011).
