# Supplementary material for: PARTICLE triplexes cluster in the tumor suppressor WWOX and may extend throughout the human genome
Source: Sci Rep. 2017 Aug 2;7:7163. doi: 10.1038/s41598-017-07295-5 (PMC5541130; doi:10.1038/s41598-017-07295-5)
Supplement: Supplementary file 2 — Supplementary Dataset 1 [file 41598_2017_7295_MOESM2_ESM.doc]

**Supplementary Table S1**

***PARTICLE* triplexes cluster in the tumor suppressor *WWOX* and may extend throughout the human genome.**

**Valerie Bríd O'Leary, Jan Smida, Fabian Andreas Buske, Laura Garcia Carrascosa, Omid Azimzadeh, Doris Maugg, Sarah Hain, Soile Tapio, Wolfgang Heidenreich, James Kerr, Matt Trau, Saak Victor Ovsepian, Michael John Atkinson.**

| **#** | **Human Genome Target Region** | **Associated Gene** | ***PARTICLE* triplex binding sites** |
| --- | --- | --- | --- |
| **1** | **chr 1: 14362-29806** | **DDX11L1** | **1** |
| **1** | **chr 1: 14362-29806** | **WASH7P** | **1** |
| **2** | **chr 1: 317719-453948** | **LOC100132287** | **1** |
| **2** | **chr 1: 317719-453948** | **LOC100132062** | **1** |
| **3** | **chr 1: 529832-532878** | **none** | **1** |
| **4** | **chr 1: 762987-794826** | **LINC00115** | **1** |
| **4** | **chr 1: 762987-794826** | **LOC643837** | **1** |
| **5** | **chr 1: 1567473-1570639** | **MMP23B** | **1** |
| **5** | **chr 1: 1567473-1570639** | **MMP23A** | **1** |
| **6** | **chr 1: 1634168-1677431** | **CDK11B** | **1** |
| **7** | **chr 1: 1716728-1822495** | **GNB1** | **1** |
| **8** | **chr 1: 1853395-1935276** | **KIAA1751** | **2** |
| **9** | **chr 1: 2115902-2145620** | **PRKCZ** | **1** |
| **10** | **chr 1: 2160133-2241558** | **SKI** | **1** |
| **11** | **chr 1: 2985731-3355185** | **PRDM16** | **3** |
| **11** | **chr 1: 2985731-3355185** | **MIR4251** | **3** |
| **12** | **chr 1: 3569083-3652765** | **TP73** | **2** |
| **13** | **chr 1: 5647427-5728355** | **MIR4417** | **1** |
| **14** | **chr 1: 6051525-6161253** | **KCNAB2** | **1** |
| **14** | **chr 1: 6051525-6161253** | **NPHP4** | **1** |
| **15** | **chr 1: 6161852-6240183** | **CHD5** | **2** |
| **16** | **chr 1: 6324328-6454451** | **ACOT7** | **1** |
| **17** | **chr 1: 6484847-6521430** | **MIR4252** | **2** |
| **17** | **chr 1: 6484847-6521430** | **ESPN** | **2** |
| **18** | **chr 1: 6521210-6580121** | **TNFRSF25** | **1** |
| **19** | **chr 1: 6845383-7829766** | **CAMTA1** | **10** |
| **20** | **chr 1: 8086797-8182762** | **ERRFI1** | **1** |
| **21** | **chr 1: 8412456-8877702** | **RERE** | **2** |
| **22** | **chr 1: 8921060-8939308** | **ENO1** | **1** |
| **23** | **chr 1: 9005925-9035151** | **CA6** | **1** |
| **24** | **chr 1: 9095165-9148537** | **SLC2A5** | **1** |
| **25** | **chr 1: 9789083-9884584** | **PIK3CD** | **3** |
| **26** | **chr 1: 10270862-10441661** | **KIF1B** | **1** |
| **27** | **chr 1: 10696660-10856707** | **CASZ1** | **2** |
| **28** | **chr 1: 11539222-11597641** | **PTCHD2** | **1** |
| **29** | **chr 1: 11821843-11849642** | **MTHFR** | **1** |
| **30** | **chr 1: 11845779-11866977** | **MTHFR** | **1** |
| **30** | **chr 1: 11845779-11866977** | **CLCN6** | **1** |
| **31** | **chr 1: 12290123-12572099** | **VPS13D** | **2** |
| **31** | **chr 1: 12290123-12572099** | **SNORA59A** | **2** |
| **32** | **chr 1: 13801444-13840543** | **LRRC38** | **1** |
| **33** | **chr 1: 13909959-13944452** | **PDPN** | **1** |
| **34** | **chr 1: 14925199-15444539** | **KAZN** | **4** |
| **35** | **chr 1: 15479027-15546976** | **TMEM51-AS1** | **2** |
| **35** | **chr 1: 15479027-15546976** | **TMEM51** | **2** |
| **36** | **chr 1: 15573767-15726779** | **FHAD1** | **1** |
| **37** | **chr 1: 16268363-16302627** | **ZBTB17** | **2** |
| **38** | **chr 1: 16713611-16763919** | **SZRD1** | **1** |
| **38** | **chr 1: 16713611-16763919** | **SPATA21** | **1** |
| **39** | **chr 1: 16793930-16825752** | **CROCCP3** | **1** |
| **40** | **chr 1: 16944750-16971178** | **CROCCP2** | **1** |
| **40** | **chr 1: 16944750-16971178** | **MST1P2** | **1** |
| **41** | **chr 1: 17013833-17046652** | **ESPNP** | **3** |
| **42** | **chr 1: 17066767-17299474** | **MST1L** | **3** |
| **42** | **chr 1: 17066767-17299474** | **MIR3675** | **3** |
| **43** | **chr 1: 17393255-17445948** | **PADI2** | **1** |
| **44** | **chr 1: 17698690-17728195** | **PADI6** | **1** |
| **45** | **chr 1: 17866329-18024369** | **ARHGEF10L** | **3** |
| **46** | **chr 1: 18434239-18704977** | **IGSF21** | **2** |
| **47** | **chr 1: 18957499-19075360** | **PAX7** | **1** |
| **48** | **chr 1: 19166092-19283180** | **TAS1R2** | **1** |
| **48** | **chr 1: 19166092-19283180** | **ALDH4A1** | **1** |
| **49** | **chr 1: 19665266-19812066** | **CAPZB** | **2** |
| **50** | **chr 1: 20008705-20126438** | **TMCO4** | **1** |
| **51** | **chr 1: 20487745-20503917** | **PLA2G2C** | **1** |
| **52** | **chr 1: 20510734-20512979** | **UBXN10** | **1** |
| **53** | **chr 1: 21132962-21503377** | **EIF4G3** | **1** |
| **54** | **chr 1: 21835857-21904905** | **ALPL** | **1** |
| **55** | **chr 1: 22004790-22110099** | **USP48** | **1** |
| **56** | **chr 1: 22443797-22470462** | **WNT4** | **1** |
| **57** | **chr 1: 22890056-22930087** | **EPHA8** | **5** |
| **58** | **chr 1: 23037331-23241818** | **EPHB2** | **1** |
| **58** | **chr 1: 23037331-23241818** | **MIR4684** | **1** |
| **59** | **chr 1: 23907984-23967058** | **MDS2** | **1** |
| **60** | **chr 1: 24882601-24935819** | **NCMAP** | **1** |
| **61** | **chr 1: 27860545-27930942** | **AHDC1** | **1** |
| **62** | **chr 1: 28099693-28150963** | **STX12** | **1** |
| **63** | **chr 1: 28696113-28826881** | **PHACTR4** | **2** |
| **64** | **chr 1: 29213602-29446553** | **EPB41** | **1** |
| **65** | **chr 1: 29445939-29450447** | **EPB41** | **1** |
| **66** | **chr 1: 31205315-31230667** | **LAPTM5** | **1** |
| **66** | **chr 1: 31205315-31230667** | **MIR4420** | **1** |
| **67** | **chr 1: 31404352-31538838** | **PUM1** | **1** |
| **67** | **chr 1: 31404352-31538838** | **SNORD103A** | **1** |
| **68** | **chr 1: 31769841-31837783** | **SNRNP40** | **1** |
| **68** | **chr 1: 31769841-31837783** | **ZCCHC17** | **1** |
| **69** | **chr 1: 32083286-32098119** | **HCRTR1** | **1** |
| **69** | **chr 1: 32083286-32098119** | **PEF1** | **1** |
| **70** | **chr 1: 32307619-32410457** | **PTP4A2** | **2** |
| **71** | **chr 1: 32517891-32539075** | **TMEM39B** | **1** |
| **71** | **chr 1: 32517891-32539075** | **KHDRBS1** | **1** |
| **72** | **chr 1: 32537631-32568467** | **TMEM39B** | **1** |
| **73** | **chr 1: 32817121-32829913** | **FAM229A** | **1** |
| **73** | **chr 1: 32817121-32829913** | **TSSK3** | **1** |
| **74** | **chr 1: 32930657-33071540** | **ZBTB8A** | **1** |
| **74** | **chr 1: 32930657-33071540** | **ZBTB8B** | **1** |
| **75** | **chr 1: 33240839-33283754** | **S100PBP** | **1** |
| **75** | **chr 1: 33240839-33283754** | **YARS** | **1** |
| **76** | **chr 1: 33402045-33430286** | **RNF19B** | **1** |
| **77** | **chr 1: 33789223-33896653** | **PHC2** | **1** |
| **78** | **chr 1: 33979608-34631443** | **CSMD2** | **3** |
| **78** | **chr 1: 33979608-34631443** | **HMGB4** | **3** |
| **79** | **chr 1: 34632483-34684732** | **C1ORF94** | **3** |
| **80** | **chr 1: 35178337-35325417** | **GJB5** | **3** |
| **80** | **chr 1: 35178337-35325417** | **GJB4** | **3** |
| **81** | **chr 1: 35525386-35581460** | **ZMYM1** | **1** |
| **82** | **chr 1: 36273772-36323491** | **AGO4** | **1** |
| **83** | **chr 1: 36396318-36538101** | **AGO3** | **1** |
| **84** | **chr 1: 36602172-36615098** | **TRAPPC3** | **1** |
| **85** | **chr 1: 37261127-37499730** | **GRIK3** | **2** |
| **86** | **chr 1: 38022519-38032458** | **DNALI1** | **1** |
| **86** | **chr 1: 38022519-38032458** | **GNL2** | **1** |
| **87** | **chr 1: 38076950-38100595** | **RSPO1** | **1** |
| **88** | **chr 1: 38179551-38230805** | **EPHA10** | **1** |
| **89** | **chr 1: 38674705-38680439** | **LOC339442** | **1** |
| **90** | **chr 1: 39546987-39952849** | **KIAA0754** | **2** |
| **90** | **chr 1: 39546987-39952849** | **MACF1** | **2** |
| **91** | **chr 1: 40144319-40157361** | **HPCAL4** | **2** |
| **92** | **chr 1: 40915773-40929390** | **ZFP69B** | **1** |
| **93** | **chr 1: 40942886-40962015** | **ZFP69** | **5** |
| **94** | **chr 1: 41086350-41131329** | **RIMS3** | **1** |
| **95** | **chr 1: 41492871-41707826** | **SLFNL1-AS1** | **1** |
| **96** | **chr 1: 41708044-41750533** | **SCMH1** | **1** |
| **97** | **chr 1: 41972035-42501596** | **HIVEP3** | **3** |
| **98** | **chr 1: 42895999-42921938** | **ZMYND12** | **1** |
| **98** | **chr 1: 42895999-42921938** | **PPCS** | **1** |
| **99** | **chr 1: 42921787-43120335** | **ZMYND12** | **2** |
| **99** | **chr 1: 42921787-43120335** | **PPCS** | **2** |
| **100** | **chr 1: 43124095-43142429** | **PPIH** | **1** |
| **101** | **chr 1: 44115828-44171186** | **KDM4A-AS1** | **1** |
| **101** | **chr 1: 44115828-44171186** | **KDM4A** | **1** |
| **102** | **chr 1: 44171494-44396831** | **KDM4A-AS1** | **3** |
| **102** | **chr 1: 44171494-44396831** | **ST3GAL3** | **3** |
| **103** | **chr 1: 44870865-45117396** | **RNF220** | **2** |
| **103** | **chr 1: 44870865-45117396** | **MIR5584** | **2** |
| **104** | **chr 1: 45205489-45233439** | **KIF2C** | **1** |
| **105** | **chr 1: 45316449-45452282** | **EIF2B3** | **2** |
| **106** | **chr 1: 45482070-45771881** | **ZSWIM5** | **6** |
| **106** | **chr 1: 45482070-45771881** | **LOC400752** | **6** |
| **107** | **chr 1: 46654353-46685977** | **POMGNT1** | **1** |
| **108** | **chr 1: 46669005-46686933** | **POMGNT1** | **1** |
| **108** | **chr 1: 46669005-46686933** | **LURAP1** | **1** |
| **109** | **chr 1: 46859936-46879520** | **FAAH** | **2** |
| **110** | **chr 1: 47004367-47035927** | **KNCN** | **1** |
| **110** | **chr 1: 47004367-47035927** | **MKNK1-AS1** | **1** |
| **111** | **chr 1: 47011315-47017199** | **KNCN** | **1** |
| **111** | **chr 1: 47011315-47017199** | **MKNK1-AS1** | **1** |
| **112** | **chr 1: 47223509-47285085** | **CYP4B1** | **2** |
| **113** | **chr 1: 47799468-47844511** | **CMPK1** | **1** |
| **114** | **chr 1: 48154134-48169055** | **none** | **1** |
| **115** | **chr 1: 48226199-48462567** | **TRABD2B** | **7** |
| **116** | **chr 1: 48761043-48937845** | **SPATA6** | **1** |
| **117** | **chr 1: 48998526-50489585** | **BEND5** | **6** |
| **117** | **chr 1: 48998526-50489585** | **AGBL4** | **6** |
| **118** | **chr 1: 49513987-49653257** | **AGBL4** | **2** |
| **119** | **chr 1: 52042850-52254889** | **OSBPL9** | **4** |
| **120** | **chr 1: 52254862-52344477** | **OSBPL9** | **2** |
| **121** | **chr 1: 53552854-53608289** | **SLC1A7** | **1** |
| **122** | **chr 1: 53971909-54199877** | **GLIS1** | **2** |
| **123** | **chr 1: 55007929-55104865** | **ACOT11** | **1** |
| **124** | **chr 1: 55074854-55094111** | **ACOT11** | **1** |
| **125** | **chr 1: 55271735-55307925** | **C1ORF177** | **1** |
| **126** | **chr 1: 57460450-59012474** | **DAB1** | **17** |
| **126** | **chr 1: 57460450-59012474** | **OMA1** | **17** |
| **127** | **chr 1: 59250822-59365384** | **LOC100131060** | **1** |
| **128** | **chr 1: 59522314-59553867** | **HSD52** | **1** |
| **129** | **chr 1: 59597607-59664293** | **HSD52** | **1** |
| **130** | **chr 1: 59762309-60233347** | **FGGY** | **3** |
| **131** | **chr 1: 60220418-60254854** | **FGGY** | **1** |
| **132** | **chr 1: 60280457-60342050** | **HOOK1** | **1** |
| **133** | **chr 1: 61330930-61928465** | **NFIA** | **2** |
| **134** | **chr 1: 63833260-63904233** | **ALG6** | **1** |
| **135** | **chr 1: 63906440-64059392** | **ITGB3BP** | **2** |
| **135** | **chr 1: 63906440-64059392** | **EFCAB7** | **2** |
| **136** | **chr 1: 64239692-64647181** | **ROR1** | **1** |
| **137** | **chr 1: 65298911-65432187** | **RAVER2** | **3** |
| **137** | **chr 1: 65298911-65432187** | **JAK1** | **3** |
| **138** | **chr 1: 66258196-66840259** | **PDE4B** | **3** |
| **139** | **chr 1: 67557847-67697536** | **C1ORF141** | **1** |
| **139** | **chr 1: 67557847-67697536** | **IL23R** | **1** |
| **140** | **chr 1: 67632082-67725662** | **IL23R** | **1** |
| **141** | **chr 1: 68297985-68668670** | **GNG12-AS1** | **2** |
| **141** | **chr 1: 68297985-68668670** | **GNG12** | **2** |
| **142** | **chr 1: 68564141-68698803** | **GNG12-AS1** | **1** |
| **143** | **chr 1: 69521580-69650686** | **none** | **1** |
| **144** | **chr 1: 70034080-70617628** | **PIN1P1** | **2** |
| **144** | **chr 1: 70034080-70617628** | **LRRC7** | **2** |
| **145** | **chr 1: 70610487-70671303** | **LRRC40** | **1** |
| **145** | **chr 1: 70610487-70671303** | **SRSF11** | **1** |
| **146** | **chr 1: 71172135-71252151** | **none** | **1** |
| **147** | **chr 1: 71861622-72748417** | **NEGR1** | **3** |
| **147** | **chr 1: 71861622-72748417** | **NEGR1-IT1** | **3** |
| **148** | **chr 1: 73771852-73820934** | **none** | **1** |
| **149** | **chr 1: 75667815-76076801** | **SLC44A5** | **4** |
| **150** | **chr 1: 76507375-76531913** | **ST6GALNAC3** | **1** |
| **151** | **chr 1: 76540403-77100286** | **ST6GALNAC3** | **1** |
| **152** | **chr 1: 77554674-77685115** | **PIGK** | **1** |
| **153** | **chr 1: 77747735-78025651** | **AK5** | **2** |
| **154** | **chr 1: 78028100-78149104** | **ZZZ3** | **3** |
| **155** | **chr 1: 81771844-82458120** | **LPHN2** | **4** |
| **156** | **chr 1: 84543744-84704181** | **PRKACB** | **1** |
| **157** | **chr 1: 84971973-85031877** | **CTBS** | **1** |
| **157** | **chr 1: 84971973-85031877** | **GNG5** | **1** |
| **158** | **chr 1: 85464829-85598821** | **WDR63** | **1** |
| **158** | **chr 1: 85464829-85598821** | **MCOLN3** | **1** |
| **159** | **chr 1: 85742397-85913807** | **LOC646626** | **1** |
| **159** | **chr 1: 85742397-85913807** | **BCL10** | **1** |
| **160** | **chr 1: 86194915-86622626** | **COL24A1** | **2** |
| **161** | **chr 1: 87036863-87158886** | **CLCA3P** | **1** |
| **161** | **chr 1: 87036863-87158886** | **CLCA4** | **1** |
| **162** | **chr 1: 89646830-89664615** | **GBP4** | **1** |
| **163** | **chr 1: 91177095-91182794** | **BARHL2** | **1** |
| **164** | **chr 1: 91380858-91487829** | **ZNF644** | **1** |
| **165** | **chr 1: 92145901-92371892** | **TGFBR3** | **2** |
| **166** | **chr 1: 92940318-92952433** | **GFI1** | **1** |
| **167** | **chr 1: 93727742-93811582** | **CCDC18** | **2** |
| **167** | **chr 1: 93727742-93811582** | **LOC100131564** | **2** |
| **168** | **chr 1: 94027346-94312706** | **BCAR3** | **3** |
| **169** | **chr 1: 94458392-94586688** | **ABCA4** | **2** |
| **170** | **chr 1: 94614543-94740624** | **ARHGAP29** | **3** |
| **171** | **chr 1: 95104016-95285837** | **SLC44A3** | **3** |
| **172** | **chr 1: 95582893-95712781** | **TMEM56** | **1** |
| **173** | **chr 1: 95628774-95699538** | **TMEM56** | **1** |
| **174** | **chr 1: 97187220-97289294** | **PTBP2** | **2** |
| **175** | **chr 1: 97543298-98386605** | **DPYD-AS1** | **2** |
| **175** | **chr 1: 97543298-98386605** | **DPYD** | **2** |
| **176** | **chr 1: 97561478-97788511** | **DPYD-AS1** | **2** |
| **176** | **chr 1: 97561478-97788511** | **DPYD** | **2** |
| **177** | **chr 1: 99127235-99226056** | **SNX7** | **1** |
| **178** | **chr 1: 99355805-99470588** | **LOC100129620** | **2** |
| **178** | **chr 1: 99355805-99470588** | **LPPR5** | **2** |
| **179** | **chr 1: 100435344-100492535** | **SLC35A3** | **1** |
| **180** | **chr 1: 100652474-100715390** | **DBT** | **1** |
| **181** | **chr 1: 102268129-102462586** | **OLFM3** | **2** |
| **181** | **chr 1: 102268129-102462586** | **DNAJA1P5** | **2** |
| **182** | **chr 1: 103342022-103574052** | **COL11A1** | **1** |
| **183** | **chr 1: 107682628-108026080** | **NTNG1** | **1** |
| **184** | **chr 1: 109102710-109187522** | **FAM102B** | **2** |
| **185** | **chr 1: 109358519-109506111** | **AKNAD1** | **2** |
| **186** | **chr 1: 109512835-109584850** | **WDR47** | **1** |
| **187** | **chr 1: 109792640-109818372** | **CELSR2** | **1** |
| **188** | **chr 1: 110026100-110035426** | **ATXN7L2** | **2** |
| **189** | **chr 1: 110574198-110617263** | **STRIP1** | **1** |
| **190** | **chr 1: 110602615-110613322** | **ALX3** | **1** |
| **191** | **chr 1: 110625309-110652341** | **UBL4B** | **1** |
| **191** | **chr 1: 110625309-110652341** | **ALX3** | **1** |
| **192** | **chr 1: 110753964-110825722** | **KCNC4** | **1** |
| **193** | **chr 1: 110828996-110881793** | **LOC440600** | **1** |
| **193** | **chr 1: 110828996-110881793** | **RBM15** | **1** |
| **194** | **chr 1: 111743392-111786062** | **DENND2D** | **2** |
| **195** | **chr 1: 111888909-111895635** | **PIFO** | **1** |
| **196** | **chr 1: 112223251-112298446** | **FAM212B** | **1** |
| **196** | **chr 1: 112223251-112298446** | **RAP1A** | **1** |
| **197** | **chr 1: 112313283-112531777** | **KCND3** | **2** |
| **198** | **chr 1: 112719855-112903150** | **CTTNBP2NL** | **1** |
| **199** | **chr 1: 112938802-113006078** | **MIR4256** | **1** |
| **199** | **chr 1: 112938802-113006078** | **CTTNBP2NL** | **1** |
| **200** | **chr 1: 113009162-113072787** | **WNT2B** | **1** |
| **201** | **chr 1: 113263040-113269857** | **FAM19A3** | **1** |
| **202** | **chr 1: 113499036-113542118** | **SLC16A1** | **1** |
| **203** | **chr 1: 113933370-114228545** | **MAGI3** | **1** |
| **204** | **chr 1: 115125468-115213043** | **DENND2C** | **2** |
| **205** | **chr 1: 115825654-115910693** | **NGF** | **1** |
| **206** | **chr 1: 115828538-115880857** | **NGF** | **1** |
| **207** | **chr 1: 116519118-116612675** | **SLC22A15** | **1** |
| **208** | **chr 1: 116966345-117021464** | **CD58** | **1** |
| **208** | **chr 1: 116966345-117021464** | **ATP1A1OS** | **1** |
| **209** | **chr 1: 117117030-117210375** | **IGSF3** | **1** |
| **210** | **chr 1: 117452678-117532980** | **PTGFRN** | **1** |
| **211** | **chr 1: 117910070-118071494** | **MAN1A2** | **1** |
| **212** | **chr 1: 118139453-118148392** | **FAM46C** | **1** |
| **213** | **chr 1: 119425668-119532179** | **TBX15** | **1** |
| **214** | **chr 1: 119573838-119683294** | **WARS2** | **1** |
| **215** | **chr 1: 119683018-119818596** | **WARS2** | **1** |
| **216** | **chr 1: 119802887-119869926** | **HAO2** | **1** |
| **217** | **chr 1: 143913614-144094424** | **FAM72D** | **3** |
| **218** | **chr 1: 144339737-144521058** | **LOC728875** | **2** |
| **218** | **chr 1: 144339737-144521058** | **LINC00623** | **2** |
| **219** | **chr 1: 144836156-145076186** | **PDE4DIP** | **1** |
| **220** | **chr 1: 145373053-145382434** | **HFE2** | **1** |
| **220** | **chr 1: 145373053-145382434** | **NBPF10** | **1** |
| **221** | **chr 1: 145524890-145543868** | **ITGA10** | **4** |
| **222** | **chr 1: 145924387-145942619** | **PDZK1P1** | **1** |
| **223** | **chr 1: 146032646-146253110** | **NBPF24** | **2** |
| **223** | **chr 1: 146032646-146253110** | **NBPF11** | **2** |
| **224** | **chr 1: 146310554-146349531** | **HYDIN2** | **1** |
| **225** | **chr 1: 146373545-146467744** | **LOC728989** | **1** |
| **225** | **chr 1: 146373545-146467744** | **HYDIN2** | **1** |
| **226** | **chr 1: 146490894-146596107** | **LOC728989** | **1** |
| **227** | **chr 1: 147466093-147484331** | **PDZK1P1** | **1** |
| **228** | **chr 1: 147574427-147634886** | **NBPF24** | **2** |
| **228** | **chr 1: 147574427-147634886** | **NBPF11** | **2** |
| **229** | **chr 1: 148555978-148596267** | **NBPF15** | **3** |
| **230** | **chr 1: 149909704-149982625** | **OTUD7B** | **1** |
| **230** | **chr 1: 149909704-149982625** | **MTMR11** | **1** |
| **231** | **chr 1: 150190716-150208504** | **ANP32E** | **1** |
| **232** | **chr 1: 150782180-150849244** | **ARNT** | **1** |
| **233** | **chr 1: 150933058-150947479** | **CERS2** | **1** |
| **233** | **chr 1: 150933058-150947479** | **SETDB1** | **1** |
| **234** | **chr 1: 150937735-150938734** | **CERS2** | **1** |
| **235** | **chr 1: 150980895-151008189** | **FAM63A** | **1** |
| **235** | **chr 1: 150980895-151008189** | **PRUNE** | **1** |
| **236** | **chr 1: 151023446-151042801** | **C1ORF56** | **1** |
| **236** | **chr 1: 151023446-151042801** | **CDC42SE1** | **1** |
| **237** | **chr 1: 151030233-151040970** | **MLLT11** | **1** |
| **237** | **chr 1: 151030233-151040970** | **CDC42SE1** | **1** |
| **238** | **chr 1: 151482985-151511168** | **CGN** | **1** |
| **239** | **chr 1: 151512780-151556059** | **MIR554** | **1** |
| **239** | **chr 1: 151512780-151556059** | **TUFT1** | **1** |
| **240** | **chr 1: 151673501-151680862** | **CELF3** | **2** |
| **241** | **chr 1: 151674879-151689290** | **CELF3** | **2** |
| **242** | **chr 1: 151772739-151804348** | **LINGO4** | **1** |
| **242** | **chr 1: 151772739-151804348** | **RORC** | **1** |
| **243** | **chr 1: 151814352-151822861** | **LOC100132111** | **1** |
| **243** | **chr 1: 151814352-151822861** | **THEM5** | **1** |
| **244** | **chr 1: 152647770-152659877** | **LCE2C** | **1** |
| **244** | **chr 1: 152647770-152659877** | **LCE2B** | **1** |
| **245** | **chr 1: 153777200-153895451** | **GATAD2B** | **1** |
| **246** | **chr 1: 153920144-153931101** | **CRTC2** | **1** |
| **246** | **chr 1: 153920144-153931101** | **DENND4B** | **1** |
| **247** | **chr 1: 153954126-153958834** | **RAB13** | **1** |
| **248** | **chr 1: 154192654-154243986** | **C1ORF43** | **1** |
| **248** | **chr 1: 154192654-154243986** | **UBAP2L** | **1** |
| **249** | **chr 1: 154374803-154379040** | **IL6R** | **1** |
| **250** | **chr 1: 154377668-154441926** | **IL6R** | **1** |
| **251** | **chr 1: 154975126-154990998** | **ZBTB7B** | **1** |
| **252** | **chr 1: 155006299-155035252** | **DCST2** | **1** |
| **252** | **chr 1: 155006299-155035252** | **DCST1** | **1** |
| **253** | **chr 1: 155247373-155259639** | **HCN3** | **1** |
| **254** | **chr 1: 156182772-156213123** | **PMF1-BGLAP** | **2** |
| **254** | **chr 1: 156182772-156213123** | **PMF1** | **2** |
| **255** | **chr 1: 156219014-156252620** | **SMG5** | **1** |
| **255** | **chr 1: 156219014-156252620** | **TMEM79** | **1** |
| **256** | **chr 1: 156720401-156770607** | **HDGF** | **1** |
| **257** | **chr 1: 156785431-156851642** | **SH2D2A** | **2** |
| **258** | **chr 1: 156809854-156828810** | **NTRK1** | **1** |
| **258** | **chr 1: 156809854-156828810** | **INSRR** | **1** |
| **259** | **chr 1: 156904631-157015162** | **ARHGEF11** | **2** |
| **259** | **chr 1: 156904631-157015162** | **MIR765** | **2** |
| **260** | **chr 1: 157090982-157108266** | **CYCSP52** | **1** |
| **260** | **chr 1: 157090982-157108266** | **ETV3** | **1** |
| **261** | **chr 1: 157543538-157567870** | **FCRL4** | **1** |
| **262** | **chr 1: 157963062-158070052** | **KIRREL** | **3** |
| **263** | **chr 1: 160121359-160156767** | **ATP1A4** | **1** |
| **264** | **chr 1: 160185504-160256138** | **DCAF8** | **1** |
| **265** | **chr 1: 160313061-160328742** | **COPA** | **1** |
| **266** | **chr 1: 161154097-161168846** | **ADAMTS4** | **3** |
| **266** | **chr 1: 161154097-161168846** | **NDUFS2** | **3** |
| **267** | **chr 1: 161166893-161184185** | **ADAMTS4** | **1** |
| **267** | **chr 1: 161166893-161184185** | **NDUFS2** | **1** |
| **268** | **chr 1: 161334520-161337664** | **C1ORF192** | **1** |
| **268** | **chr 1: 161334520-161337664** | **SDHC** | **1** |
| **269** | **chr 1: 162601162-162757190** | **DDR2** | **1** |
| **270** | **chr 1: 162794247-162838605** | **C1ORF110** | **1** |
| **271** | **chr 1: 162794584-162819257** | **C1ORF110** | **1** |
| **271** | **chr 1: 162794584-162819257** | **HSD17B7** | **1** |
| **272** | **chr 1: 163080910-163291684** | **RGS5** | **1** |
| **273** | **chr 1: 163236365-163325554** | **RGS5** | **2** |
| **274** | **chr 1: 164524820-164868533** | **LOC100505795** | **1** |
| **274** | **chr 1: 164524820-164868533** | **PBX1** | **1** |
| **275** | **chr 1: 165446077-165551392** | **LRRC52** | **1** |
| **275** | **chr 1: 165446077-165551392** | **LOC400794** | **1** |
| **276** | **chr 1: 165567699-165592568** | **LRRC52** | **1** |
| **276** | **chr 1: 165567699-165592568** | **MGST3** | **1** |
| **277** | **chr 1: 165631452-165668100** | **LOC440700** | **2** |
| **277** | **chr 1: 165631452-165668100** | **ALDH9A1** | **2** |
| **278** | **chr 1: 166356963-166421869** | **none** | **2** |
| **279** | **chr 1: 167690428-167761156** | **MPZL1** | **1** |
| **280** | **chr 1: 169101768-169337205** | **NME7** | **1** |
| **280** | **chr 1: 169101768-169337205** | **ATP1B1** | **1** |
| **281** | **chr 1: 169631244-169823221** | **SELL** | **2** |
| **282** | **chr 1: 169890466-170054349** | **KIFAP3** | **1** |
| **283** | **chr 1: 171810620-172387606** | **DNM3OS** | **2** |
| **283** | **chr 1: 171810620-172387606** | **DNM3** | **2** |
| **284** | **chr 1: 172501488-172580971** | **SUCO** | **1** |
| **285** | **chr 1: 172745044-173033155** | **TNFSF18** | **1** |
| **286** | **chr 1: 173578699-173639001** | **ANKRD45** | **1** |
| **286** | **chr 1: 173578699-173639001** | **LOC730159** | **1** |
| **287** | **chr 1: 175036993-175117202** | **TNN** | **4** |
| **288** | **chr 1: 175284329-175712906** | **TNR** | **7** |
| **289** | **chr 1: 175913966-176176629** | **SCARNA3** | **1** |
| **289** | **chr 1: 175913966-176176629** | **RFWD2** | **1** |
| **290** | **chr 1: 176432306-176814735** | **PAPPA2** | **2** |
| **291** | **chr 1: 176826437-177134109** | **ASTN1** | **1** |
| **292** | **chr 1: 177140632-177251558** | **FAM5B** | **1** |
| **293** | **chr 1: 177893090-178007142** | **SEC16B** | **2** |
| **293** | **chr 1: 177893090-178007142** | **LOC730102** | **2** |
| **294** | **chr 1: 178482211-178518024** | **TEX35** | **1** |
| **295** | **chr 1: 179712297-179785333** | **FAM163A** | **1** |
| **296** | **chr 1: 180601139-180859387** | **XPR1** | **1** |
| **297** | **chr 1: 180882289-180920750** | **KIAA1614** | **1** |
| **298** | **chr 1: 181382237-181777219** | **CACNA1E** | **4** |
| **299** | **chr 1: 182367251-182369751** | **TEDDM1** | **1** |
| **300** | **chr 1: 183217371-183387737** | **NMNAT2** | **1** |
| **301** | **chr 1: 183605219-183897665** | **RGL1** | **4** |
| **301** | **chr 1: 183605219-183897665** | **ARPC5** | **4** |
| **302** | **chr 1: 184356191-184598154** | **C1ORF21** | **2** |
| **303** | **chr 1: 184759857-184943682** | **FAM129A** | **5** |
| **304** | **chr 1: 185014495-185071740** | **RNF2** | **2** |
| **305** | **chr 1: 185703682-186160085** | **HMCN1** | **2** |
| **305** | **chr 1: 185703682-186160085** | **MIR548F1** | **2** |
| **306** | **chr 1: 190066791-190446759** | **FAM5C** | **1** |
| **307** | **chr 1: 190594019-190770788** | **LOC440704** | **1** |
| **308** | **chr 1: 191844624-191980390** | **none** | **1** |
| **309** | **chr 1: 192605274-192629390** | **RGS13** | **2** |
| **310** | **chr 1: 196194908-196578355** | **KCNT2** | **1** |
| **310** | **chr 1: 196194908-196578355** | **MIR4735** | **1** |
| **311** | **chr 1: 197053257-197115824** | **ASPM** | **1** |
| **312** | **chr 1: 197871776-197876497** | **C1ORF53** | **1** |
| **313** | **chr 1: 198607800-198726545** | **PTPRC** | **1** |
| **314** | **chr 1: 200374067-200379184** | **ZNF281** | **1** |
| **315** | **chr 1: 201159952-201198080** | **IGFN1** | **1** |
| **316** | **chr 1: 201476368-201502736** | **CSRP1** | **1** |
| **317** | **chr 1: 201592012-201796102** | **NAV1** | **4** |
| **317** | **chr 1: 201592012-201796102** | **MIR5191** | **4** |
| **318** | **chr 1: 202317826-202561834** | **PPP1R12B** | **2** |
| **319** | **chr 1: 203059781-203136533** | **ADORA1** | **1** |
| **320** | **chr 1: 203113091-203122028** | **ADORA1** | **1** |
| **321** | **chr 1: 203181954-203242769** | **CHIT1** | **1** |
| **322** | **chr 1: 203267885-203274437** | **LOC730227** | **1** |
| **322** | **chr 1: 203267885-203274437** | **BTG2** | **1** |
| **323** | **chr 1: 204042242-204096863** | **SOX13** | **1** |
| **324** | **chr 1: 204346977-204404974** | **PIK3C2B** | **1** |
| **324** | **chr 1: 204346977-204404974** | **PPP1R15B** | **1** |
| **325** | **chr 1: 204485510-204542871** | **MDM4** | **2** |
| **326** | **chr 1: 204586297-204654861** | **LRRN2** | **1** |
| **327** | **chr 1: 204797778-204991950** | **NFASC** | **1** |
| **328** | **chr 1: 205055269-205091143** | **RBBP5** | **1** |
| **329** | **chr 1: 205111631-205180727** | **DSTYK** | **1** |
| **330** | **chr 1: 205305219-205326218** | **KLHDC8A** | **1** |
| **331** | **chr 1: 205350505-205425082** | **LEMD1-AS1** | **3** |
| **331** | **chr 1: 205350505-205425082** | **LEMD1** | **3** |
| **332** | **chr 1: 206516199-206637783** | **SRGAP2B** | **1** |
| **332** | **chr 1: 206516199-206637783** | **SRGAP2C** | **1** |
| **333** | **chr 1: 206858288-206907628** | **MAPKAPK2** | **1** |
| **334** | **chr 1: 206972214-207016324** | **IL19** | **1** |
| **335** | **chr 1: 207669491-207813992** | **CR1** | **4** |
| **336** | **chr 1: 207974862-208042495** | **MIR29C** | **1** |
| **336** | **chr 1: 207974862-208042495** | **MIR29B2** | **1** |
| **337** | **chr 1: 208195586-208417665** | **PLXNA2** | **2** |
| **338** | **chr 1: 209701799-209741018** | **CAMK1G** | **1** |
| **339** | **chr 1: 210501595-210849638** | **HHAT** | **1** |
| **340** | **chr 1: 210856554-211307457** | **KCNH1** | **2** |
| **341** | **chr 1: 211499956-211548288** | **TRAF5** | **1** |
| **342** | **chr 1: 211649863-211666259** | **RD3** | **1** |
| **343** | **chr 1: 212537272-212588243** | **TMEM206** | **1** |
| **344** | **chr 1: 213025449-213031430** | **FLVCR1** | **1** |
| **344** | **chr 1: 213025449-213031430** | **FLVCR1-AS1** | **1** |
| **345** | **chr 1: 214517514-214725792** | **PTPN14** | **1** |
| **346** | **chr 1: 215179117-215410436** | **KCNK2** | **1** |
| **347** | **chr 1: 216676587-217311097** | **ESRRG** | **1** |
| **348** | **chr 1: 217600333-217804424** | **SPATA17** | **1** |
| **348** | **chr 1: 217600333-217804424** | **GPATCH2** | **1** |
| **349** | **chr 1: 217804665-218045038** | **SPATA17** | **1** |
| **349** | **chr 1: 217804665-218045038** | **GPATCH2** | **1** |
| **350** | **chr 1: 219259943-219347303** | **LYPLAL1** | **1** |
| **350** | **chr 1: 219259943-219347303** | **LOC643723** | **1** |
| **351** | **chr 1: 219347185-219386207** | **LYPLAL1** | **1** |
| **351** | **chr 1: 219347185-219386207** | **LOC643723** | **1** |
| **352** | **chr 1: 219858768-220131989** | **RNU5F-1** | **2** |
| **352** | **chr 1: 219858768-220131989** | **SLC30A10** | **2** |
| **353** | **chr 1: 222262510-222560776** | **none** | **1** |
| **354** | **chr 1: 222626081-222628047** | **none** | **1** |
| **355** | **chr 1: 222910548-222924147** | **FAM177B** | **2** |
| **356** | **chr 1: 223394160-223537544** | **SUSD4** | **1** |
| **357** | **chr 1: 223711348-223853436** | **CAPN8** | **1** |
| **358** | **chr 1: 223889294-223963720** | **CAPN2** | **2** |
| **359** | **chr 1: 224622361-224928251** | **WDR26** | **2** |
| **360** | **chr 1: 225634944-225653045** | **ENAH** | **1** |
| **360** | **chr 1: 225634944-225653045** | **LBR** | **1** |
| **361** | **chr 1: 225674536-225840844** | **ENAH** | **1** |
| **362** | **chr 1: 226819390-226927024** | **ITPKB** | **1** |
| **363** | **chr 1: 227085236-227175246** | **ADCK3** | **1** |
| **364** | **chr 1: 227177565-227506175** | **CDC42BPA** | **3** |
| **365** | **chr 1: 228395830-228566577** | **C1ORF145** | **1** |
| **366** | **chr 1: 228595640-228604562** | **TRIM17** | **1** |
| **367** | **chr 1: 229394027-229407346** | **RAB4A** | **1** |
| **368** | **chr 1: 230193535-230417870** | **GALNT2** | **2** |
| **369** | **chr 1: 230457391-230561475** | **PGBD5** | **3** |
| **370** | **chr 1: 230838268-230931238** | **AGT** | **1** |
| **370** | **chr 1: 230838268-230931238** | **CAPN9** | **1** |
| **371** | **chr 1: 230883129-230937749** | **CAPN9** | **1** |
| **372** | **chr 1: 231297857-231357302** | **LOC149373** | **2** |
| **372** | **chr 1: 231297857-231357302** | **TRIM67** | **2** |
| **373** | **chr 1: 231664398-232177018** | **TSNAX-DISC1** | **4** |
| **373** | **chr 1: 231664398-232177018** | **TSNAX** | **4** |
| **374** | **chr 1: 232295836-232321389** | **none** | **1** |
| **375** | **chr 1: 232310677-232315859** | **none** | **1** |
| **376** | **chr 1: 233119180-233431473** | **PCNXL2** | **1** |
| **377** | **chr 1: 234040678-234460262** | **MIR4671** | **1** |
| **377** | **chr 1: 234040678-234460262** | **SLC35F3** | **1** |
| **378** | **chr 1: 235294497-235491534** | **RBM34** | **3** |
| **379** | **chr 1: 235530674-235612283** | **TBCE** | **1** |
| **379** | **chr 1: 235530674-235612283** | **B3GALNT2** | **1** |
| **380** | **chr 1: 235613237-235667781** | **B3GALNT2** | **1** |
| **381** | **chr 1: 237205504-237997288** | **RYR2** | **5** |
| **382** | **chr 1: 239549864-240078750** | **CHRM3** | **4** |
| **383** | **chr 1: 240177647-240638489** | **FMN2** | **3** |
| **384** | **chr 1: 240652872-240775449** | **GREM2** | **1** |
| **385** | **chr 1: 240931553-241520530** | **MIR3123** | **1** |
| **385** | **chr 1: 240931553-241520530** | **RGS7** | **1** |
| **386** | **chr 1: 241753403-241840678** | **KMO** | **3** |
| **386** | **chr 1: 241753403-241840678** | **OPN3** | **3** |
| **387** | **chr 1: 241815579-241965435** | **WDR64** | **3** |
| **388** | **chr 1: 242246287-242687998** | **PLD5** | **2** |
| **389** | **chr 1: 243419319-243663394** | **SDCCAG8** | **1** |
| **389** | **chr 1: 243419319-243663394** | **CEP170** | **1** |
| **390** | **chr 1: 243651534-244014381** | **SDCCAG8** | **3** |
| **391** | **chr 1: 245318286-245872733** | **KIF26B** | **6** |
| **392** | **chr 1: 245912641-246670614** | **SMYD3** | **2** |
| **393** | **chr 1: 246887348-246931439** | **SCCPDH** | **1** |
| **394** | **chr 1: 247002399-247095280** | **AHCTF1** | **1** |
| **395** | **chr 1: 248020500-248060449** | **OR2W3** | **1** |
| **395** | **chr 1: 248020500-248060449** | **TRIM58** | **1** |
| **396** | **chr 1: 249104647-249120832** | **MIR3124** | **1** |
| **396** | **chr 1: 249104647-249120832** | **SH3BP5L** | **1** |
| **397** | **chr 1: 249200394-249214145** | **PGBD2** | **1** |
| **398** | **chr 10: 320129-735683** | **MIR5699** | **4** |
| **398** | **chr 10: 320129-735683** | **DIP2C** | **4** |
| **399** | **chr 10: 1228072-1779670** | **ADARB2-AS1** | **6** |
| **399** | **chr 10: 1228072-1779670** | **ADARB2** | **6** |
| **400** | **chr 10: 3793258-3805418** | **KLF6** | **1** |
| **401** | **chr 10: 5454513-5500426** | **NET1** | **1** |
| **402** | **chr 10: 5931534-5979556** | **ANKRD16** | **2** |
| **403** | **chr 10: 7200585-7453450** | **SFMBT2** | **4** |
| **404** | **chr 10: 7487519-7513904** | **SFMBT2** | **1** |
| **405** | **chr 10: 7860466-8058590** | **TAF3** | **2** |
| **406** | **chr 10: 8095566-8117161** | **GATA3-AS1** | **1** |
| **406** | **chr 10: 8095566-8117161** | **GATA3** | **1** |
| **407** | **chr 10: 11495944-11653753** | **USP6NL** | **1** |
| **408** | **chr 10: 11962020-12085169** | **UPF2** | **1** |
| **409** | **chr 10: 12391480-12877545** | **CAMK1D** | **7** |
| **410** | **chr 10: 12938626-13141652** | **CCDC3** | **1** |
| **410** | **chr 10: 12938626-13141652** | **OPTN** | **1** |
| **411** | **chr 10: 13263766-13276334** | **UCMA** | **1** |
| **412** | **chr 10: 13480483-13570974** | **BEND7** | **1** |
| **413** | **chr 10: 13685705-14504141** | **FRMD4A** | **8** |
| **413** | **chr 10: 13685705-14504141** | **MIR1265** | **8** |
| **414** | **chr 10: 14560555-14816896** | **FAM107B** | **2** |
| **415** | **chr 10: 15139178-15181817** | **RPP38** | **2** |
| **415** | **chr 10: 15139178-15181817** | **C10ORF111** | **2** |
| **416** | **chr 10: 15144582-15210692** | **RPP38** | **2** |
| **416** | **chr 10: 15144582-15210692** | **NMT2** | **2** |
| **417** | **chr 10: 15555947-15762124** | **ITGA8** | **1** |
| **418** | **chr 10: 16865962-17171830** | **CUBN** | **3** |
| **419** | **chr 10: 17360381-17496329** | **ST8SIA6** | **2** |
| **419** | **chr 10: 17360381-17496329** | **ST8SIA6-AS1** | **2** |
| **420** | **chr 10: 17851361-17953178** | **MRC1** | **1** |
| **420** | **chr 10: 17851361-17953178** | **MIR511-1** | **1** |
| **421** | **chr 10: 18098351-18200091** | **MRC1** | **1** |
| **421** | **chr 10: 18098351-18200091** | **MIR511-1** | **1** |
| **422** | **chr 10: 18429605-18830798** | **CACNB2** | **2** |
| **423** | **chr 10: 19492778-20079330** | **PLXDC2** | **5** |
| **424** | **chr 10: 20105167-20578785** | **PLXDC2** | **4** |
| **425** | **chr 10: 21802406-21814611** | **SKIDA1** | **1** |
| **426** | **chr 10: 22634398-22743153** | **SPAG6** | **1** |
| **426** | **chr 10: 22634398-22743153** | **LOC100499489** | **1** |
| **427** | **chr 10: 23556123-23633774** | **C10ORF67** | **7** |
| **428** | **chr 10: 23983674-24836772** | **KIAA1217** | **2** |
| **429** | **chr 10: 25401986-25450165** | **GPR158-AS1** | **1** |
| **430** | **chr 10: 25463990-25891155** | **GPR158-AS1** | **2** |
| **430** | **chr 10: 25463990-25891155** | **GPR158** | **2** |
| **431** | **chr 10: 26505235-26593487** | **GAD2** | **2** |
| **432** | **chr 10: 27280842-27389421** | **ANKRD26** | **1** |
| **433** | **chr 10: 27399382-27444195** | **MASTL** | **1** |
| **433** | **chr 10: 27399382-27444195** | **YME1L1** | **1** |
| **434** | **chr 10: 28033714-28056723** | **MKX** | **1** |
| **435** | **chr 10: 28339921-28623415** | **MPP7** | **3** |
| **436** | **chr 10: 28811580-28821672** | **WAC** | **1** |
| **436** | **chr 10: 28811580-28821672** | **WAC-AS1** | **1** |
| **437** | **chr 10: 28821421-28912041** | **WAC** | **1** |
| **437** | **chr 10: 28821421-28912041** | **WAC-AS1** | **1** |
| **438** | **chr 10: 29746266-30025710** | **SVIL** | **5** |
| **439** | **chr 10: 30301728-30404423** | **KIAA1462** | **1** |
| **440** | **chr 10: 30960494-31006195** | **LYZL2** | **1** |
| **441** | **chr 10: 32556678-32667726** | **EPC1** | **1** |
| **442** | **chr 10: 33189246-33294720** | **ITGB1** | **1** |
| **443** | **chr 10: 33247772-33371030** | **ITGB1** | **1** |
| **444** | **chr 10: 33466419-33625190** | **NRP1** | **1** |
| **445** | **chr 10: 34398487-35104253** | **PARD3** | **4** |
| **446** | **chr 10: 35535952-35860852** | **CCNY** | **1** |
| **447** | **chr 10: 38064298-38073059** | **ZNF248** | **1** |
| **448** | **chr 10: 43633933-43680756** | **CSGALNACT2** | **1** |
| **449** | **chr 10: 44793037-44881941** | **CXCL12** | **1** |
| **450** | **chr 10: 45306471-45455137** | **TMEM72** | **4** |
| **450** | **chr 10: 45306471-45455137** | **TMEM72-AS1** | **4** |
| **451** | **chr 10: 45869660-45941561** | **ALOX5** | **3** |
| **452** | **chr 10: 46657134-46681603** | **PTPN20B** | **1** |
| **453** | **chr 10: 46951471-46966835** | **SYT15** | **2** |
| **454** | **chr 10: 46955443-46971400** | **SYT15** | **2** |
| **455** | **chr 10: 48355023-48373866** | **ZNF488** | **2** |
| **456** | **chr 10: 48844035-48868504** | **FRMPD2P1** | **1** |
| **457** | **chr 10: 49514697-49647403** | **MAPK8** | **2** |
| **458** | **chr 10: 49892920-50191001** | **WDFY4** | **1** |
| **458** | **chr 10: 49892920-50191001** | **LRRC18** | **1** |
| **459** | **chr 10: 50362772-50396630** | **C10ORF128** | **2** |
| **460** | **chr 10: 52750944-54058110** | **PRKG1** | **4** |
| **461** | **chr 10: 55562530-57387702** | **PCDH15** | **6** |
| **462** | **chr 10: 56245989-56415811** | **PCDH15** | **1** |
| **463** | **chr 10: 61548520-61666414** | **CCDC6** | **1** |
| **464** | **chr 10: 61786055-62493248** | **ANK3** | **6** |
| **465** | **chr 10: 63661058-63856703** | **MIR548AV** | **1** |
| **465** | **chr 10: 63661058-63856703** | **ARID5B** | **1** |
| **466** | **chr 10: 64133950-64431771** | **ZNF365** | **2** |
| **467** | **chr 10: 65424423-65750328** | **REEP3** | **1** |
| **468** | **chr 10: 67672275-69455927** | **CTNNA3** | **7** |
| **469** | **chr 10: 69865911-69971774** | **MYPN** | **1** |
| **470** | **chr 10: 70042416-70092806** | **PBLD** | **1** |
| **471** | **chr 10: 70173820-70231879** | **DNA2** | **1** |
| **472** | **chr 10: 70320412-70454239** | **TET1** | **2** |
| **473** | **chr 10: 72007084-72043432** | **NPFFR1** | **2** |
| **474** | **chr 10: 73156690-73575702** | **CDH23** | **3** |
| **475** | **chr 10: 73507315-73533255** | **CDH23** | **1** |
| **476** | **chr 10: 74092587-74114988** | **DNAJB12** | **1** |
| **477** | **chr 10: 74766974-74856732** | **P4HA1** | **1** |
| **478** | **chr 10: 75434032-75490272** | **AGAP5** | **1** |
| **478** | **chr 10: 75434032-75490272** | **BMS1P4** | **1** |
| **479** | **chr 10: 75757871-75879918** | **VCL** | **1** |
| **480** | **chr 10: 75910959-76469061** | **AP3M1** | **1** |
| **480** | **chr 10: 75910959-76469061** | **ADK** | **1** |
| **481** | **chr 10: 76585339-76792380** | **KAT6B** | **1** |
| **482** | **chr 10: 76797593-76818272** | **DUPD1** | **1** |
| **483** | **chr 10: 77160758-77168738** | **ZNF503** | **1** |
| **483** | **chr 10: 77160758-77168738** | **ZNF503-AS2** | **1** |
| **484** | **chr 10: 77360997-78319925** | **C10ORF11** | **6** |
| **485** | **chr 10: 78629358-79398353** | **KCNMA1** | **6** |
| **486** | **chr 10: 78647801-78740382** | **KCNMA1** | **1** |
| **487** | **chr 10: 79550548-79686378** | **DLG5-AS1** | **1** |
| **487** | **chr 10: 79550548-79686378** | **DLG5** | **1** |
| **488** | **chr 10: 80008504-80434724** | **LINC00856** | **3** |
| **488** | **chr 10: 80008504-80434724** | **LINC00595** | **3** |
| **489** | **chr 10: 81142080-81205383** | **ZCCHC24** | **3** |
| **490** | **chr 10: 83635069-84746935** | **NRG3** | **3** |
| **491** | **chr 10: 86004808-86019716** | **RGR** | **1** |
| **492** | **chr 10: 87359311-88126250** | **GRID1** | **3** |
| **492** | **chr 10: 87359311-88126250** | **GRID1-AS1** | **3** |
| **493** | **chr 10: 88516406-88692595** | **BMPR1A** | **3** |
| **494** | **chr 10: 88963631-89102369** | **NUTM2A-AS1** | **2** |
| **494** | **chr 10: 88963631-89102369** | **NUTM2A** | **2** |
| **495** | **chr 10: 89622869-89731687** | **PTEN** | **1** |
| **495** | **chr 10: 89622869-89731687** | **KLLN** | **1** |
| **496** | **chr 10: 90033620-90344287** | **RNLS** | **1** |
| **497** | **chr 10: 90750413-90775542** | **ACTA2** | **1** |
| **497** | **chr 10: 90750413-90775542** | **FAS** | **1** |
| **498** | **chr 10: 90973325-91174314** | **LIPA** | **1** |
| **499** | **chr 10: 91461366-91534700** | **KIF20B** | **1** |
| **500** | **chr 10: 92162277-92300562** | **none** | **1** |
| **501** | **chr 10: 93558068-93625033** | **TNKS2** | **1** |
| **502** | **chr 10: 94590934-94819250** | **EXOC6** | **2** |
| **503** | **chr 10: 94819419-94820183** | **CYP26C1** | **1** |
| **504** | **chr 10: 95517565-95557916** | **LGI1** | **2** |
| **505** | **chr 10: 95753745-96092580** | **PLCE1** | **2** |
| **506** | **chr 10: 96953956-96988685** | **C10ORF129** | **1** |
| **507** | **chr 10: 97365695-97416463** | **ALDH18A1** | **1** |
| **508** | **chr 10: 97512962-97849995** | **ENTPD1-AS1** | **1** |
| **508** | **chr 10: 97512962-97849995** | **ENTPD1** | **1** |
| **509** | **chr 10: 97709724-97792441** | **ENTPD1-AS1** | **1** |
| **510** | **chr 10: 97889471-97965044** | **ZNF518A** | **2** |
| **510** | **chr 10: 97889471-97965044** | **BLNK** | **2** |
| **511** | **chr 10: 97951457-98031344** | **BLNK** | **1** |
| **512** | **chr 10: 98592016-98740800** | **LCOR** | **1** |
| **513** | **chr 10: 98757794-99052413** | **LOC100505540** | **4** |
| **513** | **chr 10: 98757794-99052413** | **SLIT1** | **4** |
| **514** | **chr 10: 99344079-99436191** | **HOGA1** | **2** |
| **514** | **chr 10: 99344079-99436191** | **C10ORF62** | **2** |
| **515** | **chr 10: 99609995-99631337** | **CRTAC1** | **1** |
| **515** | **chr 10: 99609995-99631337** | **GOLGA7B** | **1** |
| **516** | **chr 10: 99624756-99790585** | **CRTAC1** | **1** |
| **516** | **chr 10: 99624756-99790585** | **GOLGA7B** | **1** |
| **517** | **chr 10: 100007446-100028007** | **LOXL4** | **1** |
| **518** | **chr 10: 100143321-100174941** | **MIR1287** | **2** |
| **518** | **chr 10: 100143321-100174941** | **PYROXD2** | **2** |
| **519** | **chr 10: 100218874-100995619** | **HPSE2** | **2** |
| **520** | **chr 10: 101088855-101154087** | **CNNM1** | **2** |
| **521** | **chr 10: 101542488-101611949** | **ABCC2** | **1** |
| **522** | **chr 10: 102246398-102289757** | **SEC31B** | **2** |
| **522** | **chr 10: 102246398-102289757** | **NDUFB8** | **2** |
| **523** | **chr 10: 102495359-102589698** | **PAX2** | **1** |
| **524** | **chr 10: 102989350-103029905** | **FLJ41350** | **2** |
| **524** | **chr 10: 102989350-103029905** | **LBX1** | **2** |
| **525** | **chr 10: 103989942-104001231** | **PITX3** | **1** |
| **526** | **chr 10: 104433487-104474164** | **ARL3** | **1** |
| **526** | **chr 10: 104433487-104474164** | **SFXN2** | **1** |
| **527** | **chr 10: 104678049-104849978** | **CNNM2** | **1** |
| **528** | **chr 10: 105239359-105277150** | **CALHM3** | **2** |
| **528** | **chr 10: 105239359-105277150** | **NEURL** | **2** |
| **529** | **chr 10: 105253735-105352309** | **NEURL** | **2** |
| **530** | **chr 10: 105348284-105615301** | **SH3PXD2A** | **5** |
| **530** | **chr 10: 105348284-105615301** | **NEURL** | **5** |
| **531** | **chr 10: 105889645-105992120** | **MIR609** | **1** |
| **531** | **chr 10: 105889645-105992120** | **WDR96** | **1** |
| **532** | **chr 10: 106113521-106214848** | **CCDC147** | **1** |
| **533** | **chr 10: 106400858-107024993** | **SORCS3** | **2** |
| **534** | **chr 10: 108333420-108924292** | **SORCS1** | **2** |
| **535** | **chr 10: 109517590-109871360** | **none** | **1** |
| **536** | **chr 10: 111756125-111895323** | **ADD3** | **1** |
| **536** | **chr 10: 111756125-111895323** | **LOC100505933** | **1** |
| **537** | **chr 10: 112257595-112271302** | **DUSP5** | **1** |
| **538** | **chr 10: 114206755-114578503** | **VTI1A** | **1** |
| **538** | **chr 10: 114206755-114578503** | **ZDHHC6** | **1** |
| **539** | **chr 10: 114710008-114927437** | **TCF7L2** | **2** |
| **540** | **chr 10: 116581502-116659591** | **FAM160B1** | **1** |
| **541** | **chr 10: 116853123-117708503** | **ATRNL1** | **1** |
| **542** | **chr 10: 118305442-118327367** | **PNLIP** | **1** |
| **543** | **chr 10: 118643741-118886097** | **KIAA1598** | **3** |
| **544** | **chr 10: 119039999-119134978** | **PDZD8** | **1** |
| **545** | **chr 10: 119184704-119249930** | **EMX2OS** | **1** |
| **546** | **chr 10: 119321594-119331968** | **EMX2** | **1** |
| **547** | **chr 10: 120001075-120027222** | **FAM204A** | **2** |
| **547** | **chr 10: 120001075-120027222** | **CASC2** | **2** |
| **548** | **chr 10: 120967100-121215131** | **GRK5** | **5** |
| **548** | **chr 10: 120967100-121215131** | **MIR4681** | **5** |
| **549** | **chr 10: 121485608-121588652** | **INPP5F** | **2** |
| **550** | **chr 10: 124221040-124274424** | **HTRA1** | **2** |
| **551** | **chr 10: 124320180-124403252** | **DMBT1** | **1** |
| **552** | **chr 10: 124516209-124558696** | **FLJ46361** | **1** |
| **553** | **chr 10: 125115965-125277224** | **none** | **3** |
| **554** | **chr 10: 125767183-125853206** | **CHST15** | **1** |
| **555** | **chr 10: 126676420-126849739** | **CTBP2** | **3** |
| **556** | **chr 10: 128113565-128359079** | **C10ORF90** | **3** |
| **557** | **chr 10: 128593977-129250781** | **FAM196A** | **4** |
| **557** | **chr 10: 128593977-129250781** | **DOCK1** | **4** |
| **558** | **chr 10: 129705324-129884119** | **PTPRE** | **1** |
| **559** | **chr 10: 132890653-133109984** | **TCERG1L** | **2** |
| **560** | **chr 10: 133918174-133998313** | **JAKMIP3** | **1** |
| **561** | **chr 10: 134210671-134231367** | **PWWP2B** | **1** |
| **562** | **chr 10: 134257830-134262912** | **C10ORF91** | **1** |
| **563** | **chr 10: 134351323-134596979** | **INPP5A** | **1** |
| **564** | **chr 10: 134757470-134778793** | **LOC399829** | **1** |
| **565** | **chr 10: 134779037-134789858** | **LOC399829** | **2** |
| **566** | **chr 10: 134884432-134945179** | **GPR123** | **2** |
| **567** | **chr 11: 369795-382116** | **B4GALNT4** | **1** |
| **568** | **chr 11: 405715-417455** | **SIGIRR** | **1** |
| **569** | **chr 11: 694437-727727** | **TMEM80** | **1** |
| **569** | **chr 11: 694437-727727** | **DEAF1** | **1** |
| **570** | **chr 11: 708563-727047** | **EPS8L2** | **1** |
| **571** | **chr 11: 790474-798316** | **SLC25A22** | **2** |
| **571** | **chr 11: 790474-798316** | **CEND1** | **2** |
| **572** | **chr 11: 1411128-1483919** | **BRSK2** | **1** |
| **573** | **chr 11: 1592582-1620414** | **MOB2** | **1** |
| **573** | **chr 11: 1592582-1620414** | **DUSP8** | **1** |
| **574** | **chr 11: 1874199-1913497** | **LSP1** | **1** |
| **574** | **chr 11: 1874199-1913497** | **MIR4298** | **1** |
| **575** | **chr 11: 2323226-2339430** | **TSPAN32** | **2** |
| **575** | **chr 11: 2323226-2339430** | **C11ORF21** | **2** |
| **576** | **chr 11: 2465913-2870339** | **KCNQ1** | **1** |
| **577** | **chr 11: 3108345-3187969** | **OSBPL5** | **3** |
| **578** | **chr 11: 3631130-3658789** | **TRPC2** | **1** |
| **579** | **chr 11: 4115936-4160106** | **RRM1** | **1** |
| **580** | **chr 11: 5274419-5667019** | **HBE1** | **2** |
| **580** | **chr 11: 5274419-5667019** | **HBG2** | **2** |
| **581** | **chr 11: 7041676-7092539** | **ZNF214** | **1** |
| **581** | **chr 11: 7041676-7092539** | **NLRP14** | **1** |
| **582** | **chr 11: 7260008-7490273** | **SYT9** | **1** |
| **583** | **chr 11: 7710668-7927502** | **OVCH2** | **2** |
| **583** | **chr 11: 7710668-7927502** | **OR5P2** | **2** |
| **584** | **chr 11: 7991797-8023409** | **EIF3F** | **1** |
| **585** | **chr 11: 8127596-8190602** | **TUB** | **1** |
| **586** | **chr 11: 8413417-8615836** | **STK33** | **3** |
| **587** | **chr 11: 8714897-8932498** | **ST5** | **2** |
| **588** | **chr 11: 9800213-10315754** | **SBF2-AS1** | **2** |
| **588** | **chr 11: 9800213-10315754** | **SBF2** | **2** |
| **589** | **chr 11: 10329859-10529126** | **AMPD3** | **1** |
| **590** | **chr 11: 10578512-10715535** | **MRVI1-AS1** | **1** |
| **590** | **chr 11: 10578512-10715535** | **LYVE1** | **1** |
| **591** | **chr 11: 11292422-11643552** | **CSNK2A3** | **2** |
| **591** | **chr 11: 11292422-11643552** | **GALNT18** | **2** |
| **592** | **chr 11: 12398731-12552348** | **PARVA** | **1** |
| **593** | **chr 11: 12695968-12966298** | **TEAD1** | **1** |
| **594** | **chr 11: 13298198-13408813** | **ARNTL** | **1** |
| **595** | **chr 11: 13409547-13484844** | **BTBD10** | **1** |
| **596** | **chr 11: 13806936-13869549** | **none** | **1** |
| **597** | **chr 11: 13848389-13901046** | **none** | **1** |
| **598** | **chr 11: 13983913-14289646** | **SPON1** | **2** |
| **599** | **chr 11: 14299471-14386052** | **RRAS2** | **1** |
| **600** | **chr 11: 14665268-14892350** | **PSMA1** | **1** |
| **601** | **chr 11: 15723508-15780444** | **none** | **3** |
| **602** | **chr 11: 15932502-15948989** | **SOX6** | **2** |
| **603** | **chr 11: 15987994-16761138** | **SOX6** | **1** |
| **604** | **chr 11: 16799841-17035990** | **PLEKHA7** | **1** |
| **605** | **chr 11: 17229699-17371521** | **NUCB2** | **1** |
| **606** | **chr 11: 17373272-17398888** | **NCR3LG1** | **1** |
| **607** | **chr 11: 17414431-17498449** | **ABCC8** | **1** |
| **608** | **chr 11: 17515441-17565963** | **USH1C** | **1** |
| **609** | **chr 11: 17568919-17668697** | **USH1C** | **1** |
| **610** | **chr 11: 17756358-17804602** | **KCNC1** | **3** |
| **611** | **chr 11: 18343841-18388591** | **HPS5** | **2** |
| **611** | **chr 11: 18343841-18388591** | **GTF2H1** | **2** |
| **612** | **chr 11: 18433853-18473605** | **LDHC** | **1** |
| **613** | **chr 11: 18714668-18726332** | **TMEM86A** | **1** |
| **613** | **chr 11: 18714668-18726332** | **IGSF22** | **1** |
| **614** | **chr 11: 19218321-19302973** | **CSRP3** | **1** |
| **614** | **chr 11: 19218321-19302973** | **E2F8** | **1** |
| **615** | **chr 11: 19372270-20143144** | **NAV2** | **3** |
| **615** | **chr 11: 19372270-20143144** | **NAV2-AS5** | **3** |
| **616** | **chr 11: 20620945-20680831** | **SLC6A5** | **2** |
| **617** | **chr 11: 20691116-21597227** | **NELL1** | **8** |
| **618** | **chr 11: 22646859-22834601** | **FANCF** | **1** |
| **618** | **chr 11: 22646859-22834601** | **GAS2** | **1** |
| **619** | **chr 11: 24518515-25104150** | **LUZP2** | **6** |
| **620** | **chr 11: 27068732-27241660** | **BBOX1** | **1** |
| **621** | **chr 11: 27676439-27899195** | **BDNF-AS** | **1** |
| **622** | **chr 11: 28042166-28129855** | **KIF18A** | **1** |
| **622** | **chr 11: 28042166-28129855** | **MIR610** | **1** |
| **623** | **chr 11: 28724161-29085368** | **none** | **1** |
| **624** | **chr 11: 30065604-30105890** | **KCNA4** | **3** |
| **625** | **chr 11: 30406039-30608419** | **MPPED2** | **2** |
| **626** | **chr 11: 30851915-31391357** | **DCDC5** | **3** |
| **626** | **chr 11: 30851915-31391357** | **DCDC1** | **3** |
| **627** | **chr 11: 31531296-31805546** | **IMMP1L** | **3** |
| **627** | **chr 11: 31531296-31805546** | **ELP4** | **3** |
| **628** | **chr 11: 31658874-31789501** | **ELP4** | **1** |
| **629** | **chr 11: 31806339-31839509** | **PAX6** | **2** |
| **630** | **chr 11: 31833938-32127301** | **PAX6** | **3** |
| **631** | **chr 11: 32133594-32364955** | **WT1** | **2** |
| **631** | **chr 11: 32133594-32364955** | **RCN1** | **2** |
| **632** | **chr 11: 33098733-33183917** | **LINC00294** | **1** |
| **632** | **chr 11: 33098733-33183917** | **CSTF3** | **1** |
| **633** | **chr 11: 33563617-33695648** | **KIAA1549L** | **1** |
| **634** | **chr 11: 34500339-34535352** | **ELF5** | **1** |
| **635** | **chr 11: 34874640-34938046** | **PDHX** | **1** |
| **635** | **chr 11: 34874640-34938046** | **APIP** | **1** |
| **636** | **chr 11: 35160416-35253949** | **CD44** | **1** |
| **637** | **chr 11: 35684352-35829775** | **TRIM44** | **2** |
| **638** | **chr 11: 35965530-36253686** | **MIR3973** | **1** |
| **638** | **chr 11: 35965530-36253686** | **LDLRAD3** | **1** |
| **639** | **chr 11: 36317837-36486754** | **PRR5L** | **1** |
| **640** | **chr 11: 40135752-41481323** | **LRRC4C** | **4** |
| **641** | **chr 11: 43380481-43516483** | **TTC17** | **1** |
| **642** | **chr 11: 43577985-43878167** | **MIR670** | **1** |
| **642** | **chr 11: 43577985-43878167** | **MIR129-2** | **1** |
| **643** | **chr 11: 44748014-44953972** | **TSPAN18** | **5** |
| **643** | **chr 11: 44748014-44953972** | **TP53I11** | **5** |
| **644** | **chr 11: 44907453-44972840** | **TSPAN18** | **1** |
| **644** | **chr 11: 44907453-44972840** | **TP53I11** | **1** |
| **645** | **chr 11: 45115563-45247734** | **PRDM11** | **4** |
| **646** | **chr 11: 45237365-45256843** | **PRDM11** | **1** |
| **647** | **chr 11: 45392947-45410059** | **none** | **1** |
| **648** | **chr 11: 45868668-45904798** | **CRY2** | **1** |
| **649** | **chr 11: 45907201-45928016** | **MAPK8IP1** | **1** |
| **650** | **chr 11: 45943171-45950647** | **GYLTL1B** | **1** |
| **651** | **chr 11: 46354454-46402104** | **DGKZ** | **1** |
| **652** | **chr 11: 46417963-46615675** | **AMBRA1** | **1** |
| **653** | **chr 11: 47290711-47351582** | **MADD** | **1** |
| **654** | **chr 11: 47404698-47430741** | **SLC39A13** | **1** |
| **655** | **chr 11: 47608197-47610746** | **FAM180B** | **1** |
| **656** | **chr 11: 47799638-47870107** | **NUP160** | **1** |
| **657** | **chr 11: 56615953-56645554** | **none** | **3** |
| **658** | **chr 11: 57067111-57092426** | **TNKS1BP1** | **1** |
| **659** | **chr 11: 57480071-57587018** | **MED19** | **3** |
| **659** | **chr 11: 57480071-57587018** | **TMX2** | **3** |
| **660** | **chr 11: 57791352-57949088** | **OR9Q1** | **1** |
| **660** | **chr 11: 57791352-57949088** | **OR6Q1** | **1** |
| **661** | **chr 11: 58897605-58910488** | **FAM111A** | **2** |
| **662** | **chr 11: 58938902-58976060** | **DTX4** | **1** |
| **662** | **chr 11: 58938902-58976060** | **MPEG1** | **1** |
| **663** | **chr 11: 59404188-59436453** | **PATL1** | **1** |
| **664** | **chr 11: 60685940-60692869** | **TMEM109** | **1** |
| **664** | **chr 11: 60685940-60692869** | **TMEM132A** | **1** |
| **665** | **chr 11: 60739114-60787849** | **CD6** | **1** |
| **666** | **chr 11: 60869866-60895324** | **CD5** | **1** |
| **667** | **chr 11: 61025761-61062896** | **VWCE** | **1** |
| **668** | **chr 11: 61282784-61348620** | **SYT7** | **2** |
| **669** | **chr 11: 61664772-61687741** | **RAB3IL1** | **2** |
| **670** | **chr 11: 62457746-62494856** | **LRRN4CL** | **2** |
| **670** | **chr 11: 62457746-62494856** | **BSCL2** | **2** |
| **671** | **chr 11: 62676150-62689279** | **CHRM1** | **1** |
| **672** | **chr 11: 63448917-63527363** | **RTN3** | **1** |
| **673** | **chr 11: 63606399-63678491** | **MARK2** | **2** |
| **674** | **chr 11: 63766029-63933578** | **FLRT1** | **2** |
| **674** | **chr 11: 63766029-63933578** | **MACROD1** | **2** |
| **675** | **chr 11: 63803441-63886645** | **FLRT1** | **1** |
| **675** | **chr 11: 63803441-63886645** | **MACROD1** | **1** |
| **676** | **chr 11: 64358112-64369820** | **SLC22A12** | **1** |
| **677** | **chr 11: 64373645-64490660** | **NRXN2** | **3** |
| **678** | **chr 11: 64570981-64578766** | **MAP4K2** | **1** |
| **678** | **chr 11: 64570981-64578766** | **MEN1** | **1** |
| **679** | **chr 11: 64981310-65010228** | **SLC22A20** | **3** |
| **680** | **chr 11: 65154069-65180996** | **FRMD8** | **2** |
| **681** | **chr 11: 65837833-66012218** | **PACS1** | **2** |
| **682** | **chr 11: 66247483-66301098** | **BBS1** | **1** |
| **682** | **chr 11: 66247483-66301098** | **DPP3** | **1** |
| **683** | **chr 11: 66615703-66725847** | **PC** | **1** |
| **684** | **chr 11: 67118247-67141648** | **POLD4** | **1** |
| **684** | **chr 11: 67118247-67141648** | **LOC100130987** | **1** |
| **685** | **chr 11: 67202980-67211292** | **CORO1B** | **1** |
| **685** | **chr 11: 67202980-67211292** | **PTPRCAP** | **1** |
| **686** | **chr 11: 67758574-67772452** | **UNC93B1** | **1** |
| **687** | **chr 11: 69061604-69182494** | **MYEOV** | **1** |
| **688** | **chr 11: 69924407-70035634** | **ANO1** | **2** |
| **689** | **chr 11: 70313960-70963623** | **SHANK2** | **7** |
| **690** | **chr 11: 71164154-71235153** | **NADSYN1** | **1** |
| **691** | **chr 11: 71292900-71318487** | **KRTAP5-11** | **1** |
| **692** | **chr 11: 71314305-71319086** | **KRTAP5-11** | **1** |
| **693** | **chr 11: 71567322-71574704** | **DEFB108B** | **1** |
| **693** | **chr 11: 71567322-71574704** | **LOC100133315** | **1** |
| **694** | **chr 11: 71639746-71708643** | **LOC100133315** | **1** |
| **695** | **chr 11: 72003468-72145692** | **CLPB** | **2** |
| **696** | **chr 11: 72287184-72385635** | **PDE2A** | **1** |
| **697** | **chr 11: 72396113-72504726** | **ARAP1** | **2** |
| **698** | **chr 11: 72547789-72853306** | **FCHSD2** | **1** |
| **699** | **chr 11: 73019333-73080136** | **ARHGEF17** | **1** |
| **700** | **chr 11: 73386682-73472182** | **RAB6A** | **1** |
| **701** | **chr 11: 74811607-74917594** | **SLCO2B1** | **4** |
| **702** | **chr 11: 75145684-75236948** | **GDPD5** | **1** |
| **703** | **chr 11: 75428863-75444003** | **MOGAT2** | **1** |
| **704** | **chr 11: 75526211-75854239** | **UVRAG** | **1** |
| **705** | **chr 11: 76839309-76926284** | **MYO7A** | **1** |
| **706** | **chr 11: 76927602-77012732** | **GDPD4** | **2** |
| **707** | **chr 11: 77032751-77185680** | **PAK1** | **1** |
| **708** | **chr 11: 77225980-77348850** | **CLNS1A** | **1** |
| **708** | **chr 11: 77225980-77348850** | **AQP11** | **1** |
| **709** | **chr 11: 77926342-78129394** | **GAB2** | **1** |
| **710** | **chr 11: 78035803-78155095** | **GAB2** | **1** |
| **711** | **chr 11: 78363875-79151992** | **MIR708** | **5** |
| **711** | **chr 11: 78363875-79151992** | **TENM4** | **5** |
| **712** | **chr 11: 80032663-80038955** | **none** | **1** |
| **713** | **chr 11: 81590892-82429124** | **MIR4300** | **3** |
| **714** | **chr 11: 82534543-82782965** | **PRCP** | **1** |
| **714** | **chr 11: 82534543-82782965** | **C11ORF82** | **1** |
| **715** | **chr 11: 82904780-82971736** | **ANKRD42** | **1** |
| **716** | **chr 11: 83166054-85338966** | **DLG2** | **8** |
| **717** | **chr 11: 86085777-86134151** | **CCDC81** | **1** |
| **718** | **chr 11: 89057523-89322779** | **NOX4** | **1** |
| **719** | **chr 11: 89553249-89560090** | **TRIM49** | **1** |
| **719** | **chr 11: 89553249-89560090** | **MIR5692A1** | **1** |
| **720** | **chr 11: 89750778-89757679** | **TRIM53AP** | **1** |
| **720** | **chr 11: 89750778-89757679** | **TRIM49C** | **1** |
| **721** | **chr 11: 89984399-90648220** | **MIR4490** | **3** |
| **722** | **chr 11: 92085261-92629618** | **FAT3** | **1** |
| **723** | **chr 11: 92133356-92141342** | **FAT3** | **3** |
| **724** | **chr 11: 94152894-94227074** | **MRE11A** | **1** |
| **724** | **chr 11: 94152894-94227074** | **ANKRD49** | **1** |
| **725** | **chr 11: 95566045-95658479** | **MTMR2** | **1** |
| **726** | **chr 11: 95709761-96076344** | **MAML2** | **1** |
| **726** | **chr 11: 95709761-96076344** | **MIR1260B** | **1** |
| **727** | **chr 11: 98891682-100229616** | **CNTN5** | **7** |
| **728** | **chr 11: 100558383-100862668** | **TMEM133** | **1** |
| **728** | **chr 11: 100558383-100862668** | **ARHGAP42** | **1** |
| **729** | **chr 11: 100900354-101001255** | **PGR** | **2** |
| **730** | **chr 11: 101018880-101080322** | **LOC101054525** | **1** |
| **731** | **chr 11: 101981191-102104154** | **YAP1** | **1** |
| **732** | **chr 11: 102337985-102369532** | **MMP7** | **1** |
| **732** | **chr 11: 102337985-102369532** | **TMEM123** | **1** |
| **733** | **chr 11: 102617698-102707497** | **WTAPP1** | **2** |
| **733** | **chr 11: 102617698-102707497** | **MMP10** | **2** |
| **734** | **chr 11: 102932804-102962944** | **DCUN1D5** | **1** |
| **735** | **chr 11: 103777913-104035107** | **PDGFD** | **1** |
| **735** | **chr 11: 103777913-104035107** | **DDI1** | **1** |
| **736** | **chr 11: 104756444-104769397** | **CASP12** | **1** |
| **737** | **chr 11: 105028653-105402424** | **CARD18** | **1** |
| **738** | **chr 11: 106544737-106889250** | **GUCY1A2** | **4** |
| **739** | **chr 11: 107661716-107799019** | **RAB39A** | **3** |
| **739** | **chr 11: 107661716-107799019** | **SLC35F2** | **3** |
| **740** | **chr 11: 108376157-108464465** | **EXPH5** | **11** |
| **741** | **chr 11: 108535751-108811657** | **DDX10** | **4** |
| **742** | **chr 11: 109225811-109454633** | **C11ORF87** | **1** |
| **743** | **chr 11: 110045604-110167447** | **RDX** | **1** |
| **744** | **chr 11: 110225854-110277124** | **FDX1** | **1** |
| **745** | **chr 11: 111895537-111935114** | **PIH1D2** | **1** |
| **745** | **chr 11: 111895537-111935114** | **DLAT** | **1** |
| **746** | **chr 11: 112141471-112233257** | **C11ORF34** | **2** |
| **747** | **chr 11: 112263840-112492452** | **none** | **1** |
| **748** | **chr 11: 113149158-113185159** | **TTC12** | **3** |
| **749** | **chr 11: 113558271-113577095** | **TMPRSS5** | **1** |
| **750** | **chr 11: 113668595-113746292** | **USP28** | **1** |
| **751** | **chr 11: 113845602-113861035** | **HTR3A** | **1** |
| **752** | **chr 11: 113930314-114121398** | **ZBTB16** | **2** |
| **753** | **chr 11: 114081337-114227293** | **ZBTB16** | **1** |
| **754** | **chr 11: 115039937-115375675** | **CADM1** | **5** |
| **755** | **chr 11: 115530375-115713864** | **LOC283143** | **1** |
| **756** | **chr 11: 116691418-116694022** | **APOA4** | **1** |
| **757** | **chr 11: 116714117-116969153** | **SIK3** | **1** |
| **758** | **chr 11: 117049448-117068160** | **LOC100652768** | **1** |
| **758** | **chr 11: 117049448-117068160** | **SIDT2** | **1** |
| **759** | **chr 11: 117075052-117103241** | **PCSK7** | **1** |
| **759** | **chr 11: 117075052-117103241** | **TAGLN** | **1** |
| **760** | **chr 11: 117298488-117748201** | **FXYD2** | **8** |
| **760** | **chr 11: 117298488-117748201** | **DSCAML1** | **8** |
| **761** | **chr 11: 118307204-118397539** | **TTC36** | **1** |
| **761** | **chr 11: 118307204-118397539** | **MLL** | **1** |
| **762** | **chr 11: 118967212-118979041** | **DPAGT1** | **1** |
| **762** | **chr 11: 118967212-118979041** | **C2CD2L** | **1** |
| **763** | **chr 11: 119019721-119033360** | **ABCG4** | **1** |
| **764** | **chr 11: 119252487-119397374** | **USP2** | **1** |
| **764** | **chr 11: 119252487-119397374** | **LOC100499227** | **1** |
| **765** | **chr 11: 119479134-119529994** | **PVRL1** | **1** |
| **766** | **chr 11: 119494119-119599794** | **PVRL1** | **2** |
| **767** | **chr 11: 119580629-119584635** | **PVRL1** | **1** |
| **768** | **chr 11: 120081474-120101041** | **OAF** | **1** |
| **769** | **chr 11: 120207786-120360645** | **ARHGEF12** | **1** |
| **770** | **chr 11: 120382467-120859613** | **GRIK4** | **1** |
| **771** | **chr 11: 121899062-121987031** | **MIR125B1** | **1** |
| **771** | **chr 11: 121899062-121987031** | **MIR100HG** | **1** |
| **772** | **chr 11: 122026129-122293579** | **MIR100HG** | **2** |
| **773** | **chr 11: 122943034-123065989** | **CLMP** | **2** |
| **774** | **chr 11: 123059509-123098985** | **CLMP** | **4** |
| **775** | **chr 11: 123499894-123525952** | **SCN3B** | **1** |
| **776** | **chr 11: 125034582-125303285** | **PKNOX2** | **6** |
| **777** | **chr 11: 125970399-125972308** | **CDON** | **1** |
| **778** | **chr 11: 126081308-126132881** | **RPUSD4** | **1** |
| **778** | **chr 11: 126081308-126132881** | **FAM118B** | **1** |
| **779** | **chr 11: 126293253-126873355** | **KIRREL3** | **2** |
| **780** | **chr 11: 126891648-126923448** | **KIRREL3-AS3** | **1** |
| **781** | **chr 11: 126989220-127009630** | **none** | **1** |
| **782** | **chr 11: 128328655-128457453** | **ETS1** | **5** |
| **783** | **chr 11: 128485761-128491701** | **ETS1** | **1** |
| **784** | **chr 11: 128499547-128556817** | **FLI1** | **2** |
| **785** | **chr 11: 128556429-128683162** | **FLI1-AS1** | **2** |
| **785** | **chr 11: 128556429-128683162** | **FLI1** | **2** |
| **786** | **chr 11: 129245834-129322171** | **BARX2** | **2** |
| **787** | **chr 11: 129769600-129872730** | **PRDM10** | **1** |
| **788** | **chr 11: 131240372-132206716** | **NTM** | **7** |
| **789** | **chr 11: 131372652-131410761** | **NTM** | **1** |
| **790** | **chr 11: 132284870-133402414** | **OPCML** | **4** |
| **791** | **chr 12: 73724-91218** | **LOC100288778** | **1** |
| **792** | **chr 12: 175930-287626** | **IQSEC3** | **1** |
| **793** | **chr 12: 246576-258335** | **IQSEC3** | **1** |
| **794** | **chr 12: 389294-498620** | **CCDC77** | **5** |
| **794** | **chr 12: 389294-498620** | **KDM5A** | **5** |
| **795** | **chr 12: 569529-672675** | **B4GALNT3** | **3** |
| **796** | **chr 12: 673461-772945** | **NINJ2** | **1** |
| **797** | **chr 12: 1021242-1100356** | **ERC1** | **1** |
| **797** | **chr 12: 1021242-1100356** | **RAD52** | **1** |
| **798** | **chr 12: 1099674-1605099** | **ERC1** | **4** |
| **799** | **chr 12: 1489225-1500668** | **ERC1** | **2** |
| **800** | **chr 12: 2079951-2802108** | **CACNA1C** | **4** |
| **800** | **chr 12: 2079951-2802108** | **DCP1B** | **4** |
| **801** | **chr 12: 2851791-2881074** | **LOC283440** | **1** |
| **802** | **chr 12: 2921787-2968957** | **ITFG2** | **2** |
| **802** | **chr 12: 2921787-2968957** | **NRIP2** | **2** |
| **803** | **chr 12: 2958396-2966213** | **LOC100507424** | **1** |
| **804** | **chr 12: 3490514-3703139** | **PRMT8** | **1** |
| **805** | **chr 12: 3715798-3873985** | **EFCAB4B** | **2** |
| **806** | **chr 12: 4357930-4385350** | **CCND2** | **6** |
| **807** | **chr 12: 4382937-4414516** | **CCND2** | **1** |
| **808** | **chr 12: 4647949-4669214** | **RAD51AP1** | **2** |
| **808** | **chr 12: 4647949-4669214** | **C12ORF4** | **2** |
| **809** | **chr 12: 4671369-4960277** | **DYRK4** | **3** |
| **809** | **chr 12: 4671369-4960277** | **AKAP3** | **3** |
| **810** | **chr 12: 5153084-5155949** | **KCNA5** | **1** |
| **811** | **chr 12: 5541277-5630702** | **NTF3** | **1** |
| **812** | **chr 12: 5641034-6055398** | **ANO2** | **6** |
| **813** | **chr 12: 6308880-6347425** | **CD9** | **1** |
| **814** | **chr 12: 6775642-6798738** | **ZNF384** | **1** |
| **815** | **chr 12: 6802957-6809966** | **PIANP** | **1** |
| **816** | **chr 12: 7187512-7261869** | **C1R** | **1** |
| **816** | **chr 12: 7187512-7261869** | **C1RL** | **1** |
| **817** | **chr 12: 7282293-7311541** | **RBP5** | **1** |
| **817** | **chr 12: 7282293-7311541** | **CLSTN3** | **1** |
| **818** | **chr 12: 8662070-8674962** | **CLEC4D** | **1** |
| **819** | **chr 12: 9429830-9466684** | **LOC642846** | **1** |
| **820** | **chr 12: 9570308-9600825** | **DDX12P** | **1** |
| **821** | **chr 12: 10138240-10166023** | **CLEC12B** | **1** |
| **821** | **chr 12: 10138240-10166023** | **CLEC1B** | **1** |
| **822** | **chr 12: 10163225-10171218** | **CLEC12B** | **1** |
| **823** | **chr 12: 10167838-10183205** | **CLEC12B** | **1** |
| **824** | **chr 12: 10771537-10826917** | **STYK1** | **1** |
| **825** | **chr 12: 10977558-11324212** | **PRR4** | **2** |
| **825** | **chr 12: 10977558-11324212** | **TAS2R10** | **2** |
| **826** | **chr 12: 11504756-11653975** | **PRB1** | **3** |
| **826** | **chr 12: 11504756-11653975** | **PRB2** | **3** |
| **827** | **chr 12: 11802787-12048336** | **RNU6-19** | **3** |
| **827** | **chr 12: 11802787-12048336** | **ETV6** | **3** |
| **828** | **chr 12: 12202777-12364018** | **BCL2L14** | **1** |
| **829** | **chr 12: 12268958-12419946** | **LRP6** | **1** |
| **830** | **chr 12: 12878850-12982915** | **MIR613** | **2** |
| **830** | **chr 12: 12878850-12982915** | **APOLD1** | **2** |
| **831** | **chr 12: 13693164-14133053** | **GRIN2B** | **2** |
| **832** | **chr 12: 14765575-14849519** | **GUCY2C** | **1** |
| **833** | **chr 12: 14818588-14910897** | **GUCY2C** | **1** |
| **834** | **chr 12: 15260716-15501609** | **PTPRO** | **1** |
| **834** | **chr 12: 15260716-15501609** | **RERG** | **1** |
| **835** | **chr 12: 15475330-15750333** | **PTPRO** | **1** |
| **836** | **chr 12: 16035324-16056412** | **STRAP** | **1** |
| **837** | **chr 12: 16341418-16430619** | **SLC15A5** | **2** |
| **838** | **chr 12: 19556978-19873735** | **AEBP2** | **3** |
| **839** | **chr 12: 20167713-20251802** | **LOC100506393** | **1** |
| **840** | **chr 12: 20963635-21392180** | **SLCO1B3** | **3** |
| **840** | **chr 12: 20963635-21392180** | **SLCO1B7** | **3** |
| **841** | **chr 12: 21689122-21757781** | **GYS2** | **1** |
| **842** | **chr 12: 21950334-22094336** | **ABCC9** | **3** |
| **843** | **chr 12: 21980143-22040891** | **ABCC9** | **2** |
| **844** | **chr 12: 22216706-22589975** | **CMAS** | **2** |
| **844** | **chr 12: 22216706-22589975** | **ST8SIA1** | **2** |
| **845** | **chr 12: 22852792-23344521** | **ETNK1** | **3** |
| **846** | **chr 12: 23682439-24103966** | **SOX5** | **1** |
| **847** | **chr 12: 24366189-24715524** | **SOX5** | **2** |
| **848** | **chr 12: 24857498-24927147** | **BCAT1** | **1** |
| **849** | **chr 12: 25348149-25362579** | **CASC1** | **1** |
| **849** | **chr 12: 25348149-25362579** | **LYRM5** | **1** |
| **850** | **chr 12: 26274923-26452223** | **SSPN** | **2** |
| **850** | **chr 12: 26274923-26452223** | **BHLHE41** | **2** |
| **851** | **chr 12: 26364096-26488789** | **SSPN** | **2** |
| **852** | **chr 12: 26490341-26986131** | **ITPR2** | **2** |
| **853** | **chr 12: 26776301-26802412** | **ITPR2** | **2** |
| **854** | **chr 12: 27676363-27848497** | **PPFIBP1** | **2** |
| **854** | **chr 12: 27676363-27848497** | **REP15** | **2** |
| **855** | **chr 12: 27932952-27955973** | **KLHL42** | **1** |
| **856** | **chr 12: 28286181-28732883** | **CCDC91** | **1** |
| **857** | **chr 12: 30862485-30907885** | **CAPRIN2** | **2** |
| **858** | **chr 12: 31226778-31257725** | **DDX11-AS1** | **1** |
| **858** | **chr 12: 31226778-31257725** | **DDX11** | **1** |
| **859** | **chr 12: 31824070-31882108** | **AMN1** | **1** |
| **860** | **chr 12: 32259768-32536567** | **BICD1** | **2** |
| **861** | **chr 12: 33527172-33592754** | **SYT10** | **1** |
| **862** | **chr 12: 39040623-39301232** | **CPNE8** | **1** |
| **863** | **chr 12: 40590545-40763087** | **LRRK2** | **1** |
| **864** | **chr 12: 40787196-40964632** | **LRRK2** | **2** |
| **865** | **chr 12: 41086243-41466220** | **CNTN1** | **1** |
| **866** | **chr 12: 42550905-42632151** | **YAF2** | **1** |
| **867** | **chr 12: 42632248-42859930** | **YAF2** | **4** |
| **868** | **chr 12: 44229769-44783545** | **TMEM117** | **2** |
| **869** | **chr 12: 44902057-45315631** | **NELL2** | **4** |
| **870** | **chr 12: 45566846-45609824** | **RNY5** | **1** |
| **870** | **chr 12: 45566846-45609824** | **PLEKHA8P1** | **1** |
| **871** | **chr 12: 47473385-47630443** | **AMIGO2** | **1** |
| **872** | **chr 12: 48057069-48099844** | **RPAP3** | **1** |
| **873** | **chr 12: 48498921-48540187** | **SENP1** | **1** |
| **874** | **chr 12: 49159974-49182820** | **ADCY6** | **2** |
| **875** | **chr 12: 49250927-49259681** | **RND1** | **1** |
| **876** | **chr 12: 49483203-49488602** | **DHH** | **1** |
| **877** | **chr 12: 49760366-49921205** | **LOC100335030** | **1** |
| **877** | **chr 12: 49760366-49921205** | **SPATS2** | **1** |
| **878** | **chr 12: 50101507-50158717** | **FMNL3** | **1** |
| **878** | **chr 12: 50101507-50158717** | **TMBIM6** | **1** |
| **879** | **chr 12: 50370705-50426919** | **AQP6** | **1** |
| **879** | **chr 12: 50370705-50426919** | **RACGAP1** | **1** |
| **880** | **chr 12: 50898767-51142450** | **DIP2B** | **1** |
| **881** | **chr 12: 51150674-51151216** | **DIP2B** | **1** |
| **881** | **chr 12: 51150674-51151216** | **ATF1** | **1** |
| **882** | **chr 12: 51785100-51902980** | **GALNT6** | **1** |
| **882** | **chr 12: 51785100-51902980** | **SLC4A8** | **1** |
| **883** | **chr 12: 51984049-52206648** | **SCN8A** | **1** |
| **884** | **chr 12: 52217286-52229280** | **FIGNL2** | **2** |
| **885** | **chr 12: 52626303-52702947** | **KRT81** | **1** |
| **885** | **chr 12: 52626303-52702947** | **KRT7** | **1** |
| **886** | **chr 12: 52774243-52796191** | **KRT82** | **1** |
| **886** | **chr 12: 52774243-52796191** | **KRT84** | **1** |
| **887** | **chr 12: 52979372-52995322** | **KRT72** | **1** |
| **888** | **chr 12: 53604353-53626764** | **RARG** | **1** |
| **889** | **chr 12: 53900471-54020199** | **NPFF** | **2** |
| **889** | **chr 12: 53900471-54020199** | **ATF7** | **2** |
| **890** | **chr 12: 53907767-53911392** | **ATF7** | **1** |
| **891** | **chr 12: 54356091-54368740** | **HOTAIR** | **1** |
| **892** | **chr 12: 54366909-54371427** | **HOXC11** | **1** |
| **892** | **chr 12: 54366909-54371427** | **HOTAIR** | **1** |
| **893** | **chr 12: 54375292-54379303** | **HOXC10** | **1** |
| **894** | **chr 12: 54378848-54449814** | **HOXC10** | **5** |
| **894** | **chr 12: 54378848-54449814** | **MIR196A2** | **5** |
| **895** | **chr 12: 54452037-54516018** | **LOC100240735** | **2** |
| **895** | **chr 12: 54452037-54516018** | **FLJ12825** | **2** |
| **896** | **chr 12: 54478915-54519776** | **FLJ12825** | **1** |
| **897** | **chr 12: 54624723-54673886** | **CBX5** | **1** |
| **898** | **chr 12: 54747444-54891472** | **ZNF385A** | **1** |
| **898** | **chr 12: 54747444-54891472** | **GPR84** | **1** |
| **899** | **chr 12: 54762916-54785082** | **ZNF385A** | **1** |
| **900** | **chr 12: 54943133-54973023** | **PDE1B** | **2** |
| **901** | **chr 12: 55341801-55378530** | **TESPA1** | **1** |
| **902** | **chr 12: 55828520-55979255** | **OR6C2** | **1** |
| **902** | **chr 12: 55828520-55979255** | **OR6C70** | **1** |
| **903** | **chr 12: 56109819-56118489** | **BLOC1S1-RDH5** | **1** |
| **903** | **chr 12: 56109819-56118489** | **BLOC1S1** | **1** |
| **904** | **chr 12: 56119106-56123491** | **CD63** | **1** |
| **905** | **chr 12: 56321102-56347811** | **WIBG** | **2** |
| **906** | **chr 12: 56546039-56557280** | **MYL6** | **1** |
| **906** | **chr 12: 56546039-56557280** | **MYL6B** | **1** |
| **907** | **chr 12: 56915712-56984745** | **RBMS2** | **1** |
| **908** | **chr 12: 57345218-57353158** | **RDH16** | **1** |
| **909** | **chr 12: 57403783-57444982** | **TAC3** | **1** |
| **909** | **chr 12: 57403783-57444982** | **MYO1A** | **1** |
| **910** | **chr 12: 57449425-57481846** | **TMEM194A** | **1** |
| **910** | **chr 12: 57449425-57481846** | **NAB2** | **1** |
| **911** | **chr 12: 57489190-57525922** | **STAT6** | **1** |
| **911** | **chr 12: 57489190-57525922** | **NAB2** | **1** |
| **912** | **chr 12: 58937906-59206842** | **none** | **1** |
| **913** | **chr 12: 58959742-59175529** | **none** | **1** |
| **914** | **chr 12: 62102039-62672931** | **FAM19A2** | **1** |
| **914** | **chr 12: 62102039-62672931** | **USP15** | **1** |
| **915** | **chr 12: 63686408-63753817** | **none** | **1** |
| **916** | **chr 12: 63952692-64189498** | **DPY19L2** | **1** |
| **916** | **chr 12: 63952692-64189498** | **TMEM5** | **1** |
| **917** | **chr 12: 64238072-64541613** | **SRGAP1** | **1** |
| **918** | **chr 12: 64580095-64616076** | **C12ORF66** | **3** |
| **919** | **chr 12: 64616116-64790845** | **C12ORF56** | **1** |
| **919** | **chr 12: 64616116-64790845** | **C12ORF66** | **1** |
| **920** | **chr 12: 64660216-64784972** | **C12ORF56** | **1** |
| **921** | **chr 12: 64798129-64844907** | **XPOT** | **1** |
| **921** | **chr 12: 64798129-64844907** | **TBK1** | **1** |
| **922** | **chr 12: 65004292-65091347** | **RASSF3** | **2** |
| **922** | **chr 12: 65004292-65091347** | **MIR548C** | **2** |
| **923** | **chr 12: 65277173-65371302** | **FLJ41278** | **1** |
| **924** | **chr 12: 65860599-66036152** | **MSRB3** | **1** |
| **925** | **chr 12: 66151799-66220754** | **RPSAP52** | **1** |
| **925** | **chr 12: 66151799-66220754** | **HMGA2** | **1** |
| **926** | **chr 12: 66516841-66563852** | **TMBIM4** | **1** |
| **926** | **chr 12: 66516841-66563852** | **LLPH** | **1** |
| **927** | **chr 12: 66741210-67197966** | **GRIP1** | **1** |
| **928** | **chr 12: 67913608-67960906** | **none** | **2** |
| **929** | **chr 12: 68052770-68064699** | **DYRK2** | **1** |
| **930** | **chr 12: 68383224-68628466** | **IFNG** | **1** |
| **930** | **chr 12: 68383224-68628466** | **IL26** | **1** |
| **931** | **chr 12: 68595130-68619601** | **IL26** | **1** |
| **932** | **chr 12: 68726667-68845443** | **MDM1** | **1** |
| **933** | **chr 12: 69068150-69081535** | **LOC100507250** | **1** |
| **933** | **chr 12: 69068150-69081535** | **NUP107** | **1** |
| **934** | **chr 12: 69139885-69187743** | **SLC35E3** | **1** |
| **935** | **chr 12: 70107412-70132348** | **RAB3IP** | **2** |
| **936** | **chr 12: 70132460-70352877** | **RAB3IP** | **1** |
| **937** | **chr 12: 70636773-70748773** | **CNOT2** | **1** |
| **938** | **chr 12: 71031852-71314623** | **PTPRB** | **1** |
| **938** | **chr 12: 71031852-71314623** | **PTPRR** | **1** |
| **939** | **chr 12: 71427901-71512027** | **TSPAN8** | **1** |
| **940** | **chr 12: 71518864-71835678** | **LGR5** | **1** |
| **940** | **chr 12: 71518864-71835678** | **TSPAN8** | **1** |
| **941** | **chr 12: 71833549-71980090** | **LGR5** | **1** |
| **942** | **chr 12: 72003251-72061505** | **THAP2** | **1** |
| **942** | **chr 12: 72003251-72061505** | **ZFC3H1** | **1** |
| **943** | **chr 12: 75433856-75603648** | **KCNC2** | **1** |
| **944** | **chr 12: 75956981-76377795** | **PHLDA1** | **2** |
| **945** | **chr 12: 77718420-77966055** | **none** | **2** |
| **946** | **chr 12: 78224684-78606790** | **NAV3** | **3** |
| **947** | **chr 12: 78746298-78876799** | **none** | **2** |
| **948** | **chr 12: 78842774-78934455** | **none** | **2** |
| **949** | **chr 12: 79257772-79845788** | **SYT1** | **2** |
| **950** | **chr 12: 79734984-79897176** | **SYT1** | **2** |
| **951** | **chr 12: 79968758-80084877** | **PAWR** | **1** |
| **952** | **chr 12: 80083923-80172231** | **PAWR** | **1** |
| **952** | **chr 12: 80083923-80172231** | **PPP1R12A** | **1** |
| **953** | **chr 12: 80799773-81072802** | **PTPRQ** | **2** |
| **954** | **chr 12: 81331593-81650533** | **LIN7A** | **1** |
| **955** | **chr 12: 81652044-82153332** | **PPFIA2** | **1** |
| **956** | **chr 12: 81664453-81706201** | **PPFIA2** | **1** |
| **957** | **chr 12: 82752275-82873015** | **CCDC59** | **1** |
| **958** | **chr 12: 83080658-83528649** | **TMTC2** | **3** |
| **959** | **chr 12: 85430091-85657002** | **TSPAN19** | **1** |
| **959** | **chr 12: 85430091-85657002** | **LRRIQ1** | **1** |
| **960** | **chr 12: 88427622-88443937** | **C12ORF29** | **1** |
| **960** | **chr 12: 88427622-88443937** | **CEP290** | **1** |
| **961** | **chr 12: 89981827-90103077** | **ATP2B1** | **2** |
| **961** | **chr 12: 89981827-90103077** | **LOC338758** | **2** |
| **962** | **chr 12: 92378755-92539673** | **LOC256021** | **4** |
| **963** | **chr 12: 93115280-93166231** | **PLEKHG7** | **1** |
| **964** | **chr 12: 93164412-93323107** | **EEA1** | **1** |
| **964** | **chr 12: 93164412-93323107** | **PLEKHG7** | **1** |
| **965** | **chr 12: 93397190-93609455** | **LOC643339** | **1** |
| **966** | **chr 12: 93771658-93797024** | **LOC643339** | **2** |
| **966** | **chr 12: 93771658-93797024** | **NUDT4** | **2** |
| **967** | **chr 12: 93963589-93977263** | **SOCS2-AS1** | **1** |
| **967** | **chr 12: 93963589-93977263** | **SOCS2** | **1** |
| **968** | **chr 12: 94288740-94337379** | **CRADD** | **2** |
| **969** | **chr 12: 94700224-94853764** | **PLXNC1** | **1** |
| **970** | **chr 12: 95290830-95397546** | **NDUFA12** | **1** |
| **971** | **chr 12: 95770983-95774672** | **none** | **2** |
| **972** | **chr 12: 96189122-96252617** | **SNRPF** | **1** |
| **973** | **chr 12: 96252705-96297606** | **CCDC38** | **1** |
| **973** | **chr 12: 96252705-96297606** | **SNRPF** | **1** |
| **974** | **chr 12: 96260825-96336752** | **AMDHD1** | **1** |
| **974** | **chr 12: 96260825-96336752** | **CCDC38** | **1** |
| **975** | **chr 12: 96366439-96390143** | **HAL** | **1** |
| **976** | **chr 12: 96390298-96405267** | **LTA4H** | **1** |
| **976** | **chr 12: 96390298-96405267** | **HAL** | **1** |
| **977** | **chr 12: 96394605-96437298** | **LTA4H** | **1** |
| **978** | **chr 12: 96883348-97269333** | **NEDD1** | **2** |
| **979** | **chr 12: 98987368-98995946** | **SLC25A3** | **1** |
| **979** | **chr 12: 98987368-98995946** | **SNORA53** | **1** |
| **980** | **chr 12: 99120234-100378432** | **ANKS1B** | **2** |
| **980** | **chr 12: 99120234-100378432** | **APAF1** | **2** |
| **981** | **chr 12: 99325459-99370434** | **ANKS1B** | **1** |
| **982** | **chr 12: 101111303-101522419** | **ANO4** | **1** |
| **983** | **chr 12: 101962130-102079796** | **MYBPC1** | **1** |
| **984** | **chr 12: 103631368-103889749** | **C12ORF42** | **2** |
| **985** | **chr 12: 103981050-104160505** | **STAB2** | **1** |
| **986** | **chr 12: 106457117-106533811** | **NUAK1** | **1** |
| **987** | **chr 12: 106889735-107168696** | **POLR3B** | **1** |
| **988** | **chr 12: 106976684-107156581** | **RFX4** | **1** |
| **988** | **chr 12: 106976684-107156581** | **LOC100287944** | **1** |
| **989** | **chr 12: 108523247-108644314** | **WSCD2** | **2** |
| **990** | **chr 12: 109304657-109459045** | **SVOP** | **5** |
| **991** | **chr 12: 109915206-109974507** | **UBE3B** | **1** |
| **991** | **chr 12: 109915206-109974507** | **KCTD10** | **1** |
| **992** | **chr 12: 110288747-110318293** | **GLTP** | **2** |
| **993** | **chr 12: 110338068-110421646** | **TCHP** | **1** |
| **994** | **chr 12: 110367606-110434194** | **GIT2** | **1** |
| **995** | **chr 12: 110562139-110656602** | **IFT81** | **1** |
| **996** | **chr 12: 111471827-111788358** | **CUX2** | **2** |
| **997** | **chr 12: 111890017-112037480** | **ATXN2** | **1** |
| **998** | **chr 12: 113008183-113336686** | **RPH3A** | **4** |
| **999** | **chr 12: 113658854-113736390** | **TPCN1** | **2** |
| **999** | **chr 12: 113658854-113736390** | **IQCD** | **2** |
| **1000** | **chr 12: 113736563-113797298** | **SLC24A6** | **4** |
| **1000** | **chr 12: 113736563-113797298** | **PLBD2** | **4** |
| **1001** | **chr 12: 113830249-113864106** | **SDSL** | **1** |
| **1001** | **chr 12: 113830249-113864106** | **SDS** | **1** |
| **1002** | **chr 12: 114117263-114211488** | **RBM19** | **4** |
| **1003** | **chr 12: 114791735-114846247** | **TBX5** | **1** |
| **1004** | **chr 12: 115151615-115205762** | **TBX3** | **1** |
| **1005** | **chr 12: 116395710-116715143** | **MED13L** | **2** |
| **1005** | **chr 12: 116395710-116715143** | **MIR620** | **2** |
| **1006** | **chr 12: 117153592-117175875** | **C12ORF49** | **1** |
| **1006** | **chr 12: 117153592-117175875** | **RNFT2** | **1** |
| **1007** | **chr 12: 117537285-117579896** | **TESC** | **2** |
| **1008** | **chr 12: 117645946-117889975** | **NOS1** | **5** |
| **1009** | **chr 12: 117890816-118406788** | **KSR2** | **5** |
| **1010** | **chr 12: 118587605-118810750** | **TAOK3** | **1** |
| **1011** | **chr 12: 119287158-119346735** | **none** | **2** |
| **1012** | **chr 12: 119772516-119978852** | **CCDC60** | **1** |
| **1013** | **chr 12: 119825791-120105884** | **CCDC60** | **2** |
| **1013** | **chr 12: 119825791-120105884** | **TMEM233** | **2** |
| **1014** | **chr 12: 120427672-120532298** | **CCDC64** | **1** |
| **1015** | **chr 12: 121078354-121105127** | **CABP1** | **1** |
| **1016** | **chr 12: 121746047-121837699** | **ANAPC5** | **2** |
| **1017** | **chr 12: 122277432-122301502** | **HPD** | **1** |
| **1018** | **chr 12: 122326636-122441833** | **PSMD9** | **3** |
| **1018** | **chr 12: 122326636-122441833** | **HPD** | **3** |
| **1019** | **chr 12: 122445339-122457920** | **WDR66** | **1** |
| **1019** | **chr 12: 122445339-122457920** | **BCL7A** | **1** |
| **1020** | **chr 12: 123237320-123255611** | **DENR** | **1** |
| **1021** | **chr 12: 123405497-123466196** | **ABCB9** | **1** |
| **1022** | **chr 12: 123468026-123634562** | **MIR4304** | **1** |
| **1022** | **chr 12: 123468026-123634562** | **PITPNM2** | **1** |
| **1023** | **chr 12: 124247041-124420753** | **DNAH10** | **2** |
| **1024** | **chr 12: 124456391-124800570** | **ZNF664** | **1** |
| **1024** | **chr 12: 124456391-124800570** | **CCDC92** | **1** |
| **1025** | **chr 12: 124808960-125052135** | **NCOR2** | **1** |
| **1026** | **chr 12: 125671381-126146917** | **TMEM132B** | **3** |
| **1027** | **chr 12: 127399765-127544946** | **LOC440117** | **3** |
| **1028** | **chr 12: 128751947-129192460** | **MIR3612** | **1** |
| **1028** | **chr 12: 128751947-129192460** | **TMEM132C** | **1** |
| **1029** | **chr 12: 129337971-129469509** | **GLT1D1** | **11** |
| **1030** | **chr 12: 129556269-130388211** | **TMEM132D** | **18** |
| **1031** | **chr 12: 129594173-129597839** | **TMEM132D** | **1** |
| **1032** | **chr 12: 130880681-131200826** | **RIMBP2** | **7** |
| **1033** | **chr 12: 131135915-131153778** | **none** | **4** |
| **1034** | **chr 12: 131274144-131323811** | **STX2** | **1** |
| **1035** | **chr 12: 131438451-131626014** | **GPR133** | **2** |
| **1036** | **chr 12: 131649555-131697476** | **LOC116437** | **2** |
| **1037** | **chr 12: 132568827-132613029** | **EP400NL** | **3** |
| **1038** | **chr 12: 132680923-132905935** | **GALNT9** | **1** |
| **1039** | **chr 12: 133200347-133263951** | **POLE** | **2** |
| **1039** | **chr 12: 133200347-133263951** | **PXMP2** | **2** |
| **1040** | **chr 13: 19520894-19636858** | **LINC00442** | **1** |
| **1041** | **chr 13: 19864732-19918965** | **LINC00421** | **1** |
| **1041** | **chr 13: 19864732-19918965** | **ANKRD26P3** | **1** |
| **1042** | **chr 13: 21351468-21477187** | **XPO4** | **1** |
| **1043** | **chr 13: 21714652-21723221** | **SAP18** | **1** |
| **1044** | **chr 13: 23902964-24007841** | **SACS** | **2** |
| **1045** | **chr 13: 24040709-24061603** | **LINC00327** | **1** |
| **1046** | **chr 13: 24553838-24896673** | **SPATA13** | **2** |
| **1046** | **chr 13: 24553838-24896673** | **MIR2276** | **2** |
| **1047** | **chr 13: 24982294-25171814** | **PARP4** | **1** |
| **1047** | **chr 13: 24982294-25171814** | **TPTE2P6** | **1** |
| **1048** | **chr 13: 25498814-25542625** | **TPTE2P1** | **1** |
| **1049** | **chr 13: 25755578-25763898** | **AMER2** | **2** |
| **1050** | **chr 13: 25946208-26599989** | **ATP8A2** | **3** |
| **1051** | **chr 13: 26828275-26979375** | **CDK8** | **1** |
| **1052** | **chr 13: 27131839-27263085** | **WASF3** | **1** |
| **1053** | **chr 13: 28407580-28495541** | **PDX1** | **2** |
| **1054** | **chr 13: 28577410-28674729** | **FLT3** | **1** |
| **1055** | **chr 13: 28874488-29069265** | **FLT1** | **1** |
| **1056** | **chr 13: 31032883-31191734** | **HMGB1** | **1** |
| **1056** | **chr 13: 31032883-31191734** | **USPL1** | **1** |
| **1057** | **chr 13: 32420977-32527609** | **EEF1DP3** | **2** |
| **1058** | **chr 13: 32605436-32870794** | **FRY** | **1** |
| **1059** | **chr 13: 32877836-32889481** | **BRCA2** | **1** |
| **1059** | **chr 13: 32877836-32889481** | **ZAR1L** | **1** |
| **1060** | **chr 13: 32889610-32973805** | **BRCA2** | **1** |
| **1061** | **chr 13: 35009586-35245319** | **LINC00457** | **1** |
| **1062** | **chr 13: 37418967-37494902** | **SMAD9** | **1** |
| **1063** | **chr 13: 39261265-39460074** | **FREM2** | **1** |
| **1064** | **chr 13: 39540061-39565203** | **STOML3** | **1** |
| **1065** | **chr 13: 39612442-39624246** | **NHLRC3** | **1** |
| **1065** | **chr 13: 39612442-39624246** | **PROSER1** | **1** |
| **1066** | **chr 13: 39917028-40177665** | **LHFP** | **1** |
| **1067** | **chr 13: 41303431-41345309** | **MRPS31** | **1** |
| **1068** | **chr 13: 41396431-41495910** | **TPTE2P5** | **1** |
| **1069** | **chr 13: 42140972-42535256** | **MIR5006** | **1** |
| **1069** | **chr 13: 42140972-42535256** | **VWA8** | **1** |
| **1070** | **chr 13: 42614175-42830714** | **DGKH** | **1** |
| **1071** | **chr 13: 43787653-44361044** | **ENOX1** | **2** |
| **1072** | **chr 13: 44482849-44604597** | **LINC00284** | **2** |
| **1073** | **chr 13: 45007654-45151283** | **TSC22D1** | **1** |
| **1074** | **chr 13: 45513383-45657260** | **KIAA1704** | **1** |
| **1074** | **chr 13: 45513383-45657260** | **NUFIP1** | **1** |
| **1075** | **chr 13: 46108656-46189874** | **COG3** | **1** |
| **1075** | **chr 13: 46108656-46189874** | **FAM194B** | **1** |
| **1076** | **chr 13: 46354404-46425871** | **SIAH3** | **1** |
| **1077** | **chr 13: 46916138-47012325** | **KIAA0226L** | **1** |
| **1078** | **chr 13: 49882785-50020554** | **CAB39L** | **1** |
| **1079** | **chr 13: 50656306-51297372** | **DLEU2** | **3** |
| **1080** | **chr 13: 51095068-51101585** | **none** | **1** |
| **1081** | **chr 13: 51568646-51654998** | **GUCY1B2** | **1** |
| **1082** | **chr 13: 52158643-52336171** | **WDFY2** | **1** |
| **1083** | **chr 13: 61983817-62002220** | **PCDH20** | **1** |
| **1084** | **chr 13: 66876966-67804468** | **PCDH9** | **3** |
| **1084** | **chr 13: 66876966-67804468** | **PCDH9-AS2** | **3** |
| **1085** | **chr 13: 67399300-67489163** | **PCDH9** | **1** |
| **1085** | **chr 13: 67399300-67489163** | **PCDH9-AS2** | **1** |
| **1086** | **chr 13: 72012097-72441330** | **DACH1** | **1** |
| **1087** | **chr 13: 73629113-73651676** | **KLF5** | **1** |
| **1088** | **chr 13: 74260225-74708394** | **KLF12** | **1** |
| **1089** | **chr 13: 74993309-75009296** | **LINC00381** | **1** |
| **1090** | **chr 13: 75858807-76056250** | **TBC1D4** | **1** |
| **1091** | **chr 13: 76123618-76434004** | **UCHL3** | **2** |
| **1092** | **chr 13: 78402356-78408085** | **none** | **1** |
| **1093** | **chr 13: 78493823-79191463** | **EDNRB** | **5** |
| **1094** | **chr 13: 92050928-93519490** | **GPC5** | **4** |
| **1094** | **chr 13: 92050928-93519490** | **GPC5-AS1** | **4** |
| **1095** | **chr 13: 93879094-95059655** | **GPC6-AS2** | **6** |
| **1095** | **chr 13: 93879094-95059655** | **GPC6** | **6** |
| **1096** | **chr 13: 95672082-95953687** | **ABCC4** | **1** |
| **1097** | **chr 13: 96230456-96296957** | **CLDN10** | **2** |
| **1098** | **chr 13: 96329392-96447243** | **DNAJC3** | **1** |
| **1099** | **chr 13: 96743092-97485671** | **HS6ST3** | **5** |
| **1100** | **chr 13: 97873687-98046374** | **MBNL2** | **1** |
| **1101** | **chr 13: 98605911-98676551** | **IPO5** | **1** |
| **1102** | **chr 13: 98794815-99102027** | **FARP1** | **3** |
| **1103** | **chr 13: 99445740-99738879** | **DOCK9** | **2** |
| **1104** | **chr 13: 99853027-100038688** | **UBAC2-AS1** | **1** |
| **1105** | **chr 13: 100153670-100216260** | **TM9SF2** | **1** |
| **1106** | **chr 13: 100258918-100549387** | **CLYBL** | **1** |
| **1106** | **chr 13: 100258918-100549387** | **MIR4306** | **1** |
| **1107** | **chr 13: 100741268-101182686** | **PCCA-AS1** | **2** |
| **1107** | **chr 13: 100741268-101182686** | **PCCA** | **2** |
| **1108** | **chr 13: 101706129-102068843** | **NALCN-AS1** | **1** |
| **1108** | **chr 13: 101706129-102068843** | **NALCN** | **1** |
| **1109** | **chr 13: 103451398-103528345** | **KDELC1** | **1** |
| **1109** | **chr 13: 103451398-103528345** | **BIVM** | **1** |
| **1110** | **chr 13: 107820882-108519083** | **FAM155A** | **4** |
| **1111** | **chr 13: 108903587-108960832** | **TNFSF13B** | **1** |
| **1112** | **chr 13: 110958158-111165374** | **COL4A2** | **1** |
| **1112** | **chr 13: 110958158-111165374** | **COL4A1** | **1** |
| **1113** | **chr 13: 113344642-113541482** | **ATP11A** | **2** |
| **1114** | **chr 13: 114747193-114898086** | **RASA3** | **1** |
| **1115** | **chr 14: 20078032-20148263** | **OR11H2** | **1** |
| **1116** | **chr 14: 20724716-20774153** | **TTC5** | **1** |
| **1117** | **chr 14: 20833825-20881588** | **TEP1** | **1** |
| **1118** | **chr 14: 20914569-20923264** | **APEX1** | **1** |
| **1118** | **chr 14: 20914569-20923264** | **OSGEP** | **1** |
| **1119** | **chr 14: 21338436-21341751** | **RNASE3** | **5** |
| **1120** | **chr 14: 21484921-21539031** | **NDRG2** | **1** |
| **1121** | **chr 14: 21492267-21504435** | **NDRG2** | **1** |
| **1122** | **chr 14: 21538428-21558399** | **ZNF219** | **1** |
| **1122** | **chr 14: 21538428-21558399** | **ARHGEF40** | **1** |
| **1123** | **chr 14: 21576201-21584862** | **TMEM253** | **1** |
| **1123** | **chr 14: 21576201-21584862** | **OR5AU1** | **1** |
| **1124** | **chr 14: 21989231-22005350** | **SALL2** | **1** |
| **1125** | **chr 14: 22615948-22616587** | **none** | **1** |
| **1126** | **chr 14: 22849082-22951948** | **none** | **1** |
| **1127** | **chr 14: 23171819-23235771** | **OXA1L** | **1** |
| **1128** | **chr 14: 23594503-23652883** | **SLC7A8** | **1** |
| **1129** | **chr 14: 23851198-23877486** | **MIR208A** | **1** |
| **1129** | **chr 14: 23851198-23877486** | **MYH6** | **1** |
| **1130** | **chr 14: 24028773-24048009** | **THTPA** | **1** |
| **1131** | **chr 14: 24615891-24636611** | **PSME2** | **1** |
| **1131** | **chr 14: 24615891-24636611** | **RNF31** | **1** |
| **1132** | **chr 14: 25278861-25519503** | **STXBP6** | **1** |
| **1133** | **chr 14: 27305933-27629626** | **MIR4307** | **1** |
| **1134** | **chr 14: 29269289-29299410** | **C14ORF23** | **1** |
| **1135** | **chr 14: 29299449-29497969** | **C14ORF23** | **2** |
| **1136** | **chr 14: 29734030-29850501** | **MIR548AI** | **1** |
| **1137** | **chr 14: 30045686-30766249** | **PRKD1** | **3** |
| **1137** | **chr 14: 30045686-30766249** | **MIR548AI** | **3** |
| **1138** | **chr 14: 31717833-31771945** | **HEATR5A** | **1** |
| **1139** | **chr 14: 32476107-32487891** | **none** | **2** |
| **1140** | **chr 14: 32798478-33300567** | **AKAP6** | **1** |
| **1141** | **chr 14: 33404138-34273382** | **NPAS3** | **4** |
| **1142** | **chr 14: 34393436-34931980** | **EGLN3** | **7** |
| **1142** | **chr 14: 34393436-34931980** | **SPTSSA** | **7** |
| **1143** | **chr 14: 35591051-35786699** | **PPP2R3C** | **1** |
| **1143** | **chr 14: 35591051-35786699** | **KIAA0391** | **1** |
| **1144** | **chr 14: 36295523-36532949** | **BRMS1L** | **1** |
| **1145** | **chr 14: 36539632-36634494** | **PTCSC3** | **2** |
| **1145** | **chr 14: 36539632-36634494** | **LINC00609** | **2** |
| **1146** | **chr 14: 36605313-36645674** | **PTCSC3** | **1** |
| **1146** | **chr 14: 36605313-36645674** | **LINC00609** | **1** |
| **1147** | **chr 14: 37667117-38021566** | **MIPOL1** | **2** |
| **1148** | **chr 14: 38065051-38510647** | **FOXA1** | **4** |
| **1149** | **chr 14: 39699434-39856156** | **CTAGE5** | **1** |
| **1149** | **chr 14: 39699434-39856156** | **MIA2** | **1** |
| **1150** | **chr 14: 42076772-42373752** | **LRFN5** | **1** |
| **1151** | **chr 14: 42832185-43172858** | **none** | **2** |
| **1152** | **chr 14: 45232359-45252032** | **none** | **1** |
| **1153** | **chr 14: 46410145-46971026** | **none** | **3** |
| **1154** | **chr 14: 47308825-48144157** | **MDGA2** | **1** |
| **1155** | **chr 14: 49524430-49544223** | **none** | **1** |
| **1156** | **chr 14: 51186480-51297839** | **NIN** | **1** |
| **1157** | **chr 14: 51314839-51332377** | **NIN** | **1** |
| **1157** | **chr 14: 51314839-51332377** | **ABHD12B** | **1** |
| **1158** | **chr 14: 51441979-51562779** | **TRIM9** | **1** |
| **1159** | **chr 14: 51771133-51795104** | **LINC00640** | **1** |
| **1159** | **chr 14: 51771133-51795104** | **TMX1** | **1** |
| **1160** | **chr 14: 52471520-52535712** | **NID2** | **1** |
| **1161** | **chr 14: 55033814-55260033** | **SAMD4A** | **2** |
| **1162** | **chr 14: 55493947-55516206** | **SOCS4** | **1** |
| **1162** | **chr 14: 55493947-55516206** | **WDHD1** | **1** |
| **1163** | **chr 14: 55833109-55878576** | **ATG14** | **2** |
| **1164** | **chr 14: 56025789-56168244** | **KTN1-AS1** | **1** |
| **1164** | **chr 14: 56025789-56168244** | **KTN1** | **1** |
| **1165** | **chr 14: 56777597-56811719** | **PELI2** | **2** |
| **1166** | **chr 14: 57115682-57197253** | **TMEM260** | **1** |
| **1167** | **chr 14: 57267424-57277197** | **OTX2** | **1** |
| **1168** | **chr 14: 57279900-57618895** | **OTX2-AS1** | **1** |
| **1169** | **chr 14: 58030639-58448912** | **SLC35F4** | **4** |
| **1170** | **chr 14: 58862633-58893843** | **TOMM20L** | **1** |
| **1170** | **chr 14: 58862633-58893843** | **TIMM9** | **1** |
| **1171** | **chr 14: 58875211-58894332** | **TOMM20L** | **1** |
| **1171** | **chr 14: 58875211-58894332** | **TIMM9** | **1** |
| **1172** | **chr 14: 58894102-59015216** | **KIAA0586** | **1** |
| **1172** | **chr 14: 58894102-59015216** | **TIMM9** | **1** |
| **1173** | **chr 14: 59294951-59484046** | **none** | **1** |
| **1174** | **chr 14: 59895739-60043549** | **GPR135** | **1** |
| **1174** | **chr 14: 59895739-60043549** | **L3HYPDH** | **1** |
| **1175** | **chr 14: 60062693-60337684** | **RTN1** | **1** |
| **1176** | **chr 14: 60863186-60982261** | **SIX6** | **1** |
| **1176** | **chr 14: 60863186-60982261** | **C14ORF39** | **1** |
| **1177** | **chr 14: 60981836-61021634** | **SIX6** | **1** |
| **1178** | **chr 14: 61176245-61191066** | **SIX4** | **2** |
| **1179** | **chr 14: 61201459-61550451** | **MNAT1** | **2** |
| **1179** | **chr 14: 61201459-61550451** | **TRMT5** | **2** |
| **1180** | **chr 14: 61654276-62125414** | **PRKCH** | **1** |
| **1180** | **chr 14: 61654276-62125414** | **TMEM30B** | **1** |
| **1181** | **chr 14: 62278691-62437037** | **SNAPC1** | **1** |
| **1181** | **chr 14: 62278691-62437037** | **SYT16** | **1** |
| **1182** | **chr 14: 63173286-63568755** | **KCNH5** | **2** |
| **1183** | **chr 14: 63838074-64010092** | **PPP2R5E** | **1** |
| **1184** | **chr 14: 64319682-64693165** | **SYNE2** | **1** |
| **1185** | **chr 14: 64550949-64805317** | **SYNE2** | **1** |
| **1186** | **chr 14: 65170819-65213610** | **PLEKHG3** | **2** |
| **1186** | **chr 14: 65170819-65213610** | **SPTB** | **2** |
| **1187** | **chr 14: 65213001-65346601** | **SPTB** | **1** |
| **1188** | **chr 14: 65381078-65529368** | **CHURC1-FNTB** | **1** |
| **1188** | **chr 14: 65381078-65529368** | **CHURC1** | **1** |
| **1189** | **chr 14: 66679527-66976112** | **LINC00238** | **1** |
| **1189** | **chr 14: 66679527-66976112** | **GPHN** | **1** |
| **1190** | **chr 14: 66974124-67648520** | **GPHN** | **2** |
| **1191** | **chr 14: 67707825-67802536** | **MPP5** | **1** |
| **1192** | **chr 14: 67913800-68000456** | **PLEKHH1** | **2** |
| **1192** | **chr 14: 67913800-68000456** | **TMEM229B** | **2** |
| **1193** | **chr 14: 68000017-68056329** | **PIGH** | **1** |
| **1193** | **chr 14: 68000017-68056329** | **PLEKHH1** | **1** |
| **1194** | **chr 14: 68086514-68118437** | **ARG2** | **1** |
| **1194** | **chr 14: 68086514-68118437** | **VTI1B** | **1** |
| **1195** | **chr 14: 68113791-68162531** | **ARG2** | **1** |
| **1195** | **chr 14: 68113791-68162531** | **VTI1B** | **1** |
| **1196** | **chr 14: 68286495-69196935** | **RAD51B** | **2** |
| **1197** | **chr 14: 70510933-70655787** | **SLC8A3** | **1** |
| **1198** | **chr 14: 71165414-71178870** | **MAP3K9** | **1** |
| **1198** | **chr 14: 71165414-71178870** | **TTC9** | **1** |
| **1199** | **chr 14: 71787165-72207946** | **SNORD56B** | **1** |
| **1199** | **chr 14: 71787165-72207946** | **LOC145474** | **1** |
| **1200** | **chr 14: 72315322-72375147** | **RGS6** | **1** |
| **1201** | **chr 14: 72399155-73030654** | **RGS6** | **1** |
| **1202** | **chr 14: 73086003-73360809** | **DPF3** | **4** |
| **1203** | **chr 14: 73436158-73493920** | **ZFYVE1** | **1** |
| **1204** | **chr 14: 73603125-73690399** | **PSEN1** | **1** |
| **1205** | **chr 14: 73704204-73741348** | **PAPLN** | **1** |
| **1206** | **chr 14: 74181824-74256988** | **PNMA1** | **2** |
| **1206** | **chr 14: 74181824-74256988** | **ELMSAN1** | **2** |
| **1207** | **chr 14: 74551498-74667936** | **ALDH6A1** | **1** |
| **1207** | **chr 14: 74551498-74667936** | **LIN52** | **1** |
| **1208** | **chr 14: 74964872-75079306** | **LTBP2** | **1** |
| **1209** | **chr 14: 75598172-75643334** | **TMED10** | **1** |
| **1210** | **chr 14: 76044959-76550928** | **FLVCR2** | **2** |
| **1211** | **chr 14: 76424441-76449334** | **TGFB3** | **1** |
| **1212** | **chr 14: 76776956-76968178** | **ESRRB** | **1** |
| **1213** | **chr 14: 77388182-77390992** | **none** | **1** |
| **1214** | **chr 14: 77787226-77797940** | **POMT2** | **1** |
| **1214** | **chr 14: 77787226-77797940** | **GSTZ1** | **1** |
| **1215** | **chr 14: 78266425-78401355** | **ADCK1** | **1** |
| **1216** | **chr 14: 78708733-80330762** | **NRXN3** | **4** |
| **1217** | **chr 14: 80128008-80257606** | **NRXN3** | **3** |
| **1218** | **chr 14: 80943329-81425861** | **CEP128** | **1** |
| **1218** | **chr 14: 80943329-81425861** | **TSHR** | **1** |
| **1219** | **chr 14: 81421332-81612646** | **TSHR** | **2** |
| **1220** | **chr 14: 82207345-82496693** | **none** | **3** |
| **1221** | **chr 14: 87819413-88039366** | **none** | **2** |
| **1222** | **chr 14: 88176762-88460009** | **GALC** | **3** |
| **1223** | **chr 14: 88649112-88793251** | **KCNK10** | **1** |
| **1224** | **chr 14: 89591214-90421121** | **FOXN3** | **7** |
| **1225** | **chr 14: 90742579-90798481** | **NRDE2** | **1** |
| **1226** | **chr 14: 91006931-91282823** | **TTC7B** | **2** |
| **1227** | **chr 14: 91526676-91691976** | **RPS6KA5** | **2** |
| **1228** | **chr 14: 91698875-91720269** | **GPR68** | **2** |
| **1229** | **chr 14: 91709102-91717426** | **GPR68** | **2** |
| **1230** | **chr 14: 91737666-91884188** | **CCDC88C** | **1** |
| **1231** | **chr 14: 92432334-92507240** | **TRIP11** | **1** |
| **1232** | **chr 14: 92524895-92572965** | **ATXN3** | **2** |
| **1233** | **chr 14: 93799564-94174222** | **BTBD7** | **2** |
| **1234** | **chr 14: 94371075-94393412** | **FAM181A-AS1** | **2** |
| **1234** | **chr 14: 94371075-94393412** | **FAM181A** | **2** |
| **1235** | **chr 14: 94385239-94395954** | **FAM181A-AS1** | **2** |
| **1235** | **chr 14: 94385239-94395954** | **FAM181A** | **2** |
| **1236** | **chr 14: 94594115-94596590** | **IFI27L2** | **1** |
| **1237** | **chr 14: 95648276-95786243** | **CLMN** | **9** |
| **1238** | **chr 14: 96087250-96109622** | **TCL6** | **1** |
| **1239** | **chr 14: 97207527-97256950** | **VRK1** | **1** |
| **1240** | **chr 14: 97263640-97411731** | **VRK1** | **1** |
| **1241** | **chr 14: 99635623-99737861** | **BCL11B** | **1** |
| **1242** | **chr 14: 99864082-99947216** | **SETD3** | **1** |
| **1243** | **chr 14: 100204029-100408397** | **EML1** | **1** |
| **1244** | **chr 14: 100437785-100610573** | **EVL** | **1** |
| **1245** | **chr 14: 100673743-100704892** | **YY1** | **3** |
| **1246** | **chr 14: 100746295-100772884** | **SLC25A29** | **1** |
| **1247** | **chr 14: 102018557-102026768** | **MIR1247** | **1** |
| **1247** | **chr 14: 102018557-102026768** | **DIO3OS** | **1** |
| **1248** | **chr 14: 102690836-102771537** | **MOK** | **1** |
| **1249** | **chr 14: 103606011-103608296** | **LINC00605** | **1** |
| **1249** | **chr 14: 103606011-103608296** | **TNFAIP2** | **1** |
| **1250** | **chr 14: 103851728-103970168** | **MARK3** | **1** |
| **1251** | **chr 14: 104028232-104167888** | **BAG5** | **1** |
| **1252** | **chr 14: 104394798-104519004** | **TDRD9** | **2** |
| **1252** | **chr 14: 104394798-104519004** | **RD3L** | **2** |
| **1253** | **chr 14: 104689920-104754406** | **KIF26A** | **1** |
| **1254** | **chr 14: 105119884-105122124** | **TMEM179** | **1** |
| **1254** | **chr 14: 105119884-105122124** | **MIR4710** | **1** |
| **1255** | **chr 14: 105952653-105965912** | **CRIP1** | **1** |
| **1255** | **chr 14: 105952653-105965912** | **C14ORF80** | **1** |
| **1256** | **chr 15: 22892004-23006016** | **CYFIP1** | **2** |
| **1257** | **chr 15: 24686273-24693114** | **none** | **1** |
| **1258** | **chr 15: 25068793-25664609** | **SNRPN** | **1** |
| **1259** | **chr 15: 25729977-25823938** | **UBE3A** | **2** |
| **1260** | **chr 15: 26147506-26298267** | **LOC100128714** | **2** |
| **1261** | **chr 15: 26360959-26378184** | **LOC503519** | **1** |
| **1262** | **chr 15: 26788692-27184686** | **GABRB3** | **3** |
| **1263** | **chr 15: 27111509-27194354** | **GABRA5** | **1** |
| **1264** | **chr 15: 27216428-27778373** | **GABRG3** | **12** |
| **1265** | **chr 15: 28000020-28344504** | **OCA2** | **1** |
| **1266** | **chr 15: 29129628-29410518** | **APBA2** | **5** |
| **1267** | **chr 15: 29412456-29862927** | **FAM189A1** | **3** |
| **1267** | **chr 15: 29412456-29862927** | **NDNL2** | **3** |
| **1268** | **chr 15: 29991570-30261068** | **TJP1** | **1** |
| **1269** | **chr 15: 30916696-31065196** | **LOC100288637** | **1** |
| **1269** | **chr 15: 30916696-31065196** | **ARHGAP11B** | **1** |
| **1270** | **chr 15: 31293263-31453476** | **TRPM1** | **1** |
| **1271** | **chr 15: 31619057-31727868** | **KLF13** | **1** |
| **1272** | **chr 15: 31775328-32162992** | **OTUD7A** | **2** |
| **1273** | **chr 15: 32322690-32464722** | **CHRNA7** | **1** |
| **1274** | **chr 15: 33057746-33486897** | **FMN1** | **2** |
| **1275** | **chr 15: 33603162-34158303** | **RYR3** | **1** |
| **1276** | **chr 15: 34158427-34331377** | **CHRM5** | **1** |
| **1276** | **chr 15: 34158427-34331377** | **AVEN** | **1** |
| **1277** | **chr 15: 34525459-34630261** | **SLC12A6** | **1** |
| **1278** | **chr 15: 35509545-35838394** | **ANP32AP1** | **2** |
| **1278** | **chr 15: 35509545-35838394** | **ATPBD4** | **2** |
| **1279** | **chr 15: 35838395-36151202** | **ATPBD4** | **2** |
| **1280** | **chr 15: 36871811-37102449** | **C15ORF41** | **1** |
| **1281** | **chr 15: 36933386-36961298** | **C15ORF41** | **1** |
| **1282** | **chr 15: 37657227-37783009** | **none** | **1** |
| **1283** | **chr 15: 39157522-39719396** | **C15ORF54** | **3** |
| **1284** | **chr 15: 40331511-40359491** | **LOC100131089** | **1** |
| **1284** | **chr 15: 40331511-40359491** | **SRP14** | **1** |
| **1285** | **chr 15: 40780239-40850183** | **C15ORF57** | **2** |
| **1285** | **chr 15: 40780239-40850183** | **MRPL42P5** | **2** |
| **1286** | **chr 15: 40886217-40956540** | **CASC5** | **1** |
| **1287** | **chr 15: 40978921-40987305** | **LOC100505648** | **1** |
| **1287** | **chr 15: 40978921-40987305** | **RAD51** | **1** |
| **1288** | **chr 15: 41128005-41136585** | **SPINT1** | **1** |
| **1289** | **chr 15: 41474922-41522941** | **CHP1** | **2** |
| **1289** | **chr 15: 41474922-41522941** | **EXD1** | **2** |
| **1290** | **chr 15: 42190949-42264776** | **EHD4** | **1** |
| **1291** | **chr 15: 42273779-42343388** | **PLA2G4E** | **3** |
| **1292** | **chr 15: 42433331-42448839** | **PLA2G4F** | **1** |
| **1293** | **chr 15: 42450898-42500514** | **VPS39** | **1** |
| **1293** | **chr 15: 42450898-42500514** | **MIR627** | **1** |
| **1294** | **chr 15: 43015756-43029324** | **CDAN1** | **1** |
| **1295** | **chr 15: 43030931-43213007** | **TTBK2** | **2** |
| **1296** | **chr 15: 43031315-43035400** | **TTBK2** | **1** |
| **1296** | **chr 15: 43031315-43035400** | **CDAN1** | **1** |
| **1297** | **chr 15: 43235094-43398311** | **UBR1** | **1** |
| **1298** | **chr 15: 43699406-43802926** | **TP53BP1** | **1** |
| **1298** | **chr 15: 43699406-43802926** | **RNU6-28** | **1** |
| **1299** | **chr 15: 45248899-45271427** | **C15ORF43** | **1** |
| **1300** | **chr 15: 45997275-46223267** | **SQRDL** | **4** |
| **1301** | **chr 15: 47476297-48066420** | **SEMA6D** | **3** |
| **1302** | **chr 15: 48176506-48341309** | **none** | **1** |
| **1303** | **chr 15: 48483735-48596275** | **SLC12A1** | **1** |
| **1303** | **chr 15: 48483735-48596275** | **CTXN2** | **1** |
| **1304** | **chr 15: 48700502-48938046** | **FBN1** | **2** |
| **1305** | **chr 15: 50150434-50475014** | **ATP8B4** | **1** |
| **1306** | **chr 15: 50844669-50979012** | **TRPM7** | **1** |
| **1307** | **chr 15: 52043757-52108565** | **LYSMD2** | **1** |
| **1307** | **chr 15: 52043757-52108565** | **TMOD2** | **1** |
| **1308** | **chr 15: 52413116-52483566** | **GNB5** | **1** |
| **1309** | **chr 15: 52472289-52498071** | **GNB5** | **1** |
| **1310** | **chr 15: 53408561-53421895** | **none** | **2** |
| **1311** | **chr 15: 53805937-54055075** | **WDR72** | **1** |
| **1312** | **chr 15: 54305100-54920806** | **UNC13C** | **3** |
| **1313** | **chr 15: 55495163-55611359** | **RAB27A** | **3** |
| **1314** | **chr 15: 56536206-56738195** | **RFX7** | **3** |
| **1314** | **chr 15: 56536206-56738195** | **TEX9** | **3** |
| **1315** | **chr 15: 56922378-57210769** | **ZNF280D** | **2** |
| **1316** | **chr 15: 57210820-57591479** | **TCF12** | **3** |
| **1316** | **chr 15: 57210820-57591479** | **LOC145783** | **3** |
| **1317** | **chr 15: 58245621-58790934** | **ALDH1A2** | **1** |
| **1318** | **chr 15: 59664891-59815748** | **MYO1E** | **1** |
| **1319** | **chr 15: 60639332-60695082** | **ANXA2** | **2** |
| **1320** | **chr 15: 60780482-61521518** | **RORA** | **4** |
| **1321** | **chr 15: 61592524-61927648** | **none** | **3** |
| **1322** | **chr 15: 63613576-63674360** | **CA12** | **1** |
| **1323** | **chr 15: 63748492-63761550** | **USP3** | **1** |
| **1324** | **chr 15: 64457715-64679886** | **KIAA0101** | **2** |
| **1324** | **chr 15: 64457715-64679886** | **CSNK1G1** | **2** |
| **1325** | **chr 15: 64752940-64978264** | **ZNF609** | **3** |
| **1326** | **chr 15: 65032090-65067786** | **MIR1272** | **2** |
| **1326** | **chr 15: 65032090-65067786** | **RBPMS2** | **2** |
| **1327** | **chr 15: 65345678-65369028** | **SLC51B** | **1** |
| **1327** | **chr 15: 65345678-65369028** | **RASL12** | **1** |
| **1328** | **chr 15: 66187416-66546085** | **MIR4311** | **2** |
| **1328** | **chr 15: 66187416-66546085** | **MEGF11** | **2** |
| **1329** | **chr 15: 66994565-67074338** | **SMAD6** | **2** |
| **1330** | **chr 15: 67547137-67794598** | **AAGAB** | **1** |
| **1331** | **chr 15: 68483042-68549549** | **CLN6** | **1** |
| **1331** | **chr 15: 68483042-68549549** | **CALML4** | **1** |
| **1332** | **chr 15: 68594049-68724501** | **ITGA11** | **1** |
| **1333** | **chr 15: 68871307-69020145** | **CORO2B** | **1** |
| **1334** | **chr 15: 69110559-69355083** | **ANP32A** | **1** |
| **1335** | **chr 15: 69365265-69392275** | **MIR548H4** | **1** |
| **1336** | **chr 15: 69591285-69700119** | **PAQR5** | **1** |
| **1337** | **chr 15: 69884467-69988089** | **LOC145837** | **1** |
| **1338** | **chr 15: 71145577-71342414** | **LARP6** | **2** |
| **1339** | **chr 15: 71389290-72075722** | **THSD4** | **1** |
| **1339** | **chr 15: 71389290-72075722** | **CT62** | **1** |
| **1340** | **chr 15: 72084976-72110600** | **NR2E3** | **1** |
| **1341** | **chr 15: 72114631-72410918** | **MYO9A** | **1** |
| **1341** | **chr 15: 72114631-72410918** | **SENP8** | **1** |
| **1342** | **chr 15: 72700118-72767509** | **TMEM202** | **1** |
| **1342** | **chr 15: 72700118-72767509** | **ARIH1** | **1** |
| **1343** | **chr 15: 72766666-72879692** | **MIR630** | **2** |
| **1343** | **chr 15: 72766666-72879692** | **ARIH1** | **2** |
| **1344** | **chr 15: 74032140-74045088** | **C15ORF59** | **1** |
| **1345** | **chr 15: 74218329-74244478** | **LOXL1-AS1** | **2** |
| **1346** | **chr 15: 74495045-74628813** | **STRA6** | **1** |
| **1347** | **chr 15: 75136070-75165706** | **ULK3** | **1** |
| **1347** | **chr 15: 75136070-75165706** | **SCAMP2** | **1** |
| **1348** | **chr 15: 75315895-75409803** | **PPCDC** | **2** |
| **1349** | **chr 15: 75759461-75918810** | **PTPN9** | **1** |
| **1349** | **chr 15: 75759461-75918810** | **SNUPN** | **1** |
| **1350** | **chr 15: 75931425-75941047** | **IMP3** | **1** |
| **1350** | **chr 15: 75931425-75941047** | **SNX33** | **1** |
| **1351** | **chr 15: 76135621-76193419** | **UBE2Q2** | **1** |
| **1352** | **chr 15: 76352177-76521462** | **ETFA** | **2** |
| **1352** | **chr 15: 76352177-76521462** | **C15ORF27** | **2** |
| **1353** | **chr 15: 77285699-77329673** | **PSTPIP1** | **1** |
| **1354** | **chr 15: 77336358-77376326** | **TSPAN3** | **1** |
| **1355** | **chr 15: 77400470-77712486** | **LINC00597** | **2** |
| **1355** | **chr 15: 77400470-77712486** | **PEAK1** | **2** |
| **1356** | **chr 15: 77905368-78113242** | **LOC253044** | **1** |
| **1356** | **chr 15: 77905368-78113242** | **LINGO1** | **1** |
| **1357** | **chr 15: 78542843-78556498** | **DNAJA4** | **1** |
| **1358** | **chr 15: 78916460-79020096** | **CHRNB4** | **1** |
| **1359** | **chr 15: 78952985-79027837** | **LOC646938** | **1** |
| **1359** | **chr 15: 78952985-79027837** | **CHRNB4** | **1** |
| **1360** | **chr 15: 80696691-80890278** | **ARNT2** | **1** |
| **1361** | **chr 15: 81071683-81244117** | **MIR549** | **1** |
| **1361** | **chr 15: 81071683-81244117** | **KIAA1199** | **1** |
| **1362** | **chr 15: 81299373-81441516** | **C15ORF26** | **1** |
| **1363** | **chr 15: 81702857-81711358** | **TMC3** | **2** |
| **1364** | **chr 15: 82647359-82731338** | **UBE2Q2P3** | **2** |
| **1364** | **chr 15: 82647359-82731338** | **UBE2Q2P2** | **2** |
| **1365** | **chr 15: 82664458-82748784** | **UBE2Q2P3** | **2** |
| **1365** | **chr 15: 82664458-82748784** | **UBE2Q2P2** | **2** |
| **1366** | **chr 15: 83023846-83108085** | **UBE2Q2P3** | **2** |
| **1366** | **chr 15: 83023846-83108085** | **UBE2Q2P2** | **2** |
| **1367** | **chr 15: 83040999-83125316** | **UBE2Q2P3** | **2** |
| **1367** | **chr 15: 83040999-83125316** | **UBE2Q2P2** | **2** |
| **1368** | **chr 15: 83509837-83654661** | **FAM103A1** | **1** |
| **1368** | **chr 15: 83509837-83654661** | **HOMER2** | **1** |
| **1369** | **chr 15: 85051115-85114447** | **GOLGA6L5** | **2** |
| **1369** | **chr 15: 85051115-85114447** | **UBE2Q2P1** | **2** |
| **1370** | **chr 15: 85144216-85171027** | **ZSCAN2** | **1** |
| **1371** | **chr 15: 85427884-85518876** | **SLC28A1** | **2** |
| **1372** | **chr 15: 86626575-86659948** | **AGBL1** | **2** |
| **1373** | **chr 15: 86685226-87572283** | **LOC727915** | **6** |
| **1373** | **chr 15: 86685226-87572283** | **AGBL1** | **6** |
| **1374** | **chr 15: 87975288-88247083** | **LINC00052** | **1** |
| **1375** | **chr 15: 88418229-88799999** | **NTRK3** | **1** |
| **1376** | **chr 15: 89420518-89438857** | **HAPLN3** | **1** |
| **1377** | **chr 15: 89904809-89939471** | **LOC254559** | **1** |
| **1377** | **chr 15: 89904809-89939471** | **MIR9-3** | **1** |
| **1378** | **chr 15: 91163238-91260372** | **BLM** | **1** |
| **1378** | **chr 15: 91163238-91260372** | **CRTC3** | **1** |
| **1379** | **chr 15: 91541645-91565833** | **VPS33B** | **1** |
| **1380** | **chr 15: 91951937-92148441** | **none** | **1** |
| **1381** | **chr 15: 92396924-92715665** | **SLCO3A1** | **1** |
| **1382** | **chr 15: 93160672-93353114** | **ASB9P1** | **1** |
| **1382** | **chr 15: 93160672-93353114** | **FAM174B** | **1** |
| **1383** | **chr 15: 93855785-94112712** | **none** | **2** |
| **1384** | **chr 15: 94133095-94304028** | **none** | **1** |
| **1385** | **chr 15: 94261770-94651167** | **none** | **2** |
| **1386** | **chr 15: 96897465-96946964** | **NR2F2** | **1** |
| **1387** | **chr 15: 97839110-97975324** | **none** | **1** |
| **1388** | **chr 15: 97913600-97971182** | **none** | **1** |
| **1389** | **chr 15: 98058516-98065041** | **none** | **1** |
| **1390** | **chr 15: 99192199-99507759** | **IGF1R** | **2** |
| **1390** | **chr 15: 99192199-99507759** | **MIR4714** | **2** |
| **1391** | **chr 15: 99638419-99675798** | **SYNM** | **1** |
| **1392** | **chr 15: 99669036-99672011** | **SYNM** | **1** |
| **1393** | **chr 15: 100511793-100882210** | **ADAMTS17** | **2** |
| **1394** | **chr 15: 101256453-101368702** | **none** | **2** |
| **1395** | **chr 15: 101840817-102065405** | **LOC100507472** | **1** |
| **1395** | **chr 15: 101840817-102065405** | **PCSK6** | **1** |
| **1396** | **chr 15: 102501355-102516768** | **DDX11L9** | **1** |
| **1396** | **chr 15: 102501355-102516768** | **WASH3P** | **1** |
| **1397** | **chr 16: 475618-573011** | **RAB11FIP3** | **1** |
| **1398** | **chr 16: 903633-1031596** | **LMF1** | **1** |
| **1399** | **chr 16: 2138710-2185899** | **PKD1** | **3** |
| **1399** | **chr 16: 2138710-2185899** | **TSC2** | **3** |
| **1400** | **chr 16: 2389150-2476700** | **ABCA17P** | **2** |
| **1400** | **chr 16: 2389150-2476700** | **ABCA3** | **2** |
| **1401** | **chr 16: 2787076-2802601** | **SRRM2-AS1** | **1** |
| **1402** | **chr 16: 3038961-3044510** | **LINC00514** | **1** |
| **1403** | **chr 16: 4003387-4166186** | **ADCY9** | **2** |
| **1404** | **chr 16: 4381549-4475706** | **GLIS2** | **1** |
| **1404** | **chr 16: 4381549-4475706** | **PAM16** | **1** |
| **1405** | **chr 16: 4746512-4817625** | **ANKS3** | **1** |
| **1406** | **chr 16: 4784272-4799397** | **ANKS3** | **1** |
| **1407** | **chr 16: 5008317-5137380** | **NAGPA** | **2** |
| **1407** | **chr 16: 5008317-5137380** | **SEC14L5** | **2** |
| **1408** | **chr 16: 5094122-5116111** | **C16ORF89** | **1** |
| **1409** | **chr 16: 5289802-7763340** | **RBFOX1** | **13** |
| **1410** | **chr 16: 8359963-8407862** | **none** | **1** |
| **1411** | **chr 16: 8946798-8962866** | **CARHSP1** | **2** |
| **1412** | **chr 16: 8985950-9058371** | **USP7** | **1** |
| **1413** | **chr 16: 9852375-10276611** | **GRIN2A** | **2** |
| **1414** | **chr 16: 10420290-10577495** | **ATF7IP2** | **1** |
| **1415** | **chr 16: 11343475-11445619** | **TNP2** | **1** |
| **1415** | **chr 16: 11343475-11445619** | **SOCS1** | **1** |
| **1416** | **chr 16: 11641852-11730237** | **LITAF** | **4** |
| **1417** | **chr 16: 11844441-11891123** | **ZC3H7A** | **1** |
| **1418** | **chr 16: 12070593-12668146** | **SNX29** | **2** |
| **1419** | **chr 16: 12756918-12897874** | **MIR4718** | **1** |
| **1419** | **chr 16: 12756918-12897874** | **CPPED1** | **1** |
| **1420** | **chr 16: 12995476-13334272** | **SHISA9** | **1** |
| **1421** | **chr 16: 15005407-15045915** | **NPIP** | **1** |
| **1422** | **chr 16: 15068447-15233196** | **NTAN1** | **1** |
| **1422** | **chr 16: 15068447-15233196** | **PDXDC1** | **1** |
| **1423** | **chr 16: 15198177-15248421** | **RRN3** | **3** |
| **1423** | **chr 16: 15198177-15248421** | **MIR3180-4** | **3** |
| **1424** | **chr 16: 15489610-15718885** | **C16ORF45** | **1** |
| **1424** | **chr 16: 15489610-15718885** | **MPV17L** | **1** |
| **1425** | **chr 16: 15797028-15950890** | **NDE1** | **2** |
| **1426** | **chr 16: 16242784-16317379** | **ABCC6** | **6** |
| **1427** | **chr 16: 16404197-16444447** | **PKD1P1** | **1** |
| **1428** | **chr 16: 16450080-16471364** | **PKD1P1** | **1** |
| **1429** | **chr 16: 17195625-17564738** | **XYLT1** | **3** |
| **1430** | **chr 16: 18027045-18266920** | **none** | **1** |
| **1431** | **chr 16: 18411798-18495797** | **MIR3180-1** | **3** |
| **1432** | **chr 16: 18938184-18948365** | **SMG1** | **1** |
| **1433** | **chr 16: 18995255-19075264** | **TMC7** | **2** |
| **1434** | **chr 16: 19098177-19109432** | **COQ7** | **2** |
| **1434** | **chr 16: 19098177-19109432** | **ITPRIPL2** | **2** |
| **1435** | **chr 16: 19125253-19279652** | **ITPRIPL2** | **2** |
| **1435** | **chr 16: 19125253-19279652** | **SYT17** | **2** |
| **1436** | **chr 16: 19727777-19868907** | **IQCK** | **1** |
| **1436** | **chr 16: 19727777-19868907** | **KNOP1** | **1** |
| **1437** | **chr 16: 20320893-20339130** | **GP2** | **1** |
| **1438** | **chr 16: 20420855-20452658** | **ACSM5** | **2** |
| **1439** | **chr 16: 20621564-20808903** | **ACSM1** | **1** |
| **1439** | **chr 16: 20621564-20808903** | **THUMPD1** | **1** |
| **1440** | **chr 16: 20944432-21170762** | **DNAH3** | **3** |
| **1440** | **chr 16: 20944432-21170762** | **TMEM159** | **3** |
| **1441** | **chr 16: 22308729-22346424** | **POLR3E** | **1** |
| **1442** | **chr 16: 23289551-23392620** | **SCNN1B** | **2** |
| **1443** | **chr 16: 23614487-23652631** | **PALB2** | **1** |
| **1443** | **chr 16: 23614487-23652631** | **DCTN5** | **1** |
| **1444** | **chr 16: 23617161-23635428** | **PALB2** | **1** |
| **1445** | **chr 16: 23847321-24231932** | **PRKCB** | **6** |
| **1446** | **chr 16: 24266873-24374122** | **CACNG3** | **2** |
| **1447** | **chr 16: 25123049-25189552** | **LCMT1** | **1** |
| **1447** | **chr 16: 25123049-25189552** | **LOC100506655** | **1** |
| **1448** | **chr 16: 25703346-26149009** | **HS3ST4** | **1** |
| **1449** | **chr 16: 26329427-26345749** | **none** | **1** |
| **1450** | **chr 16: 27214806-27233089** | **KDM8** | **1** |
| **1451** | **chr 16: 27279525-27301813** | **FLJ21408** | **1** |
| **1451** | **chr 16: 27279525-27301813** | **NSMCE1** | **1** |
| **1452** | **chr 16: 27798849-28074830** | **GSG1L** | **2** |
| **1453** | **chr 16: 28109299-28223241** | **XPO6** | **1** |
| **1454** | **chr 16: 28303839-28335170** | **SBK1** | **1** |
| **1455** | **chr 16: 29086162-29229181** | **RRN3P2** | **1** |
| **1456** | **chr 16: 29817426-29827201** | **MAZ** | **3** |
| **1457** | **chr 16: 29817816-29819053** | **MAZ** | **2** |
| **1458** | **chr 16: 29819965-29832573** | **MAZ** | **1** |
| **1459** | **chr 16: 29869677-29875057** | **LOC440356** | **1** |
| **1459** | **chr 16: 29869677-29875057** | **CDIPT** | **1** |
| **1460** | **chr 16: 30016829-30034591** | **DOC2A** | **1** |
| **1460** | **chr 16: 30016829-30034591** | **INO80E** | **1** |
| **1461** | **chr 16: 30483978-30534506** | **MIR4518** | **1** |
| **1461** | **chr 16: 30483978-30534506** | **ITGAL** | **1** |
| **1462** | **chr 16: 30773065-30787628** | **C16ORF93** | **1** |
| **1463** | **chr 16: 31366454-31394318** | **ITGAX** | **1** |
| **1464** | **chr 16: 32019758-32096106** | **none** | **1** |
| **1465** | **chr 16: 32963313-33039358** | **none** | **1** |
| **1466** | **chr 16: 33817728-33853828** | **none** | **3** |
| **1467** | **chr 16: 46614465-46655538** | **SHCBP1** | **1** |
| **1468** | **chr 16: 47188297-47498060** | **ITFG1** | **1** |
| **1468** | **chr 16: 47188297-47498060** | **PHKB** | **1** |
| **1469** | **chr 16: 47333357-47351725** | **ITFG1** | **1** |
| **1470** | **chr 16: 48116883-48189929** | **ABCC12** | **2** |
| **1471** | **chr 16: 48198862-48281479** | **ABCC11** | **1** |
| **1472** | **chr 16: 48657346-48778553** | **N4BP1** | **6** |
| **1473** | **chr 16: 49407733-49433342** | **C16ORF78** | **1** |
| **1474** | **chr 16: 49521434-49891830** | **ZNF423** | **6** |
| **1475** | **chr 16: 50424826-50429044** | **BRD7** | **1** |
| **1476** | **chr 16: 52039398-52112386** | **C16ORF97** | **1** |
| **1477** | **chr 16: 52586001-52686017** | **LOC643714** | **1** |
| **1478** | **chr 16: 52656026-52665000** | **LOC643714** | **1** |
| **1479** | **chr 16: 53069601-53086785** | **CHD9** | **2** |
| **1480** | **chr 16: 53088944-53363062** | **CHD9** | **1** |
| **1481** | **chr 16: 54968824-54988577** | **IRX5** | **1** |
| **1482** | **chr 16: 55293880-55367500** | **IRX6** | **1** |
| **1483** | **chr 16: 55357671-55364672** | **IRX6** | **1** |
| **1484** | **chr 16: 55423611-55540603** | **MMP2** | **5** |
| **1485** | **chr 16: 55461469-55496209** | **MMP2** | **2** |
| **1486** | **chr 16: 55762026-55784023** | **CES1P2** | **1** |
| **1487** | **chr 16: 55794459-55828070** | **CES1P1** | **1** |
| **1488** | **chr 16: 55836762-55867249** | **CES1** | **1** |
| **1489** | **chr 16: 55880065-55989943** | **CES5A** | **1** |
| **1490** | **chr 16: 56225301-56391356** | **LOC283856** | **2** |
| **1490** | **chr 16: 56225301-56391356** | **GNAO1** | **2** |
| **1491** | **chr 16: 56395363-56459450** | **AMFR** | **1** |
| **1492** | **chr 16: 56764016-56878797** | **NUP93** | **2** |
| **1493** | **chr 16: 56995761-57017757** | **CETP** | **1** |
| **1494** | **chr 16: 57023396-57117443** | **NLRC5** | **1** |
| **1495** | **chr 16: 57126448-57181878** | **CPNE2** | **1** |
| **1496** | **chr 16: 57715035-57735642** | **GPR97** | **1** |
| **1496** | **chr 16: 57715035-57735642** | **CCDC135** | **1** |
| **1497** | **chr 16: 57728704-57765717** | **CCDC135** | **1** |
| **1498** | **chr 16: 57769641-57791162** | **KATNB1** | **1** |
| **1499** | **chr 16: 57792128-57896957** | **KIFC3** | **1** |
| **1500** | **chr 16: 58163432-58193037** | **C16ORF80** | **1** |
| **1500** | **chr 16: 58163432-58193037** | **CSNK2A2** | **1** |
| **1501** | **chr 16: 58191810-58231824** | **CSNK2A2** | **1** |
| **1502** | **chr 16: 58265060-58317740** | **PRSS54** | **1** |
| **1502** | **chr 16: 58265060-58317740** | **CCDC113** | **1** |
| **1503** | **chr 16: 58455229-58496374** | **GINS3** | **1** |
| **1503** | **chr 16: 58455229-58496374** | **NDRG4** | **1** |
| **1504** | **chr 16: 58496749-58547532** | **NDRG4** | **1** |
| **1505** | **chr 16: 58767815-59142878** | **GOT2** | **1** |
| **1506** | **chr 16: 61681145-62070939** | **CDH8** | **1** |
| **1507** | **chr 16: 63348167-63651950** | **none** | **1** |
| **1508** | **chr 16: 64276511-64377813** | **none** | **1** |
| **1509** | **chr 16: 64398683-64634150** | **none** | **1** |
| **1510** | **chr 16: 64977655-65160015** | **CDH11** | **3** |
| **1511** | **chr 16: 65266958-65466723** | **LOC283867** | **5** |
| **1512** | **chr 16: 65318401-65610203** | **LOC283867** | **6** |
| **1513** | **chr 16: 65614570-65680850** | **LOC283867** | **2** |
| **1514** | **chr 16: 66400532-66438686** | **CDH5** | **1** |
| **1515** | **chr 16: 66648652-66730610** | **CMTM4** | **1** |
| **1516** | **chr 16: 66836777-66907159** | **NAE1** | **1** |
| **1516** | **chr 16: 66836777-66907159** | **CA7** | **1** |
| **1517** | **chr 16: 67471916-67515140** | **ATP6V0D1** | **1** |
| **1518** | **chr 16: 67552320-67580691** | **FAM65A** | **1** |
| **1519** | **chr 16: 67596309-67673086** | **CTCF** | **1** |
| **1520** | **chr 16: 67696847-67701168** | **ENKD1** | **1** |
| **1520** | **chr 16: 67696847-67701168** | **C16ORF86** | **1** |
| **1521** | **chr 16: 67757004-67840555** | **TSNAXIP1** | **1** |
| **1521** | **chr 16: 67757004-67840555** | **RANBP10** | **1** |
| **1522** | **chr 16: 67906925-67922758** | **NRN1L** | **1** |
| **1522** | **chr 16: 67906925-67922758** | **EDC4** | **1** |
| **1523** | **chr 16: 68021648-68263162** | **DDX28** | **1** |
| **1523** | **chr 16: 68021648-68263162** | **DPEP2** | **1** |
| **1524** | **chr 16: 68392230-68482591** | **SMPD3** | **3** |
| **1525** | **chr 16: 68670091-68756519** | **CDH3** | **1** |
| **1526** | **chr 16: 68877506-69119083** | **TANGO6** | **1** |
| **1527** | **chr 16: 69165193-69358949** | **CHTF8** | **2** |
| **1528** | **chr 16: 69598996-69738569** | **NFAT5** | **4** |
| **1528** | **chr 16: 69598996-69738569** | **MIR1538** | **4** |
| **1529** | **chr 16: 70147528-70195203** | **PDPR** | **1** |
| **1530** | **chr 16: 70190242-70207351** | **PDPR** | **1** |
| **1530** | **chr 16: 70190242-70207351** | **CLEC18C** | **1** |
| **1531** | **chr 16: 70488323-70514177** | **FUK** | **1** |
| **1532** | **chr 16: 70514470-70557468** | **SF3B3** | **3** |
| **1532** | **chr 16: 70514470-70557468** | **COG4** | **3** |
| **1533** | **chr 16: 70613797-70694585** | **IL34** | **2** |
| **1534** | **chr 16: 70721341-70835064** | **LOC100130894** | **2** |
| **1534** | **chr 16: 70721341-70835064** | **VAC14** | **2** |
| **1535** | **chr 16: 71315291-71323618** | **FTSJD1** | **1** |
| **1536** | **chr 16: 71963440-72033877** | **IST1** | **1** |
| **1537** | **chr 16: 72459383-72698908** | **none** | **1** |
| **1538** | **chr 16: 72816783-73093597** | **ZFHX3** | **2** |
| **1539** | **chr 16: 74088049-74330660** | **PSMD7** | **1** |
| **1540** | **chr 16: 74907467-75034071** | **ZNRF1** | **3** |
| **1540** | **chr 16: 74907467-75034071** | **WDR59** | **3** |
| **1541** | **chr 16: 75032927-75144892** | **ZNRF1** | **2** |
| **1542** | **chr 16: 75327595-75499395** | **TMEM170A** | **1** |
| **1542** | **chr 16: 75327595-75499395** | **CFDP1** | **1** |
| **1543** | **chr 16: 76141180-76181704** | **none** | **1** |
| **1544** | **chr 16: 76770238-76962066** | **MIR4719** | **1** |
| **1545** | **chr 16: 77235370-77469011** | **ADAMTS18** | **1** |
| **1545** | **chr 16: 77235370-77469011** | **SYCE1L** | **1** |
| **1546** | **chr 16: 77822426-78014004** | **VAT1L** | **2** |
| **1547** | **chr 16: 78133309-79246564** | **WWOX** | **24** |
| **1548** | **chr 16: 79710004-79841819** | **none** | **3** |
| **1549** | **chr 16: 79750621-79804460** | **none** | **2** |
| **1550** | **chr 16: 80099732-80201474** | **none** | **1** |
| **1551** | **chr 16: 80188949-80597032** | **DYNLRB2** | **1** |
| **1552** | **chr 16: 81134479-81253975** | **PKD1L2** | **2** |
| **1553** | **chr 16: 81478774-81745367** | **CMIP** | **4** |
| **1554** | **chr 16: 82660407-83830204** | **CDH13** | **4** |
| **1555** | **chr 16: 83841447-83853342** | **HSBP1** | **1** |
| **1556** | **chr 16: 84178864-84212373** | **DNAAF1** | **1** |
| **1556** | **chr 16: 84178864-84212373** | **HSDL1** | **1** |
| **1557** | **chr 16: 84211457-84220669** | **TAF1C** | **1** |
| **1557** | **chr 16: 84211457-84220669** | **DNAAF1** | **1** |
| **1558** | **chr 16: 84328251-84363450** | **WFDC1** | **2** |
| **1559** | **chr 16: 84682130-84701292** | **KLHL36** | **1** |
| **1560** | **chr 16: 84733583-84813528** | **USP10** | **1** |
| **1561** | **chr 16: 84853589-84954374** | **CRISPLD2** | **2** |
| **1562** | **chr 16: 85061374-85127836** | **KIAA0513** | **1** |
| **1563** | **chr 16: 85723689-85784735** | **C16ORF74** | **1** |
| **1563** | **chr 16: 85723689-85784735** | **MIR1910** | **1** |
| **1564** | **chr 16: 86508134-86542705** | **FENDRR** | **1** |
| **1565** | **chr 16: 87117167-87425748** | **C16ORF95** | **2** |
| **1566** | **chr 16: 87730090-87799598** | **JPH3** | **2** |
| **1567** | **chr 16: 87982849-88110924** | **BANP** | **1** |
| **1568** | **chr 16: 88210903-88219696** | **none** | **1** |
| **1569** | **chr 16: 88519724-88603424** | **MIR5189** | **1** |
| **1569** | **chr 16: 88519724-88603424** | **ZFPM1** | **1** |
| **1570** | **chr 16: 88579367-88597461** | **ZFPM1** | **1** |
| **1571** | **chr 16: 88781750-88851619** | **CTU2** | **1** |
| **1571** | **chr 16: 88781750-88851619** | **PIEZO1** | **1** |
| **1572** | **chr 16: 88809174-88812156** | **PIEZO1** | **1** |
| **1573** | **chr 16: 88941265-89043612** | **CBFA2T3** | **1** |
| **1574** | **chr 16: 89006196-89017932** | **CBFA2T3** | **1** |
| **1575** | **chr 16: 89050439-89061777** | **CBFA2T3** | **3** |
| **1576** | **chr 16: 89679715-89704839** | **DPEP1** | **1** |
| **1577** | **chr 16: 89747144-89762772** | **CDK10** | **2** |
| **1577** | **chr 16: 89747144-89762772** | **SPATA2L** | **2** |
| **1578** | **chr 16: 89749027-89752977** | **CDK10** | **2** |
| **1579** | **chr 16: 89939999-90005169** | **TCF25** | **1** |
| **1579** | **chr 16: 89939999-90005169** | **MC1R** | **1** |
| **1580** | **chr 16: 90106168-90114181** | **GAS8** | **2** |
| **1581** | **chr 17: 62292-236045** | **RPH3AL** | **2** |
| **1581** | **chr 17: 62292-236045** | **LOC100506388** | **2** |
| **1582** | **chr 17: 411907-624957** | **VPS53** | **2** |
| **1583** | **chr 17: 426379-443816** | **VPS53** | **1** |
| **1584** | **chr 17: 702552-883010** | **NXN** | **1** |
| **1585** | **chr 17: 906757-1132315** | **ABR** | **2** |
| **1586** | **chr 17: 1367391-1396106** | **MYO1C** | **1** |
| **1587** | **chr 17: 1619816-1641893** | **MIR22HG** | **1** |
| **1588** | **chr 17: 1629041-1641879** | **WDR81** | **1** |
| **1589** | **chr 17: 1837970-1928639** | **RTN4RL1** | **3** |
| **1590** | **chr 17: 1898907-1906965** | **RTN4RL1** | **2** |
| **1591** | **chr 17: 1963132-2207065** | **SMG6** | **2** |
| **1592** | **chr 17: 2308855-2415185** | **LOC284009** | **1** |
| **1592** | **chr 17: 2308855-2415185** | **METTL16** | **1** |
| **1593** | **chr 17: 2592679-2615957** | **CLUH** | **1** |
| **1594** | **chr 17: 3468737-3539616** | **TRPV1** | **1** |
| **1595** | **chr 17: 3617921-3704537** | **GSG2** | **1** |
| **1595** | **chr 17: 3617921-3704537** | **ITGAE** | **1** |
| **1596** | **chr 17: 4336982-4391503** | **SPNS3** | **1** |
| **1597** | **chr 17: 4402132-4442330** | **SPNS2** | **1** |
| **1597** | **chr 17: 4402132-4442330** | **MYBBP1A** | **1** |
| **1598** | **chr 17: 4736682-4801356** | **MINK1** | **2** |
| **1598** | **chr 17: 4736682-4801356** | **CHRNE** | **2** |
| **1599** | **chr 17: 4848946-4852356** | **PFN1** | **1** |
| **1600** | **chr 17: 4851386-4860426** | **PFN1** | **1** |
| **1600** | **chr 17: 4851386-4860426** | **ENO3** | **1** |
| **1601** | **chr 17: 4871286-4890960** | **SPAG7** | **1** |
| **1601** | **chr 17: 4871286-4890960** | **CAMTA2** | **1** |
| **1602** | **chr 17: 5328458-5372380** | **RPAIN** | **1** |
| **1603** | **chr 17: 5402746-5522744** | **NLRP1** | **3** |
| **1603** | **chr 17: 5402746-5522744** | **LOC728392** | **3** |
| **1604** | **chr 17: 5675553-6027747** | **WSCD1** | **2** |
| **1604** | **chr 17: 5675553-6027747** | **LOC339166** | **2** |
| **1605** | **chr 17: 6588031-6616886** | **SLC13A5** | **1** |
| **1606** | **chr 17: 7239847-7254797** | **KCTD11** | **1** |
| **1606** | **chr 17: 7239847-7254797** | **ACAP1** | **1** |
| **1607** | **chr 17: 7308192-7323179** | **TMEM256** | **1** |
| **1607** | **chr 17: 7308192-7323179** | **PLSCR3** | **1** |
| **1608** | **chr 17: 7452207-7464925** | **TNFSF12** | **1** |
| **1608** | **chr 17: 7452207-7464925** | **TNFSF13** | **1** |
| **1609** | **chr 17: 7905911-7923657** | **GUCY2D** | **3** |
| **1610** | **chr 17: 8150935-8173809** | **CTC1** | **1** |
| **1610** | **chr 17: 8150935-8173809** | **PFAS** | **1** |
| **1611** | **chr 17: 8316448-8393729** | **MYH10** | **1** |
| **1611** | **chr 17: 8316448-8393729** | **NDEL1** | **1** |
| **1612** | **chr 17: 8706040-8770994** | **PIK3R6** | **1** |
| **1613** | **chr 17: 8782232-8869029** | **PIK3R5** | **1** |
| **1614** | **chr 17: 8924858-9147317** | **NTN1** | **1** |
| **1615** | **chr 17: 9153787-9479908** | **STX8** | **1** |
| **1615** | **chr 17: 9153787-9479908** | **WDR16** | **1** |
| **1616** | **chr 17: 9548014-9633008** | **USP43** | **1** |
| **1617** | **chr 17: 10195136-10221243** | **MYH13** | **2** |
| **1618** | **chr 17: 10201400-10276447** | **MYH13** | **3** |
| **1619** | **chr 17: 10286448-10527203** | **MYH8** | **2** |
| **1619** | **chr 17: 10286448-10527203** | **MYH4** | **2** |
| **1620** | **chr 17: 10633093-10718481** | **TMEM220** | **1** |
| **1621** | **chr 17: 10752857-10784212** | **PIRT** | **1** |
| **1622** | **chr 17: 11144579-11467380** | **SHISA6** | **3** |
| **1623** | **chr 17: 11501747-11873065** | **DNAH9** | **2** |
| **1624** | **chr 17: 11857205-11900827** | **DNAH9** | **1** |
| **1625** | **chr 17: 12569206-12672266** | **MYOCD** | **1** |
| **1626** | **chr 17: 12663456-12693559** | **MYOCD** | **1** |
| **1627** | **chr 17: 12692855-12894960** | **ARHGAP44** | **1** |
| **1627** | **chr 17: 12692855-12894960** | **ELAC2** | **1** |
| **1628** | **chr 17: 13659794-13972812** | **COX10-AS1** | **2** |
| **1628** | **chr 17: 13659794-13972812** | **CDRT15P1** | **2** |
| **1629** | **chr 17: 14277455-14324343** | **none** | **2** |
| **1630** | **chr 17: 14737954-14803871** | **none** | **1** |
| **1631** | **chr 17: 15468796-15587625** | **CDRT1** | **1** |
| **1631** | **chr 17: 15468796-15587625** | **TRIM16** | **1** |
| **1632** | **chr 17: 15554903-15557803** | **TRIM16** | **1** |
| **1633** | **chr 17: 15932470-16121499** | **TTC19** | **2** |
| **1633** | **chr 17: 15932470-16121499** | **NCOR1** | **2** |
| **1634** | **chr 17: 16945858-17120993** | **MPRIP** | **1** |
| **1634** | **chr 17: 16945858-17120993** | **PLD6** | **1** |
| **1635** | **chr 17: 17584786-17714767** | **RAI1** | **1** |
| **1635** | **chr 17: 17584786-17714767** | **SMCR5** | **1** |
| **1636** | **chr 17: 17746827-17875736** | **LRRC48** | **1** |
| **1636** | **chr 17: 17746827-17875736** | **TOM1L2** | **1** |
| **1637** | **chr 17: 18128900-18148189** | **FLII** | **1** |
| **1637** | **chr 17: 18128900-18148189** | **LLGL1** | **1** |
| **1638** | **chr 17: 18853657-18925908** | **SLC5A10** | **1** |
| **1639** | **chr 17: 18923985-18950950** | **SLC5A10** | **2** |
| **1640** | **chr 17: 19030781-19062489** | **GRAPL** | **2** |
| **1641** | **chr 17: 19374431-19396273** | **SLC47A1** | **1** |
| **1642** | **chr 17: 19398697-19482347** | **SLC47A1** | **1** |
| **1642** | **chr 17: 19398697-19482347** | **SNORA59A** | **1** |
| **1643** | **chr 17: 19463423-19501235** | **SLC47A1** | **1** |
| **1644** | **chr 17: 19551448-19580911** | **ALDH3A2** | **2** |
| **1645** | **chr 17: 19912656-20306870** | **SPECC1** | **5** |
| **1646** | **chr 17: 20902909-20947073** | **USP22** | **1** |
| **1647** | **chr 17: 21536013-21544603** | **none** | **1** |
| **1648** | **chr 17: 26083791-26221778** | **NOS2** | **2** |
| **1648** | **chr 17: 26083791-26221778** | **LYRM9** | **2** |
| **1649** | **chr 17: 26583463-26592577** | **KRT18P55** | **1** |
| **1649** | **chr 17: 26583463-26592577** | **PPY2** | **1** |
| **1650** | **chr 17: 26603011-26644809** | **KRT18P55** | **1** |
| **1651** | **chr 17: 26782769-26941218** | **SLC13A2** | **1** |
| **1651** | **chr 17: 26782769-26941218** | **FOXN1** | **1** |
| **1652** | **chr 17: 26925807-26944393** | **SPAG5** | **2** |
| **1652** | **chr 17: 26925807-26944393** | **SPAG5-AS1** | **2** |
| **1653** | **chr 17: 26989108-27029697** | **SDF2** | **3** |
| **1654** | **chr 17: 26997071-26999822** | **SUPT6H** | **2** |
| **1655** | **chr 17: 27277530-27384234** | **PHF12** | **2** |
| **1656** | **chr 17: 27281918-27333458** | **SEZ6** | **2** |
| **1657** | **chr 17: 27400527-27507430** | **TIAF1** | **1** |
| **1657** | **chr 17: 27400527-27507430** | **MYO18A** | **1** |
| **1658** | **chr 17: 27679086-27878922** | **TAOK1** | **1** |
| **1658** | **chr 17: 27679086-27878922** | **MIR4523** | **1** |
| **1659** | **chr 17: 27952955-28257294** | **EFCAB5** | **1** |
| **1659** | **chr 17: 27952955-28257294** | **SSH2** | **1** |
| **1660** | **chr 17: 28256217-28435470** | **EFCAB5** | **2** |
| **1660** | **chr 17: 28256217-28435470** | **SSH2** | **2** |
| **1661** | **chr 17: 28417383-28444515** | **EFCAB5** | **1** |
| **1661** | **chr 17: 28417383-28444515** | **NSRP1** | **1** |
| **1662** | **chr 17: 28521336-28563020** | **SLC6A4** | **1** |
| **1663** | **chr 17: 28903481-28999807** | **SH3GL1P2** | **2** |
| **1663** | **chr 17: 28903481-28999807** | **LRRC37BP1** | **2** |
| **1664** | **chr 17: 29335514-29420145** | **DPRXP4** | **1** |
| **1664** | **chr 17: 29335514-29420145** | **MIR4733** | **1** |
| **1665** | **chr 17: 29910180-29964562** | **MIR365B** | **1** |
| **1666** | **chr 17: 30334890-30380523** | **SH3GL1P1** | **1** |
| **1666** | **chr 17: 30334890-30380523** | **LRRC37B** | **1** |
| **1667** | **chr 17: 30819539-31204195** | **MYO1D** | **1** |
| **1668** | **chr 17: 31340104-32522956** | **ASIC2** | **10** |
| **1669** | **chr 17: 33474835-33518721** | **UNC45B** | **1** |
| **1670** | **chr 17: 34083267-34122711** | **C17ORF50** | **1** |
| **1670** | **chr 17: 34083267-34122711** | **MMP28** | **1** |
| **1671** | **chr 17: 35218934-35293960** | **LHX1** | **2** |
| **1672** | **chr 17: 36187924-36244363** | **LOC284100** | **2** |
| **1673** | **chr 17: 36584661-36668628** | **ARHGAP23** | **1** |
| **1674** | **chr 17: 36686250-36762183** | **SRCIN1** | **2** |
| **1675** | **chr 17: 36890149-36906070** | **CISD3** | **3** |
| **1675** | **chr 17: 36890149-36906070** | **PCGF2** | **3** |
| **1676** | **chr 17: 37159500-37169285** | **FBXO47** | **1** |
| **1676** | **chr 17: 37159500-37169285** | **LRRC37A11P** | **1** |
| **1677** | **chr 17: 37366788-37382125** | **STAC2** | **1** |
| **1678** | **chr 17: 37415383-37558776** | **FBXL20** | **2** |
| **1679** | **chr 17: 37617763-37721160** | **CDK12** | **1** |
| **1680** | **chr 17: 37702356-37702497** | **CDK12** | **1** |
| **1681** | **chr 17: 38333262-38351908** | **RAPGEFL1** | **1** |
| **1682** | **chr 17: 39077681-39132178** | **KRT39** | **2** |
| **1682** | **chr 17: 39077681-39132178** | **KRT23** | **2** |
| **1683** | **chr 17: 39604434-39610307** | **KRT38** | **2** |
| **1683** | **chr 17: 39604434-39610307** | **KRT32** | **2** |
| **1684** | **chr 17: 39775688-39943183** | **KRT17** | **3** |
| **1684** | **chr 17: 39775688-39943183** | **KRT42P** | **3** |
| **1685** | **chr 17: 40023160-40086795** | **ACLY** | **3** |
| **1685** | **chr 17: 40023160-40086795** | **TTC25** | **3** |
| **1686** | **chr 17: 40351185-40428725** | **STAT5B** | **2** |
| **1687** | **chr 17: 40724332-40729849** | **MLX** | **1** |
| **1687** | **chr 17: 40724332-40729849** | **PSMC3IP** | **1** |
| **1688** | **chr 17: 40819931-40829048** | **PLEKHH3** | **1** |
| **1689** | **chr 17: 40932695-40948954** | **WNK4** | **1** |
| **1690** | **chr 17: 41102542-41132545** | **AARSD1** | **1** |
| **1690** | **chr 17: 41102542-41132545** | **PTGES3L** | **1** |
| **1691** | **chr 17: 41196311-41277500** | **BRCA1** | **1** |
| **1692** | **chr 17: 41605211-41656988** | **ETV4** | **1** |
| **1693** | **chr 17: 41622152-41687706** | **ETV4** | **1** |
| **1694** | **chr 17: 42015730-42016327** | **PPY** | **1** |
| **1694** | **chr 17: 42015730-42016327** | **FAM215A** | **1** |
| **1695** | **chr 17: 43002076-43025082** | **KIF18B** | **2** |
| **1696** | **chr 17: 43128977-43186384** | **DCAKD** | **1** |
| **1697** | **chr 17: 43224683-43229468** | **HEXIM1** | **2** |
| **1698** | **chr 17: 43340487-43394414** | **MAP3K14-AS1** | **1** |
| **1699** | **chr 17: 43971747-44105700** | **MAPT-AS1** | **2** |
| **1700** | **chr 17: 44107281-44302733** | **KANSL1** | **2** |
| **1701** | **chr 17: 44352149-44439130** | **LRRC37A** | **1** |
| **1701** | **chr 17: 44352149-44439130** | **ARL17A** | **1** |
| **1702** | **chr 17: 44594067-44657088** | **LRRC37A2** | **1** |
| **1702** | **chr 17: 44594067-44657088** | **ARL17A** | **1** |
| **1703** | **chr 17: 45331211-45518678** | **ITGB3** | **1** |
| **1703** | **chr 17: 45331211-45518678** | **EFCAB13** | **1** |
| **1704** | **chr 17: 46018871-46059140** | **PRR15L** | **1** |
| **1704** | **chr 17: 46018871-46059140** | **PNPO** | **1** |
| **1705** | **chr 17: 46210801-46507637** | **SKAP1** | **1** |
| **1705** | **chr 17: 46210801-46507637** | **MIR1203** | **1** |
| **1706** | **chr 17: 46684593-46710934** | **HOXB8** | **1** |
| **1706** | **chr 17: 46684593-46710934** | **HOXB7** | **1** |
| **1707** | **chr 17: 47209821-47247351** | **B4GALNT2** | **1** |
| **1708** | **chr 17: 47787693-47866542** | **FAM117A** | **1** |
| **1708** | **chr 17: 47787693-47866542** | **KAT7** | **1** |
| **1709** | **chr 17: 48211103-48227991** | **PPP1R9B** | **1** |
| **1710** | **chr 17: 48240883-48242677** | **SGCA** | **1** |
| **1711** | **chr 17: 48450580-48458844** | **MRPL27** | **1** |
| **1711** | **chr 17: 48450580-48458844** | **EME1** | **1** |
| **1712** | **chr 17: 48556160-48563336** | **RSAD1** | **1** |
| **1713** | **chr 17: 48638428-48704835** | **CACNA1G-AS1** | **2** |
| **1713** | **chr 17: 48638428-48704835** | **CACNA1G** | **2** |
| **1714** | **chr 17: 48712137-48769613** | **ABCC3** | **1** |
| **1715** | **chr 17: 48796904-48833574** | **LUC7L3** | **1** |
| **1716** | **chr 17: 49039534-49198226** | **SPAG9** | **2** |
| **1717** | **chr 17: 49119884-49162832** | **SPAG9** | **1** |
| **1718** | **chr 17: 53046087-53241646** | **COX11** | **2** |
| **1719** | **chr 17: 53545426-53588299** | **MMD** | **1** |
| **1720** | **chr 17: 54188318-54589020** | **ANKFN1** | **1** |
| **1721** | **chr 17: 54869273-54916134** | **DGKE** | **1** |
| **1721** | **chr 17: 54869273-54916134** | **C17ORF67** | **1** |
| **1722** | **chr 17: 55333211-55762046** | **MSI2** | **5** |
| **1723** | **chr 17: 56160775-56172897** | **DYNLL2** | **1** |
| **1724** | **chr 17: 56378591-56406152** | **BZRAP1-AS1** | **1** |
| **1724** | **chr 17: 56378591-56406152** | **BZRAP1** | **1** |
| **1725** | **chr 17: 56833229-57058983** | **PPM1E** | **3** |
| **1726** | **chr 17: 57409049-57479090** | **MIR4729** | **2** |
| **1726** | **chr 17: 57409049-57479090** | **YPEL2** | **2** |
| **1727** | **chr 17: 57508229-57604307** | **DHX40** | **1** |
| **1728** | **chr 17: 57784552-57919616** | **PTRH2** | **1** |
| **1728** | **chr 17: 57784552-57919616** | **VMP1** | **1** |
| **1729** | **chr 17: 58227296-58248260** | **CA4** | **2** |
| **1730** | **chr 17: 58256454-58499831** | **USP32** | **1** |
| **1730** | **chr 17: 58256454-58499831** | **SCARNA20** | **1** |
| **1731** | **chr 17: 59529764-59562471** | **TBX4** | **1** |
| **1732** | **chr 17: 60776604-60815283** | **MARCH10** | **2** |
| **1733** | **chr 17: 60778674-60885705** | **MARCH10** | **1** |
| **1734** | **chr 17: 61086916-61505060** | **MIR548W** | **1** |
| **1734** | **chr 17: 61086916-61505060** | **TANC2** | **1** |
| **1735** | **chr 17: 61458591-61509572** | **TANC2** | **1** |
| **1736** | **chr 17: 61554421-61599209** | **ACE** | **2** |
| **1737** | **chr 17: 61926651-61941739** | **TCAM1P** | **1** |
| **1738** | **chr 17: 61949371-61951126** | **CSH2** | **1** |
| **1739** | **chr 17: 61957577-61959295** | **GH2** | **1** |
| **1740** | **chr 17: 61972274-61974021** | **CSH1** | **1** |
| **1741** | **chr 17: 61986956-61996198** | **GH1** | **1** |
| **1741** | **chr 17: 61986956-61996198** | **CSHL1** | **1** |
| **1742** | **chr 17: 62224586-62340661** | **TEX2** | **1** |
| **1743** | **chr 17: 63524680-64188202** | **AXIN2** | **2** |
| **1743** | **chr 17: 63524680-64188202** | **CEP112** | **2** |
| **1744** | **chr 17: 64208150-64252643** | **APOH** | **2** |
| **1745** | **chr 17: 64298753-64806861** | **PRKCA** | **2** |
| **1745** | **chr 17: 64298753-64806861** | **MIR634** | **2** |
| **1746** | **chr 17: 64831234-64881603** | **CACNG5** | **1** |
| **1747** | **chr 17: 64961025-65029514** | **CACNG4** | **1** |
| **1748** | **chr 17: 65040705-65052909** | **CACNG1** | **1** |
| **1749** | **chr 17: 65066553-65242105** | **HELZ** | **1** |
| **1750** | **chr 17: 65373574-65693372** | **PITPNC1** | **2** |
| **1751** | **chr 17: 65821639-65980494** | **BPTF** | **1** |
| **1752** | **chr 17: 66148916-66155184** | **LINC00674** | **1** |
| **1752** | **chr 17: 66148916-66155184** | **LOC440461** | **1** |
| **1753** | **chr 17: 66255322-66547460** | **ARSG** | **2** |
| **1753** | **chr 17: 66255322-66547460** | **SLC16A6** | **2** |
| **1754** | **chr 17: 66263166-66287408** | **ARSG** | **2** |
| **1754** | **chr 17: 66263166-66287408** | **SLC16A6** | **2** |
| **1755** | **chr 17: 66531253-66597530** | **FAM20A** | **2** |
| **1755** | **chr 17: 66531253-66597530** | **PRKAR1A** | **2** |
| **1756** | **chr 17: 66553656-66578718** | **FAM20A** | **2** |
| **1757** | **chr 17: 66587936-66760023** | **FAM20A** | **1** |
| **1758** | **chr 17: 67573391-67899141** | **MAP2K6** | **2** |
| **1759** | **chr 17: 70319263-70636611** | **LINC00511** | **2** |
| **1759** | **chr 17: 70319263-70636611** | **LINC00673** | **2** |
| **1760** | **chr 17: 70642087-71088851** | **SLC39A11** | **2** |
| **1761** | **chr 17: 71330522-71640228** | **SDK2** | **7** |
| **1762** | **chr 17: 71733992-71752696** | **LINC00469** | **1** |
| **1763** | **chr 17: 72039590-72150351** | **RPL38** | **1** |
| **1764** | **chr 17: 72744751-72765492** | **SLC9A3R1** | **1** |
| **1764** | **chr 17: 72744751-72765492** | **MIR3615** | **1** |
| **1765** | **chr 17: 72772621-72835918** | **NAT9** | **2** |
| **1765** | **chr 17: 72772621-72835918** | **TMEM104** | **2** |
| **1766** | **chr 17: 72838161-72857627** | **GRIN2C** | **1** |
| **1767** | **chr 17: 72946837-72969261** | **HID1** | **1** |
| **1768** | **chr 17: 73131342-73179078** | **HN1** | **1** |
| **1769** | **chr 17: 73257754-73262454** | **GGA3** | **1** |
| **1770** | **chr 17: 74000582-74023533** | **TEN1-CDK3** | **1** |
| **1770** | **chr 17: 74000582-74023533** | **CDK3** | **1** |
| **1771** | **chr 17: 74077086-74117657** | **ZACN** | **1** |
| **1771** | **chr 17: 74077086-74117657** | **EXOC7** | **1** |
| **1772** | **chr 17: 74136636-74150731** | **FOXJ1** | **1** |
| **1772** | **chr 17: 74136636-74150731** | **RNF157-AS1** | **1** |
| **1773** | **chr 17: 74138533-74236454** | **RNF157** | **1** |
| **1773** | **chr 17: 74138533-74236454** | **RNF157-AS1** | **1** |
| **1774** | **chr 17: 74668632-74707098** | **MXRA7** | **1** |
| **1775** | **chr 17: 75082797-75213179** | **SEC14L1** | **2** |
| **1775** | **chr 17: 75082797-75213179** | **LINC00338** | **2** |
| **1776** | **chr 17: 75276650-75496678** | **40057** | **1** |
| **1777** | **chr 17: 76356533-76369992** | **SOCS3** | **1** |
| **1778** | **chr 17: 76419777-76573476** | **DNAH17** | **1** |
| **1778** | **chr 17: 76419777-76573476** | **PGS1** | **1** |
| **1779** | **chr 17: 76480963-76499138** | **DNAH17** | **1** |
| **1780** | **chr 17: 76849058-76921469** | **LOC100653515** | **3** |
| **1780** | **chr 17: 76849058-76921469** | **TIMP2** | **3** |
| **1781** | **chr 17: 77085426-77613550** | **RBFOX3** | **5** |
| **1782** | **chr 17: 77765930-77775482** | **CBX8** | **1** |
| **1783** | **chr 17: 78010434-78074412** | **TBC1D16** | **1** |
| **1784** | **chr 17: 80059335-80170706** | **CCDC57** | **1** |
| **1785** | **chr 17: 80186272-80219005** | **SLC16A3** | **1** |
| **1786** | **chr 17: 80196898-80231607** | **SLC16A3** | **1** |
| **1787** | **chr 17: 80347098-80376513** | **HEXDC** | **1** |
| **1787** | **chr 17: 80347098-80376513** | **OGFOD3** | **1** |
| **1788** | **chr 17: 80477588-80602538** | **WDR45B** | **6** |
| **1788** | **chr 17: 80477588-80602538** | **FOXK2** | **6** |
| **1789** | **chr 17: 80612848-80656604** | **RAB40B** | **1** |
| **1790** | **chr 17: 80709939-80900724** | **ZNF750** | **1** |
| **1790** | **chr 17: 80709939-80900724** | **TBCD** | **1** |
| **1791** | **chr 17: 81159764-81168959** | **FLJ43681** | **1** |
| **1792** | **chr 18: 319360-500722** | **COLEC12** | **1** |
| **1793** | **chr 18: 1509183-1647098** | **none** | **1** |
| **1794** | **chr 18: 2847027-2915991** | **EMILIN2** | **1** |
| **1795** | **chr 18: 3496029-4455335** | **DLGAP1** | **10** |
| **1796** | **chr 18: 4264601-4295405** | **DLGAP1** | **1** |
| **1797** | **chr 18: 5392382-5630699** | **EPB41L3** | **1** |
| **1798** | **chr 18: 5954704-6415236** | **MIR4317** | **1** |
| **1798** | **chr 18: 5954704-6415236** | **L3MBTL4** | **1** |
| **1799** | **chr 18: 6025089-6082454** | **L3MBTL4** | **1** |
| **1800** | **chr 18: 7566779-8406859** | **PTPRM** | **6** |
| **1800** | **chr 18: 7566779-8406859** | **LOC100192426** | **6** |
| **1801** | **chr 18: 8705658-8832776** | **SOGA2** | **1** |
| **1802** | **chr 18: 9102627-9285983** | **NDUFV2** | **1** |
| **1802** | **chr 18: 9102627-9285983** | **ANKRD12** | **1** |
| **1803** | **chr 18: 10454624-10489945** | **APCDD1** | **1** |
| **1804** | **chr 18: 10666479-11148587** | **PIEZO2** | **1** |
| **1805** | **chr 18: 11688954-11885684** | **GNAL** | **2** |
| **1806** | **chr 18: 12446510-12658133** | **SPIRE1** | **2** |
| **1807** | **chr 18: 12658041-12725739** | **SPIRE1** | **1** |
| **1807** | **chr 18: 12658041-12725739** | **PSMG2** | **1** |
| **1808** | **chr 18: 18822202-19105378** | **GREB1L** | **1** |
| **1809** | **chr 18: 19109241-19180845** | **ESCO1** | **1** |
| **1810** | **chr 18: 20378223-20606451** | **MIR4741** | **1** |
| **1810** | **chr 18: 20378223-20606451** | **RBBP8** | **1** |
| **1811** | **chr 18: 20777107-21017925** | **CABLES1** | **3** |
| **1812** | **chr 18: 21269406-21535030** | **LAMA3** | **3** |
| **1813** | **chr 18: 21572736-21715574** | **TTC39C** | **2** |
| **1814** | **chr 18: 21574176-21595303** | **TTC39C** | **2** |
| **1815** | **chr 18: 22641889-22932154** | **ZNF521** | **2** |
| **1816** | **chr 18: 24034873-24237365** | **KCTD1** | **1** |
| **1817** | **chr 18: 24235705-24770662** | **LOC728606** | **1** |
| **1817** | **chr 18: 24235705-24770662** | **KCTD1** | **1** |
| **1818** | **chr 18: 24916342-25175128** | **none** | **1** |
| **1819** | **chr 18: 25530929-25757410** | **CDH2** | **1** |
| **1820** | **chr 18: 28681550-28742752** | **DSC2** | **1** |
| **1820** | **chr 18: 28681550-28742752** | **DSC1** | **1** |
| **1821** | **chr 18: 30413416-30518280** | **CCDC178** | **1** |
| **1822** | **chr 18: 30517365-31021065** | **CCDC178** | **2** |
| **1823** | **chr 18: 32073253-32471808** | **DTNA** | **2** |
| **1824** | **chr 18: 33161080-33291798** | **GALNT1** | **2** |
| **1824** | **chr 18: 33161080-33291798** | **MIR3975** | **2** |
| **1825** | **chr 18: 33531765-33546081** | **C18ORF21** | **3** |
| **1825** | **chr 18: 33531765-33546081** | **MIR187** | **3** |
| **1826** | **chr 18: 33877676-34360018** | **TPGS2** | **3** |
| **1826** | **chr 18: 33877676-34360018** | **FHOD3** | **3** |
| **1827** | **chr 18: 34409068-34812135** | **TPGS2** | **2** |
| **1828** | **chr 18: 34823009-35146000** | **CELF4** | **3** |
| **1829** | **chr 18: 37421137-37504804** | **none** | **1** |
| **1830** | **chr 18: 39047394-39212149** | **KC6** | **1** |
| **1831** | **chr 18: 39739246-40271387** | **LOC284260** | **1** |
| **1832** | **chr 18: 40323191-40695657** | **RIT2** | **1** |
| **1833** | **chr 18: 42260137-42648475** | **SETBP1** | **2** |
| **1834** | **chr 18: 42792959-43263072** | **SLC14A2** | **1** |
| **1835** | **chr 18: 43906771-44043103** | **RNF165** | **2** |
| **1836** | **chr 18: 44388352-44500123** | **PIAS2** | **1** |
| **1837** | **chr 18: 44497454-44627658** | **PIAS2** | **2** |
| **1838** | **chr 18: 44812095-45120914** | **none** | **2** |
| **1839** | **chr 18: 45553043-45937123** | **ZBTB7C** | **3** |
| **1840** | **chr 18: 46065416-46389588** | **CTIF** | **3** |
| **1840** | **chr 18: 46065416-46389588** | **MIR4743** | **3** |
| **1841** | **chr 18: 46570038-46987717** | **DYM** | **1** |
| **1841** | **chr 18: 46570038-46987717** | **MIR4744** | **1** |
| **1842** | **chr 18: 47309868-47721463** | **ACAA2** | **2** |
| **1842** | **chr 18: 47309868-47721463** | **SCARNA17** | **2** |
| **1843** | **chr 18: 49866541-51057784** | **DCC** | **3** |
| **1844** | **chr 18: 52385090-52562747** | **RAB27B** | **1** |
| **1845** | **chr 18: 52553096-52565314** | **RAB27B** | **1** |
| **1846** | **chr 18: 52889561-53332018** | **TCF4** | **1** |
| **1847** | **chr 18: 55297533-55405276** | **ATP8B1** | **4** |
| **1847** | **chr 18: 55297533-55405276** | **LOC100505549** | **4** |
| **1848** | **chr 18: 55313657-55470333** | **ATP8B1** | **4** |
| **1848** | **chr 18: 55313657-55470333** | **LOC100505549** | **4** |
| **1849** | **chr 18: 55711598-56068772** | **NEDD4L** | **1** |
| **1850** | **chr 18: 56529831-56653712** | **ZNF532** | **1** |
| **1851** | **chr 18: 59252978-59416065** | **CDH20** | **1** |
| **1852** | **chr 18: 59992519-60058516** | **TNFRSF11A** | **1** |
| **1853** | **chr 18: 60382671-60647666** | **PHLPP1** | **5** |
| **1854** | **chr 18: 60790578-60987361** | **BCL2** | **1** |
| **1855** | **chr 18: 61771324-62090836** | **LOC284294** | **2** |
| **1855** | **chr 18: 61771324-62090836** | **LINC00305** | **2** |
| **1856** | **chr 18: 63273325-63319289** | **none** | **1** |
| **1857** | **chr 18: 65183782-65566856** | **DSEL** | **3** |
| **1857** | **chr 18: 65183782-65566856** | **LOC643542** | **3** |
| **1858** | **chr 18: 67068290-67516323** | **DOK6** | **4** |
| **1859** | **chr 18: 67671028-67873181** | **RTTN** | **1** |
| **1860** | **chr 18: 70203914-70305756** | **CBLN2** | **2** |
| **1861** | **chr 18: 70409548-70535381** | **NETO1** | **2** |
| **1862** | **chr 18: 71983047-72026422** | **C18ORF63** | **1** |
| **1863** | **chr 18: 72201674-72254448** | **CNDP1** | **1** |
| **1864** | **chr 18: 72265105-72777627** | **LOC400657** | **1** |
| **1864** | **chr 18: 72265105-72777627** | **ZNF407** | **1** |
| **1865** | **chr 18: 74069643-74207146** | **FLJ44313** | **3** |
| **1865** | **chr 18: 74069643-74207146** | **ZNF516** | **3** |
| **1866** | **chr 18: 74084671-74090230** | **ZNF516** | **1** |
| **1867** | **chr 18: 74240611-74322925** | **LOC284276** | **1** |
| **1868** | **chr 18: 74690782-74845639** | **MBP** | **3** |
| **1869** | **chr 18: 77398927-77439745** | **CTDP1** | **5** |
| **1870** | **chr 19: 60104-71626** | **WASH5P** | **1** |
| **1871** | **chr 19: 416582-460996** | **SHC2** | **1** |
| **1872** | **chr 19: 463345-474983** | **ODF3L2** | **2** |
| **1873** | **chr 19: 531713-542097** | **CDC34** | **1** |
| **1874** | **chr 19: 544033-549919** | **GZMM** | **1** |
| **1875** | **chr 19: 1065921-1086627** | **HMHA1** | **1** |
| **1876** | **chr 19: 1189405-1228428** | **STK11** | **1** |
| **1877** | **chr 19: 1481426-1490751** | **PCSK4** | **1** |
| **1877** | **chr 19: 1481426-1490751** | **REEP6** | **1** |
| **1878** | **chr 19: 1752371-1780987** | **ONECUT3** | **1** |
| **1879** | **chr 19: 1782073-1812275** | **ATP8B3** | **2** |
| **1880** | **chr 19: 1815247-1848452** | **REXO1** | **1** |
| **1880** | **chr 19: 1815247-1848452** | **MIR1909** | **1** |
| **1881** | **chr 19: 1985446-2034880** | **BTBD2** | **3** |
| **1882** | **chr 19: 2071036-2096672** | **MOB3A** | **1** |
| **1882** | **chr 19: 2071036-2096672** | **IZUMO4** | **1** |
| **1883** | **chr 19: 2511216-2702707** | **GNG7** | **5** |
| **1884** | **chr 19: 2997635-3047633** | **TLE2** | **1** |
| **1885** | **chr 19: 3769086-3772233** | **RAX2** | **1** |
| **1886** | **chr 19: 3804021-3869030** | **ZFR2** | **1** |
| **1887** | **chr 19: 3958450-3971121** | **MIR637** | **1** |
| **1887** | **chr 19: 3958450-3971121** | **DAPK3** | **1** |
| **1888** | **chr 19: 4007643-4039384** | **PIAS4** | **1** |
| **1889** | **chr 19: 4153597-4173051** | **CREB3L3** | **1** |
| **1890** | **chr 19: 4360366-4400544** | **SH3GL1** | **1** |
| **1891** | **chr 19: 4641385-4670382** | **TNFAIP8L1** | **1** |
| **1892** | **chr 19: 4969124-5153606** | **KDM4B** | **1** |
| **1893** | **chr 19: 5158505-5340814** | **PTPRS** | **4** |
| **1894** | **chr 19: 5720687-5778745** | **LONP1** | **1** |
| **1895** | **chr 19: 5993174-6199583** | **LOC100128568** | **1** |
| **1895** | **chr 19: 5993174-6199583** | **RFX2** | **1** |
| **1896** | **chr 19: 6109332-6125808** | **RFX2** | **2** |
| **1897** | **chr 19: 6212965-6279959** | **MLLT1** | **1** |
| **1898** | **chr 19: 6467217-6482568** | **DENND1C** | **1** |
| **1898** | **chr 19: 6467217-6482568** | **CRB3** | **1** |
| **1899** | **chr 19: 6887576-6940470** | **EMR1** | **2** |
| **1900** | **chr 19: 7112265-7294045** | **INSR** | **7** |
| **1901** | **chr 19: 7413847-7537363** | **ARHGEF18** | **2** |
| **1902** | **chr 19: 7557861-7585912** | **ZNF358** | **1** |
| **1902** | **chr 19: 7557861-7585912** | **C19ORF45** | **1** |
| **1903** | **chr 19: 8130285-8214730** | **FBN3** | **1** |
| **1904** | **chr 19: 8585673-8642461** | **MYO1F** | **1** |
| **1905** | **chr 19: 9964393-10047228** | **OLFM2** | **2** |
| **1906** | **chr 19: 10982188-11033453** | **YIPF2** | **1** |
| **1906** | **chr 19: 10982188-11033453** | **CARM1** | **1** |
| **1907** | **chr 19: 11309970-11373157** | **C19ORF80** | **2** |
| **1907** | **chr 19: 11309970-11373157** | **DOCK6** | **2** |
| **1908** | **chr 19: 11562140-11639989** | **ZNF653** | **4** |
| **1908** | **chr 19: 11562140-11639989** | **ELAVL3** | **4** |
| **1909** | **chr 19: 11670188-11729950** | **ELOF1** | **2** |
| **1909** | **chr 19: 11670188-11729950** | **ACP5** | **2** |
| **1910** | **chr 19: 12873816-12983554** | **HOOK2** | **1** |
| **1910** | **chr 19: 12873816-12983554** | **JUNB** | **1** |
| **1911** | **chr 19: 13106421-13209610** | **NFIX** | **3** |
| **1912** | **chr 19: 13215715-13228381** | **TRMT1** | **1** |
| **1913** | **chr 19: 13317255-13734804** | **CACNA1A** | **6** |
| **1914** | **chr 19: 14063303-14072254** | **DCAF15** | **1** |
| **1914** | **chr 19: 14063303-14072254** | **PODNL1** | **1** |
| **1915** | **chr 19: 14142559-14163743** | **IL27RA** | **2** |
| **1916** | **chr 19: 14198651-14201848** | **SAMD1** | **1** |
| **1917** | **chr 19: 14365000-14404017** | **LPHN1** | **2** |
| **1918** | **chr 19: 14676889-14682874** | **NDUFB7** | **1** |
| **1919** | **chr 19: 14843204-14889353** | **EMR2** | **3** |
| **1920** | **chr 19: 14903301-14946188** | **OR7C1** | **1** |
| **1920** | **chr 19: 14903301-14946188** | **OR7A5** | **1** |
| **1921** | **chr 19: 15579455-15609767** | **PGLYRP2** | **3** |
| **1922** | **chr 19: 15904760-15905892** | **OR10H5** | **1** |
| **1923** | **chr 19: 16466049-16582896** | **EPS15L1** | **2** |
| **1924** | **chr 19: 16830786-16928774** | **NWD1** | **2** |
| **1925** | **chr 19: 17003757-17137625** | **CPAMD8** | **3** |
| **1926** | **chr 19: 17342691-17393595** | **USHBP1** | **1** |
| **1926** | **chr 19: 17342691-17393595** | **NR2F6** | **1** |
| **1927** | **chr 19: 17434031-17445638** | **ANO8** | **1** |
| **1927** | **chr 19: 17434031-17445638** | **DDA1** | **1** |
| **1928** | **chr 19: 17546317-17571763** | **NXNL1** | **1** |
| **1928** | **chr 19: 17546317-17571763** | **TMEM221** | **1** |
| **1929** | **chr 19: 17712136-17799401** | **UNC13A** | **1** |
| **1930** | **chr 19: 17982781-18005983** | **SLC5A5** | **1** |
| **1931** | **chr 19: 18208602-18262502** | **MAST3** | **2** |
| **1932** | **chr 19: 18794486-18893004** | **CRTC1** | **1** |
| **1933** | **chr 19: 19010322-19052070** | **DDX49** | **1** |
| **1933** | **chr 19: 19010322-19052070** | **COPE** | **1** |
| **1934** | **chr 19: 19229977-19249322** | **TMEM161A** | **1** |
| **1935** | **chr 19: 19256375-19303400** | **MEF2B** | **1** |
| **1935** | **chr 19: 19256375-19303400** | **MEF2BNB** | **1** |
| **1936** | **chr 19: 19322781-19363042** | **NCAN** | **1** |
| **1937** | **chr 19: 20011721-20074273** | **ZNF93** | **1** |
| **1938** | **chr 19: 20236185-20432114** | **ZNF486** | **1** |
| **1939** | **chr 19: 21106027-21133503** | **ZNF85** | **1** |
| **1940** | **chr 19: 28926294-29218684** | **none** | **1** |
| **1941** | **chr 19: 29777917-30017855** | **VSTM2B** | **5** |
| **1941** | **chr 19: 29777917-30017855** | **LOC284395** | **5** |
| **1942** | **chr 19: 30094923-30108144** | **POP4** | **1** |
| **1943** | **chr 19: 30719196-31204445** | **ZNF536** | **14** |
| **1944** | **chr 19: 31437487-31448098** | **none** | **5** |
| **1945** | **chr 19: 32880955-32896466** | **DPY19L3** | **1** |
| **1945** | **chr 19: 32880955-32896466** | **LOC400684** | **1** |
| **1946** | **chr 19: 33210658-33320483** | **TDRD12** | **1** |
| **1947** | **chr 19: 33321414-33360672** | **SLC7A9** | **1** |
| **1948** | **chr 19: 33463114-33469128** | **CEP89** | **1** |
| **1948** | **chr 19: 33463114-33469128** | **C19ORF40** | **1** |
| **1949** | **chr 19: 33571785-33621448** | **GPATCH1** | **1** |
| **1950** | **chr 19: 33699569-33716756** | **LRP3** | **1** |
| **1950** | **chr 19: 33699569-33716756** | **SLC7A10** | **1** |
| **1951** | **chr 19: 35485687-35517375** | **GRAMD1A** | **1** |
| **1952** | **chr 19: 35615416-35633355** | **LGI4** | **1** |
| **1952** | **chr 19: 35615416-35633355** | **FXYD1** | **1** |
| **1953** | **chr 19: 35629711-35645204** | **FXYD1** | **4** |
| **1954** | **chr 19: 36195428-36207940** | **ZBTB32** | **1** |
| **1954** | **chr 19: 36195428-36207940** | **MLL4** | **1** |
| **1955** | **chr 19: 36265433-36279724** | **ARHGAP33** | **1** |
| **1956** | **chr 19: 36316865-36360189** | **KIRREL2** | **1** |
| **1956** | **chr 19: 36316865-36360189** | **NPHS1** | **1** |
| **1957** | **chr 19: 36505409-36536874** | **ALKBH6** | **1** |
| **1958** | **chr 19: 36525886-36545664** | **THAP8** | **1** |
| **1958** | **chr 19: 36525886-36545664** | **WDR62** | **1** |
| **1959** | **chr 19: 36827161-36870101** | **ZFP14** | **1** |
| **1960** | **chr 19: 38397867-38699012** | **SIPA1L3** | **6** |
| **1960** | **chr 19: 38397867-38699012** | **WDR87** | **6** |
| **1961** | **chr 19: 38924338-39078204** | **RYR1** | **1** |
| **1962** | **chr 19: 39138288-39222223** | **CAPN12** | **1** |
| **1962** | **chr 19: 39138288-39222223** | **ACTN4** | **1** |
| **1963** | **chr 19: 39574552-39602133** | **PAPL** | **1** |
| **1964** | **chr 19: 40534166-40596845** | **ZNF780A** | **1** |
| **1964** | **chr 19: 40534166-40596845** | **ZNF780B** | **1** |
| **1965** | **chr 19: 40825442-40854434** | **C19ORF47** | **1** |
| **1966** | **chr 19: 40953695-40971747** | **BLVRB** | **1** |
| **1967** | **chr 19: 41277552-41406413** | **MIA** | **4** |
| **1968** | **chr 19: 41725107-41767671** | **HNRNPUL1** | **1** |
| **1968** | **chr 19: 41725107-41767671** | **AXL** | **1** |
| **1969** | **chr 19: 41807491-41859816** | **HNRNPUL1** | **1** |
| **1970** | **chr 19: 41856757-41930910** | **TGFB1** | **4** |
| **1970** | **chr 19: 41856757-41930910** | **B9D2** | **4** |
| **1971** | **chr 19: 42348805-42356401** | **LYPD4** | **1** |
| **1971** | **chr 19: 42348805-42356401** | **DMRTC2** | **1** |
| **1972** | **chr 19: 42502472-42573650** | **GRIK5** | **1** |
| **1973** | **chr 19: 42572628-42585701** | **ZNF574** | **2** |
| **1974** | **chr 19: 42590262-42700737** | **POU2F2** | **4** |
| **1975** | **chr 19: 42901279-43156507** | **LOC100996307** | **2** |
| **1975** | **chr 19: 42901279-43156507** | **LIPE** | **2** |
| **1976** | **chr 19: 43979105-44009087** | **PHLDB3** | **1** |
| **1977** | **chr 19: 44047191-44100287** | **XRCC1** | **1** |
| **1977** | **chr 19: 44047191-44100287** | **PINLYP** | **1** |
| **1978** | **chr 19: 44126519-44143991** | **CADM4** | **1** |
| **1979** | **chr 19: 44270684-44285409** | **KCNN4** | **1** |
| **1980** | **chr 19: 45135499-45222031** | **PVR** | **1** |
| **1980** | **chr 19: 45135499-45222031** | **IGSF23** | **1** |
| **1981** | **chr 19: 45417503-45422606** | **APOC1** | **1** |
| **1982** | **chr 19: 45430060-45434643** | **APOC1P1** | **1** |
| **1983** | **chr 19: 45582452-45808541** | **GEMIN7** | **1** |
| **1984** | **chr 19: 45809671-45826235** | **CKM** | **1** |
| **1985** | **chr 19: 45992034-46005768** | **RTN2** | **1** |
| **1986** | **chr 19: 46272974-46296060** | **SIX5** | **2** |
| **1986** | **chr 19: 46272974-46296060** | **DMPK** | **2** |
| **1987** | **chr 19: 46318667-46366548** | **SYMPK** | **2** |
| **1987** | **chr 19: 46318667-46366548** | **RSPH6A** | **2** |
| **1988** | **chr 19: 47341392-47354249** | **AP2S1** | **1** |
| **1989** | **chr 19: 47421932-47508334** | **ARHGAP35** | **1** |
| **1990** | **chr 19: 48110780-48246391** | **GLTSCR1** | **2** |
| **1990** | **chr 19: 48110780-48246391** | **EHD2** | **2** |
| **1991** | **chr 19: 48322702-48364769** | **CRX** | **1** |
| **1992** | **chr 19: 48497907-48528410** | **ELSPBP1** | **4** |
| **1993** | **chr 19: 48551099-48614074** | **PLA2G4C** | **1** |
| **1994** | **chr 19: 48564627-48568202** | **PLA2G4C** | **1** |
| **1995** | **chr 19: 48673948-48790865** | **CARD8** | **1** |
| **1995** | **chr 19: 48673948-48790865** | **LIG1** | **1** |
| **1996** | **chr 19: 48799713-48825151** | **CCDC114** | **2** |
| **1997** | **chr 19: 49316273-49339935** | **HSD17B14** | **1** |
| **1998** | **chr 19: 49891474-49921251** | **CCDC155** | **1** |
| **1999** | **chr 19: 50194154-50216988** | **ADM5** | **2** |
| **1999** | **chr 19: 50194154-50216988** | **CPT1C** | **2** |
| **2000** | **chr 19: 50270224-50310370** | **FUZ** | **1** |
| **2000** | **chr 19: 50270224-50310370** | **AP2A1** | **1** |
| **2001** | **chr 19: 50310125-50320633** | **FUZ** | **1** |
| **2001** | **chr 19: 50310125-50320633** | **AP2A1** | **1** |
| **2002** | **chr 19: 50392910-50464429** | **IL4I1** | **1** |
| **2003** | **chr 19: 50431958-50437192** | **IL4I1** | **1** |
| **2004** | **chr 19: 50887460-50934570** | **SPIB** | **1** |
| **2004** | **chr 19: 50887460-50934570** | **POLD1** | **1** |
| **2005** | **chr 19: 51020148-51071302** | **LRRC4B** | **1** |
| **2006** | **chr 19: 51124563-51222707** | **SYT3** | **4** |
| **2007** | **chr 19: 51152701-51162567** | **C19ORF81** | **1** |
| **2008** | **chr 19: 51328544-51340469** | **KLK15** | **1** |
| **2009** | **chr 19: 51333785-51354345** | **KLK15** | **1** |
| **2010** | **chr 19: 51471465-51515385** | **KLK6** | **1** |
| **2011** | **chr 19: 51499262-51512890** | **KLK8** | **1** |
| **2011** | **chr 19: 51499262-51512890** | **KLK9** | **1** |
| **2012** | **chr 19: 51656179-51685222** | **SIGLEC17P** | **1** |
| **2012** | **chr 19: 51656179-51685222** | **SIGLEC7** | **1** |
| **2013** | **chr 19: 51897741-51906904** | **LOC147646** | **1** |
| **2013** | **chr 19: 51897741-51906904** | **SIGLEC10** | **1** |
| **2014** | **chr 19: 51900008-51906780** | **LOC147646** | **1** |
| **2014** | **chr 19: 51900008-51906780** | **SIGLEC10** | **1** |
| **2015** | **chr 19: 52616343-52674896** | **ZNF836** | **2** |
| **2015** | **chr 19: 52616343-52674896** | **ZNF616** | **2** |
| **2016** | **chr 19: 53430387-53541151** | **ZNF321P** | **1** |
| **2016** | **chr 19: 53430387-53541151** | **ZNF816-ZNF321P** | **1** |
| **2017** | **chr 19: 53868945-53930574** | **ZNF765** | **1** |
| **2017** | **chr 19: 53868945-53930574** | **ZNF525** | **1** |
| **2018** | **chr 19: 54369476-54379691** | **MYADM** | **1** |
| **2019** | **chr 19: 54382443-54410906** | **PRKCG** | **2** |
| **2020** | **chr 19: 54466293-54493469** | **CACNG8** | **1** |
| **2020** | **chr 19: 54466293-54493469** | **MIR935** | **1** |
| **2021** | **chr 19: 54597932-54606000** | **OSCAR** | **2** |
| **2021** | **chr 19: 54597932-54606000** | **NDUFA3** | **2** |
| **2022** | **chr 19: 55644161-55660722** | **TNNT1** | **1** |
| **2023** | **chr 19: 55890612-55895966** | **TMEM238** | **1** |
| **2024** | **chr 19: 56165511-56186081** | **U2AF2** | **1** |
| **2024** | **chr 19: 56165511-56186081** | **EPN1** | **1** |
| **2025** | **chr 19: 56173266-56186081** | **U2AF2** | **1** |
| **2025** | **chr 19: 56173266-56186081** | **EPN1** | **1** |
| **2026** | **chr 19: 56186591-56221224** | **EPN1** | **1** |
| **2027** | **chr 19: 56598731-56632649** | **ZNF787** | **1** |
| **2028** | **chr 19: 56732680-56879752** | **ZSCAN5A** | **1** |
| **2028** | **chr 19: 56732680-56879752** | **ZNF542** | **1** |
| **2029** | **chr 19: 56784137-56821819** | **ZSCAN5A** | **1** |
| **2030** | **chr 19: 58125600-58134724** | **ZNF134** | **1** |
| **2031** | **chr 19: 58360098-58427978** | **ZNF587** | **1** |
| **2031** | **chr 19: 58360098-58427978** | **ZNF814** | **1** |
| **2032** | **chr 19: 58570606-58597677** | **ZNF135** | **1** |
| **2033** | **chr 19: 59070495-59111168** | **LOC100131691** | **2** |
| **2033** | **chr 19: 59070495-59111168** | **UBE2M** | **2** |
| **2034** | **chr 2: 946553-1371385** | **SNTG2** | **1** |
| **2035** | **chr 2: 1792884-2335032** | **MYT1L** | **2** |
| **2035** | **chr 2: 1792884-2335032** | **LOC730811** | **2** |
| **2036** | **chr 2: 2898819-3129798** | **none** | **1** |
| **2037** | **chr 2: 3192695-3381653** | **TSSC1** | **3** |
| **2038** | **chr 2: 3383445-3488865** | **TRAPPC12** | **1** |
| **2039** | **chr 2: 3642425-3692048** | **COLEC11** | **3** |
| **2040** | **chr 2: 6968644-6980595** | **CMPK2** | **1** |
| **2041** | **chr 2: 8026900-8039786** | **LOC339788** | **1** |
| **2042** | **chr 2: 8062555-8523751** | **LOC339788** | **3** |
| **2042** | **chr 2: 8062555-8523751** | **LINC00299** | **3** |
| **2043** | **chr 2: 8683721-8723922** | **none** | **1** |
| **2044** | **chr 2: 9346893-9545812** | **ASAP2** | **1** |
| **2045** | **chr 2: 9628614-9695921** | **ADAM17** | **1** |
| **2046** | **chr 2: 9983482-10074545** | **TAF1B** | **1** |
| **2047** | **chr 2: 11319886-11488456** | **ROCK2** | **1** |
| **2048** | **chr 2: 11988747-12718474** | **MIR4262** | **3** |
| **2049** | **chr 2: 16909390-16948822** | **none** | **2** |
| **2050** | **chr 2: 17720392-17838285** | **VSNL1** | **2** |
| **2051** | **chr 2: 18059113-18542882** | **KCNS3** | **2** |
| **2052** | **chr 2: 19167728-19546509** | **MIR4757** | **2** |
| **2053** | **chr 2: 19911475-19917337** | **none** | **1** |
| **2054** | **chr 2: 21444046-22193831** | **LOC645949** | **9** |
| **2055** | **chr 2: 22156207-22753977** | **none** | **2** |
| **2056** | **chr 2: 23570524-23574735** | **KLHL29** | **1** |
| **2057** | **chr 2: 24299395-24423718** | **SF3B14** | **1** |
| **2057** | **chr 2: 24299395-24423718** | **TP53I3** | **1** |
| **2058** | **chr 2: 24388752-24397874** | **FAM228B** | **2** |
| **2058** | **chr 2: 24388752-24397874** | **FAM228A** | **2** |
| **2059** | **chr 2: 24425732-24583583** | **ITSN2** | **1** |
| **2060** | **chr 2: 24714782-24993571** | **NCOA1** | **1** |
| **2061** | **chr 2: 25455844-25565459** | **DNMT3A** | **1** |
| **2062** | **chr 2: 26149470-26205618** | **KIF3C** | **1** |
| **2063** | **chr 2: 26785449-26802400** | **C2ORF70** | **1** |
| **2064** | **chr 2: 27498288-27504367** | **TRIM54** | **1** |
| **2064** | **chr 2: 27498288-27504367** | **DNAJC5G** | **1** |
| **2065** | **chr 2: 27532359-27548547** | **MPV17** | **1** |
| **2066** | **chr 2: 27548715-27579868** | **GTF3C2** | **1** |
| **2066** | **chr 2: 27548715-27579868** | **LOC100505624** | **1** |
| **2067** | **chr 2: 27650656-27665126** | **KRTCAP3** | **3** |
| **2067** | **chr 2: 27650656-27665126** | **NRBP1** | **3** |
| **2068** | **chr 2: 27719708-27746554** | **GCKR** | **1** |
| **2069** | **chr 2: 27805896-27874375** | **ZNF512** | **1** |
| **2069** | **chr 2: 27805896-27874375** | **CCDC121** | **1** |
| **2070** | **chr 2: 27994583-28561768** | **RBKS** | **1** |
| **2070** | **chr 2: 27994583-28561768** | **MRPL33** | **1** |
| **2071** | **chr 2: 29179476-29284239** | **FAM179A** | **2** |
| **2072** | **chr 2: 29415639-30144432** | **ALK** | **2** |
| **2073** | **chr 2: 30369806-30383399** | **YPEL5** | **1** |
| **2074** | **chr 2: 30454396-30546596** | **LBH** | **2** |
| **2075** | **chr 2: 30670091-30867091** | **LCLAT1** | **1** |
| **2076** | **chr 2: 30945636-31043408** | **CAPN13** | **3** |
| **2077** | **chr 2: 31133332-31378068** | **GALNT14** | **3** |
| **2078** | **chr 2: 31747549-31806136** | **SRD5A2** | **1** |
| **2079** | **chr 2: 31752011-31788534** | **SRD5A2** | **1** |
| **2080** | **chr 2: 32090128-32264881** | **DPY30** | **2** |
| **2080** | **chr 2: 32090128-32264881** | **MEMO1** | **2** |
| **2081** | **chr 2: 32390932-32449448** | **SLC30A6** | **1** |
| **2082** | **chr 2: 32449521-32490923** | **NLRC4** | **1** |
| **2083** | **chr 2: 32853098-33046118** | **TTC27** | **2** |
| **2083** | **chr 2: 32853098-33046118** | **MIR4765** | **2** |
| **2084** | **chr 2: 33661390-33789817** | **RASGRP3** | **1** |
| **2085** | **chr 2: 33931952-34522820** | **MYADML** | **1** |
| **2086** | **chr 2: 34608854-34860407** | **none** | **2** |
| **2087** | **chr 2: 35024916-35410755** | **none** | **4** |
| **2088** | **chr 2: 36583068-36778278** | **LOC100288911** | **2** |
| **2088** | **chr 2: 36583068-36778278** | **CRIM1** | **2** |
| **2089** | **chr 2: 36758947-36873230** | **CRIM1** | **6** |
| **2089** | **chr 2: 36758947-36873230** | **FEZ2** | **6** |
| **2090** | **chr 2: 36923832-37041935** | **VIT** | **1** |
| **2091** | **chr 2: 38053389-38103417** | **RMDN2** | **1** |
| **2092** | **chr 2: 38430504-38466732** | **CYP1B1-AS1** | **1** |
| **2093** | **chr 2: 38522021-38604427** | **ATL2** | **1** |
| **2094** | **chr 2: 38893051-38968379** | **GALM** | **2** |
| **2095** | **chr 2: 39663777-39945103** | **MAP4K3** | **2** |
| **2095** | **chr 2: 39663777-39945103** | **LOC728730** | **2** |
| **2096** | **chr 2: 40013592-40482349** | **SLC8A1** | **2** |
| **2096** | **chr 2: 40013592-40482349** | **SLC8A1-AS1** | **2** |
| **2097** | **chr 2: 40324409-40838193** | **SLC8A1** | **2** |
| **2097** | **chr 2: 40324409-40838193** | **SLC8A1-AS1** | **2** |
| **2098** | **chr 2: 40973626-40994592** | **none** | **2** |
| **2099** | **chr 2: 42994228-43019733** | **HAAO** | **1** |
| **2100** | **chr 2: 43393799-43823185** | **LOC100129726** | **5** |
| **2100** | **chr 2: 43393799-43823185** | **ZFP36L2** | **5** |
| **2101** | **chr 2: 45392008-45550434** | **UNQ6975** | **1** |
| **2102** | **chr 2: 45878483-46415129** | **PRKCE** | **4** |
| **2103** | **chr 2: 48132816-48134949** | **FBXO11** | **1** |
| **2104** | **chr 2: 49189295-49381676** | **FSHR** | **2** |
| **2105** | **chr 2: 50145642-51259674** | **NRXN1** | **3** |
| **2106** | **chr 2: 51259738-52635055** | **NRXN1** | **1** |
| **2107** | **chr 2: 54091203-54197977** | **PSME4** | **1** |
| **2108** | **chr 2: 54197974-54532437** | **PSME4** | **1** |
| **2108** | **chr 2: 54197974-54532437** | **ACYP2** | **1** |
| **2109** | **chr 2: 55463730-55496483** | **MTIF2** | **1** |
| **2110** | **chr 2: 56179292-56613308** | **MIR217** | **1** |
| **2110** | **chr 2: 56179292-56613308** | **MIR216A** | **1** |
| **2111** | **chr 2: 56190325-56274461** | **MIR217** | **1** |
| **2111** | **chr 2: 56190325-56274461** | **MIR216A** | **1** |
| **2112** | **chr 2: 58654933-59290901** | **FLJ30838** | **3** |
| **2113** | **chr 2: 59445814-60327335** | **none** | **7** |
| **2114** | **chr 2: 60678301-60780702** | **BCL11A** | **1** |
| **2115** | **chr 2: 61167356-61245394** | **PUS10** | **1** |
| **2115** | **chr 2: 61167356-61245394** | **PEX13** | **1** |
| **2116** | **chr 2: 62817389-62889789** | **EHBP1** | **1** |
| **2117** | **chr 2: 62900985-63273622** | **EHBP1** | **1** |
| **2118** | **chr 2: 63348517-64054977** | **WDPCP** | **1** |
| **2118** | **chr 2: 63348517-64054977** | **MDH1** | **1** |
| **2119** | **chr 2: 64858754-64978139** | **SERTAD2** | **2** |
| **2120** | **chr 2: 65537984-65659771** | **SPRED2** | **1** |
| **2121** | **chr 2: 65663844-66311773** | **SPRED2** | **6** |
| **2122** | **chr 2: 66660583-66801001** | **MEIS1** | **1** |
| **2122** | **chr 2: 66660583-66801001** | **MEIS1-AS3** | **1** |
| **2123** | **chr 2: 66801161-66957289** | **MEIS1** | **1** |
| **2124** | **chr 2: 67313577-67523207** | **LOC644838** | **2** |
| **2125** | **chr 2: 67350488-67442451** | **LOC644838** | **1** |
| **2126** | **chr 2: 69240309-69476459** | **ANTXR1** | **2** |
| **2127** | **chr 2: 69871556-70053596** | **AAK1** | **2** |
| **2128** | **chr 2: 70377011-70475792** | **TIA1** | **1** |
| **2128** | **chr 2: 70377011-70475792** | **C2ORF42** | **1** |
| **2129** | **chr 2: 70674411-70781325** | **TGFA** | **2** |
| **2130** | **chr 2: 70834749-70995357** | **ADD2** | **1** |
| **2131** | **chr 2: 71163011-71192536** | **ATP6V1B1** | **1** |
| **2132** | **chr 2: 71166447-71222466** | **ATP6V1B1** | **1** |
| **2132** | **chr 2: 71166447-71222466** | **ANKRD53** | **1** |
| **2133** | **chr 2: 71680851-71913898** | **DYSF** | **1** |
| **2134** | **chr 2: 72403112-73053170** | **EXOC6B** | **1** |
| **2135** | **chr 2: 73169164-73383849** | **SFXN5** | **6** |
| **2135** | **chr 2: 73169164-73383849** | **RAB11FIP5** | **6** |
| **2136** | **chr 2: 73481809-73511559** | **FBXO41** | **2** |
| **2137** | **chr 2: 73872045-73912703** | **ALMS1P** | **1** |
| **2138** | **chr 2: 74438730-74619214** | **SLC4A5** | **1** |
| **2138** | **chr 2: 74438730-74619214** | **MTHFD2** | **1** |
| **2139** | **chr 2: 74753771-74757066** | **DQX1** | **1** |
| **2139** | **chr 2: 74753771-74757066** | **AUP1** | **1** |
| **2140** | **chr 2: 76974844-77820445** | **LRRTM4** | **1** |
| **2141** | **chr 2: 79385499-80875905** | **REG3A** | **7** |
| **2142** | **chr 2: 80926512-81097315** | **none** | **1** |
| **2143** | **chr 2: 85048773-85134132** | **TRABD2A** | **1** |
| **2143** | **chr 2: 85048773-85134132** | **TMSB10** | **1** |
| **2144** | **chr 2: 85360532-85537511** | **TCF7L1** | **6** |
| **2145** | **chr 2: 85581516-85618875** | **ELMOD3** | **1** |
| **2145** | **chr 2: 85581516-85618875** | **RETSAT** | **1** |
| **2146** | **chr 2: 85645843-85664152** | **SH2D6** | **3** |
| **2147** | **chr 2: 86441115-86565206** | **REEP1** | **1** |
| **2148** | **chr 2: 86667769-86719839** | **KDM3A** | **1** |
| **2149** | **chr 2: 87042461-87089047** | **CD8B** | **1** |
| **2150** | **chr 2: 87754886-87906324** | **LINC00152** | **2** |
| **2151** | **chr 2: 88838237-88875128** | **EIF2AK3** | **1** |
| **2152** | **chr 2: 98372798-98612388** | **TMEM131** | **1** |
| **2153** | **chr 2: 98703578-98929762** | **VWA3B** | **6** |
| **2154** | **chr 2: 99235568-99347589** | **MGAT4A** | **1** |
| **2155** | **chr 2: 99757947-99939204** | **TSGA10** | **1** |
| **2156** | **chr 2: 99935444-99957165** | **TXNDC9** | **1** |
| **2156** | **chr 2: 99935444-99957165** | **EIF5B** | **1** |
| **2157** | **chr 2: 99953815-100017789** | **TXNDC9** | **1** |
| **2157** | **chr 2: 99953815-100017789** | **EIF5B** | **1** |
| **2158** | **chr 2: 100162322-100759201** | **AFF3** | **3** |
| **2159** | **chr 2: 101008326-101034118** | **CHST10** | **1** |
| **2160** | **chr 2: 101436613-101613291** | **NPAS2** | **3** |
| **2161** | **chr 2: 101624078-101869328** | **TBC1D8** | **2** |
| **2161** | **chr 2: 101624078-101869328** | **RPL31** | **2** |
| **2162** | **chr 2: 102313311-102511150** | **MAP4K4** | **2** |
| **2163** | **chr 2: 102681003-102796334** | **IL1R1** | **1** |
| **2164** | **chr 2: 102803432-102856462** | **IL1RL2** | **1** |
| **2165** | **chr 2: 103583865-103600887** | **none** | **1** |
| **2166** | **chr 2: 103726216-103792718** | **none** | **1** |
| **2167** | **chr 2: 104490767-104694236** | **none** | **1** |
| **2168** | **chr 2: 105469742-105542510** | **POU3F3** | **2** |
| **2169** | **chr 2: 105552868-105654954** | **MRPS9** | **1** |
| **2170** | **chr 2: 105974168-106054970** | **FHL2** | **1** |
| **2171** | **chr 2: 106361353-106510730** | **NCK2** | **4** |
| **2172** | **chr 2: 106462989-106473633** | **NCK2** | **1** |
| **2173** | **chr 2: 106709758-106810795** | **UXS1** | **3** |
| **2174** | **chr 2: 108002087-108159105** | **none** | **1** |
| **2175** | **chr 2: 109745803-110262207** | **SH3RF3** | **2** |
| **2175** | **chr 2: 109745803-110262207** | **SH3RF3-AS1** | **2** |
| **2176** | **chr 2: 110550334-110615272** | **RGPD6** | **1** |
| **2176** | **chr 2: 110550334-110615272** | **RGPD5** | **1** |
| **2177** | **chr 2: 111271388-111334762** | **RGPD6** | **1** |
| **2177** | **chr 2: 111271388-111334762** | **RGPD5** | **1** |
| **2178** | **chr 2: 111490149-111875799** | **ACOXL** | **4** |
| **2179** | **chr 2: 111953926-112252677** | **MIR4435-2** | **2** |
| **2179** | **chr 2: 111953926-112252677** | **MIR4435-1** | **2** |
| **2180** | **chr 2: 113239730-113299316** | **POLR1B** | **1** |
| **2180** | **chr 2: 113239730-113299316** | **TTL** | **1** |
| **2181** | **chr 2: 113341816-113372008** | **CHCHD5** | **2** |
| **2182** | **chr 2: 113914901-113966973** | **PSD4** | **1** |
| **2183** | **chr 2: 115199875-116603328** | **DPP10** | **4** |
| **2184** | **chr 2: 119599746-119605254** | **EN1** | **1** |
| **2185** | **chr 2: 120517206-120741394** | **PTPN4** | **1** |
| **2186** | **chr 2: 121493198-121750229** | **GLI2** | **4** |
| **2187** | **chr 2: 121974162-122042783** | **TFCP2L1** | **2** |
| **2188** | **chr 2: 122095351-122407163** | **RNU4ATAC** | **1** |
| **2188** | **chr 2: 122095351-122407163** | **CLASP1** | **1** |
| **2189** | **chr 2: 122407225-122485784** | **CLASP1** | **1** |
| **2190** | **chr 2: 124782863-125672864** | **CNTNAP5** | **3** |
| **2191** | **chr 2: 127656471-127659673** | **none** | **1** |
| **2192** | **chr 2: 127941695-127977654** | **CYP27C1** | **2** |
| **2193** | **chr 2: 128293377-128395304** | **MYO7B** | **1** |
| **2194** | **chr 2: 128395955-128439360** | **LIMS2** | **3** |
| **2195** | **chr 2: 128403438-128410213** | **LIMS2** | **2** |
| **2196** | **chr 2: 128994289-129076151** | **HS6ST1** | **1** |
| **2197** | **chr 2: 131513007-131525707** | **AMER3** | **1** |
| **2198** | **chr 2: 131588550-131594567** | **none** | **2** |
| **2199** | **chr 2: 133174146-133404132** | **GPR39** | **1** |
| **2199** | **chr 2: 133174146-133404132** | **LYPD1** | **1** |
| **2200** | **chr 2: 133429373-134326034** | **LYPD1** | **2** |
| **2201** | **chr 2: 134877553-135212192** | **MIR3679** | **1** |
| **2201** | **chr 2: 134877553-135212192** | **MGAT5** | **1** |
| **2202** | **chr 2: 135213329-135476570** | **TMEM163** | **2** |
| **2203** | **chr 2: 135894485-136288806** | **ZRANB3** | **2** |
| **2203** | **chr 2: 135894485-136288806** | **RAB3GAP1** | **2** |
| **2204** | **chr 2: 136545409-136594750** | **LCT** | **1** |
| **2204** | **chr 2: 136545409-136594750** | **LOC100507600** | **1** |
| **2205** | **chr 2: 136664246-136743670** | **DARS** | **1** |
| **2206** | **chr 2: 137523114-138435287** | **THSD7B** | **8** |
| **2207** | **chr 2: 140988991-142895399** | **LRP1B** | **9** |
| **2208** | **chr 2: 143848930-144525921** | **ARHGAP15** | **1** |
| **2209** | **chr 2: 144524188-144533642** | **ARHGAP15** | **1** |
| **2210** | **chr 2: 144694639-144721723** | **GTDC1** | **1** |
| **2211** | **chr 2: 144695634-145090135** | **GTDC1** | **2** |
| **2212** | **chr 2: 145141647-145282147** | **ZEB2** | **2** |
| **2213** | **chr 2: 145275663-145279058** | **ZEB2** | **1** |
| **2214** | **chr 2: 148778579-149275805** | **ORC4** | **1** |
| **2215** | **chr 2: 149894620-150071776** | **LYPD6B** | **3** |
| **2216** | **chr 2: 151324708-151395525** | **RND3** | **1** |
| **2217** | **chr 2: 152214105-152236560** | **MIR4773-1** | **1** |
| **2217** | **chr 2: 152214105-152236560** | **TNFAIP6** | **1** |
| **2218** | **chr 2: 152341849-152591001** | **NEB** | **1** |
| **2219** | **chr 2: 154277190-154449801** | **RPRM** | **1** |
| **2220** | **chr 2: 154728425-155310361** | **GALNT13** | **2** |
| **2220** | **chr 2: 154728425-155310361** | **LOC100144595** | **2** |
| **2221** | **chr 2: 155554810-155714863** | **KCNJ3** | **2** |
| **2222** | **chr 2: 158383278-158485517** | **ACVR1C** | **3** |
| **2223** | **chr 2: 158733213-158992666** | **UPP2** | **2** |
| **2223** | **chr 2: 158733213-158992666** | **ACVR1** | **2** |
| **2224** | **chr 2: 159313475-159539391** | **CCDC148** | **1** |
| **2225** | **chr 2: 159651828-159719797** | **DAPL1** | **1** |
| **2226** | **chr 2: 159825145-160089170** | **TANC1** | **1** |
| **2227** | **chr 2: 160956176-161128399** | **ITGB6** | **1** |
| **2228** | **chr 2: 161952741-162016735** | **TANK** | **2** |
| **2229** | **chr 2: 162164548-162268228** | **PSMD14** | **2** |
| **2230** | **chr 2: 162848750-162931052** | **DPP4** | **1** |
| **2231** | **chr 2: 163123588-163175213** | **IFIH1** | **2** |
| **2232** | **chr 2: 164606082-165208733** | **FIGN** | **2** |
| **2233** | **chr 2: 165349321-165478358** | **GRB14** | **1** |
| **2234** | **chr 2: 165510133-165700189** | **SNORA70F** | **1** |
| **2234** | **chr 2: 165510133-165700189** | **COBLL1** | **1** |
| **2235** | **chr 2: 165848493-165947803** | **SCN3A** | **2** |
| **2236** | **chr 2: 166326156-166545917** | **CSRNP3** | **3** |
| **2237** | **chr 2: 166713984-166810353** | **LOC100506124** | **1** |
| **2237** | **chr 2: 166713984-166810353** | **TTC21B** | **1** |
| **2238** | **chr 2: 166790366-166806401** | **TTC21B** | **1** |
| **2238** | **chr 2: 166790366-166806401** | **LOC100506134** | **1** |
| **2239** | **chr 2: 168149680-168414843** | **XIRP2** | **1** |
| **2240** | **chr 2: 168810529-169104651** | **STK39** | **1** |
| **2241** | **chr 2: 169312371-169631644** | **CERS6** | **1** |
| **2241** | **chr 2: 169312371-169631644** | **MIR4774** | **1** |
| **2242** | **chr 2: 169779447-169887832** | **ABCB11** | **1** |
| **2243** | **chr 2: 170335687-170382772** | **KLHL41** | **1** |
| **2243** | **chr 2: 170335687-170382772** | **BBS5** | **1** |
| **2244** | **chr 2: 170666590-170681441** | **METTL5** | **1** |
| **2244** | **chr 2: 170666590-170681441** | **SSB** | **1** |
| **2245** | **chr 2: 171034654-171511681** | **MYO3B** | **2** |
| **2246** | **chr 2: 171669722-171717661** | **GAD1** | **1** |
| **2247** | **chr 2: 171847332-172087824** | **TLK1** | **1** |
| **2248** | **chr 2: 172378756-172414643** | **CYBRD1** | **1** |
| **2249** | **chr 2: 172864489-172947158** | **METAP1D** | **1** |
| **2250** | **chr 2: 173292081-173371181** | **ITGA6** | **1** |
| **2251** | **chr 2: 173420100-173489823** | **PDK1** | **1** |
| **2252** | **chr 2: 173600001-173917621** | **RAPGEF4** | **3** |
| **2252** | **chr 2: 173600001-173917621** | **RAPGEF4-AS1** | **3** |
| **2253** | **chr 2: 175190754-175195371** | **LOC285084** | **1** |
| **2254** | **chr 2: 175296965-175351822** | **GPR155** | **1** |
| **2255** | **chr 2: 176121977-176319368** | **none** | **2** |
| **2256** | **chr 2: 176968943-176984670** | **HOXD10** | **1** |
| **2256** | **chr 2: 176968943-176984670** | **HOXD11** | **1** |
| **2257** | **chr 2: 177588995-177684038** | **none** | **1** |
| **2258** | **chr 2: 177855235-178077390** | **HNRNPA3** | **1** |
| **2258** | **chr 2: 177855235-178077390** | **MIR4444-1** | **1** |
| **2259** | **chr 2: 178257371-178408564** | **AGPS** | **1** |
| **2259** | **chr 2: 178257371-178408564** | **LOC100130691** | **1** |
| **2260** | **chr 2: 178487979-178973066** | **PDE11A** | **2** |
| **2261** | **chr 2: 179059207-179264160** | **OSBPL6** | **2** |
| **2262** | **chr 2: 179390715-179914813** | **MIR548N** | **1** |
| **2263** | **chr 2: 180306708-180726232** | **ZNF385B** | **3** |
| **2264** | **chr 2: 181436438-181557181** | **none** | **2** |
| **2265** | **chr 2: 183773842-183903586** | **NCKAP1** | **1** |
| **2266** | **chr 2: 188328956-188430487** | **TFPI** | **1** |
| **2267** | **chr 2: 188577542-189152418** | **GULP1** | **1** |
| **2268** | **chr 2: 189156395-189460653** | **GULP1** | **2** |
| **2269** | **chr 2: 189463517-189704171** | **DIRC1** | **1** |
| **2270** | **chr 2: 190539015-190625919** | **ANKAR** | **1** |
| **2270** | **chr 2: 190539015-190625919** | **OSGEPL1** | **1** |
| **2271** | **chr 2: 192109910-192290115** | **MYO1B** | **1** |
| **2272** | **chr 2: 192711264-192908909** | **TMEFF2** | **1** |
| **2272** | **chr 2: 192711264-192908909** | **SDPR** | **1** |
| **2273** | **chr 2: 192813768-193060435** | **TMEFF2** | **2** |
| **2274** | **chr 2: 196440700-196602426** | **SLC39A10** | **4** |
| **2275** | **chr 2: 196602426-196933536** | **DNAH7** | **4** |
| **2276** | **chr 2: 197059093-197458416** | **HECW2** | **3** |
| **2277** | **chr 2: 198669425-199437305** | **PLCL1** | **3** |
| **2278** | **chr 2: 199417918-199637080** | **none** | **1** |
| **2279** | **chr 2: 200134222-200335989** | **SATB2** | **1** |
| **2280** | **chr 2: 201170603-201346986** | **SPATS2L** | **1** |
| **2281** | **chr 2: 201838440-201936394** | **NDUFB3** | **1** |
| **2281** | **chr 2: 201838440-201936394** | **FAM126B** | **1** |
| **2282** | **chr 2: 202047603-202094129** | **CASP10** | **1** |
| **2283** | **chr 2: 202241929-202316302** | **TRAK2** | **1** |
| **2283** | **chr 2: 202241929-202316302** | **STRADB** | **1** |
| **2284** | **chr 2: 202252580-202345569** | **TRAK2** | **1** |
| **2284** | **chr 2: 202252580-202345569** | **STRADB** | **1** |
| **2285** | **chr 2: 202937977-203062585** | **SUMO1** | **1** |
| **2285** | **chr 2: 202937977-203062585** | **FZD7** | **1** |
| **2286** | **chr 2: 203879601-204091101** | **NBEAL1** | **1** |
| **2287** | **chr 2: 204192941-204312446** | **ABI2** | **2** |
| **2287** | **chr 2: 204192941-204312446** | **RAPH1** | **2** |
| **2288** | **chr 2: 204259067-204400133** | **ABI2** | **1** |
| **2288** | **chr 2: 204259067-204400133** | **RAPH1** | **1** |
| **2289** | **chr 2: 205410515-206484886** | **PARD3B** | **3** |
| **2290** | **chr 2: 206546713-206662857** | **NRP2** | **1** |
| **2291** | **chr 2: 208104587-208394519** | **CREB1** | **2** |
| **2292** | **chr 2: 210288781-210598842** | **MAP2** | **1** |
| **2293** | **chr 2: 211154873-211179914** | **MYL1** | **1** |
| **2294** | **chr 2: 211189482-211333970** | **LANCL1** | **1** |
| **2295** | **chr 2: 212240445-213403565** | **MIR548F2** | **1** |
| **2295** | **chr 2: 212240445-213403565** | **ERBB4** | **1** |
| **2296** | **chr 2: 213446080-214017151** | **MIR4776-2** | **3** |
| **2296** | **chr 2: 213446080-214017151** | **MIR4776-1** | **3** |
| **2297** | **chr 2: 214149112-215275225** | **LOC100130451** | **5** |
| **2297** | **chr 2: 214149112-215275225** | **SPAG16** | **5** |
| **2298** | **chr 2: 215674952-215827998** | **ABCA12** | **2** |
| **2298** | **chr 2: 215674952-215827998** | **BARD1** | **2** |
| **2299** | **chr 2: 216476285-216708445** | **LINC00607** | **1** |
| **2300** | **chr 2: 217250010-217277419** | **SMARCAL1** | **1** |
| **2301** | **chr 2: 217277136-217347776** | **SMARCAL1** | **1** |
| **2302** | **chr 2: 217362911-217443903** | **RPL37A** | **1** |
| **2303** | **chr 2: 217559186-217858802** | **TNP1** | **5** |
| **2303** | **chr 2: 217559186-217858802** | **IGFBP5** | **5** |
| **2304** | **chr 2: 218147461-218200843** | **DIRC3** | **1** |
| **2305** | **chr 2: 218148741-218621316** | **DIRC3** | **4** |
| **2306** | **chr 2: 218664511-218867718** | **TNS1** | **2** |
| **2307** | **chr 2: 219128849-219134980** | **PNKD** | **1** |
| **2307** | **chr 2: 219128849-219134980** | **AAMP** | **1** |
| **2308** | **chr 2: 219262978-219270664** | **CTDSP1** | **1** |
| **2309** | **chr 2: 219745084-219764303** | **WNT10A** | **1** |
| **2310** | **chr 2: 219867567-219906249** | **CCDC108** | **1** |
| **2310** | **chr 2: 219867567-219906249** | **LOC100129175** | **1** |
| **2311** | **chr 2: 220036618-220042828** | **CNPPD1** | **1** |
| **2311** | **chr 2: 220036618-220042828** | **FAM134A** | **1** |
| **2312** | **chr 2: 220346794-220381599** | **SPEG** | **1** |
| **2313** | **chr 2: 220363588-220371710** | **GMPPA** | **1** |
| **2314** | **chr 2: 220403668-220408509** | **CHPF** | **1** |
| **2315** | **chr 2: 220970376-221569286** | **none** | **6** |
| **2316** | **chr 2: 224616402-224702744** | **AP1S3** | **3** |
| **2317** | **chr 2: 224839828-224904036** | **SERPINE2** | **1** |
| **2318** | **chr 2: 225334866-225450110** | **CUL3** | **1** |
| **2319** | **chr 2: 226265363-226518734** | **NYAP2** | **3** |
| **2320** | **chr 2: 227044759-227050087** | **LOC646736** | **5** |
| **2321** | **chr 2: 227867426-228028829** | **COL4A4** | **1** |
| **2321** | **chr 2: 227867426-228028829** | **COL4A3** | **1** |
| **2322** | **chr 2: 228029280-228179508** | **COL4A4** | **2** |
| **2322** | **chr 2: 228029280-228179508** | **COL4A3** | **2** |
| **2323** | **chr 2: 228336847-228425930** | **MIR5703** | **1** |
| **2323** | **chr 2: 228336847-228425930** | **AGFG1** | **1** |
| **2324** | **chr 2: 228735769-228789060** | **DAW1** | **1** |
| **2325** | **chr 2: 229715241-230136001** | **PID1** | **1** |
| **2326** | **chr 2: 230222344-230579274** | **DNER** | **5** |
| **2327** | **chr 2: 230899697-230933715** | **SLC16A14** | **1** |
| **2328** | **chr 2: 232825954-233209060** | **DIS3L2** | **1** |
| **2329** | **chr 2: 233470766-233547491** | **EFHD1** | **1** |
| **2330** | **chr 2: 233924676-234116549** | **INPP5D** | **2** |
| **2331** | **chr 2: 234216461-234255701** | **SAG** | **1** |
| **2332** | **chr 2: 234826042-234928166** | **TRPM8** | **1** |
| **2333** | **chr 2: 235860616-235964358** | **SH3BP4** | **1** |
| **2334** | **chr 2: 236402732-237040444** | **AGAP1** | **4** |
| **2335** | **chr 2: 238232645-238323018** | **COL6A3** | **1** |
| **2336** | **chr 2: 239140324-239164274** | **LOC643387** | **1** |
| **2336** | **chr 2: 239140324-239164274** | **LOC151174** | **1** |
| **2337** | **chr 2: 239969863-240323348** | **MIR4440** | **1** |
| **2337** | **chr 2: 239969863-240323348** | **HDAC4** | **1** |
| **2338** | **chr 2: 240684553-240724577** | **LOC150935** | **2** |
| **2339** | **chr 2: 240831866-240964819** | **MIR4786** | **3** |
| **2339** | **chr 2: 240831866-240964819** | **NDUFA10** | **3** |
| **2340** | **chr 2: 241375087-241407493** | **GPC1** | **1** |
| **2340** | **chr 2: 241375087-241407493** | **PP14571** | **1** |
| **2341** | **chr 2: 241418838-241508626** | **DUSP28** | **1** |
| **2341** | **chr 2: 241418838-241508626** | **ANKMY1** | **1** |
| **2342** | **chr 2: 241938254-242034983** | **SNED1** | **1** |
| **2342** | **chr 2: 241938254-242034983** | **MTERFD2** | **1** |
| **2343** | **chr 2: 241955015-242003533** | **SNED1** | **1** |
| **2344** | **chr 2: 242127923-242164792** | **ANO7** | **1** |
| **2345** | **chr 2: 242166678-242256476** | **HDLBP** | **1** |
| **2346** | **chr 2: 242295657-242434256** | **STK25** | **1** |
| **2346** | **chr 2: 242295657-242434256** | **FARP2** | **1** |
| **2347** | **chr 2: 242523819-242576864** | **THAP4** | **1** |
| **2348** | **chr 2: 242823513-243020873** | **CXXC11** | **1** |
| **2348** | **chr 2: 242823513-243020873** | **LOC728323** | **1** |
| **2349** | **chr 2: 242989845-243026289** | **LOC728323** | **1** |
| **2350** | **chr 20: 251503-271390** | **C20ORF96** | **2** |
| **2351** | **chr 20: 939094-982907** | **RSPO4** | **1** |
| **2352** | **chr 20: 1206699-1289972** | **RAD21L1** | **1** |
| **2352** | **chr 20: 1206699-1289972** | **SNPH** | **1** |
| **2353** | **chr 20: 1497404-1600707** | **SIRPD** | **2** |
| **2353** | **chr 20: 1497404-1600707** | **SIRPB1** | **2** |
| **2354** | **chr 20: 1875153-1920543** | **SIRPA** | **1** |
| **2355** | **chr 20: 1927891-1988163** | **PDYN** | **2** |
| **2356** | **chr 20: 2517252-2622430** | **TMC2** | **2** |
| **2357** | **chr 20: 2673523-2740753** | **EBF4** | **1** |
| **2358** | **chr 20: 2821348-3019722** | **PCED1A** | **1** |
| **2359** | **chr 20: 3827486-3849280** | **MAVS** | **1** |
| **2360** | **chr 20: 5917880-5931182** | **TRMT6** | **1** |
| **2360** | **chr 20: 5917880-5931182** | **MCM8** | **1** |
| **2361** | **chr 20: 6427369-6509106** | **none** | **1** |
| **2362** | **chr 20: 7050260-7127303** | **none** | **2** |
| **2363** | **chr 20: 8112823-8949003** | **PLCB1** | **2** |
| **2364** | **chr 20: 9049409-9461889** | **PLCB4** | **4** |
| **2365** | **chr 20: 9518035-9819689** | **PAK7** | **2** |
| **2366** | **chr 20: 10415950-10617477** | **SLX4IP** | **1** |
| **2367** | **chr 20: 10653575-10975572** | **JAG1** | **3** |
| **2368** | **chr 20: 11214817-11282173** | **LOC339593** | **3** |
| **2369** | **chr 20: 12845849-12933167** | **none** | **1** |
| **2370** | **chr 20: 12989626-13147411** | **SPTLC3** | **1** |
| **2371** | **chr 20: 13976014-16033842** | **SEL1L2** | **9** |
| **2371** | **chr 20: 13976014-16033842** | **MACROD2** | **9** |
| **2372** | **chr 20: 16252748-16554078** | **KIF16B** | **1** |
| **2373** | **chr 20: 18568536-18744561** | **DTD1** | **2** |
| **2374** | **chr 20: 18655038-18679353** | **DTD1** | **2** |
| **2375** | **chr 20: 19193289-19703581** | **LOC100130264** | **5** |
| **2375** | **chr 20: 19193289-19703581** | **SLC24A3** | **5** |
| **2376** | **chr 20: 20033157-20341346** | **C20ORF26** | **4** |
| **2376** | **chr 20: 20033157-20341346** | **CRNKL1** | **4** |
| **2377** | **chr 20: 20370195-20693131** | **RALGAPA2** | **1** |
| **2378** | **chr 20: 21106623-21227260** | **PLK1S1** | **1** |
| **2379** | **chr 20: 21129381-21198927** | **PLK1S1** | **2** |
| **2380** | **chr 20: 22561642-22566093** | **FOXA2** | **1** |
| **2381** | **chr 20: 24449834-24647252** | **SYNDIG1** | **7** |
| **2382** | **chr 20: 26084051-26094664** | **NCOR1P1** | **1** |
| **2383** | **chr 20: 29558487-29596406** | **FRG1B** | **1** |
| **2384** | **chr 20: 29956420-29961726** | **DEFB118** | **1** |
| **2385** | **chr 20: 30073580-30075377** | **LINC00028** | **1** |
| **2386** | **chr 20: 30639990-30689659** | **HCK** | **3** |
| **2387** | **chr 20: 31446728-31549006** | **MAPRE1** | **1** |
| **2387** | **chr 20: 31446728-31549006** | **SUN5** | **1** |
| **2388** | **chr 20: 31571578-31592239** | **SUN5** | **2** |
| **2389** | **chr 20: 31619453-31631853** | **BPIFB6** | **1** |
| **2390** | **chr 20: 31667449-31699557** | **BPIFB4** | **1** |
| **2391** | **chr 20: 31861285-31897684** | **BPIFB1** | **1** |
| **2392** | **chr 20: 32077880-32237842** | **CBFA2T2** | **1** |
| **2393** | **chr 20: 32581451-32696114** | **MIR4755** | **3** |
| **2393** | **chr 20: 32581451-32696114** | **RALY** | **3** |
| **2394** | **chr 20: 32782374-32857150** | **ASIP** | **1** |
| **2395** | **chr 20: 32822645-32899608** | **AHCY** | **3** |
| **2395** | **chr 20: 32822645-32899608** | **ASIP** | **3** |
| **2396** | **chr 20: 33563205-33590240** | **MIR499A** | **1** |
| **2396** | **chr 20: 33563205-33590240** | **MYH7B** | **1** |
| **2397** | **chr 20: 33703166-33872788** | **EDEM2** | **2** |
| **2397** | **chr 20: 33703166-33872788** | **PROCR** | **2** |
| **2398** | **chr 20: 34021144-34042568** | **CEP250** | **1** |
| **2398** | **chr 20: 34021144-34042568** | **GDF5** | **1** |
| **2399** | **chr 20: 34129769-34145405** | **ERGIC3** | **2** |
| **2400** | **chr 20: 34213952-34287281** | **CPNE1** | **4** |
| **2401** | **chr 20: 34359895-34538303** | **PHF20** | **1** |
| **2402** | **chr 20: 34894257-35157040** | **DLGAP4** | **3** |
| **2403** | **chr 20: 35136562-35232932** | **DLGAP4** | **1** |
| **2404** | **chr 20: 35201890-35240960** | **TGIF2** | **1** |
| **2405** | **chr 20: 35405844-35492089** | **SOGA1** | **1** |
| **2406** | **chr 20: 35518631-35580246** | **TLDC2** | **1** |
| **2406** | **chr 20: 35518631-35580246** | **SAMHD1** | **1** |
| **2407** | **chr 20: 36120873-36156333** | **BLCAP** | **2** |
| **2408** | **chr 20: 36756862-36794980** | **TGM2** | **1** |
| **2409** | **chr 20: 37230576-37279678** | **ARHGAP40** | **1** |
| **2410** | **chr 20: 37434347-37551667** | **PPP1R16B** | **3** |
| **2411** | **chr 20: 37590941-37668366** | **DHX35** | **1** |
| **2412** | **chr 20: 40701391-41818610** | **PTPRT** | **7** |
| **2413** | **chr 20: 42543503-42698256** | **TOX2** | **1** |
| **2414** | **chr 20: 42984339-43061485** | **HNF4A** | **1** |
| **2415** | **chr 20: 43160425-43252888** | **PKIG** | **3** |
| **2415** | **chr 20: 43160425-43252888** | **ADA** | **3** |
| **2416** | **chr 20: 43285091-43374662** | **WISP2** | **2** |
| **2416** | **chr 20: 43285091-43374662** | **LOC79015** | **2** |
| **2417** | **chr 20: 44179791-44207965** | **WFDC8** | **1** |
| **2418** | **chr 20: 44802371-44937137** | **CDH22** | **4** |
| **2419** | **chr 20: 45129700-45142198** | **ZNF334** | **1** |
| **2420** | **chr 20: 45186462-45304714** | **SLC13A3** | **1** |
| **2421** | **chr 20: 45523262-45817492** | **MIR3616** | **3** |
| **2421** | **chr 20: 45523262-45817492** | **EYA2** | **3** |
| **2422** | **chr 20: 45837858-45985567** | **LOC100131496** | **2** |
| **2422** | **chr 20: 45837858-45985567** | **ZMYND8** | **2** |
| **2423** | **chr 20: 46285091-46415360** | **NCOA3** | **1** |
| **2423** | **chr 20: 46285091-46415360** | **SULF2** | **1** |
| **2424** | **chr 20: 47538426-47653230** | **ARFGEF2** | **3** |
| **2425** | **chr 20: 48697660-48770335** | **UBE2V1** | **1** |
| **2426** | **chr 20: 48909256-48931459** | **LOC284751** | **1** |
| **2427** | **chr 20: 48927247-48937879** | **LOC284751** | **1** |
| **2428** | **chr 20: 49202644-49308065** | **FAM65C** | **1** |
| **2429** | **chr 20: 50003493-50179370** | **NFATC2** | **1** |
| **2430** | **chr 20: 50213052-50385173** | **ATP9A** | **3** |
| **2431** | **chr 20: 50668201-50820847** | **ZFP64** | **2** |
| **2432** | **chr 20: 51588945-52111869** | **TSHZ2** | **1** |
| **2433** | **chr 20: 51872517-52120853** | **TSHZ2** | **6** |
| **2434** | **chr 20: 52553315-52687304** | **MIR4756** | **1** |
| **2434** | **chr 20: 52553315-52687304** | **BCAS1** | **1** |
| **2435** | **chr 20: 52556698-52559047** | **BCAS1** | **1** |
| **2436** | **chr 20: 53092135-53267710** | **DOK5** | **1** |
| **2437** | **chr 20: 55959215-55968118** | **MIR5095** | **1** |
| **2438** | **chr 20: 56806187-56884495** | **PPP4R1L** | **1** |
| **2438** | **chr 20: 56806187-56884495** | **RAB22A** | **1** |
| **2439** | **chr 20: 57090434-57194944** | **APCDD1L-AS1** | **1** |
| **2439** | **chr 20: 57090434-57194944** | **APCDD1L** | **1** |
| **2440** | **chr 20: 57414772-57486247** | **GNAS-AS1** | **1** |
| **2440** | **chr 20: 57414772-57486247** | **GNAS** | **1** |
| **2441** | **chr 20: 57875481-57901047** | **EDN3** | **2** |
| **2442** | **chr 20: 57927249-57932829** | **EDN3** | **3** |
| **2443** | **chr 20: 58438617-58508710** | **SYCP2** | **2** |
| **2443** | **chr 20: 58438617-58508710** | **FAM217B** | **2** |
| **2444** | **chr 20: 58630979-58648008** | **C20ORF197** | **1** |
| **2445** | **chr 20: 58662894-59102516** | **MIR4533** | **4** |
| **2445** | **chr 20: 58662894-59102516** | **LOC284757** | **4** |
| **2446** | **chr 20: 59827481-60515673** | **CDH4** | **4** |
| **2447** | **chr 20: 60718821-60757540** | **PSMA7** | **1** |
| **2447** | **chr 20: 60718821-60757540** | **SS18L1** | **1** |
| **2448** | **chr 20: 61340188-61394123** | **NTSR1** | **1** |
| **2449** | **chr 20: 61447595-61472511** | **COL9A3** | **1** |
| **2449** | **chr 20: 61447595-61472511** | **TCFL5** | **1** |
| **2450** | **chr 20: 61509089-61569304** | **DIDO1** | **1** |
| **2451** | **chr 20: 61640734-61716423** | **LOC63930** | **1** |
| **2451** | **chr 20: 61640734-61716423** | **LINC00029** | **1** |
| **2452** | **chr 20: 61726844-61733671** | **HAR1B** | **1** |
| **2452** | **chr 20: 61726844-61733671** | **HAR1A** | **1** |
| **2453** | **chr 20: 62271060-62284780** | **STMN3** | **2** |
| **2454** | **chr 21: 17442841-17999716** | **LINC00478** | **2** |
| **2455** | **chr 21: 19641432-19858917** | **TMPRSS15** | **4** |
| **2456** | **chr 21: 20290251-20308177** | **none** | **1** |
| **2457** | **chr 21: 21265592-21272069** | **none** | **1** |
| **2458** | **chr 21: 22114907-22175534** | **LINC00320** | **1** |
| **2459** | **chr 21: 22370632-22915650** | **RNU6-67** | **1** |
| **2459** | **chr 21: 22370632-22915650** | **NCAM2** | **1** |
| **2460** | **chr 21: 26212863-26442219** | **LOC339622** | **1** |
| **2461** | **chr 21: 26541744-26706139** | **none** | **1** |
| **2462** | **chr 21: 28731203-28820898** | **MIR5009** | **2** |
| **2463** | **chr 21: 29488412-29600986** | **none** | **3** |
| **2464** | **chr 21: 29811666-30047170** | **LINC00161** | **1** |
| **2465** | **chr 21: 30449791-30548210** | **MAP3K7CL** | **1** |
| **2466** | **chr 21: 30565800-31003071** | **LINC00189** | **2** |
| **2466** | **chr 21: 30565800-31003071** | **BACH1** | **2** |
| **2467** | **chr 21: 30909253-31312351** | **GRIK1** | **2** |
| **2468** | **chr 21: 32490733-32932290** | **TIAM1** | **4** |
| **2469** | **chr 21: 33245627-33416946** | **HUNK** | **3** |
| **2470** | **chr 21: 33683328-33765335** | **MRAP** | **1** |
| **2471** | **chr 21: 33784313-33887707** | **EVA1C** | **3** |
| **2472** | **chr 21: 34285956-34443410** | **OLIG2** | **1** |
| **2472** | **chr 21: 34285956-34443410** | **OLIG1** | **1** |
| **2473** | **chr 21: 34931847-35288284** | **SON** | **2** |
| **2474** | **chr 21: 35014705-35272165** | **ITSN1** | **2** |
| **2474** | **chr 21: 35014705-35272165** | **CRYZL1** | **2** |
| **2475** | **chr 21: 35445523-35732332** | **MRPS6** | **2** |
| **2475** | **chr 21: 35445523-35732332** | **SLC5A3** | **2** |
| **2476** | **chr 21: 35818987-35884573** | **KCNE1** | **1** |
| **2477** | **chr 21: 35885439-35987441** | **RCAN1** | **1** |
| **2477** | **chr 21: 35885439-35987441** | **KCNE1** | **1** |
| **2478** | **chr 21: 36041687-36090525** | **CLIC6** | **1** |
| **2479** | **chr 21: 36160097-37376965** | **RUNX1** | **9** |
| **2480** | **chr 21: 37529079-37666572** | **CBR3-AS1** | **1** |
| **2481** | **chr 21: 38071432-38122218** | **SIM2** | **4** |
| **2482** | **chr 21: 38123188-38362536** | **HLCS** | **1** |
| **2483** | **chr 21: 38445525-38575413** | **PIGP** | **2** |
| **2484** | **chr 21: 38595720-38640262** | **DSCR3** | **2** |
| **2485** | **chr 21: 38979677-39288749** | **KCNJ6** | **1** |
| **2486** | **chr 21: 39323727-39493454** | **DSCR4** | **1** |
| **2486** | **chr 21: 39323727-39493454** | **DSCR8** | **1** |
| **2487** | **chr 21: 39493544-39679279** | **DSCR4** | **1** |
| **2487** | **chr 21: 39493544-39679279** | **DSCR8** | **1** |
| **2488** | **chr 21: 40360632-40378079** | **none** | **2** |
| **2489** | **chr 21: 40928368-41098012** | **C21ORF88** | **3** |
| **2490** | **chr 21: 41117333-41174023** | **IGSF5** | **1** |
| **2491** | **chr 21: 41382925-42219065** | **DSCAM** | **6** |
| **2491** | **chr 21: 41382925-42219065** | **MIR4760** | **6** |
| **2492** | **chr 21: 42539727-42654445** | **BACE2** | **1** |
| **2492** | **chr 21: 42539727-42654445** | **PLAC4** | **1** |
| **2493** | **chr 21: 42948061-42953246** | **none** | **1** |
| **2494** | **chr 21: 43218384-43299591** | **PRDM15** | **3** |
| **2495** | **chr 21: 43619798-43717354** | **ABCG1** | **1** |
| **2496** | **chr 21: 43916117-44001550** | **RSPH1** | **1** |
| **2496** | **chr 21: 43916117-44001550** | **SLC37A1** | **1** |
| **2497** | **chr 21: 45770045-45862964** | **TRPM2** | **1** |
| **2498** | **chr 21: 46352728-46359828** | **C21ORF67** | **1** |
| **2499** | **chr 21: 46825051-46933634** | **COL18A1** | **1** |
| **2499** | **chr 21: 46825051-46933634** | **COL18A1-AS2** | **1** |
| **2500** | **chr 21: 47063607-47362368** | **LOC100129027** | **1** |
| **2500** | **chr 21: 47063607-47362368** | **PCBP3** | **1** |
| **2501** | **chr 22: 17840836-18037850** | **CECR2** | **1** |
| **2502** | **chr 22: 18074901-18111584** | **ATP6V1E1** | **1** |
| **2503** | **chr 22: 18111620-18213388** | **ATP6V1E1** | **1** |
| **2503** | **chr 22: 18111620-18213388** | **BCL2L13** | **1** |
| **2504** | **chr 22: 18270414-18507325** | **MICAL3** | **1** |
| **2505** | **chr 22: 19863039-19929341** | **TXNRD2** | **1** |
| **2505** | **chr 22: 19863039-19929341** | **COMT** | **1** |
| **2506** | **chr 22: 20778873-20850170** | **SCARF2** | **1** |
| **2506** | **chr 22: 20778873-20850170** | **KLHL22** | **1** |
| **2507** | **chr 22: 21319395-21353327** | **AIFM3** | **1** |
| **2508** | **chr 22: 22108788-22221970** | **MAPK1** | **1** |
| **2509** | **chr 22: 23401592-23487208** | **RTDR1** | **1** |
| **2509** | **chr 22: 23401592-23487208** | **GNAZ** | **1** |
| **2510** | **chr 22: 23412539-23467224** | **RTDR1** | **1** |
| **2510** | **chr 22: 23412539-23467224** | **GNAZ** | **1** |
| **2511** | **chr 22: 23521890-23660224** | **BCR** | **1** |
| **2511** | **chr 22: 23521890-23660224** | **FBXW4P1** | **1** |
| **2512** | **chr 22: 24129149-24176703** | **DERL3** | **1** |
| **2512** | **chr 22: 24129149-24176703** | **SMARCB1** | **1** |
| **2513** | **chr 22: 24243618-24249255** | **MIF** | **1** |
| **2514** | **chr 22: 24385937-24401899** | **GSTTP2** | **1** |
| **2515** | **chr 22: 24615621-24641110** | **GGT5** | **1** |
| **2516** | **chr 22: 24863205-24924358** | **ADORA2A-AS1** | **1** |
| **2517** | **chr 22: 25348696-25593415** | **LOC100128531** | **7** |
| **2517** | **chr 22: 25348696-25593415** | **KIAA1671** | **7** |
| **2518** | **chr 22: 25960815-26125261** | **ADRBK2** | **1** |
| **2519** | **chr 22: 26138110-26427007** | **MYO18B** | **2** |
| **2520** | **chr 22: 26565439-26779562** | **SEZ6L** | **3** |
| **2521** | **chr 22: 27017927-27026636** | **CRYBA4** | **2** |
| **2522** | **chr 22: 27703443-27714499** | **none** | **2** |
| **2523** | **chr 22: 28374003-29075853** | **TTC28-AS1** | **2** |
| **2524** | **chr 22: 29601839-29655586** | **EMID1** | **1** |
| **2525** | **chr 22: 29832817-29874164** | **RFPL1** | **1** |
| **2525** | **chr 22: 29832817-29874164** | **RFPL1S** | **1** |
| **2526** | **chr 22: 30476162-30573064** | **HORMAD2** | **1** |
| **2527** | **chr 22: 30580632-30603098** | **HORMAD2** | **1** |
| **2527** | **chr 22: 30580632-30603098** | **LIF** | **1** |
| **2528** | **chr 22: 30681105-30723035** | **GATSL3** | **1** |
| **2528** | **chr 22: 30681105-30723035** | **TBC1D10A** | **1** |
| **2529** | **chr 22: 31089768-31303811** | **MIR3200** | **1** |
| **2529** | **chr 22: 31089768-31303811** | **OSBP2** | **1** |
| **2530** | **chr 22: 31460090-31500743** | **SMTN** | **1** |
| **2531** | **chr 22: 31677578-31688520** | **PIK3IP1** | **1** |
| **2532** | **chr 22: 32149943-32303012** | **DEPDC5** | **1** |
| **2533** | **chr 22: 32439018-32509016** | **SLC5A1** | **1** |
| **2534** | **chr 22: 32908538-33454358** | **SYN3** | **5** |
| **2535** | **chr 22: 33197686-33259030** | **SYN3** | **1** |
| **2536** | **chr 22: 33558211-34318829** | **LARGE** | **7** |
| **2536** | **chr 22: 33558211-34318829** | **MIR4764** | **7** |
| **2537** | **chr 22: 34413461-34584414** | **none** | **3** |
| **2538** | **chr 22: 35318598-35468259** | **ISX** | **1** |
| **2539** | **chr 22: 35515816-35627049** | **ISX** | **3** |
| **2539** | **chr 22: 35515816-35627049** | **HMGXB4** | **3** |
| **2540** | **chr 22: 36134782-36424473** | **RBFOX2** | **1** |
| **2541** | **chr 22: 36841444-36850991** | **TXN2** | **2** |
| **2542** | **chr 22: 36959967-37099603** | **CACNG2** | **1** |
| **2543** | **chr 22: 37461475-37505603** | **TMPRSS6** | **3** |
| **2544** | **chr 22: 37886399-37915549** | **CARD10** | **1** |
| **2545** | **chr 22: 38348613-38437922** | **C22ORF23** | **2** |
| **2546** | **chr 22: 38366692-38383429** | **SOX10** | **1** |
| **2547** | **chr 22: 39214456-39239987** | **NPTXR** | **1** |
| **2548** | **chr 22: 39317231-39320713** | **CBX6** | **1** |
| **2548** | **chr 22: 39317231-39320713** | **APOBEC3A** | **1** |
| **2549** | **chr 22: 39436608-39483748** | **APOBEC3F** | **1** |
| **2550** | **chr 22: 39966757-40085742** | **CACNA1I** | **1** |
| **2551** | **chr 22: 40297085-40369725** | **GRAP2** | **1** |
| **2552** | **chr 22: 41487789-41576081** | **EP300** | **1** |
| **2552** | **chr 22: 41487789-41576081** | **MIR1281** | **1** |
| **2553** | **chr 22: 41829495-41843027** | **TOB2** | **1** |
| **2554** | **chr 22: 42069933-42086508** | **NHP2L1** | **1** |
| **2554** | **chr 22: 42069933-42086508** | **C22ORF46** | **1** |
| **2555** | **chr 22: 42229108-42303312** | **SREBF2** | **1** |
| **2555** | **chr 22: 42229108-42303312** | **MIR33A** | **1** |
| **2556** | **chr 22: 42556018-42739622** | **LOC388906** | **1** |
| **2556** | **chr 22: 42556018-42739622** | **TCF20** | **1** |
| **2557** | **chr 22: 42665758-42671202** | **LOC388906** | **1** |
| **2558** | **chr 22: 43192507-43411151** | **ARFGAP3** | **2** |
| **2558** | **chr 22: 43192507-43411151** | **PACSIN2** | **2** |
| **2559** | **chr 22: 43807201-43903728** | **MPPED1** | **1** |
| **2560** | **chr 22: 43924623-44208217** | **EFCAB6-AS1** | **1** |
| **2560** | **chr 22: 43924623-44208217** | **EFCAB6** | **1** |
| **2561** | **chr 22: 45064592-45258665** | **PRR5** | **3** |
| **2562** | **chr 22: 45277041-45405880** | **PHF21B** | **1** |
| **2563** | **chr 22: 45559721-45583896** | **LOC100506714** | **1** |
| **2563** | **chr 22: 45559721-45583896** | **NUP50** | **1** |
| **2564** | **chr 22: 46271878-46283628** | **WNT7B** | **1** |
| **2565** | **chr 22: 46756730-46933067** | **CELSR1** | **2** |
| **2566** | **chr 22: 48016791-48027318** | **FLJ46257** | **1** |
| **2567** | **chr 22: 48885271-49246724** | **LOC284933** | **4** |
| **2567** | **chr 22: 48885271-49246724** | **FAM19A5** | **4** |
| **2568** | **chr 22: 50311814-50321188** | **CRELD2** | **1** |
| **2568** | **chr 22: 50311814-50321188** | **ALG12** | **1** |
| **2569** | **chr 3: 2004064-2029154** | **none** | **1** |
| **2570** | **chr 3: 2140496-3099645** | **CNTN4** | **4** |
| **2570** | **chr 3: 2140496-3099645** | **CNTN4-AS2** | **4** |
| **2571** | **chr 3: 3292370-3668980** | **none** | **2** |
| **2572** | **chr 3: 3742497-4508965** | **SETMAR** | **3** |
| **2572** | **chr 3: 3742497-4508965** | **LRRN1** | **3** |
| **2573** | **chr 3: 4535031-4889524** | **ITPR1** | **1** |
| **2573** | **chr 3: 4535031-4889524** | **EGOT** | **1** |
| **2574** | **chr 3: 6004528-6165807** | **none** | **2** |
| **2575** | **chr 3: 6532165-6777816** | **none** | **2** |
| **2576** | **chr 3: 6673375-6847136** | **none** | **1** |
| **2577** | **chr 3: 6811687-7783215** | **GRM7** | **1** |
| **2578** | **chr 3: 7994491-8653610** | **LMCD1** | **1** |
| **2578** | **chr 3: 7994491-8653610** | **LMCD1-AS1** | **1** |
| **2579** | **chr 3: 8661085-8786726** | **SSUH2** | **1** |
| **2580** | **chr 3: 8817087-9005457** | **RAD18** | **2** |
| **2581** | **chr 3: 9022274-9440263** | **THUMPD3** | **4** |
| **2581** | **chr 3: 9022274-9440263** | **SRGAP3** | **4** |
| **2582** | **chr 3: 9975505-9987097** | **CRELD1** | **1** |
| **2583** | **chr 3: 10289706-10335133** | **GHRLOS** | **1** |
| **2583** | **chr 3: 10289706-10335133** | **TATDN2** | **1** |
| **2584** | **chr 3: 10365706-10749716** | **MIR885** | **6** |
| **2584** | **chr 3: 10365706-10749716** | **ATP2B2** | **6** |
| **2585** | **chr 3: 10857884-10982419** | **SLC6A11** | **1** |
| **2586** | **chr 3: 11313994-11599139** | **ATG7** | **2** |
| **2586** | **chr 3: 11313994-11599139** | **VGLL4** | **2** |
| **2587** | **chr 3: 11597543-11766453** | **ATG7** | **2** |
| **2587** | **chr 3: 11597543-11766453** | **VGLL4** | **2** |
| **2588** | **chr 3: 12045875-12232900** | **SYN2** | **1** |
| **2589** | **chr 3: 12328866-12475855** | **PPARG** | **1** |
| **2590** | **chr 3: 12775023-12810956** | **TMEM40** | **3** |
| **2591** | **chr 3: 13357736-13461809** | **NUP210** | **1** |
| **2592** | **chr 3: 13573823-13679922** | **FBLN2** | **2** |
| **2593** | **chr 3: 13857754-13921618** | **WNT7A** | **2** |
| **2594** | **chr 3: 14219857-14242619** | **XPC** | **1** |
| **2594** | **chr 3: 14219857-14242619** | **LSM3** | **1** |
| **2595** | **chr 3: 14444075-14530857** | **SLC6A6** | **1** |
| **2596** | **chr 3: 14716605-14814541** | **C3ORF20** | **2** |
| **2597** | **chr 3: 14961853-14989931** | **FGD5** | **1** |
| **2597** | **chr 3: 14961853-14989931** | **FGD5-AS1** | **1** |
| **2598** | **chr 3: 14989090-15095107** | **FGD5-AS1** | **1** |
| **2599** | **chr 3: 15422781-15563258** | **EAF1** | **2** |
| **2599** | **chr 3: 15422781-15563258** | **METTL6** | **2** |
| **2600** | **chr 3: 15642847-15687329** | **HACL1** | **1** |
| **2600** | **chr 3: 15642847-15687329** | **BTD** | **1** |
| **2601** | **chr 3: 16355080-16555533** | **RFTN1** | **1** |
| **2602** | **chr 3: 16729492-16738986** | **none** | **1** |
| **2603** | **chr 3: 17198653-18487080** | **TBC1D5** | **8** |
| **2604** | **chr 3: 18486515-18959184** | **SATB1** | **1** |
| **2605** | **chr 3: 21459914-22414812** | **ZNF385D** | **3** |
| **2606** | **chr 3: 23236560-23244069** | **UBE2E2** | **1** |
| **2607** | **chr 3: 23244510-23633284** | **MIR548AC** | **3** |
| **2607** | **chr 3: 23244510-23633284** | **UBE2E2** | **3** |
| **2608** | **chr 3: 23847393-23932807** | **UBE2E1** | **1** |
| **2609** | **chr 3: 23986750-24022109** | **NR1D2** | **1** |
| **2610** | **chr 3: 24158650-24536773** | **THRB-AS1** | **2** |
| **2610** | **chr 3: 24158650-24536773** | **THRB** | **2** |
| **2611** | **chr 3: 25215822-25639423** | **RARB** | **2** |
| **2612** | **chr 3: 28390636-28579613** | **AZI2** | **1** |
| **2613** | **chr 3: 29322472-30051886** | **RBMS3** | **3** |
| **2614** | **chr 3: 30647993-30735634** | **TGFBR2** | **1** |
| **2615** | **chr 3: 30767691-30936257** | **GADL1** | **1** |
| **2616** | **chr 3: 31574129-31679112** | **STT3B** | **1** |
| **2617** | **chr 3: 31699381-32119072** | **OSBPL10** | **3** |
| **2617** | **chr 3: 31699381-32119072** | **OSBPL10-AS1** | **3** |
| **2618** | **chr 3: 32147180-32210205** | **GPD1L** | **1** |
| **2619** | **chr 3: 32280170-32411817** | **CMTM8** | **1** |
| **2620** | **chr 3: 33318516-33445154** | **FBXL2** | **1** |
| **2621** | **chr 3: 33429827-33482863** | **UBP1** | **1** |
| **2622** | **chr 3: 33537736-33759848** | **CLASP2** | **1** |
| **2623** | **chr 3: 34200825-34604369** | **none** | **3** |
| **2624** | **chr 3: 37094116-37225180** | **LRRFIP2** | **1** |
| **2625** | **chr 3: 37493605-37865005** | **ITGA9** | **1** |
| **2626** | **chr 3: 37903450-38025960** | **CTDSPL** | **1** |
| **2626** | **chr 3: 37903450-38025960** | **MIR26A1** | **1** |
| **2627** | **chr 3: 38080695-38165516** | **DLEC1** | **2** |
| **2627** | **chr 3: 38080695-38165516** | **ACAA1** | **2** |
| **2628** | **chr 3: 38144619-38178733** | **DLEC1** | **1** |
| **2628** | **chr 3: 38144619-38178733** | **ACAA1** | **1** |
| **2629** | **chr 3: 38206579-38296979** | **OXSR1** | **1** |
| **2630** | **chr 3: 39850404-40301812** | **EIF1B-AS1** | **4** |
| **2630** | **chr 3: 39850404-40301812** | **MYRIP** | **4** |
| **2631** | **chr 3: 40807697-40904117** | **none** | **2** |
| **2632** | **chr 3: 42530790-42579059** | **VIPR1** | **1** |
| **2633** | **chr 3: 42846243-43101703** | **HIGD1A** | **1** |
| **2634** | **chr 3: 42897496-42917633** | **CYP8B1** | **1** |
| **2634** | **chr 3: 42897496-42917633** | **CCBP2** | **1** |
| **2635** | **chr 3: 43396350-43733086** | **ANO10** | **1** |
| **2635** | **chr 3: 43396350-43733086** | **ABHD5** | **1** |
| **2636** | **chr 3: 44598848-44727145** | **ZKSCAN7** | **1** |
| **2637** | **chr 3: 44803208-44914868** | **KIF15** | **2** |
| **2637** | **chr 3: 44803208-44914868** | **KIAA1143** | **2** |
| **2638** | **chr 3: 44916099-44956482** | **TGM4** | **2** |
| **2639** | **chr 3: 46556912-46621589** | **LRRC2-AS1** | **1** |
| **2639** | **chr 3: 46556912-46621589** | **LRRC2** | **1** |
| **2640** | **chr 3: 46753604-46854064** | **PRSS46** | **1** |
| **2640** | **chr 3: 46753604-46854064** | **PRSS50** | **1** |
| **2641** | **chr 3: 46899361-46923659** | **PTH1R** | **1** |
| **2641** | **chr 3: 46899361-46923659** | **MYL3** | **1** |
| **2642** | **chr 3: 46919235-46945287** | **PTH1R** | **2** |
| **2643** | **chr 3: 47057918-47205457** | **SETD2** | **1** |
| **2643** | **chr 3: 47057918-47205457** | **KIF9-AS1** | **1** |
| **2644** | **chr 3: 47626761-47823596** | **SMARCC1** | **1** |
| **2645** | **chr 3: 47892181-48130769** | **MAP4** | **1** |
| **2646** | **chr 3: 48673901-48700348** | **CELSR3** | **1** |
| **2646** | **chr 3: 48673901-48700348** | **SLC26A6** | **1** |
| **2647** | **chr 3: 49454210-49460186** | **NICN1** | **1** |
| **2647** | **chr 3: 49454210-49460186** | **AMT** | **1** |
| **2648** | **chr 3: 49591921-49708978** | **BSN** | **2** |
| **2648** | **chr 3: 49591921-49708978** | **BSN-AS2** | **2** |
| **2649** | **chr 3: 50325162-50336899** | **LSMEM2** | **1** |
| **2649** | **chr 3: 50325162-50336899** | **IFRD2** | **1** |
| **2650** | **chr 3: 50400232-50541675** | **CACNA2D2** | **2** |
| **2651** | **chr 3: 50595461-50608458** | **C3ORF18** | **2** |
| **2652** | **chr 3: 50712671-51421629** | **DOCK3** | **3** |
| **2653** | **chr 3: 52350334-52434507** | **DNAH1** | **2** |
| **2654** | **chr 3: 52846990-52931612** | **MUSTN1** | **1** |
| **2654** | **chr 3: 52846990-52931612** | **ITIH4** | **1** |
| **2655** | **chr 3: 52937587-53164478** | **SFMBT1** | **1** |
| **2655** | **chr 3: 52937587-53164478** | **RFT1** | **1** |
| **2656** | **chr 3: 54156573-55108584** | **CACNA2D3** | **8** |
| **2656** | **chr 3: 54156573-55108584** | **ESRG** | **8** |
| **2657** | **chr 3: 55542335-56502391** | **ERC2-IT1** | **2** |
| **2657** | **chr 3: 55542335-56502391** | **ERC2** | **2** |
| **2658** | **chr 3: 56761445-57113357** | **ARHGEF3** | **1** |
| **2659** | **chr 3: 57611183-57678816** | **DENND6A** | **1** |
| **2660** | **chr 3: 57614536-57640645** | **DENND6A** | **1** |
| **2661** | **chr 3: 57994126-58157982** | **FLNB** | **1** |
| **2662** | **chr 3: 58148273-58156363** | **FLNB** | **1** |
| **2663** | **chr 3: 58703091-59035810** | **C3ORF67** | **1** |
| **2664** | **chr 3: 59735035-61237133** | **FHIT** | **5** |
| **2665** | **chr 3: 61547242-62283288** | **PTPRG** | **3** |
| **2665** | **chr 3: 61547242-62283288** | **PTPRG-AS1** | **3** |
| **2666** | **chr 3: 62246539-62355005** | **PTPRG** | **2** |
| **2666** | **chr 3: 62246539-62355005** | **PTPRG-AS1** | **2** |
| **2667** | **chr 3: 62384021-62861054** | **CADPS** | **2** |
| **2668** | **chr 3: 62936104-63110738** | **LINC00698** | **4** |
| **2669** | **chr 3: 63213990-63602597** | **SYNPR** | **2** |
| **2669** | **chr 3: 63213990-63602597** | **SYNPR-AS1** | **2** |
| **2670** | **chr 3: 64079542-64431152** | **PRICKLE2-AS1** | **1** |
| **2671** | **chr 3: 64501332-64673676** | **ADAMTS9** | **2** |
| **2671** | **chr 3: 64501332-64673676** | **ADAMTS9-AS2** | **2** |
| **2672** | **chr 3: 64670584-64997143** | **ADAMTS9** | **1** |
| **2672** | **chr 3: 64670584-64997143** | **ADAMTS9-AS2** | **1** |
| **2673** | **chr 3: 65339199-66024509** | **MAGI1** | **2** |
| **2674** | **chr 3: 66429220-66551687** | **SLC25A26** | **1** |
| **2675** | **chr 3: 67410883-67705038** | **SUCLG2** | **3** |
| **2676** | **chr 3: 68053358-68594776** | **FAM19A1** | **1** |
| **2677** | **chr 3: 69219140-69591734** | **FRMD4B** | **1** |
| **2678** | **chr 3: 69788585-70017488** | **MITF** | **2** |
| **2679** | **chr 3: 70246044-70361877** | **none** | **1** |
| **2680** | **chr 3: 71003843-71633140** | **FOXP1** | **5** |
| **2681** | **chr 3: 72084450-72291716** | **LOC201617** | **1** |
| **2682** | **chr 3: 73431583-73674091** | **PDZRN3** | **1** |
| **2683** | **chr 3: 73857751-73952017** | **none** | **2** |
| **2684** | **chr 3: 74311718-74570291** | **CNTN3** | **3** |
| **2685** | **chr 3: 75495919-75540353** | **FAM86DP** | **1** |
| **2686** | **chr 3: 75589199-75629150** | **MIR1324** | **1** |
| **2687** | **chr 3: 75955845-77699115** | **ROBO2** | **5** |
| **2688** | **chr 3: 78646389-79816965** | **ROBO1** | **2** |
| **2689** | **chr 3: 81043018-81144798** | **none** | **1** |
| **2690** | **chr 3: 82035288-82512826** | **none** | **2** |
| **2691** | **chr 3: 85008131-86123579** | **CADM2** | **2** |
| **2691** | **chr 3: 85008131-86123579** | **MIR5688** | **2** |
| **2692** | **chr 3: 88101093-88199035** | **CGGBP1** | **1** |
| **2693** | **chr 3: 88108423-88188123** | **CGGBP1** | **1** |
| **2694** | **chr 3: 89156673-89531284** | **EPHA3** | **2** |
| **2695** | **chr 3: 93591880-93692910** | **PROS1** | **2** |
| **2696** | **chr 3: 96533424-97471304** | **EPHA6** | **1** |
| **2697** | **chr 3: 97660661-97691301** | **MINA** | **1** |
| **2697** | **chr 3: 97660661-97691301** | **CRYBG3** | **1** |
| **2698** | **chr 3: 97952494-98176742** | **OR5H6** | **1** |
| **2698** | **chr 3: 97952494-98176742** | **OR5H2** | **1** |
| **2699** | **chr 3: 98216755-98312567** | **CLDND1** | **2** |
| **2699** | **chr 3: 98216755-98312567** | **OR5K2** | **2** |
| **2700** | **chr 3: 98514784-98620533** | **DCBLD2** | **1** |
| **2701** | **chr 3: 99536677-99897447** | **MIR548G** | **3** |
| **2702** | **chr 3: 99548984-99833357** | **MIR548G** | **2** |
| **2703** | **chr 3: 99904530-99913035** | **TMEM30C** | **1** |
| **2704** | **chr 3: 100211462-100296288** | **TMEM45A** | **1** |
| **2705** | **chr 3: 101818087-102198685** | **ZPLD1** | **1** |
| **2706** | **chr 3: 105085752-105295744** | **ALCAM** | **3** |
| **2707** | **chr 3: 107149776-107182759** | **none** | **2** |
| **2708** | **chr 3: 107241782-107530171** | **BBX** | **1** |
| **2709** | **chr 3: 108099215-108248169** | **MYH15** | **1** |
| **2710** | **chr 3: 109044987-109056419** | **DPPA4** | **1** |
| **2711** | **chr 3: 110788917-110994410** | **PVRL3-AS1** | **1** |
| **2711** | **chr 3: 110788917-110994410** | **PVRL3** | **1** |
| **2712** | **chr 3: 111753689-111800116** | **TMPRSS7** | **1** |
| **2713** | **chr 3: 112929849-113006303** | **BOC** | **1** |
| **2713** | **chr 3: 112929849-113006303** | **WDR52** | **1** |
| **2714** | **chr 3: 113251142-113348425** | **SIDT1** | **1** |
| **2714** | **chr 3: 113251142-113348425** | **MIR4446** | **1** |
| **2715** | **chr 3: 113682983-113775460** | **QTRTD1** | **1** |
| **2715** | **chr 3: 113682983-113775460** | **KIAA1407** | **1** |
| **2716** | **chr 3: 113724679-113807269** | **QTRTD1** | **1** |
| **2716** | **chr 3: 113724679-113807269** | **KIAA1407** | **1** |
| **2717** | **chr 3: 113995759-114029135** | **TIGIT** | **2** |
| **2718** | **chr 3: 114056940-114866118** | **ZBTB20** | **1** |
| **2719** | **chr 3: 114172439-114238979** | **ZBTB20** | **3** |
| **2720** | **chr 3: 115342170-115440337** | **GAP43** | **1** |
| **2721** | **chr 3: 115521234-117716439** | **LSAMP-AS3** | **5** |
| **2721** | **chr 3: 115521234-117716439** | **LSAMP** | **5** |
| **2722** | **chr 3: 117723665-118276515** | **none** | **3** |
| **2723** | **chr 3: 118619403-118864915** | **IGSF11** | **1** |
| **2724** | **chr 3: 119013219-119139561** | **ARHGAP31** | **1** |
| **2725** | **chr 3: 119148346-119187677** | **POGLUT1** | **2** |
| **2725** | **chr 3: 119148346-119187677** | **TMEM39A** | **2** |
| **2726** | **chr 3: 119540169-119813264** | **GSK3B** | **1** |
| **2727** | **chr 3: 119883491-120003941** | **GPR156** | **1** |
| **2728** | **chr 3: 120347019-120401418** | **HGD** | **1** |
| **2729** | **chr 3: 120405527-120461840** | **GTF2E1** | **1** |
| **2729** | **chr 3: 120405527-120461840** | **RABL3** | **1** |
| **2730** | **chr 3: 120626918-121143608** | **STXBP5L** | **3** |
| **2731** | **chr 3: 121350245-121379774** | **HCLS1** | **1** |
| **2732** | **chr 3: 122140795-122233792** | **KPNA1** | **1** |
| **2733** | **chr 3: 122628040-122747452** | **SEMA5B** | **1** |
| **2734** | **chr 3: 122785908-122992977** | **PDIA5** | **1** |
| **2734** | **chr 3: 122785908-122992977** | **SEC22A** | **1** |
| **2735** | **chr 3: 123001142-123168605** | **ADCY5** | **3** |
| **2736** | **chr 3: 123687367-123711025** | **ROPN1** | **1** |
| **2737** | **chr 3: 123798869-124445172** | **KALRN** | **4** |
| **2738** | **chr 3: 124480794-124620265** | **ITGB5** | **1** |
| **2739** | **chr 3: 125493556-125516796** | **MIR548I1** | **1** |
| **2740** | **chr 3: 125725197-125820404** | **SLC41A3** | **1** |
| **2741** | **chr 3: 125985638-126010122** | **none** | **1** |
| **2742** | **chr 3: 126423062-126679249** | **CHCHD6** | **1** |
| **2743** | **chr 3: 127407908-127542051** | **MGLL** | **1** |
| **2744** | **chr 3: 127872296-128127485** | **EEFSEC** | **2** |
| **2745** | **chr 3: 128338816-128399918** | **RPN1** | **2** |
| **2746** | **chr 3: 129158967-129239198** | **MBD4** | **1** |
| **2747** | **chr 3: 130745693-131069309** | **NEK11** | **1** |
| **2747** | **chr 3: 130745693-131069309** | **ASTE1** | **1** |
| **2748** | **chr 3: 131043935-131100319** | **LOC339874** | **2** |
| **2748** | **chr 3: 131043935-131100319** | **NEK11** | **2** |
| **2749** | **chr 3: 131252398-132004254** | **MIR5704** | **2** |
| **2749** | **chr 3: 131252398-132004254** | **CPNE4** | **2** |
| **2750** | **chr 3: 132440593-132593067** | **NPHP3-ACAD11** | **2** |
| **2751** | **chr 3: 134070693-134094321** | **AMOTL2** | **1** |
| **2752** | **chr 3: 134316642-134979309** | **KY** | **3** |
| **2752** | **chr 3: 134316642-134979309** | **EPHB1** | **3** |
| **2753** | **chr 3: 135074908-135158730** | **none** | **1** |
| **2754** | **chr 3: 136055076-136471220** | **STAG1** | **2** |
| **2755** | **chr 3: 136537488-136574734** | **SLC35G2** | **2** |
| **2756** | **chr 3: 137717576-137752494** | **CLDN18** | **1** |
| **2757** | **chr 3: 138372859-138553780** | **PIK3CB** | **1** |
| **2758** | **chr 3: 138724647-139076065** | **PRR23A** | **3** |
| **2758** | **chr 3: 138724647-139076065** | **PRR23B** | **3** |
| **2759** | **chr 3: 139279021-139396859** | **NMNAT3** | **2** |
| **2760** | **chr 3: 139654026-140296239** | **CLSTN2** | **1** |
| **2761** | **chr 3: 140986194-141085979** | **ZBTB38** | **3** |
| **2761** | **chr 3: 140986194-141085979** | **ACPL2** | **3** |
| **2762** | **chr 3: 141043054-141168634** | **ZBTB38** | **3** |
| **2763** | **chr 3: 142168076-142297668** | **ATR** | **6** |
| **2764** | **chr 3: 142842203-142850735** | **CHST2** | **1** |
| **2765** | **chr 3: 142984063-143567373** | **SLC9A9** | **2** |
| **2765** | **chr 3: 142984063-143567373** | **SLC9A9-AS1** | **2** |
| **2766** | **chr 3: 146109149-146213778** | **PLSCR2** | **1** |
| **2767** | **chr 3: 146627471-147088443** | **ZIC4** | **2** |
| **2768** | **chr 3: 147111208-147228080** | **ZIC4** | **1** |
| **2769** | **chr 3: 147913515-147997885** | **none** | **2** |
| **2770** | **chr 3: 147998677-148117743** | **none** | **1** |
| **2771** | **chr 3: 148880196-148939842** | **CP** | **1** |
| **2771** | **chr 3: 148880196-148939842** | **HPS3** | **1** |
| **2772** | **chr 3: 148947142-149009587** | **TM4SF18** | **1** |
| **2772** | **chr 3: 148947142-149009587** | **CP** | **1** |
| **2773** | **chr 3: 149191760-149221068** | **TM4SF4** | **1** |
| **2774** | **chr 3: 149235021-149454501** | **WWTR1** | **1** |
| **2775** | **chr 3: 149478891-149768575** | **ANKUB1** | **4** |
| **2775** | **chr 3: 149478891-149768575** | **RNF13** | **4** |
| **2776** | **chr 3: 149530494-149679926** | **RNF13** | **2** |
| **2777** | **chr 3: 149757000-149931513** | **none** | **1** |
| **2778** | **chr 3: 150126121-150184218** | **TSC22D2** | **1** |
| **2779** | **chr 3: 150570270-150798513** | **CLRN1** | **1** |
| **2780** | **chr 3: 153102722-153697975** | **C3ORF79** | **1** |
| **2781** | **chr 3: 153838791-153975616** | **ARHGEF26-AS1** | **2** |
| **2781** | **chr 3: 153838791-153975616** | **ARHGEF26** | **2** |
| **2782** | **chr 3: 155093368-155462856** | **PLCH1** | **1** |
| **2783** | **chr 3: 155755489-156256545** | **KCNAB1** | **3** |
| **2784** | **chr 3: 156543269-156763918** | **LEKR1** | **1** |
| **2785** | **chr 3: 156977530-157251408** | **VEPH1** | **1** |
| **2786** | **chr 3: 157261034-157395538** | **C3ORF55** | **3** |
| **2787** | **chr 3: 157823643-158263519** | **RSRC1** | **2** |
| **2787** | **chr 3: 157823643-158263519** | **SHOX2** | **2** |
| **2788** | **chr 3: 158288951-158325041** | **MLF1** | **1** |
| **2789** | **chr 3: 158680023-159615155** | **IQCJ** | **2** |
| **2790** | **chr 3: 159945240-160203561** | **C3ORF80** | **2** |
| **2790** | **chr 3: 159945240-160203561** | **IFT80** | **2** |
| **2791** | **chr 3: 160801670-160823172** | **B3GALNT1** | **1** |
| **2792** | **chr 3: 164924747-165373211** | **SLITRK3** | **4** |
| **2793** | **chr 3: 166958074-167099134** | **ZBBX** | **1** |
| **2794** | **chr 3: 167110583-167126307** | **SERPINI2** | **1** |
| **2794** | **chr 3: 167110583-167126307** | **ZBBX** | **1** |
| **2795** | **chr 3: 167159576-167371771** | **WDR49** | **2** |
| **2795** | **chr 3: 167159576-167371771** | **SERPINI2** | **2** |
| **2796** | **chr 3: 167401085-167452727** | **PDCD10** | **1** |
| **2797** | **chr 3: 167726464-167813763** | **GOLIM4** | **1** |
| **2798** | **chr 3: 168801286-169381406** | **MECOM** | **3** |
| **2799** | **chr 3: 169165654-169194840** | **MECOM** | **1** |
| **2800** | **chr 3: 169556966-169587718** | **LRRC31** | **1** |
| **2801** | **chr 3: 170136652-170578169** | **CLDN11** | **3** |
| **2802** | **chr 3: 171318194-171528740** | **PLD1** | **2** |
| **2803** | **chr 3: 171561138-171656505** | **TMEM212** | **2** |
| **2804** | **chr 3: 171594141-171618530** | **TMEM212** | **2** |
| **2805** | **chr 3: 172348038-172429008** | **NCEH1** | **1** |
| **2806** | **chr 3: 172607147-172859058** | **SPATA16** | **2** |
| **2807** | **chr 3: 173114073-174004434** | **NLGN1** | **3** |
| **2808** | **chr 3: 174156362-175523428** | **NAALADL2** | **1** |
| **2808** | **chr 3: 174156362-175523428** | **NAALADL2-AS3** | **1** |
| **2809** | **chr 3: 174797096-174833032** | **NAALADL2** | **1** |
| **2809** | **chr 3: 174797096-174833032** | **NAALADL2-AS3** | **1** |
| **2810** | **chr 3: 176361728-176585626** | **none** | **6** |
| **2811** | **chr 3: 176737142-176915261** | **TBL1XR1** | **1** |
| **2812** | **chr 3: 177012229-177041206** | **none** | **1** |
| **2813** | **chr 3: 177159708-177470093** | **LINC00578** | **2** |
| **2814** | **chr 3: 177919837-178103205** | **KCNMB2-IT1** | **1** |
| **2815** | **chr 3: 177990719-178562217** | **KCNMB2-IT1** | **1** |
| **2815** | **chr 3: 177990719-178562217** | **KCNMB2** | **1** |
| **2816** | **chr 3: 178244292-178578193** | **KCNMB2** | **4** |
| **2817** | **chr 3: 179280667-179306196** | **ACTL6A** | **1** |
| **2818** | **chr 3: 179370542-179507189** | **USP13** | **2** |
| **2819** | **chr 3: 179512745-179754841** | **PEX5L** | **2** |
| **2820** | **chr 3: 180585928-180700541** | **FXR1** | **3** |
| **2821** | **chr 3: 180707557-181554668** | **DNAJC19** | **6** |
| **2822** | **chr 3: 182164757-182204152** | **FLJ46066** | **1** |
| **2823** | **chr 3: 182655861-182703741** | **DCUN1D1** | **1** |
| **2824** | **chr 3: 182895830-183146566** | **B3GNT5** | **1** |
| **2824** | **chr 3: 182895830-183146566** | **MCF2L2** | **1** |
| **2825** | **chr 3: 182971031-183016292** | **B3GNT5** | **1** |
| **2825** | **chr 3: 182971031-183016292** | **MCF2L2** | **1** |
| **2826** | **chr 3: 183205318-183273477** | **KLHL6** | **1** |
| **2827** | **chr 3: 183415605-183530413** | **YEATS2** | **2** |
| **2828** | **chr 3: 183637721-183735803** | **ABCC5** | **1** |
| **2829** | **chr 3: 183852825-184402546** | **DVL3** | **2** |
| **2829** | **chr 3: 183852825-184402546** | **EIF2B5** | **2** |
| **2830** | **chr 3: 184529930-184770402** | **VPS8** | **1** |
| **2831** | **chr 3: 184880658-184909743** | **EHHADH-AS1** | **1** |
| **2831** | **chr 3: 184880658-184909743** | **EHHADH** | **1** |
| **2832** | **chr 3: 185000728-185206885** | **MAP3K13** | **2** |
| **2833** | **chr 3: 185764096-186080026** | **ETV5** | **2** |
| **2833** | **chr 3: 185764096-186080026** | **DGKG** | **2** |
| **2834** | **chr 3: 186560462-186576252** | **ADIPOQ-AS1** | **1** |
| **2834** | **chr 3: 186560462-186576252** | **ADIPOQ** | **1** |
| **2835** | **chr 3: 186648273-186796341** | **ST6GAL1** | **1** |
| **2836** | **chr 3: 186838735-186898696** | **RPL39L** | **1** |
| **2837** | **chr 3: 187439164-187463515** | **BCL6** | **2** |
| **2837** | **chr 3: 187439164-187463515** | **LOC100131635** | **2** |
| **2838** | **chr 3: 187461473-187463208** | **BCL6** | **1** |
| **2839** | **chr 3: 187871071-188608460** | **LPP-AS2** | **3** |
| **2839** | **chr 3: 187871071-188608460** | **LPP** | **3** |
| **2840** | **chr 3: 189349204-189615068** | **TP63** | **1** |
| **2841** | **chr 3: 190231839-190375843** | **IL1RAP** | **1** |
| **2842** | **chr 3: 191046865-191116459** | **CCDC50** | **1** |
| **2842** | **chr 3: 191046865-191116459** | **UTS2B** | **1** |
| **2843** | **chr 3: 191857183-192485553** | **FGF12** | **4** |
| **2844** | **chr 3: 192862252-192894653** | **none** | **1** |
| **2845** | **chr 3: 193119865-193310900** | **ATP13A4** | **1** |
| **2845** | **chr 3: 193119865-193310900** | **OPA1** | **1** |
| **2846** | **chr 3: 193675160-193721448** | **DPPA2P3** | **1** |
| **2846** | **chr 3: 193675160-193721448** | **LOC647323** | **1** |
| **2847** | **chr 3: 193698402-193723887** | **DPPA2P3** | **1** |
| **2847** | **chr 3: 193698402-193723887** | **LOC647323** | **1** |
| **2848** | **chr 3: 194789007-194991896** | **XXYLT1** | **2** |
| **2849** | **chr 3: 195384932-195467994** | **SDHAP2** | **1** |
| **2849** | **chr 3: 195384932-195467994** | **MIR570** | **1** |
| **2850** | **chr 3: 195563063-195587275** | **TNK2** | **1** |
| **2850** | **chr 3: 195563063-195587275** | **MUC4** | **1** |
| **2851** | **chr 3: 195686618-195717189** | **SDHAP1** | **1** |
| **2852** | **chr 3: 195941092-196065374** | **PCYT1A** | **1** |
| **2852** | **chr 3: 195941092-196065374** | **SLC51A** | **1** |
| **2853** | **chr 3: 196045200-196052441** | **TCTEX1D2** | **1** |
| **2853** | **chr 3: 196045200-196052441** | **TM4SF19-TCTEX1D2** | **1** |
| **2854** | **chr 3: 196074532-196159345** | **UBXN7** | **2** |
| **2855** | **chr 3: 196673213-196695931** | **PIGZ** | **1** |
| **2856** | **chr 3: 196715491-196756687** | **MFI2** | **1** |
| **2856** | **chr 3: 196715491-196756687** | **MFI2-AS1** | **1** |
| **2857** | **chr 3: 197025122-197030618** | **DLG1** | **1** |
| **2858** | **chr 3: 197305083-197354777** | **LOC220729** | **1** |
| **2859** | **chr 3: 197615945-197687013** | **IQCG** | **5** |
| **2860** | **chr 4: 619372-664571** | **PDE6B** | **1** |
| **2861** | **chr 4: 843063-926161** | **TMEM175** | **1** |
| **2861** | **chr 4: 843063-926161** | **GAK** | **1** |
| **2862** | **chr 4: 1396719-1400119** | **CRIPAK** | **2** |
| **2863** | **chr 4: 1873150-1983934** | **WHSC1** | **1** |
| **2864** | **chr 4: 3675319-3679582** | **LOC100133461** | **2** |
| **2865** | **chr 4: 3916909-3957148** | **FAM86EP** | **1** |
| **2866** | **chr 4: 4145184-4152148** | **OTOP1** | **1** |
| **2867** | **chr 4: 4349866-4420785** | **NSG1** | **1** |
| **2868** | **chr 4: 5712923-5830772** | **CRMP1** | **2** |
| **2868** | **chr 4: 5712923-5830772** | **EVC** | **2** |
| **2869** | **chr 4: 5749810-5894785** | **CRMP1** | **2** |
| **2869** | **chr 4: 5749810-5894785** | **EVC** | **2** |
| **2870** | **chr 4: 5958844-5991545** | **JAKMIP1** | **2** |
| **2870** | **chr 4: 5958844-5991545** | **MIR378D1** | **2** |
| **2871** | **chr 4: 6271575-6304992** | **WFS1** | **1** |
| **2872** | **chr 4: 6322304-6565327** | **PPP2R2C** | **2** |
| **2873** | **chr 4: 6576901-6625089** | **MAN2B2** | **1** |
| **2874** | **chr 4: 7096297-7105112** | **FLJ36777** | **1** |
| **2875** | **chr 4: 7194264-7744554** | **SORCS2** | **7** |
| **2875** | **chr 4: 7194264-7744554** | **MIR4798** | **7** |
| **2876** | **chr 4: 7967038-8160559** | **ABLIM2** | **5** |
| **2877** | **chr 4: 8183798-8243530** | **SH3TC1** | **1** |
| **2878** | **chr 4: 8437866-8514337** | **ACOX3** | **1** |
| **2878** | **chr 4: 8437866-8514337** | **TRMT44** | **1** |
| **2879** | **chr 4: 8560451-8621488** | **GPR78** | **1** |
| **2880** | **chr 4: 8961353-8965707** | **LOC650293** | **1** |
| **2881** | **chr 4: 9424038-9431428** | **DEFB131** | **1** |
| **2882** | **chr 4: 9495379-9499710** | **DEFB131** | **1** |
| **2883** | **chr 4: 10488018-10686489** | **CLNK** | **1** |
| **2884** | **chr 4: 10686626-10699285** | **CLNK** | **1** |
| **2885** | **chr 4: 12225074-12252910** | **none** | **2** |
| **2886** | **chr 4: 13547698-13549425** | **LOC285548** | **1** |
| **2887** | **chr 4: 13656802-13978699** | **MIR5091** | **3** |
| **2888** | **chr 4: 14472088-14889793** | **LOC441009** | **5** |
| **2889** | **chr 4: 14911584-15003669** | **LOC441009** | **1** |
| **2889** | **chr 4: 14911584-15003669** | **CPEB2** | **1** |
| **2890** | **chr 4: 15006565-15429538** | **C1QTNF7** | **4** |
| **2890** | **chr 4: 15006565-15429538** | **CPEB2** | **4** |
| **2891** | **chr 4: 15341441-15447790** | **C1QTNF7** | **4** |
| **2892** | **chr 4: 16503163-16900432** | **LDB2** | **3** |
| **2892** | **chr 4: 16503163-16900432** | **MIR548AX** | **3** |
| **2893** | **chr 4: 16974897-17075526** | **none** | **3** |
| **2894** | **chr 4: 19173957-19458617** | **none** | **3** |
| **2895** | **chr 4: 19457058-19911315** | **none** | **1** |
| **2896** | **chr 4: 20254882-20622184** | **SLIT2-IT1** | **1** |
| **2896** | **chr 4: 20254882-20622184** | **SLIT2** | **1** |
| **2897** | **chr 4: 20730238-21950422** | **KCNIP4** | **5** |
| **2898** | **chr 4: 22346693-22517677** | **GPR125** | **1** |
| **2899** | **chr 4: 23756663-23905712** | **PPARGC1A** | **1** |
| **2900** | **chr 4: 24807738-24981826** | **CCDC149** | **1** |
| **2901** | **chr 4: 25863451-25931435** | **SEL1L3** | **1** |
| **2901** | **chr 4: 25863451-25931435** | **SMIM20** | **1** |
| **2902** | **chr 4: 25866502-25871172** | **SEL1L3** | **1** |
| **2902** | **chr 4: 25866502-25871172** | **SMIM20** | **1** |
| **2903** | **chr 4: 26165076-26436541** | **RBPJ** | **3** |
| **2904** | **chr 4: 30008572-30009938** | **none** | **1** |
| **2905** | **chr 4: 30722036-31148422** | **PCDH7** | **5** |
| **2906** | **chr 4: 31999018-32157028** | **none** | **5** |
| **2907** | **chr 4: 38422282-38524801** | **none** | **1** |
| **2908** | **chr 4: 40058445-40159872** | **LOC344967** | **1** |
| **2908** | **chr 4: 40058445-40159872** | **N4BP2** | **1** |
| **2909** | **chr 4: 40192672-40248587** | **RHOH** | **1** |
| **2910** | **chr 4: 40751913-40812002** | **NSUN7** | **1** |
| **2911** | **chr 4: 41361623-41702061** | **LIMCH1** | **2** |
| **2912** | **chr 4: 41750309-41826136** | **PHOX2B** | **1** |
| **2913** | **chr 4: 42283846-42393285** | **SHISA3** | **1** |
| **2914** | **chr 4: 42410389-42659122** | **ATP8A1** | **1** |
| **2915** | **chr 4: 43342891-43347617** | **none** | **1** |
| **2916** | **chr 4: 44175925-44450824** | **KCTD8** | **3** |
| **2917** | **chr 4: 44684216-44728612** | **GNPDA2** | **1** |
| **2917** | **chr 4: 44684216-44728612** | **GUF1** | **1** |
| **2918** | **chr 4: 46995739-47428461** | **GABRA4** | **2** |
| **2919** | **chr 4: 47487304-47595503** | **ATP10D** | **1** |
| **2920** | **chr 4: 48068409-48136273** | **TXK** | **1** |
| **2921** | **chr 4: 48137799-48271881** | **TEC** | **2** |
| **2922** | **chr 4: 52917496-52963458** | **SPATA18** | **2** |
| **2923** | **chr 4: 53739148-54232242** | **SCFD2** | **1** |
| **2924** | **chr 4: 54243809-55164414** | **FIP1L1** | **9** |
| **2924** | **chr 4: 54243809-55164414** | **LNX1** | **9** |
| **2925** | **chr 4: 56262123-56319564** | **TMEM165** | **1** |
| **2925** | **chr 4: 56262123-56319564** | **CLOCK** | **1** |
| **2926** | **chr 4: 57036360-57194791** | **KIAA1211** | **1** |
| **2927** | **chr 4: 59849999-59912677** | **none** | **1** |
| **2928** | **chr 4: 62066975-62944053** | **LPHN3** | **5** |
| **2929** | **chr 4: 68337520-68411324** | **CENPC1** | **1** |
| **2930** | **chr 4: 68424445-68473055** | **STAP1** | **2** |
| **2931** | **chr 4: 68566997-68946670** | **UBA6** | **1** |
| **2931** | **chr 4: 68566997-68946670** | **LOC550112** | **1** |
| **2932** | **chr 4: 68605045-68620078** | **GNRHR** | **1** |
| **2933** | **chr 4: 69313166-69363322** | **TMPRSS11E** | **4** |
| **2934** | **chr 4: 70999332-71042516** | **C4ORF40** | **1** |
| **2934** | **chr 4: 70999332-71042516** | **CSN1S2BP** | **1** |
| **2935** | **chr 4: 71768042-71896631** | **MOB1B** | **1** |
| **2936** | **chr 4: 72053002-72437804** | **SLC4A4** | **1** |
| **2937** | **chr 4: 75858304-75975325** | **PARM1** | **1** |
| **2938** | **chr 4: 76567965-76649709** | **G3BP2** | **1** |
| **2939** | **chr 4: 77356252-77704406** | **SHROOM3** | **1** |
| **2939** | **chr 4: 77356252-77704406** | **MIR4450** | **1** |
| **2940** | **chr 4: 78078303-78354542** | **CCNG2** | **2** |
| **2941** | **chr 4: 78315644-78415440** | **CXCL13** | **1** |
| **2942** | **chr 4: 78978723-79465423** | **FRAS1** | **1** |
| **2943** | **chr 4: 79892901-80229952** | **LOC100505875** | **2** |
| **2944** | **chr 4: 81187752-81884910** | **FGF5** | **1** |
| **2944** | **chr 4: 81187752-81884910** | **C4ORF22** | **1** |
| **2945** | **chr 4: 82009836-82136218** | **PRKG2** | **1** |
| **2946** | **chr 4: 82347546-82965397** | **RASGEF1B** | **1** |
| **2947** | **chr 4: 83273650-83295656** | **HNRNPD** | **1** |
| **2948** | **chr 4: 83404322-83483510** | **TMEM150C** | **1** |
| **2949** | **chr 4: 83739813-83822319** | **SEC31A** | **1** |
| **2950** | **chr 4: 83831125-83934079** | **THAP9** | **4** |
| **2950** | **chr 4: 83831125-83934079** | **LIN54** | **4** |
| **2951** | **chr 4: 84589662-84652908** | **none** | **1** |
| **2952** | **chr 4: 85504131-85572491** | **CDS1** | **1** |
| **2953** | **chr 4: 86396266-86923823** | **MIR4451** | **5** |
| **2953** | **chr 4: 86396266-86923823** | **ARHGAP24** | **5** |
| **2954** | **chr 4: 86936275-87515284** | **MAPK10** | **2** |
| **2955** | **chr 4: 87515467-87736324** | **PTPN13** | **3** |
| **2956** | **chr 4: 88081254-88161466** | **KLHL8** | **1** |
| **2957** | **chr 4: 88489186-88655108** | **DSPP** | **4** |
| **2957** | **chr 4: 88489186-88655108** | **DMP1** | **4** |
| **2958** | **chr 4: 89378267-89427314** | **HERC5** | **2** |
| **2959** | **chr 4: 89442198-89629693** | **PIGY** | **1** |
| **2959** | **chr 4: 89442198-89629693** | **PYURF** | **1** |
| **2960** | **chr 4: 91048685-92523064** | **CCSER1** | **3** |
| **2961** | **chr 4: 93225549-94695707** | **GRID2** | **3** |
| **2962** | **chr 4: 95038942-95128707** | **SMARCAD1** | **1** |
| **2963** | **chr 4: 95373036-95589377** | **PDLIM5** | **2** |
| **2964** | **chr 4: 95679118-96079599** | **BMPR1B** | **2** |
| **2965** | **chr 4: 96083654-96470357** | **UNC5C** | **2** |
| **2966** | **chr 4: 97231851-97740015** | **none** | **1** |
| **2967** | **chr 4: 98105243-99064391** | **STPG2** | **4** |
| **2968** | **chr 4: 99391517-99579780** | **TSPAN5** | **1** |
| **2969** | **chr 4: 100557685-100575805** | **MTTP** | **1** |
| **2970** | **chr 4: 101316497-101801283** | **EMCN-IT3** | **1** |
| **2970** | **chr 4: 101316497-101801283** | **EMCN** | **1** |
| **2971** | **chr 4: 101944565-102269435** | **PPP3CA** | **2** |
| **2971** | **chr 4: 101944565-102269435** | **FLJ20021** | **2** |
| **2972** | **chr 4: 102332442-102995969** | **BANK1** | **2** |
| **2973** | **chr 4: 103172197-103371167** | **SLC39A8** | **1** |
| **2974** | **chr 4: 105412121-105618749** | **CXXC4** | **3** |
| **2975** | **chr 4: 105575030-105887950** | **none** | **1** |
| **2976** | **chr 4: 105828513-106041157** | **TET2** | **1** |
| **2977** | **chr 4: 106067031-106200973** | **TET2** | **1** |
| **2978** | **chr 4: 106290233-106395238** | **PPA2** | **1** |
| **2979** | **chr 4: 107842958-108204963** | **DKK2** | **4** |
| **2980** | **chr 4: 108511432-108641608** | **PAPSS1** | **1** |
| **2981** | **chr 4: 108745718-108836203** | **SGMS2** | **1** |
| **2982** | **chr 4: 108784634-108899955** | **SGMS2** | **1** |
| **2983** | **chr 4: 108852524-108874613** | **CYP2U1** | **1** |
| **2984** | **chr 4: 108968700-109090112** | **LEF1** | **1** |
| **2985** | **chr 4: 109459345-109541616** | **RPL34-AS1** | **3** |
| **2985** | **chr 4: 109459345-109541616** | **RPL34** | **3** |
| **2986** | **chr 4: 109731876-110223813** | **COL25A1** | **1** |
| **2987** | **chr 4: 110661851-110723335** | **CFI** | **2** |
| **2988** | **chr 4: 110834039-110933422** | **EGF** | **1** |
| **2989** | **chr 4: 111286888-111486441** | **ENPEP** | **1** |
| **2990** | **chr 4: 111516670-111536614** | **PITX2** | **1** |
| **2990** | **chr 4: 111516670-111536614** | **ENPEP** | **1** |
| **2991** | **chr 4: 114821439-114900883** | **ARSJ** | **2** |
| **2992** | **chr 4: 119643977-119759838** | **SEC24D** | **3** |
| **2993** | **chr 4: 121606073-121844025** | **PRDM5** | **1** |
| **2994** | **chr 4: 121956767-121994176** | **NDNF** | **2** |
| **2995** | **chr 4: 121992583-122001631** | **NDNF** | **2** |
| **2996** | **chr 4: 122052562-122148621** | **TNIP3** | **2** |
| **2997** | **chr 4: 123747862-123819391** | **FGF2** | **1** |
| **2997** | **chr 4: 123747862-123819391** | **NUDT6** | **1** |
| **2998** | **chr 4: 124571421-124851561** | **LOC285419** | **2** |
| **2999** | **chr 4: 126597872-126673948** | **none** | **1** |
| **3000** | **chr 4: 127964584-127998917** | **none** | **1** |
| **3001** | **chr 4: 128544425-128647892** | **INTU** | **1** |
| **3002** | **chr 4: 128982422-129144086** | **LARP1B** | **1** |
| **3003** | **chr 4: 129190396-129209984** | **PGRMC2** | **1** |
| **3004** | **chr 4: 129213905-129440549** | **PGRMC2** | **2** |
| **3005** | **chr 4: 130014471-130037795** | **SCLT1** | **1** |
| **3005** | **chr 4: 130014471-130037795** | **C4ORF33** | **1** |
| **3006** | **chr 4: 130220329-130226177** | **none** | **1** |
| **3007** | **chr 4: 131297383-131307526** | **none** | **1** |
| **3008** | **chr 4: 132301167-132462552** | **none** | **1** |
| **3009** | **chr 4: 139230864-139345498** | **LINC00499** | **2** |
| **3010** | **chr 4: 139694703-139722793** | **none** | **3** |
| **3011** | **chr 4: 139949265-140098372** | **ELF2** | **1** |
| **3011** | **chr 4: 139949265-140098372** | **CCRN4L** | **1** |
| **3012** | **chr 4: 140222608-140341187** | **NDUFC1** | **3** |
| **3013** | **chr 4: 140333080-140375188** | **RAB33B** | **1** |
| **3014** | **chr 4: 140417094-140527853** | **SETD7** | **2** |
| **3015** | **chr 4: 140637906-141075338** | **MGST2** | **1** |
| **3015** | **chr 4: 140637906-141075338** | **MAML3** | **1** |
| **3016** | **chr 4: 141677681-141679075** | **TBC1D9** | **1** |
| **3017** | **chr 4: 142944312-143768585** | **INPP4B** | **3** |
| **3018** | **chr 4: 143487171-143582103** | **INPP4B** | **1** |
| **3019** | **chr 4: 143854347-144106014** | **USP38** | **1** |
| **3020** | **chr 4: 144207445-144251011** | **GAB1** | **1** |
| **3021** | **chr 4: 144257914-144395721** | **GAB1** | **1** |
| **3022** | **chr 4: 144621409-144786225** | **FREM3** | **1** |
| **3023** | **chr 4: 144833483-145047512** | **GYPA** | **2** |
| **3023** | **chr 4: 144833483-145047512** | **GYPB** | **2** |
| **3024** | **chr 4: 144917256-145061904** | **GYPA** | **1** |
| **3024** | **chr 4: 144917256-145061904** | **GYPB** | **1** |
| **3025** | **chr 4: 146031989-146101313** | **OTUD4** | **1** |
| **3025** | **chr 4: 146031989-146101313** | **ABCE1** | **1** |
| **3026** | **chr 4: 146601355-146692184** | **C4ORF51** | **2** |
| **3026** | **chr 4: 146601355-146692184** | **ZNF827** | **2** |
| **3027** | **chr 4: 146678778-146859787** | **ZNF827** | **1** |
| **3028** | **chr 4: 148653213-148993931** | **MIR4799** | **1** |
| **3028** | **chr 4: 148653213-148993931** | **ARHGAP10** | **1** |
| **3029** | **chr 4: 148999912-149365850** | **NR3C2** | **2** |
| **3030** | **chr 4: 149067621-149130031** | **NR3C2** | **1** |
| **3031** | **chr 4: 150273168-150817385** | **none** | **3** |
| **3032** | **chr 4: 151185593-151936879** | **LRBA** | **6** |
| **3033** | **chr 4: 153128572-153147022** | **none** | **2** |
| **3034** | **chr 4: 153955362-154012317** | **none** | **1** |
| **3035** | **chr 4: 154073493-154260472** | **TRIM2** | **4** |
| **3036** | **chr 4: 154387497-154557863** | **KIAA0922** | **3** |
| **3037** | **chr 4: 154631276-154681387** | **RNF175** | **1** |
| **3038** | **chr 4: 154641478-154648874** | **RNF175** | **1** |
| **3039** | **chr 4: 155153398-155412930** | **DCHS2** | **1** |
| **3040** | **chr 4: 157681605-157892546** | **PDGFC** | **2** |
| **3041** | **chr 4: 159236462-159574524** | **RXFP1** | **2** |
| **3042** | **chr 4: 159690289-159829201** | **FNIP2** | **2** |
| **3042** | **chr 4: 159690289-159829201** | **C4ORF45** | **2** |
| **3043** | **chr 4: 162305048-163085187** | **FSTL5** | **1** |
| **3044** | **chr 4: 164445449-165305202** | **MARCH1** | **1** |
| **3045** | **chr 4: 166128769-166244308** | **KLHL2** | **1** |
| **3046** | **chr 4: 166282345-166419472** | **CPE** | **1** |
| **3046** | **chr 4: 166282345-166419472** | **MIR578** | **1** |
| **3047** | **chr 4: 166794409-167025047** | **TLL1** | **2** |
| **3048** | **chr 4: 167654534-168155947** | **SPOCK3** | **2** |
| **3049** | **chr 4: 169137443-169239958** | **DDX60** | **1** |
| **3050** | **chr 4: 169277885-169458937** | **PALLD** | **2** |
| **3050** | **chr 4: 169277885-169458937** | **DDX60L** | **2** |
| **3051** | **chr 4: 169418216-169849608** | **PALLD** | **3** |
| **3052** | **chr 4: 170907747-170954182** | **MFAP3L** | **1** |
| **3053** | **chr 4: 172733404-173962710** | **GALNTL6** | **3** |
| **3054** | **chr 4: 176554084-176923815** | **GPM6A** | **1** |
| **3055** | **chr 4: 177590771-177627299** | **VEGFC** | **2** |
| **3056** | **chr 4: 177604688-177713881** | **VEGFC** | **2** |
| **3057** | **chr 4: 181652316-181680191** | **none** | **1** |
| **3058** | **chr 4: 181985241-182080302** | **LINC00290** | **5** |
| **3059** | **chr 4: 183065139-183724177** | **MIR1305** | **3** |
| **3059** | **chr 4: 183065139-183724177** | **MGC45800** | **3** |
| **3060** | **chr 4: 183811212-183839089** | **DCTD** | **2** |
| **3061** | **chr 4: 184020445-184241930** | **WWC2-AS2** | **1** |
| **3061** | **chr 4: 184020445-184241930** | **WWC2** | **1** |
| **3062** | **chr 4: 184774583-184944679** | **STOX2** | **1** |
| **3063** | **chr 4: 186366335-186392913** | **C4ORF47** | **1** |
| **3063** | **chr 4: 186366335-186392913** | **CCDC110** | **1** |
| **3064** | **chr 4: 186506597-186877806** | **SORBS2** | **2** |
| **3065** | **chr 4: 187025572-187093821** | **FAM149A** | **1** |
| **3066** | **chr 4: 187207247-187476721** | **F11** | **1** |
| **3066** | **chr 4: 187207247-187476721** | **LOC285441** | **1** |
| **3067** | **chr 4: 190701489-190861426** | **FRG1** | **1** |
| **3068** | **chr 5: 140372-190085** | **PLEKHG4B** | **1** |
| **3069** | **chr 5: 271735-438406** | **PDCD6** | **4** |
| **3070** | **chr 5: 987294-997423** | **LOC100506688** | **2** |
| **3071** | **chr 5: 1050498-1112150** | **SLC12A7** | **6** |
| **3071** | **chr 5: 1050498-1112150** | **MIR4635** | **6** |
| **3072** | **chr 5: 4452042-4866334** | **none** | **2** |
| **3073** | **chr 5: 4640843-4648367** | **none** | **1** |
| **3074** | **chr 5: 5034471-5070117** | **LOC340094** | **1** |
| **3075** | **chr 5: 6582248-6588612** | **LOC255167** | **1** |
| **3076** | **chr 5: 6686437-6707824** | **SRD5A1** | **1** |
| **3076** | **chr 5: 6686437-6707824** | **PAPD7** | **1** |
| **3077** | **chr 5: 6933782-7190925** | **none** | **1** |
| **3078** | **chr 5: 7396320-7830194** | **ADCY2** | **2** |
| **3079** | **chr 5: 7851298-7906138** | **C5ORF49** | **2** |
| **3079** | **chr 5: 7851298-7906138** | **FASTKD3** | **2** |
| **3080** | **chr 5: 8333595-8457677** | **LOC729506** | **4** |
| **3080** | **chr 5: 8333595-8457677** | **LOC100505738** | **4** |
| **3081** | **chr 5: 9035137-9546187** | **MIR4636** | **1** |
| **3081** | **chr 5: 9035137-9546187** | **SEMA5A** | **1** |
| **3082** | **chr 5: 10971951-11904155** | **CTNND2** | **5** |
| **3083** | **chr 5: 12914179-13032998** | **none** | **1** |
| **3084** | **chr 5: 14143810-14532235** | **TRIO** | **2** |
| **3085** | **chr 5: 15500304-15939900** | **FBXL7** | **3** |
| **3086** | **chr 5: 16067247-16180871** | **MARCH11** | **1** |
| **3087** | **chr 5: 16665394-16936372** | **MYO10** | **1** |
| **3088** | **chr 5: 17065706-17276943** | **BASP1** | **2** |
| **3088** | **chr 5: 17065706-17276943** | **LOC285696** | **2** |
| **3089** | **chr 5: 17369333-17375686** | **LOC401177** | **1** |
| **3090** | **chr 5: 17379014-17387419** | **LOC401177** | **2** |
| **3091** | **chr 5: 18965969-19142455** | **none** | **1** |
| **3092** | **chr 5: 19473059-20575982** | **CDH18** | **3** |
| **3093** | **chr 5: 20611948-20937800** | **none** | **2** |
| **3094** | **chr 5: 21616370-21779631** | **CDH12** | **2** |
| **3095** | **chr 5: 21750781-22853731** | **CDH12** | **5** |
| **3095** | **chr 5: 21750781-22853731** | **PMCHL1** | **5** |
| **3096** | **chr 5: 23951456-24178372** | **none** | **1** |
| **3097** | **chr 5: 24487208-24645087** | **CDH10** | **1** |
| **3098** | **chr 5: 24554126-24613331** | **CDH10** | **1** |
| **3099** | **chr 5: 26880708-27121257** | **CDH9** | **1** |
| **3100** | **chr 5: 31094083-31267717** | **CDH6** | **4** |
| **3101** | **chr 5: 31193856-31329253** | **CDH6** | **4** |
| **3102** | **chr 5: 31400603-31532303** | **C5ORF22** | **1** |
| **3102** | **chr 5: 31400603-31532303** | **DROSHA** | **1** |
| **3103** | **chr 5: 31639516-32111037** | **MIR4279** | **3** |
| **3103** | **chr 5: 31639516-32111037** | **PDZD2** | **3** |
| **3104** | **chr 5: 32689175-32791819** | **NPR3** | **1** |
| **3105** | **chr 5: 32925744-33298016** | **LOC340113** | **3** |
| **3106** | **chr 5: 33229840-33255659** | **none** | **1** |
| **3107** | **chr 5: 33440801-33469644** | **TARS** | **1** |
| **3108** | **chr 5: 33523639-33892297** | **ADAMTS12** | **3** |
| **3109** | **chr 5: 34656341-34832732** | **RAI14** | **1** |
| **3110** | **chr 5: 36666315-36725297** | **SLC1A3** | **1** |
| **3111** | **chr 5: 37379313-37753537** | **WDR70** | **1** |
| **3112** | **chr 5: 37840539-37875901** | **GDNF** | **1** |
| **3113** | **chr 5: 37899464-37920971** | **none** | **1** |
| **3114** | **chr 5: 38025798-38184034** | **none** | **1** |
| **3115** | **chr 5: 38258510-38465123** | **EGFLAM-AS4** | **1** |
| **3115** | **chr 5: 38258510-38465123** | **EGFLAM** | **1** |
| **3116** | **chr 5: 38475064-38608456** | **LIFR** | **1** |
| **3117** | **chr 5: 43065277-43192123** | **ZNF131** | **1** |
| **3117** | **chr 5: 43065277-43192123** | **LOC100132356** | **1** |
| **3118** | **chr 5: 43192172-43280952** | **NIM1** | **1** |
| **3119** | **chr 5: 43486802-43515247** | **C5ORF34** | **1** |
| **3120** | **chr 5: 45259348-45696253** | **HCN1** | **5** |
| **3121** | **chr 5: 50678920-50690564** | **LOC642366** | **1** |
| **3121** | **chr 5: 50678920-50690564** | **ISL1** | **1** |
| **3122** | **chr 5: 53179774-53606412** | **MIR581** | **1** |
| **3122** | **chr 5: 53179774-53606412** | **ARL15** | **1** |
| **3123** | **chr 5: 54408798-54469005** | **GPX8** | **1** |
| **3123** | **chr 5: 54408798-54469005** | **CDC20B** | **1** |
| **3124** | **chr 5: 54603587-54721409** | **SKIV2L2** | **1** |
| **3124** | **chr 5: 54603587-54721409** | **DHX29** | **1** |
| **3125** | **chr 5: 54720681-54830878** | **SKIV2L2** | **1** |
| **3125** | **chr 5: 54720681-54830878** | **PPAP2A** | **1** |
| **3126** | **chr 5: 55147206-55218678** | **IL31RA** | **2** |
| **3127** | **chr 5: 55230922-55290821** | **IL6ST** | **1** |
| **3128** | **chr 5: 56111400-56191979** | **MAP3K1** | **1** |
| **3129** | **chr 5: 56137842-56157991** | **MAP3K1** | **1** |
| **3130** | **chr 5: 56469774-56560506** | **GPBP1** | **1** |
| **3131** | **chr 5: 58264864-59817947** | **PDE4D** | **8** |
| **3132** | **chr 5: 60169657-60240900** | **ERCC8** | **1** |
| **3132** | **chr 5: 60169657-60240900** | **NDUFAF2** | **1** |
| **3133** | **chr 5: 60240955-60448853** | **ERCC8** | **1** |
| **3133** | **chr 5: 60240955-60448853** | **NDUFAF2** | **1** |
| **3134** | **chr 5: 60628099-60841997** | **ZSWIM6** | **1** |
| **3135** | **chr 5: 61601988-61924409** | **DIMT1** | **1** |
| **3135** | **chr 5: 61601988-61924409** | **KIF2A** | **1** |
| **3136** | **chr 5: 63253719-63276870** | **HTR1A** | **1** |
| **3137** | **chr 5: 63461670-63668696** | **RNF180** | **1** |
| **3138** | **chr 5: 63802083-63908139** | **RGS7BP** | **1** |
| **3139** | **chr 5: 64064756-64314590** | **CWC27** | **2** |
| **3139** | **chr 5: 64064756-64314590** | **SREK1IP1** | **2** |
| **3140** | **chr 5: 64961754-65018862** | **SGTB** | **1** |
| **3140** | **chr 5: 64961754-65018862** | **TRAPPC13** | **1** |
| **3141** | **chr 5: 65018022-65167553** | **SGTB** | **1** |
| **3141** | **chr 5: 65018022-65167553** | **NLN** | **1** |
| **3142** | **chr 5: 65892175-66465423** | **MAST4** | **3** |
| **3143** | **chr 5: 66675205-67101066** | **none** | **1** |
| **3144** | **chr 5: 67511547-67597649** | **PIK3R1** | **1** |
| **3145** | **chr 5: 68576001-68628636** | **CCDC125** | **1** |
| **3146** | **chr 5: 70647681-70742169** | **PMCHL2** | **2** |
| **3147** | **chr 5: 72112138-72212560** | **TNPO1** | **1** |
| **3148** | **chr 5: 72509773-72590761** | **TMEM174** | **2** |
| **3149** | **chr 5: 73379892-73399041** | **none** | **1** |
| **3150** | **chr 5: 73665200-73832801** | **none** | **2** |
| **3151** | **chr 5: 75378996-75649764** | **SV2C** | **2** |
| **3152** | **chr 5: 75699073-76003957** | **F2RL2** | **1** |
| **3152** | **chr 5: 75699073-76003957** | **IQGAP2** | **1** |
| **3153** | **chr 5: 76506273-76725632** | **PDE8B** | **1** |
| **3154** | **chr 5: 77656338-77776562** | **SCAMP1** | **1** |
| **3155** | **chr 5: 77781037-78065844** | **LHFPL2** | **1** |
| **3156** | **chr 5: 78073031-78281910** | **ARSB** | **1** |
| **3157** | **chr 5: 78532011-78623038** | **JMY** | **1** |
| **3158** | **chr 5: 78668458-78810040** | **HOMER1** | **1** |
| **3159** | **chr 5: 79287133-79379110** | **THBS4** | **1** |
| **3159** | **chr 5: 79287133-79379110** | **MTX3** | **1** |
| **3160** | **chr 5: 79348196-79379477** | **THBS4** | **1** |
| **3161** | **chr 5: 79407049-79551898** | **SERINC5** | **4** |
| **3162** | **chr 5: 79950466-80172279** | **DHFR** | **2** |
| **3163** | **chr 5: 80256490-80525975** | **RNU5E-1** | **1** |
| **3163** | **chr 5: 80256490-80525975** | **RASGRF2** | **1** |
| **3164** | **chr 5: 80597408-80609116** | **RNU5E-1** | **1** |
| **3164** | **chr 5: 80597408-80609116** | **RNU5D-1** | **1** |
| **3165** | **chr 5: 82348664-82373682** | **TMEM167A** | **1** |
| **3165** | **chr 5: 82348664-82373682** | **SCARNA18** | **1** |
| **3166** | **chr 5: 82933623-83017432** | **HAPLN1** | **1** |
| **3167** | **chr 5: 87972035-88018648** | **LINC00461** | **2** |
| **3168** | **chr 5: 87988461-87989789** | **MEF2C** | **2** |
| **3168** | **chr 5: 87988461-87989789** | **MIR9-2** | **2** |
| **3169** | **chr 5: 88179144-88762215** | **MEF2C** | **2** |
| **3170** | **chr 5: 89240726-89243390** | **none** | **1** |
| **3171** | **chr 5: 89825160-90460038** | **LYSMD3** | **2** |
| **3171** | **chr 5: 89825160-90460038** | **GPR98** | **2** |
| **3172** | **chr 5: 91378413-91815133** | **none** | **1** |
| **3173** | **chr 5: 91745962-91996570** | **none** | **1** |
| **3174** | **chr 5: 92953774-93447404** | **FAM172A** | **1** |
| **3174** | **chr 5: 92953774-93447404** | **MIR548AO** | **1** |
| **3175** | **chr 5: 93488670-93954309** | **KIAA0825** | **2** |
| **3176** | **chr 5: 94039445-94620279** | **MCTP1** | **2** |
| **3177** | **chr 5: 95297704-96115299** | **MIR583** | **3** |
| **3177** | **chr 5: 95297704-96115299** | **ELL2** | **3** |
| **3178** | **chr 5: 96271097-96373219** | **LNPEP** | **1** |
| **3179** | **chr 5: 96424778-96772921** | **LIX1** | **1** |
| **3179** | **chr 5: 96424778-96772921** | **RIOK2** | **1** |
| **3180** | **chr 5: 102766106-102862319** | **NUDT12** | **4** |
| **3181** | **chr 5: 103718998-104728671** | **RAB9BP1** | **7** |
| **3182** | **chr 5: 107194735-107717799** | **FBXL17** | **1** |
| **3183** | **chr 5: 108083522-108532542** | **FER** | **2** |
| **3184** | **chr 5: 108670409-108745695** | **PJA2** | **1** |
| **3185** | **chr 5: 109624933-110074657** | **SLC25A46** | **1** |
| **3185** | **chr 5: 109624933-110074657** | **TMEM232** | **1** |
| **3186** | **chr 5: 110998317-111333161** | **STARD4-AS1** | **1** |
| **3186** | **chr 5: 110998317-111333161** | **NREP** | **1** |
| **3187** | **chr 5: 111248204-111353006** | **NREP-AS1** | **1** |
| **3187** | **chr 5: 111248204-111353006** | **NREP** | **1** |
| **3188** | **chr 5: 111478137-111755013** | **EPB41L4A-AS1** | **1** |
| **3188** | **chr 5: 111478137-111755013** | **SNORA13** | **1** |
| **3189** | **chr 5: 111563979-111593006** | **EPB41L4A** | **1** |
| **3190** | **chr 5: 112357795-112824527** | **DCP2** | **2** |
| **3190** | **chr 5: 112357795-112824527** | **MCC** | **2** |
| **3191** | **chr 5: 113696641-113832337** | **KCNN2** | **1** |
| **3192** | **chr 5: 114460458-114516243** | **TRIM36** | **1** |
| **3193** | **chr 5: 114602884-114632528** | **CCDC112** | **1** |
| **3194** | **chr 5: 116751204-116881993** | **LOC728342** | **1** |
| **3195** | **chr 5: 117260702-117601730** | **none** | **1** |
| **3196** | **chr 5: 118788137-118972894** | **HSD17B4** | **1** |
| **3197** | **chr 5: 119581142-119669197** | **none** | **1** |
| **3198** | **chr 5: 122179133-122365049** | **PPIC** | **1** |
| **3198** | **chr 5: 122179133-122365049** | **SNX24** | **1** |
| **3199** | **chr 5: 123828467-123872041** | **none** | **1** |
| **3200** | **chr 5: 123972607-124084500** | **ZNF608** | **3** |
| **3201** | **chr 5: 125412492-125615493** | **none** | **1** |
| **3202** | **chr 5: 125695823-125832186** | **GRAMD3** | **1** |
| **3203** | **chr 5: 126112314-126172712** | **LMNB1** | **1** |
| **3204** | **chr 5: 126626522-126801429** | **MEGF10** | **1** |
| **3205** | **chr 5: 127039081-127277326** | **CTXN3** | **1** |
| **3206** | **chr 5: 127276117-127418864** | **FLJ33630** | **1** |
| **3206** | **chr 5: 127276117-127418864** | **SLC12A2** | **1** |
| **3207** | **chr 5: 127593600-127994878** | **FBN2** | **2** |
| **3208** | **chr 5: 127873705-128369335** | **SLC27A6** | **2** |
| **3208** | **chr 5: 127873705-128369335** | **FBN2** | **2** |
| **3209** | **chr 5: 130759613-131132710** | **RAPGEF6** | **1** |
| **3210** | **chr 5: 131746327-131811736** | **C5ORF56** | **2** |
| **3211** | **chr 5: 132211070-132299326** | **AFF4** | **1** |
| **3212** | **chr 5: 132532146-132948255** | **FSTL4** | **3** |
| **3212** | **chr 5: 132532146-132948255** | **MIR1289-2** | **3** |
| **3213** | **chr 5: 133307605-133340824** | **VDAC1** | **1** |
| **3214** | **chr 5: 133484632-133706738** | **PPP2CA** | **1** |
| **3214** | **chr 5: 133484632-133706738** | **SKP1** | **1** |
| **3215** | **chr 5: 133984478-134063513** | **SEC24A** | **1** |
| **3216** | **chr 5: 134240595-134347392** | **PCBD2** | **2** |
| **3216** | **chr 5: 134240595-134347392** | **MIR4461** | **2** |
| **3217** | **chr 5: 134368969-134691744** | **LOC340073** | **3** |
| **3217** | **chr 5: 134368969-134691744** | **PITX1** | **3** |
| **3218** | **chr 5: 134895266-134970564** | **CXCL14** | **2** |
| **3219** | **chr 5: 135548998-135732730** | **TRPC7** | **2** |
| **3220** | **chr 5: 136310986-136934068** | **SPOCK1** | **5** |
| **3221** | **chr 5: 136953188-137071779** | **KLHL3** | **1** |
| **3222** | **chr 5: 137273648-137387650** | **FAM13B** | **2** |
| **3222** | **chr 5: 137273648-137387650** | **PKD2L2** | **2** |
| **3223** | **chr 5: 138282408-138629246** | **SIL1** | **1** |
| **3223** | **chr 5: 138282408-138629246** | **SNHG4** | **1** |
| **3224** | **chr 5: 139026883-139063467** | **CXXC5** | **1** |
| **3225** | **chr 5: 139028583-139029313** | **CXXC5** | **1** |
| **3226** | **chr 5: 140018324-140027370** | **NDUFA2** | **1** |
| **3226** | **chr 5: 140018324-140027370** | **TMCO6** | **1** |
| **3227** | **chr 5: 140019011-140024993** | **NDUFA2** | **1** |
| **3227** | **chr 5: 140019011-140024993** | **TMCO6** | **1** |
| **3228** | **chr 5: 140050378-140071609** | **WDR55** | **1** |
| **3228** | **chr 5: 140050378-140071609** | **DND1** | **1** |
| **3229** | **chr 5: 140710251-140892546** | **PCDHGA1** | **2** |
| **3230** | **chr 5: 141323149-141349304** | **PCDH12** | **1** |
| **3230** | **chr 5: 141323149-141349304** | **RNF14** | **1** |
| **3231** | **chr 5: 141704857-142051566** | **FGF1** | **2** |
| **3231** | **chr 5: 141704857-142051566** | **SPRY4** | **2** |
| **3232** | **chr 5: 142149948-142608576** | **ARHGAP26** | **1** |
| **3232** | **chr 5: 142149948-142608576** | **ARHGAP26-AS1** | **1** |
| **3233** | **chr 5: 142657495-142815077** | **NR3C1** | **1** |
| **3234** | **chr 5: 142985192-143208337** | **MIR5197** | **1** |
| **3234** | **chr 5: 142985192-143208337** | **HMHB1** | **1** |
| **3235** | **chr 5: 143550395-143865249** | **YIPF5** | **2** |
| **3235** | **chr 5: 143550395-143865249** | **KCTD16** | **2** |
| **3236** | **chr 5: 145492600-145562223** | **LARS** | **1** |
| **3237** | **chr 5: 145967935-146464347** | **PPP2R2B** | **3** |
| **3238** | **chr 5: 146770373-146889619** | **DPYSL3** | **2** |
| **3239** | **chr 5: 146967989-147162338** | **JAKMIP2** | **1** |
| **3239** | **chr 5: 146967989-147162338** | **JAKMIP2-AS1** | **1** |
| **3240** | **chr 5: 147647742-147665817** | **SPINK13** | **2** |
| **3241** | **chr 5: 147647869-147763346** | **SPINK7** | **2** |
| **3241** | **chr 5: 147647869-147763346** | **SPINK13** | **2** |
| **3242** | **chr 5: 147830594-148056798** | **HTR4** | **1** |
| **3243** | **chr 5: 148521045-148640105** | **ABLIM3** | **1** |
| **3244** | **chr 5: 148543517-148656368** | **AFAP1L1** | **1** |
| **3244** | **chr 5: 148543517-148656368** | **ABLIM3** | **1** |
| **3245** | **chr 5: 149109860-149234585** | **PPARGC1B** | **1** |
| **3246** | **chr 5: 149340299-149373018** | **SLC26A2** | **1** |
| **3246** | **chr 5: 149340299-149373018** | **TIGD6** | **1** |
| **3247** | **chr 5: 149675905-149718870** | **ARSI** | **1** |
| **3248** | **chr 5: 149865380-149937773** | **NDST1** | **1** |
| **3249** | **chr 5: 150480272-150537443** | **ANXA6** | **1** |
| **3250** | **chr 5: 150560612-150603706** | **CCDC69** | **1** |
| **3251** | **chr 5: 150694538-150727151** | **SLC36A2** | **1** |
| **3252** | **chr 5: 151040656-151066726** | **SPARC** | **1** |
| **3253** | **chr 5: 151056505-151104343** | **SPARC** | **1** |
| **3254** | **chr 5: 151202073-151304403** | **GLRA1** | **2** |
| **3255** | **chr 5: 151329131-151650009** | **GLRA1** | **2** |
| **3256** | **chr 5: 151998524-152603103** | **none** | **1** |
| **3257** | **chr 5: 153418465-153800544** | **FAM114A2** | **3** |
| **3257** | **chr 5: 153418465-153800544** | **MFAP3** | **3** |
| **3258** | **chr 5: 153708996-153825410** | **FLJ38109** | **1** |
| **3258** | **chr 5: 153708996-153825410** | **GALNT10** | **1** |
| **3259** | **chr 5: 155297353-156194799** | **SGCD** | **4** |
| **3260** | **chr 5: 156131068-156166823** | **SGCD** | **1** |
| **3261** | **chr 5: 156456423-156486130** | **HAVCR1** | **2** |
| **3262** | **chr 5: 156693088-156822606** | **CYFIP2** | **1** |
| **3263** | **chr 5: 156789622-157002783** | **CYFIP2** | **1** |
| **3264** | **chr 5: 156887026-156901725** | **NIPAL4** | **1** |
| **3265** | **chr 5: 157158204-157168456** | **THG1L** | **1** |
| **3266** | **chr 5: 157912197-157961446** | **none** | **2** |
| **3267** | **chr 5: 158122927-158526769** | **EBF1** | **5** |
| **3268** | **chr 5: 158584416-158637061** | **RNF145** | **1** |
| **3269** | **chr 5: 158875563-158893284** | **LOC285627** | **1** |
| **3270** | **chr 5: 159203778-159298391** | **ADRA1B** | **4** |
| **3271** | **chr 5: 159678658-159772403** | **CCNJL** | **1** |
| **3272** | **chr 5: 160715435-160976050** | **GABRB2** | **4** |
| **3273** | **chr 5: 160974068-161129599** | **GABRB2** | **1** |
| **3273** | **chr 5: 160974068-161129599** | **GABRA6** | **1** |
| **3274** | **chr 5: 161494545-161582542** | **GABRG2** | **1** |
| **3275** | **chr 5: 162439270-162864332** | **CCNG1** | **3** |
| **3276** | **chr 5: 163723701-164598649** | **none** | **5** |
| **3277** | **chr 5: 164776035-165205694** | **none** | **4** |
| **3278** | **chr 5: 166711803-167691162** | **TENM2** | **18** |
| **3279** | **chr 5: 167718655-167899308** | **WWC1** | **1** |
| **3280** | **chr 5: 168088744-168728133** | **MIR218-2** | **4** |
| **3280** | **chr 5: 168088744-168728133** | **SLIT3** | **4** |
| **3281** | **chr 5: 169064250-169510386** | **DOCK2** | **2** |
| **3281** | **chr 5: 169064250-169510386** | **FAM196B** | **2** |
| **3282** | **chr 5: 169291267-169407744** | **DOCK2** | **1** |
| **3282** | **chr 5: 169291267-169407744** | **FAM196B** | **1** |
| **3283** | **chr 5: 169780490-170163636** | **KCNMB1** | **8** |
| **3283** | **chr 5: 169780490-170163636** | **KCNIP1** | **8** |
| **3284** | **chr 5: 170066161-170108441** | **KCNIP1** | **1** |
| **3285** | **chr 5: 170174050-170215654** | **GABRP** | **1** |
| **3286** | **chr 5: 170288873-170727019** | **RANBP17** | **2** |
| **3287** | **chr 5: 171469076-171615390** | **STK10** | **1** |
| **3288** | **chr 5: 172185228-172189559** | **DUSP1** | **3** |
| **3289** | **chr 5: 172189982-172204777** | **DUSP1** | **1** |
| **3290** | **chr 5: 172261277-172379688** | **ERGIC1** | **1** |
| **3291** | **chr 5: 172741715-172756506** | **STC2** | **1** |
| **3292** | **chr 5: 172890502-172911587** | **none** | **1** |
| **3293** | **chr 5: 173472606-173670504** | **HMP19** | **1** |
| **3294** | **chr 5: 173763297-173959460** | **none** | **5** |
| **3295** | **chr 5: 175487691-175559261** | **FAM153B** | **3** |
| **3295** | **chr 5: 175487691-175559261** | **LOC100507387** | **3** |
| **3296** | **chr 5: 176134356-176153244** | **TSPAN17** | **3** |
| **3297** | **chr 5: 176237477-176307897** | **HK3** | **2** |
| **3297** | **chr 5: 176237477-176307897** | **UNC5A** | **2** |
| **3298** | **chr 5: 176873445-176883283** | **PRR7-AS1** | **1** |
| **3298** | **chr 5: 176873445-176883283** | **PRR7** | **1** |
| **3299** | **chr 5: 177134981-177210399** | **FAM153A** | **3** |
| **3300** | **chr 5: 177433405-177482560** | **FAM153C** | **2** |
| **3301** | **chr 5: 177557996-177577566** | **NHP2** | **1** |
| **3301** | **chr 5: 177557996-177577566** | **RMND5B** | **1** |
| **3302** | **chr 5: 177635497-177659792** | **HNRNPAB** | **1** |
| **3302** | **chr 5: 177635497-177659792** | **AGXT2L2** | **1** |
| **3303** | **chr 5: 177664618-178017556** | **COL23A1** | **3** |
| **3304** | **chr 5: 178191861-178245436** | **AACSP1** | **1** |
| **3305** | **chr 5: 178882415-178883096** | **none** | **1** |
| **3306** | **chr 5: 178930322-178942580** | **RUFY1** | **2** |
| **3307** | **chr 5: 179105628-179157926** | **CANX** | **1** |
| **3307** | **chr 5: 179105628-179157926** | **CBY3** | **1** |
| **3308** | **chr 5: 179527794-179636153** | **RASGEF1C** | **1** |
| **3309** | **chr 5: 179921411-180005405** | **CNOT6** | **1** |
| **3310** | **chr 5: 180649498-180662809** | **MIR4638** | **1** |
| **3310** | **chr 5: 180649498-180662809** | **TRIM41** | **1** |
| **3311** | **chr 6: 1624040-2245926** | **GMDS** | **2** |
| **3312** | **chr 6: 2245981-2482256** | **GMDS** | **1** |
| **3312** | **chr 6: 2245981-2482256** | **LOC100508120** | **1** |
| **3313** | **chr 6: 3064224-3115421** | **RIPK1** | **1** |
| **3314** | **chr 6: 3269195-3457256** | **SLC22A23** | **1** |
| **3315** | **chr 6: 3905143-3912213** | **none** | **1** |
| **3316** | **chr 6: 4706392-4955785** | **CDYL** | **1** |
| **3317** | **chr 6: 5030205-5043683** | **PPP1R3G** | **2** |
| **3317** | **chr 6: 5030205-5043683** | **RPP40** | **2** |
| **3318** | **chr 6: 5031989-5054657** | **PPP1R3G** | **1** |
| **3318** | **chr 6: 5031989-5054657** | **RPP40** | **1** |
| **3319** | **chr 6: 5261276-5771813** | **LYRM4** | **3** |
| **3320** | **chr 6: 6346697-6623004** | **LY86-AS1** | **5** |
| **3320** | **chr 6: 6346697-6623004** | **LY86** | **5** |
| **3321** | **chr 6: 7107829-7252213** | **RREB1** | **1** |
| **3322** | **chr 6: 7268538-7389976** | **SSR1** | **1** |
| **3322** | **chr 6: 7268538-7389976** | **CAGE1** | **1** |
| **3323** | **chr 6: 10492455-10629601** | **GCNT2** | **1** |
| **3324** | **chr 6: 11183530-11382581** | **NEDD9** | **1** |
| **3325** | **chr 6: 12717892-13288645** | **LOC100130357** | **2** |
| **3325** | **chr 6: 12717892-13288645** | **PHACTR1** | **2** |
| **3326** | **chr 6: 13265092-13328815** | **LOC100130357** | **1** |
| **3326** | **chr 6: 13265092-13328815** | **PHACTR1** | **1** |
| **3327** | **chr 6: 14117871-14137149** | **CD83** | **1** |
| **3328** | **chr 6: 16299342-16761722** | **ATXN1** | **1** |
| **3329** | **chr 6: 17393446-17558023** | **CAP2** | **1** |
| **3330** | **chr 6: 17615268-17706656** | **NUP153** | **1** |
| **3331** | **chr 6: 17759413-17987854** | **KIF13A** | **1** |
| **3332** | **chr 6: 18368778-18469105** | **RNF144B** | **1** |
| **3333** | **chr 6: 19535174-19839311** | **ID4** | **1** |
| **3334** | **chr 6: 20100934-20212670** | **MBOAT1** | **1** |
| **3335** | **chr 6: 20534687-21232635** | **CDKAL1** | **2** |
| **3336** | **chr 6: 21665002-22214734** | **LOC729177** | **2** |
| **3336** | **chr 6: 21665002-22214734** | **LINC00340** | **2** |
| **3337** | **chr 6: 24171983-24358280** | **DCDC2** | **1** |
| **3338** | **chr 6: 24544331-24646383** | **KIAA0319** | **1** |
| **3339** | **chr 6: 24797548-25042398** | **FAM65B** | **1** |
| **3340** | **chr 6: 25279305-25620758** | **LRRC16A** | **1** |
| **3341** | **chr 6: 26839262-26924333** | **LINC00240** | **1** |
| **3341** | **chr 6: 26839262-26924333** | **GUSBP2** | **1** |
| **3342** | **chr 6: 26924770-26991703** | **LINC00240** | **2** |
| **3342** | **chr 6: 26924770-26991703** | **GUSBP2** | **2** |
| **3343** | **chr 6: 27106072-27114619** | **HIST1H4I** | **1** |
| **3343** | **chr 6: 27106072-27114619** | **HIST1H2BK** | **1** |
| **3344** | **chr 6: 28089572-28105071** | **ZSCAN16** | **1** |
| **3345** | **chr 6: 28092337-28097860** | **ZSCAN16** | **1** |
| **3346** | **chr 6: 28292469-28324048** | **ZSCAN31** | **1** |
| **3347** | **chr 6: 28317690-28336947** | **ZKSCAN3** | **1** |
| **3348** | **chr 6: 29758730-29765588** | **HCG4** | **2** |
| **3348** | **chr 6: 29758730-29765588** | **LOC554223** | **2** |
| **3349** | **chr 6: 30201815-30294927** | **HCG17** | **1** |
| **3349** | **chr 6: 30201815-30294927** | **HLA-L** | **1** |
| **3350** | **chr 6: 30227360-30260791** | **HCG17** | **1** |
| **3350** | **chr 6: 30227360-30260791** | **HLA-L** | **1** |
| **3351** | **chr 6: 30382491-30387096** | **none** | **1** |
| **3352** | **chr 6: 31082526-31107869** | **PSORS1C1** | **1** |
| **3352** | **chr 6: 31082526-31107869** | **CDSN** | **1** |
| **3353** | **chr 6: 31847535-31865464** | **EHMT2** | **1** |
| **3353** | **chr 6: 31847535-31865464** | **SLC44A4** | **1** |
| **3354** | **chr 6: 31919863-31926887** | **MIR1236** | **1** |
| **3354** | **chr 6: 31919863-31926887** | **NELFE** | **1** |
| **3355** | **chr 6: 32135988-32145873** | **PPT2-EGFL8** | **1** |
| **3355** | **chr 6: 32135988-32145873** | **EGFL8** | **1** |
| **3356** | **chr 6: 32723874-32731311** | **HLA-DQB2** | **1** |
| **3357** | **chr 6: 33032345-33048552** | **HLA-DPA1** | **1** |
| **3358** | **chr 6: 33043702-33054978** | **HLA-DPA1** | **1** |
| **3358** | **chr 6: 33043702-33054978** | **HLA-DPB1** | **1** |
| **3359** | **chr 6: 33098992-33111102** | **HLA-DPB2** | **1** |
| **3359** | **chr 6: 33098992-33111102** | **COL11A2** | **1** |
| **3360** | **chr 6: 33387846-33421466** | **SYNGAP1** | **2** |
| **3360** | **chr 6: 33387846-33421466** | **MIR5004** | **2** |
| **3361** | **chr 6: 33861099-33895408** | **LOC100507584** | **1** |
| **3362** | **chr 6: 33989627-34123399** | **GRM4** | **4** |
| **3363** | **chr 6: 34433915-34503006** | **PACSIN1** | **1** |
| **3364** | **chr 6: 34555064-34664636** | **C6ORF106** | **2** |
| **3365** | **chr 6: 35441373-35464853** | **TEAD3** | **1** |
| **3366** | **chr 6: 36210979-36276372** | **PNPLA1** | **1** |
| **3367** | **chr 6: 36935916-36954074** | **MTCH1** | **1** |
| **3368** | **chr 6: 37225547-37300746** | **TMEM217** | **1** |
| **3369** | **chr 6: 37787274-38122400** | **ZFAND3** | **2** |
| **3370** | **chr 6: 38136226-38607924** | **BTBD9** | **2** |
| **3371** | **chr 6: 39297765-39693181** | **KIF6** | **2** |
| **3372** | **chr 6: 39760141-39872648** | **DAAM2** | **1** |
| **3373** | **chr 6: 39849579-39902290** | **DAAM2** | **1** |
| **3374** | **chr 6: 40239304-40243976** | **none** | **1** |
| **3375** | **chr 6: 41303392-41318625** | **NCR2** | **1** |
| **3376** | **chr 6: 41514163-41570122** | **MIR4641** | **2** |
| **3376** | **chr 6: 41514163-41570122** | **FOXP4** | **2** |
| **3377** | **chr 6: 41604619-41621984** | **MDFI** | **1** |
| **3378** | **chr 6: 41651715-41703997** | **TFEB** | **1** |
| **3379** | **chr 6: 43008514-43042837** | **CUL7** | **1** |
| **3379** | **chr 6: 43008514-43042837** | **MRPL2** | **1** |
| **3380** | **chr 6: 43211417-43255997** | **TTBK1** | **1** |
| **3381** | **chr 6: 43477439-43497323** | **YIPF3** | **1** |
| **3381** | **chr 6: 43477439-43497323** | **LRRC73** | **1** |
| **3382** | **chr 6: 43490071-43543812** | **XPO5** | **1** |
| **3382** | **chr 6: 43490071-43543812** | **POLH** | **1** |
| **3383** | **chr 6: 43963459-44042389** | **C6ORF223** | **2** |
| **3384** | **chr 6: 43968316-43973695** | **C6ORF223** | **1** |
| **3385** | **chr 6: 44094650-44123256** | **TMEM63B** | **2** |
| **3385** | **chr 6: 44094650-44123256** | **MRPL14** | **2** |
| **3386** | **chr 6: 44126547-44152139** | **CAPN11** | **1** |
| **3387** | **chr 6: 44187241-44201888** | **SLC29A1** | **1** |
| **3388** | **chr 6: 44238202-44346694** | **TMEM151B** | **1** |
| **3388** | **chr 6: 44238202-44346694** | **TCTE1** | **1** |
| **3389** | **chr 6: 45295893-45632086** | **SUPT3H** | **8** |
| **3390** | **chr 6: 46459788-46500495** | **RCAN2** | **1** |
| **3391** | **chr 6: 46517540-46620523** | **CYP39A1** | **2** |
| **3391** | **chr 6: 46517540-46620523** | **SLC25A27** | **2** |
| **3392** | **chr 6: 47445524-47594999** | **CD2AP** | **2** |
| **3393** | **chr 6: 47845763-48036425** | **PTCHD4** | **1** |
| **3394** | **chr 6: 49431090-49460820** | **MUT** | **1** |
| **3394** | **chr 6: 49431090-49460820** | **CENPQ** | **1** |
| **3395** | **chr 6: 49533636-49547879** | **RHAG** | **1** |
| **3395** | **chr 6: 49533636-49547879** | **C6ORF141** | **1** |
| **3396** | **chr 6: 50786435-50815326** | **TFAP2B** | **2** |
| **3397** | **chr 6: 51480097-51952423** | **PKHD1** | **3** |
| **3398** | **chr 6: 53132195-53213947** | **ELOVL5** | **2** |
| **3398** | **chr 6: 53132195-53213947** | **MIR5685** | **2** |
| **3399** | **chr 6: 53362138-53481969** | **GCLC** | **1** |
| **3400** | **chr 6: 53794779-54131078** | **MLIP-IT1** | **1** |
| **3400** | **chr 6: 53794779-54131078** | **MLIP** | **1** |
| **3401** | **chr 6: 55192266-55267291** | **GFRAL** | **1** |
| **3402** | **chr 6: 55299166-55444012** | **HMGCLL1** | **3** |
| **3403** | **chr 6: 55921387-56258892** | **COL21A1** | **1** |
| **3403** | **chr 6: 55921387-56258892** | **RNU6-71** | **1** |
| **3404** | **chr 6: 57179602-57513375** | **PRIM2** | **1** |
| **3405** | **chr 6: 62340137-62996132** | **KHDRBS2** | **1** |
| **3406** | **chr 6: 64345724-64489229** | **EYS** | **1** |
| **3406** | **chr 6: 64345724-64489229** | **PHF3** | **1** |
| **3407** | **chr 6: 64429875-66417118** | **EYS** | **5** |
| **3407** | **chr 6: 64429875-66417118** | **LOC441155** | **5** |
| **3408** | **chr 6: 69345258-70099403** | **BAI3** | **7** |
| **3409** | **chr 6: 73331519-73908574** | **KCNQ5** | **1** |
| **3409** | **chr 6: 73331519-73908574** | **MIR4282** | **1** |
| **3410** | **chr 6: 74123237-74161999** | **DDX43** | **2** |
| **3410** | **chr 6: 74123237-74161999** | **MB21D1** | **2** |
| **3411** | **chr 6: 74233340-74280319** | **SLC17A5** | **1** |
| **3411** | **chr 6: 74233340-74280319** | **EEF1A1** | **1** |
| **3412** | **chr 6: 74779166-75400443** | **none** | **2** |
| **3413** | **chr 6: 76001574-76203454** | **FILIP1** | **1** |
| **3413** | **chr 6: 76001574-76203454** | **LOC100506804** | **1** |
| **3414** | **chr 6: 76066929-76096096** | **FILIP1** | **1** |
| **3415** | **chr 6: 80816363-81055987** | **BCKDHB** | **2** |
| **3416** | **chr 6: 83602116-83775560** | **UBE3D** | **1** |
| **3417** | **chr 6: 83920107-84140797** | **ME1** | **1** |
| **3418** | **chr 6: 84569361-84677143** | **CYB5R4** | **1** |
| **3419** | **chr 6: 86159808-86205500** | **NT5E** | **1** |
| **3420** | **chr 6: 88117700-88222054** | **C6ORF165** | **3** |
| **3420** | **chr 6: 88117700-88222054** | **SLC35A1** | **3** |
| **3421** | **chr 6: 89319984-89673348** | **RNGTT** | **2** |
| **3422** | **chr 6: 90036343-90062567** | **UBE2J1** | **1** |
| **3423** | **chr 6: 90142888-90343553** | **ANKRD6** | **2** |
| **3424** | **chr 6: 90636247-91006627** | **BACH2** | **1** |
| **3425** | **chr 6: 91223291-91296764** | **MAP3K7** | **1** |
| **3426** | **chr 6: 93949737-94129265** | **EPHA7** | **1** |
| **3427** | **chr 6: 96463859-96663488** | **FUT9** | **1** |
| **3428** | **chr 6: 96969470-97003152** | **UFL1** | **1** |
| **3429** | **chr 6: 97372604-97588630** | **MIR548H3** | **1** |
| **3429** | **chr 6: 97372604-97588630** | **KLHL32** | **1** |
| **3430** | **chr 6: 98264584-98847748** | **MIR2113** | **3** |
| **3431** | **chr 6: 99316419-99395849** | **FBXL4** | **2** |
| **3432** | **chr 6: 99817275-99842080** | **COQ3** | **1** |
| **3433** | **chr 6: 100441819-100524289** | **MCHR2** | **1** |
| **3434** | **chr 6: 101846663-102517958** | **GRIK2** | **4** |
| **3435** | **chr 6: 105725439-105850959** | **PREP** | **2** |
| **3436** | **chr 6: 106060541-106080071** | **none** | **1** |
| **3437** | **chr 6: 108616097-108847999** | **LACE1** | **2** |
| **3438** | **chr 6: 108881037-109005977** | **FOXO3** | **2** |
| **3439** | **chr 6: 109307639-109416022** | **SESN1** | **1** |
| **3440** | **chr 6: 109487035-109592217** | **CCDC162P** | **1** |
| **3440** | **chr 6: 109487035-109592217** | **CEP57L1** | **1** |
| **3441** | **chr 6: 109711417-109762374** | **PPIL6** | **1** |
| **3441** | **chr 6: 109711417-109762374** | **SMPD2** | **1** |
| **3442** | **chr 6: 110012498-110146631** | **AKD1** | **1** |
| **3443** | **chr 6: 110745889-110797844** | **SLC22A16** | **1** |
| **3444** | **chr 6: 110931180-111137161** | **CDK19** | **1** |
| **3445** | **chr 6: 111620233-111804918** | **TRAF3IP2-AS1** | **1** |
| **3445** | **chr 6: 111620233-111804918** | **REV3L** | **1** |
| **3446** | **chr 6: 111981534-112194655** | **FYN** | **2** |
| **3447** | **chr 6: 112557293-112672498** | **LAMA4** | **2** |
| **3448** | **chr 6: 112797739-112802838** | **none** | **4** |
| **3449** | **chr 6: 114254191-114332472** | **HDAC2** | **2** |
| **3450** | **chr 6: 114290864-114792869** | **HDAC2** | **3** |
| **3451** | **chr 6: 114376749-114664209** | **HS3ST5** | **2** |
| **3452** | **chr 6: 116252311-116381921** | **FRK** | **1** |
| **3452** | **chr 6: 116252311-116381921** | **TPI1P3** | **1** |
| **3453** | **chr 6: 116422011-116570660** | **COL10A1** | **1** |
| **3453** | **chr 6: 116422011-116570660** | **NT5DC1** | **1** |
| **3454** | **chr 6: 116575335-116762424** | **TSPYL4** | **1** |
| **3454** | **chr 6: 116575335-116762424** | **TSPYL1** | **1** |
| **3455** | **chr 6: 116816151-116866773** | **BET3L** | **1** |
| **3455** | **chr 6: 116816151-116866773** | **FAM26E** | **1** |
| **3456** | **chr 6: 116850173-116880031** | **BET3L** | **1** |
| **3457** | **chr 6: 117609462-117923691** | **DCBLD1** | **1** |
| **3457** | **chr 6: 117609462-117923691** | **ROS1** | **1** |
| **3458** | **chr 6: 117774979-117891021** | **DCBLD1** | **1** |
| **3458** | **chr 6: 117774979-117891021** | **GOPC** | **1** |
| **3459** | **chr 6: 123537482-123958238** | **TRDN** | **1** |
| **3460** | **chr 6: 124125285-125146803** | **NKAIN2** | **1** |
| **3461** | **chr 6: 125283690-125413779** | **STL** | **1** |
| **3461** | **chr 6: 125283690-125413779** | **RNF217** | **1** |
| **3462** | **chr 6: 125440194-125585553** | **TPD52L1** | **1** |
| **3463** | **chr 6: 125541107-125623282** | **TPD52L1** | **1** |
| **3464** | **chr 6: 127759550-127840500** | **SOGA3** | **1** |
| **3464** | **chr 6: 127759550-127840500** | **KIAA0408** | **1** |
| **3465** | **chr 6: 128289923-128841870** | **PTPRK** | **2** |
| **3466** | **chr 6: 128349030-128406393** | **PTPRK** | **1** |
| **3467** | **chr 6: 129204341-129837714** | **LAMA2** | **6** |
| **3468** | **chr 6: 129897276-130031370** | **ARHGAP18** | **1** |
| **3469** | **chr 6: 130454554-130686570** | **L3MBTL3** | **2** |
| **3469** | **chr 6: 130454554-130686570** | **SAMD3** | **2** |
| **3470** | **chr 6: 132223102-132241705** | **CTGF** | **1** |
| **3470** | **chr 6: 132223102-132241705** | **ENPP1** | **1** |
| **3471** | **chr 6: 132617193-132722684** | **MOXD1** | **1** |
| **3472** | **chr 6: 133065008-133084598** | **VNN2** | **1** |
| **3473** | **chr 6: 133823389-134213074** | **EYA4** | **1** |
| **3473** | **chr 6: 133823389-134213074** | **MGC34034** | **1** |
| **3474** | **chr 6: 134490383-134639250** | **SGK1** | **2** |
| **3475** | **chr 6: 134846451-134980974** | **LOC154092** | **2** |
| **3476** | **chr 6: 135027197-135028487** | **none** | **1** |
| **3477** | **chr 6: 135818488-136037193** | **AHI1** | **2** |
| **3478** | **chr 6: 136172833-136516712** | **PDE7B** | **2** |
| **3479** | **chr 6: 136415852-136546733** | **PDE7B** | **1** |
| **3480** | **chr 6: 136878184-137113656** | **MAP3K5** | **1** |
| **3481** | **chr 6: 138188350-138204449** | **LOC100130476** | **1** |
| **3481** | **chr 6: 138188350-138204449** | **TNFAIP3** | **1** |
| **3482** | **chr 6: 138266502-138293659** | **none** | **3** |
| **3483** | **chr 6: 138483057-138665800** | **PBOV1** | **1** |
| **3483** | **chr 6: 138483057-138665800** | **KIAA1244** | **1** |
| **3484** | **chr 6: 138743179-139013708** | **NHSL1** | **1** |
| **3485** | **chr 6: 139224629-139309398** | **REPS1** | **1** |
| **3485** | **chr 6: 139224629-139309398** | **ECT2L** | **1** |
| **3486** | **chr 6: 139465340-139613276** | **HECA** | **1** |
| **3486** | **chr 6: 139465340-139613276** | **TXLNB** | **1** |
| **3487** | **chr 6: 143072603-143266338** | **HIVEP2** | **1** |
| **3488** | **chr 6: 143857981-144184949** | **LOC285740** | **1** |
| **3489** | **chr 6: 144261436-144385735** | **PLAGL1** | **1** |
| **3490** | **chr 6: 144471662-144509507** | **STX11** | **3** |
| **3491** | **chr 6: 144606836-145174170** | **UTRN** | **1** |
| **3492** | **chr 6: 145822718-146057160** | **EPM2A** | **1** |
| **3492** | **chr 6: 145822718-146057160** | **LOC100507557** | **1** |
| **3493** | **chr 6: 146056705-146058354** | **EPM2A** | **1** |
| **3493** | **chr 6: 146056705-146058354** | **LOC100507557** | **1** |
| **3494** | **chr 6: 147830062-148275073** | **SAMD5** | **2** |
| **3495** | **chr 6: 148558720-148604544** | **none** | **1** |
| **3496** | **chr 6: 148593439-148873186** | **SASH1** | **3** |
| **3497** | **chr 6: 150464211-150571493** | **PPP1R14C** | **2** |
| **3498** | **chr 6: 150690027-150727105** | **IYD** | **1** |
| **3499** | **chr 6: 150920998-151164799** | **PLEKHG1** | **1** |
| **3500** | **chr 6: 151409238-151549582** | **MTHFD1L** | **2** |
| **3501** | **chr 6: 151561133-151679692** | **AKAP12** | **1** |
| **3502** | **chr 6: 151815164-151942328** | **CCDC170** | **3** |
| **3503** | **chr 6: 151977825-152450754** | **ESR1** | **1** |
| **3504** | **chr 6: 152442818-152958936** | **SYNE1** | **4** |
| **3505** | **chr 6: 153325593-153452384** | **RGS17** | **1** |
| **3506** | **chr 6: 153552454-153668623** | **none** | **1** |
| **3507** | **chr 6: 154331630-154568001** | **OPRM1** | **2** |
| **3508** | **chr 6: 154475630-154677926** | **OPRM1** | **1** |
| **3509** | **chr 6: 154708637-154831793** | **CNKSR3** | **2** |
| **3510** | **chr 6: 155054458-155578857** | **TIAM2** | **3** |
| **3510** | **chr 6: 155054458-155578857** | **SCAF8** | **3** |
| **3511** | **chr 6: 157099062-157531913** | **ARID1B** | **1** |
| **3511** | **chr 6: 157099062-157531913** | **MIR4466** | **1** |
| **3512** | **chr 6: 157802164-158099178** | **ZDHHC14** | **3** |
| **3512** | **chr 6: 157802164-158099178** | **MIR3692** | **3** |
| **3513** | **chr 6: 158244295-158366109** | **SNX9** | **2** |
| **3514** | **chr 6: 161412758-161538417** | **MAP3K4** | **1** |
| **3515** | **chr 6: 161768451-163148803** | **PACRG** | **7** |
| **3515** | **chr 6: 161768451-163148803** | **PARK2** | **7** |
| **3516** | **chr 6: 163148163-163736524** | **PACRG** | **8** |
| **3516** | **chr 6: 163148163-163736524** | **PARK2** | **8** |
| **3517** | **chr 6: 165740775-166401536** | **LINC00473** | **2** |
| **3517** | **chr 6: 165740775-166401536** | **PDE10A** | **2** |
| **3518** | **chr 6: 166822851-167370679** | **RPS6KA2** | **3** |
| **3519** | **chr 6: 168396920-168445769** | **KIF25** | **2** |
| **3520** | **chr 6: 168595037-168605181** | **none** | **1** |
| **3521** | **chr 6: 168841830-169073984** | **SMOC2** | **2** |
| **3522** | **chr 6: 169613348-169639660** | **THBS2** | **1** |
| **3523** | **chr 6: 169770092-169788480** | **none** | **1** |
| **3524** | **chr 6: 170599790-170716153** | **FAM120B** | **1** |
| **3524** | **chr 6: 170599790-170716153** | **DLL1** | **1** |
| **3525** | **chr 6: 171034284-171037576** | **none** | **1** |
| **3526** | **chr 7: 588833-767287** | **PRKAR1B** | **1** |
| **3527** | **chr 7: 937539-1015235** | **ADAP1** | **3** |
| **3527** | **chr 7: 937539-1015235** | **COX19** | **3** |
| **3528** | **chr 7: 1509912-1545489** | **INTS1** | **3** |
| **3529** | **chr 7: 1581870-1600457** | **TMEM184A** | **1** |
| **3529** | **chr 7: 1581870-1600457** | **MAFK** | **1** |
| **3530** | **chr 7: 2291404-2393953** | **EIF3B** | **1** |
| **3530** | **chr 7: 2291404-2393953** | **SNX8** | **1** |
| **3531** | **chr 7: 2767745-2883958** | **GNA12** | **1** |
| **3532** | **chr 7: 3341079-4308632** | **SDK1** | **7** |
| **3533** | **chr 7: 5313999-5346501** | **TNRC18** | **2** |
| **3533** | **chr 7: 5313999-5346501** | **SLC29A4** | **2** |
| **3534** | **chr 7: 5346420-5465045** | **TNRC18** | **2** |
| **3535** | **chr 7: 5566781-5603415** | **ACTB** | **1** |
| **3536** | **chr 7: 5659677-5821370** | **RNF216-IT1** | **1** |
| **3536** | **chr 7: 5659677-5821370** | **RNF216** | **1** |
| **3537** | **chr 7: 6414153-6443608** | **RAC1** | **1** |
| **3538** | **chr 7: 6749758-6793493** | **PMS2CL** | **1** |
| **3538** | **chr 7: 6749758-6793493** | **RSPH10B** | **1** |
| **3539** | **chr 7: 6895115-6899461** | **CCZ1B** | **1** |
| **3540** | **chr 7: 7196564-7288282** | **C1GALT1** | **1** |
| **3541** | **chr 7: 7395833-7575484** | **COL28A1** | **3** |
| **3542** | **chr 7: 8152813-8302317** | **ICA1** | **1** |
| **3543** | **chr 7: 8153654-8156191** | **ICA1** | **1** |
| **3544** | **chr 7: 8473584-8792593** | **NXPH1** | **1** |
| **3545** | **chr 7: 11013498-11209250** | **PHF14** | **1** |
| **3546** | **chr 7: 13141015-13743774** | **none** | **4** |
| **3547** | **chr 7: 14184673-15014402** | **DGKB** | **1** |
| **3548** | **chr 7: 15239942-15601640** | **AGMO** | **1** |
| **3549** | **chr 7: 16130816-16460947** | **ISPD** | **2** |
| **3549** | **chr 7: 16130816-16460947** | **LOC100506025** | **2** |
| **3550** | **chr 7: 16566504-16621193** | **LRRC72** | **2** |
| **3551** | **chr 7: 19060613-19157295** | **TWIST1** | **2** |
| **3552** | **chr 7: 19393156-19618229** | **none** | **1** |
| **3553** | **chr 7: 19758932-19813221** | **TMEM196** | **1** |
| **3554** | **chr 7: 20654829-20816658** | **ABCB5** | **4** |
| **3555** | **chr 7: 20875049-21062767** | **RPL23P8** | **3** |
| **3556** | **chr 7: 21582832-21941457** | **CDCA7L** | **3** |
| **3556** | **chr 7: 21582832-21941457** | **DNAH11** | **3** |
| **3557** | **chr 7: 22157855-22396763** | **RAPGEF5** | **2** |
| **3558** | **chr 7: 22459062-22705152** | **STEAP1B** | **3** |
| **3559** | **chr 7: 22928989-22980809** | **FAM126A** | **1** |
| **3559** | **chr 7: 22928989-22980809** | **SNORD93** | **1** |
| **3560** | **chr 7: 23349827-23510086** | **IGF2BP3** | **1** |
| **3561** | **chr 7: 23544398-23571660** | **TRA2A** | **1** |
| **3562** | **chr 7: 24737971-24809244** | **DFNA5** | **1** |
| **3563** | **chr 7: 24836157-25021253** | **OSBPL3** | **2** |
| **3564** | **chr 7: 25174315-25219975** | **C7ORF31** | **1** |
| **3565** | **chr 7: 26331540-26413949** | **SNX10** | **1** |
| **3566** | **chr 7: 26706680-27034858** | **SKAP2** | **1** |
| **3567** | **chr 7: 27139720-27142430** | **HOTAIRM1** | **1** |
| **3567** | **chr 7: 27139720-27142430** | **HOXA2** | **1** |
| **3568** | **chr 7: 27202053-27219880** | **HOXA10** | **2** |
| **3568** | **chr 7: 27202053-27219880** | **HOXA9** | **2** |
| **3569** | **chr 7: 27208237-27211534** | **HOXA-AS4** | **1** |
| **3569** | **chr 7: 27208237-27211534** | **HOXA10** | **1** |
| **3570** | **chr 7: 28338939-28865511** | **CREB5** | **2** |
| **3571** | **chr 7: 29019582-29052983** | **LOC100506497** | **1** |
| **3571** | **chr 7: 29019582-29052983** | **CPVL** | **1** |
| **3572** | **chr 7: 29034846-29235067** | **LOC100506497** | **2** |
| **3572** | **chr 7: 29034846-29235067** | **CPVL** | **2** |
| **3573** | **chr 7: 29161889-29553944** | **CHN2** | **3** |
| **3573** | **chr 7: 29161889-29553944** | **CPVL** | **3** |
| **3574** | **chr 7: 29239155-29248586** | **CHN2** | **2** |
| **3575** | **chr 7: 29689842-29782210** | **MIR550A3** | **1** |
| **3575** | **chr 7: 29689842-29782210** | **LOC646762** | **1** |
| **3576** | **chr 7: 29846101-29956682** | **WIPF3** | **2** |
| **3577** | **chr 7: 29959718-30029905** | **SCRN1** | **1** |
| **3578** | **chr 7: 30464142-30518400** | **NOD1** | **1** |
| **3579** | **chr 7: 30634296-30673649** | **GARS** | **1** |
| **3580** | **chr 7: 30737600-30965131** | **CRHR2** | **1** |
| **3580** | **chr 7: 30737600-30965131** | **INMT** | **1** |
| **3581** | **chr 7: 31553703-31698334** | **CCDC129** | **1** |
| **3582** | **chr 7: 31790792-32338941** | **PDE1C** | **2** |
| **3583** | **chr 7: 32535037-33078516** | **LSM5** | **1** |
| **3583** | **chr 7: 32535037-33078516** | **AVL9** | **1** |
| **3584** | **chr 7: 33168855-33645680** | **BBS9** | **1** |
| **3585** | **chr 7: 33832779-33842768** | **none** | **1** |
| **3586** | **chr 7: 33944522-34195484** | **BMPER** | **1** |
| **3587** | **chr 7: 34386123-34911194** | **NPSR1** | **1** |
| **3587** | **chr 7: 34386123-34911194** | **NPSR1-AS1** | **1** |
| **3588** | **chr 7: 35840541-35944917** | **39326** | **1** |
| **3589** | **chr 7: 36192757-36341152** | **EEPD1** | **2** |
| **3590** | **chr 7: 36552455-36764154** | **AOAH** | **1** |
| **3590** | **chr 7: 36552455-36764154** | **AOAH-IT1** | **1** |
| **3591** | **chr 7: 36893960-37488852** | **ELMO1** | **6** |
| **3592** | **chr 7: 37723398-37991543** | **NME8** | **1** |
| **3592** | **chr 7: 37723398-37991543** | **GPR141** | **1** |
| **3593** | **chr 7: 38762562-38971994** | **VPS41** | **1** |
| **3594** | **chr 7: 39017597-39532694** | **POU6F2** | **2** |
| **3594** | **chr 7: 39017597-39532694** | **POU6F2-AS1** | **2** |
| **3595** | **chr 7: 40174574-40900362** | **C7ORF10** | **2** |
| **3595** | **chr 7: 40174574-40900362** | **MPLKIP** | **2** |
| **3596** | **chr 7: 42948324-42971822** | **PSMA2** | **1** |
| **3596** | **chr 7: 42948324-42971822** | **C7ORF25** | **1** |
| **3597** | **chr 7: 43152197-43605600** | **MIR3943** | **1** |
| **3597** | **chr 7: 43152197-43605600** | **HECW1** | **1** |
| **3598** | **chr 7: 43278664-43288867** | **HECW1** | **1** |
| **3599** | **chr 7: 43548326-43562141** | **HECW1** | **1** |
| **3600** | **chr 7: 44418719-44530479** | **NUDCD3** | **1** |
| **3601** | **chr 7: 45613738-45762715** | **ADCY1** | **1** |
| **3602** | **chr 7: 46930222-47118726** | **none** | **2** |
| **3603** | **chr 7: 47314751-47622156** | **TNS3** | **2** |
| **3604** | **chr 7: 47735327-48019178** | **LINC00525** | **3** |
| **3604** | **chr 7: 47735327-48019178** | **PKD1L1** | **3** |
| **3605** | **chr 7: 48211054-48687092** | **ABCA13** | **1** |
| **3606** | **chr 7: 50343719-50472799** | **IKZF1** | **2** |
| **3607** | **chr 7: 50526133-50633154** | **DDC** | **1** |
| **3608** | **chr 7: 50599456-50611161** | **DDC** | **1** |
| **3609** | **chr 7: 50657759-50861159** | **GRB10** | **2** |
| **3610** | **chr 7: 50934443-51090687** | **COBL** | **1** |
| **3611** | **chr 7: 54610017-54639419** | **VSTM2A** | **1** |
| **3611** | **chr 7: 54610017-54639419** | **LOC285878** | **1** |
| **3612** | **chr 7: 55503748-55640681** | **VOPP1** | **1** |
| **3613** | **chr 7: 55954969-56067874** | **MRPS17** | **1** |
| **3613** | **chr 7: 55954969-56067874** | **ZNF713** | **1** |
| **3614** | **chr 7: 57659499-57669810** | **none** | **1** |
| **3615** | **chr 7: 62505204-62514809** | **none** | **1** |
| **3616** | **chr 7: 64330549-64391344** | **ZNF273** | **1** |
| **3617** | **chr 7: 65338253-65424550** | **VKORC1L1** | **1** |
| **3618** | **chr 7: 67615269-67753220** | **none** | **2** |
| **3619** | **chr 7: 68689682-68895909** | **none** | **2** |
| **3620** | **chr 7: 69063904-70258054** | **AUTS2** | **9** |
| **3621** | **chr 7: 70597154-71178585** | **MIR3914-1** | **4** |
| **3621** | **chr 7: 70597154-71178585** | **WBSCR17** | **4** |
| **3622** | **chr 7: 71244475-71912136** | **CALN1** | **4** |
| **3623** | **chr 7: 72742166-72772634** | **FKBP6** | **1** |
| **3623** | **chr 7: 72742166-72772634** | **TRIM50** | **1** |
| **3624** | **chr 7: 73007523-73038873** | **MLXIPL** | **2** |
| **3625** | **chr 7: 73213871-73247014** | **CLDN4** | **3** |
| **3626** | **chr 7: 73703804-73820273** | **CLIP2** | **3** |
| **3627** | **chr 7: 73868119-74016931** | **GTF2IRD1** | **1** |
| **3628** | **chr 7: 74071993-74175026** | **GTF2I** | **1** |
| **3629** | **chr 7: 74103272-74306731** | **NCF1** | **1** |
| **3629** | **chr 7: 74103272-74306731** | **GTF2I** | **1** |
| **3630** | **chr 7: 75162620-75368280** | **HIP1** | **2** |
| **3631** | **chr 7: 75831215-75916605** | **SRRM3** | **2** |
| **3632** | **chr 7: 76139744-76958850** | **LOC100133091** | **1** |
| **3632** | **chr 7: 76139744-76958850** | **UPK3B** | **1** |
| **3633** | **chr 7: 77428121-77586818** | **PHTF2** | **1** |
| **3633** | **chr 7: 77428121-77586818** | **TMEM60** | **1** |
| **3634** | **chr 7: 77646392-79082890** | **RPL13AP17** | **3** |
| **3634** | **chr 7: 77646392-79082890** | **MAGI2** | **3** |
| **3635** | **chr 7: 79082197-79100524** | **MAGI2** | **1** |
| **3636** | **chr 7: 79763270-79848718** | **GNAI1** | **1** |
| **3637** | **chr 7: 79998890-80308593** | **GNAT3** | **1** |
| **3637** | **chr 7: 79998890-80308593** | **CD36** | **1** |
| **3638** | **chr 7: 80087986-80141336** | **GNAT3** | **1** |
| **3639** | **chr 7: 81575759-82073114** | **CACNA2D1** | **3** |
| **3640** | **chr 7: 82383328-82792246** | **PCLO** | **4** |
| **3641** | **chr 7: 83585092-84122040** | **SEMA3A** | **2** |
| **3642** | **chr 7: 84624868-84816171** | **SEMA3D** | **1** |
| **3643** | **chr 7: 89964536-90839905** | **CLDN12** | **5** |
| **3643** | **chr 7: 89964536-90839905** | **GTPBP10** | **5** |
| **3644** | **chr 7: 91321322-91510034** | **MTERF** | **1** |
| **3645** | **chr 7: 93592073-93633694** | **BET1** | **1** |
| **3646** | **chr 7: 93598753-93640425** | **BET1** | **1** |
| **3647** | **chr 7: 94536513-94925727** | **PPP1R9A** | **2** |
| **3648** | **chr 7: 94784024-94843644** | **PPP1R9A** | **1** |
| **3649** | **chr 7: 95749531-95951459** | **SLC25A13** | **1** |
| **3649** | **chr 7: 95749531-95951459** | **MIR591** | **1** |
| **3650** | **chr 7: 96584452-96643377** | **DLX6** | **1** |
| **3650** | **chr 7: 96584452-96643377** | **DLX6-AS1** | **1** |
| **3651** | **chr 7: 98444110-98468394** | **TMEM130** | **1** |
| **3652** | **chr 7: 98625060-98741723** | **SMURF1** | **2** |
| **3653** | **chr 7: 98923520-98992424** | **ARPC1B** | **2** |
| **3653** | **chr 7: 98923520-98992424** | **ARPC1A** | **2** |
| **3654** | **chr 7: 99156028-99208439** | **ZNF655** | **1** |
| **3655** | **chr 7: 99933701-99997719** | **PMS2P1** | **1** |
| **3655** | **chr 7: 99933701-99997719** | **PILRB** | **1** |
| **3656** | **chr 7: 99998448-100026615** | **ZCWPW1** | **4** |
| **3656** | **chr 7: 99998448-100026615** | **MEPCE** | **4** |
| **3657** | **chr 7: 100081549-100092422** | **NYAP1** | **1** |
| **3658** | **chr 7: 100199799-100205798** | **PCOLCE-AS1** | **1** |
| **3659** | **chr 7: 100331248-100395419** | **ZAN** | **2** |
| **3660** | **chr 7: 100400186-100425121** | **EPHB4** | **1** |
| **3661** | **chr 7: 100424441-100464631** | **SLC12A9** | **1** |
| **3661** | **chr 7: 100424441-100464631** | **EPHB4** | **1** |
| **3662** | **chr 7: 100434935-100450238** | **SLC12A9** | **1** |
| **3663** | **chr 7: 100612903-100662230** | **MUC12** | **2** |
| **3664** | **chr 7: 100813773-100823557** | **NAT16** | **1** |
| **3665** | **chr 7: 101006100-101202304** | **COL26A1** | **1** |
| **3666** | **chr 7: 101458958-101927249** | **CUX1** | **3** |
| **3667** | **chr 7: 101928404-101962178** | **MIR4285** | **2** |
| **3667** | **chr 7: 101928404-101962178** | **SH2B2** | **2** |
| **3668** | **chr 7: 102073552-102097268** | **ORAI2** | **1** |
| **3668** | **chr 7: 102073552-102097268** | **ALKBH4** | **1** |
| **3669** | **chr 7: 102453307-102715286** | **FBXL13** | **1** |
| **3670** | **chr 7: 102993176-103086624** | **PSMC2** | **1** |
| **3671** | **chr 7: 103969103-104549001** | **LHFPL3-AS1** | **2** |
| **3671** | **chr 7: 103969103-104549001** | **LHFPL3** | **2** |
| **3672** | **chr 7: 104751150-105039755** | **MLL5** | **2** |
| **3672** | **chr 7: 104751150-105039755** | **SRPK2** | **2** |
| **3673** | **chr 7: 106012696-106410653** | **CCDC71L** | **2** |
| **3674** | **chr 7: 106841999-107204959** | **HBP1** | **1** |
| **3675** | **chr 7: 107220421-107269943** | **BCAP29** | **1** |
| **3676** | **chr 7: 110072295-110174811** | **none** | **1** |
| **3677** | **chr 7: 110303109-111202573** | **IMMP2L** | **5** |
| **3678** | **chr 7: 111366165-111846466** | **ZNF277** | **1** |
| **3678** | **chr 7: 111366165-111846466** | **DOCK4** | **1** |
| **3679** | **chr 7: 111846642-111983151** | **ZNF277** | **1** |
| **3679** | **chr 7: 111846642-111983151** | **DOCK4** | **1** |
| **3680** | **chr 7: 111968243-112049678** | **ZNF277** | **1** |
| **3681** | **chr 7: 113726381-114333827** | **FOXP2** | **1** |
| **3682** | **chr 7: 114562208-114659256** | **MDFIC** | **1** |
| **3683** | **chr 7: 114701590-114871409** | **MDFIC** | **2** |
| **3684** | **chr 7: 115877982-116139519** | **TES** | **1** |
| **3685** | **chr 7: 115927433-116148595** | **CAV2** | **1** |
| **3686** | **chr 7: 116593291-116870157** | **ST7** | **2** |
| **3686** | **chr 7: 116593291-116870157** | **ST7-AS1** | **2** |
| **3687** | **chr 7: 116916684-116963343** | **WNT2** | **1** |
| **3688** | **chr 7: 119913721-120390385** | **KCND2** | **3** |
| **3689** | **chr 7: 120427375-120498456** | **TSPAN12** | **1** |
| **3690** | **chr 7: 123565285-123611468** | **SPAM1** | **2** |
| **3691** | **chr 7: 123672258-124035172** | **TMEM229A** | **1** |
| **3692** | **chr 7: 125071400-125106455** | **none** | **1** |
| **3693** | **chr 7: 126019023-126064239** | **GRM8** | **1** |
| **3694** | **chr 7: 126078651-126893348** | **GRM8** | **3** |
| **3695** | **chr 7: 127228398-127242198** | **FSCN3** | **1** |
| **3695** | **chr 7: 127228398-127242198** | **ARF5** | **1** |
| **3696** | **chr 7: 128430810-128462186** | **CCDC136** | **1** |
| **3697** | **chr 7: 128470430-128499328** | **FLNC** | **1** |
| **3698** | **chr 7: 128490215-128550773** | **FLNC** | **1** |
| **3698** | **chr 7: 128490215-128550773** | **ATP6V1F** | **1** |
| **3699** | **chr 7: 128864863-129070052** | **AHCYL2** | **1** |
| **3700** | **chr 7: 129710349-129775560** | **KLHDC10** | **4** |
| **3701** | **chr 7: 129984629-130008571** | **CPA5** | **1** |
| **3702** | **chr 7: 130626518-130794935** | **FLJ43663** | **1** |
| **3703** | **chr 7: 130794854-131181395** | **MKLN1** | **1** |
| **3704** | **chr 7: 131808090-132333447** | **PLXNA4** | **5** |
| **3705** | **chr 7: 132469628-132766848** | **CHCHD3** | **1** |
| **3706** | **chr 7: 132937828-133751342** | **EXOC4** | **2** |
| **3707** | **chr 7: 134233887-134264627** | **AKR1B15** | **1** |
| **3708** | **chr 7: 134331559-134364565** | **BPGM** | **1** |
| **3709** | **chr 7: 134429002-134655479** | **CALD1** | **1** |
| **3710** | **chr 7: 134916730-134943244** | **STRA8** | **2** |
| **3711** | **chr 7: 135046546-135194875** | **CNOT4** | **2** |
| **3712** | **chr 7: 135242666-135333505** | **NUP205** | **1** |
| **3713** | **chr 7: 135347243-135378166** | **C7ORF73** | **1** |
| **3713** | **chr 7: 135347243-135378166** | **SLC13A4** | **1** |
| **3714** | **chr 7: 135365984-135433594** | **FAM180A** | **1** |
| **3714** | **chr 7: 135365984-135433594** | **SLC13A4** | **1** |
| **3715** | **chr 7: 137065782-137531838** | **DGKI** | **1** |
| **3716** | **chr 7: 137559724-137686813** | **CREB3L2** | **3** |
| **3717** | **chr 7: 138279029-138386097** | **SVOPL** | **2** |
| **3718** | **chr 7: 139024202-139108198** | **C7ORF55** | **1** |
| **3718** | **chr 7: 139024202-139108198** | **LUC7L2** | **1** |
| **3719** | **chr 7: 139476849-139720125** | **HIPK2** | **1** |
| **3719** | **chr 7: 139476849-139720125** | **TBXAS1** | **1** |
| **3720** | **chr 7: 140218219-140373793** | **DENND2A** | **3** |
| **3720** | **chr 7: 140218219-140373793** | **ADCK2** | **3** |
| **3721** | **chr 7: 140372952-140422590** | **ADCK2** | **1** |
| **3721** | **chr 7: 140372952-140422590** | **NDUFB2-AS1** | **1** |
| **3722** | **chr 7: 140774031-141180180** | **TMEM178B** | **1** |
| **3723** | **chr 7: 141250988-141355044** | **AGK** | **1** |
| **3724** | **chr 7: 143104905-143220542** | **EPHA1-AS1** | **3** |
| **3724** | **chr 7: 143104905-143220542** | **EPHA1** | **3** |
| **3725** | **chr 7: 143318042-143515397** | **FAM115C** | **2** |
| **3726** | **chr 7: 143339347-143534026** | **FAM115C** | **2** |
| **3727** | **chr 7: 144149033-144533488** | **TPK1** | **2** |
| **3728** | **chr 7: 145813452-148118090** | **CNTNAP2** | **13** |
| **3728** | **chr 7: 145813452-148118090** | **MIR548AQ** | **13** |
| **3729** | **chr 7: 150413644-150487837** | **GIMAP1** | **1** |
| **3729** | **chr 7: 150413644-150487837** | **GIMAP5** | **1** |
| **3730** | **chr 7: 150750898-150755617** | **CDK5** | **1** |
| **3731** | **chr 7: 150754296-150773614** | **CDK5** | **2** |
| **3732** | **chr 7: 150771828-150773617** | **SLC4A2** | **1** |
| **3733** | **chr 7: 150845675-150871832** | **GBX1** | **2** |
| **3734** | **chr 7: 151253196-151574210** | **PRKAG2** | **2** |
| **3735** | **chr 7: 151832009-152133090** | **MLL3** | **2** |
| **3735** | **chr 7: 151832009-152133090** | **FABP5P3** | **2** |
| **3736** | **chr 7: 153584181-154685995** | **DPP6** | **7** |
| **3737** | **chr 7: 157331749-158380480** | **PTPRN2** | **3** |
| **3737** | **chr 7: 157331749-158380480** | **MIR153-2** | **3** |
| **3738** | **chr 7: 158424002-158497520** | **NCAPG2** | **1** |
| **3739** | **chr 7: 158820865-158937649** | **VIPR2** | **1** |
| **3740** | **chr 8: 255305-281026** | **none** | **1** |
| **3741** | **chr 8: 2792874-4852494** | **CSMD1** | **10** |
| **3742** | **chr 8: 5901874-6115058** | **none** | **5** |
| **3743** | **chr 8: 8818709-8836535** | **ERI1** | **1** |
| **3744** | **chr 8: 9046520-9060364** | **PPP1R3B** | **1** |
| **3745** | **chr 8: 9106926-9271224** | **LOC157273** | **1** |
| **3746** | **chr 8: 9413423-9639856** | **TNKS** | **2** |
| **3746** | **chr 8: 9413423-9639856** | **MIR597** | **2** |
| **3747** | **chr 8: 9911777-10286401** | **MSRA** | **3** |
| **3748** | **chr 8: 10383055-10411676** | **PRSS55** | **1** |
| **3749** | **chr 8: 10753554-11058875** | **XKR6** | **1** |
| **3749** | **chr 8: 10753554-11058875** | **MIR598** | **1** |
| **3750** | **chr 8: 11197145-11296167** | **C8ORF12** | **1** |
| **3750** | **chr 8: 11197145-11296167** | **TDH** | **1** |
| **3751** | **chr 8: 11278971-11332224** | **C8ORF12** | **1** |
| **3751** | **chr 8: 11278971-11332224** | **FAM167A** | **1** |
| **3752** | **chr 8: 11534467-11617511** | **GATA4** | **3** |
| **3753** | **chr 8: 11921897-11929256** | **LOC100133267** | **1** |
| **3753** | **chr 8: 11921897-11929256** | **DEFB130** | **1** |
| **3754** | **chr 8: 12168470-12175825** | **LOC100133267** | **1** |
| **3754** | **chr 8: 12168470-12175825** | **DEFB130** | **1** |
| **3755** | **chr 8: 12940869-13373167** | **DLC1** | **4** |
| **3756** | **chr 8: 13947372-15095848** | **SGCZ** | **4** |
| **3756** | **chr 8: 13947372-15095848** | **MIR383** | **4** |
| **3757** | **chr 8: 15274723-15624158** | **TUSC3** | **1** |
| **3758** | **chr 8: 15965386-16424999** | **MSR1** | **2** |
| **3759** | **chr 8: 17658853-17718578** | **MTUS1** | **1** |
| **3760** | **chr 8: 18384810-18942240** | **PSD3** | **1** |
| **3761** | **chr 8: 18578414-18591915** | **PSD3** | **1** |
| **3762** | **chr 8: 18942501-19116979** | **LOC100128993** | **2** |
| **3763** | **chr 8: 18949242-18972534** | **none** | **1** |
| **3764** | **chr 8: 19261671-19615540** | **CSGALNACT1** | **4** |
| **3765** | **chr 8: 19759227-19824769** | **LPL** | **1** |
| **3766** | **chr 8: 20103675-20161474** | **LZTS1-AS1** | **1** |
| **3766** | **chr 8: 20103675-20161474** | **LZTS1** | **1** |
| **3767** | **chr 8: 20513201-20557645** | **none** | **1** |
| **3768** | **chr 8: 21547914-21669869** | **GFRA2** | **1** |
| **3769** | **chr 8: 21777179-21864096** | **XPO7** | **1** |
| **3770** | **chr 8: 22298331-22398652** | **PPP3CC** | **2** |
| **3771** | **chr 8: 22570768-22876848** | **RHOBTB2** | **2** |
| **3771** | **chr 8: 22570768-22876848** | **PEBP4** | **2** |
| **3772** | **chr 8: 22925741-22941132** | **TNFRSF10B** | **2** |
| **3772** | **chr 8: 22925741-22941132** | **LOC286059** | **2** |
| **3773** | **chr 8: 23127632-23153792** | **R3HCC1** | **1** |
| **3774** | **chr 8: 23564653-23647000** | **NKX2-6** | **2** |
| **3775** | **chr 8: 24151552-24216531** | **ADAM28** | **1** |
| **3776** | **chr 8: 24153326-24769586** | **ADAM28** | **1** |
| **3777** | **chr 8: 25699245-25902913** | **EBF2** | **1** |
| **3778** | **chr 8: 26240413-26363152** | **PNMA2** | **3** |
| **3778** | **chr 8: 26240413-26363152** | **BNIP3L** | **3** |
| **3779** | **chr 8: 26605666-26724790** | **ADRA1A** | **1** |
| **3780** | **chr 8: 27168998-27316903** | **PTK2B** | **3** |
| **3780** | **chr 8: 27168998-27316903** | **TRIM35** | **3** |
| **3781** | **chr 8: 27491384-27534293** | **SCARA3** | **3** |
| **3782** | **chr 8: 27727398-27850244** | **MIR4287** | **4** |
| **3782** | **chr 8: 27727398-27850244** | **SCARA5** | **4** |
| **3783** | **chr 8: 28107579-28196772** | **PNOC** | **3** |
| **3784** | **chr 8: 28203101-28347835** | **FBXO16** | **1** |
| **3784** | **chr 8: 28203101-28347835** | **ZNF395** | **1** |
| **3785** | **chr 8: 28351728-28431775** | **MIR4288** | **1** |
| **3785** | **chr 8: 28351728-28431775** | **FZD3** | **1** |
| **3786** | **chr 8: 29405687-29421858** | **none** | **1** |
| **3787** | **chr 8: 29952913-30041156** | **MIR548O2** | **1** |
| **3788** | **chr 8: 30631972-30671830** | **PPP2CB** | **1** |
| **3789** | **chr 8: 30891316-31031285** | **PURG** | **1** |
| **3790** | **chr 8: 31024593-31034683** | **WRN** | **2** |
| **3791** | **chr 8: 33228341-33371119** | **FUT10** | **2** |
| **3791** | **chr 8: 33228341-33371119** | **MAK16** | **2** |
| **3792** | **chr 8: 33462373-33896526** | **DUSP26** | **2** |
| **3793** | **chr 8: 33831218-33867113** | **none** | **1** |
| **3794** | **chr 8: 35092974-35654068** | **UNC5D** | **3** |
| **3795** | **chr 8: 36394908-36636682** | **KCNU1** | **2** |
| **3796** | **chr 8: 37278858-37411701** | **none** | **2** |
| **3797** | **chr 8: 37594116-37616619** | **ERLIN2** | **1** |
| **3798** | **chr 8: 37641708-37702414** | **BRF2** | **1** |
| **3798** | **chr 8: 37641708-37702414** | **GPR124** | **1** |
| **3799** | **chr 8: 38082735-38133076** | **DDHD2** | **1** |
| **3800** | **chr 8: 38120647-38126761** | **PPAPDC1B** | **1** |
| **3801** | **chr 8: 38585703-38710546** | **TACC1** | **1** |
| **3802** | **chr 8: 39442007-39587583** | **ADAM18** | **3** |
| **3803** | **chr 8: 40388108-40755352** | **ZMAT4** | **1** |
| **3804** | **chr 8: 41391673-41435887** | **AGPAT6** | **1** |
| **3804** | **chr 8: 41391673-41435887** | **GINS4** | **1** |
| **3805** | **chr 8: 41510738-41754280** | **ANK1** | **1** |
| **3806** | **chr 8: 42032235-42065242** | **PLAT** | **1** |
| **3807** | **chr 8: 42273992-42397069** | **SLC20A2** | **1** |
| **3808** | **chr 8: 42607762-42651535** | **CHRNA6** | **1** |
| **3809** | **chr 8: 42752074-42940931** | **RNF170** | **4** |
| **3810** | **chr 8: 48920959-48977268** | **UBE2V2** | **1** |
| **3811** | **chr 8: 49464126-49611069** | **EFCAB1** | **1** |
| **3812** | **chr 8: 50409321-50424739** | **none** | **2** |
| **3813** | **chr 8: 50822348-51706678** | **SNTG1** | **1** |
| **3814** | **chr 8: 52232137-52722005** | **PXDNL** | **2** |
| **3815** | **chr 8: 53023398-53373519** | **ST18** | **4** |
| **3816** | **chr 8: 53063379-53067452** | **ST18** | **1** |
| **3817** | **chr 8: 54879111-54935089** | **TCEA1** | **1** |
| **3818** | **chr 8: 56014948-56454613** | **XKR4** | **1** |
| **3818** | **chr 8: 56014948-56454613** | **SBF1P1** | **1** |
| **3819** | **chr 8: 57073462-57123883** | **PLAG1** | **1** |
| **3819** | **chr 8: 57073462-57123883** | **CHCHD7** | **1** |
| **3820** | **chr 8: 58055247-58153100** | **LOC100507651** | **3** |
| **3821** | **chr 8: 59717976-60031767** | **TOX** | **2** |
| **3822** | **chr 8: 61591336-61779465** | **CHD7** | **1** |
| **3823** | **chr 8: 62697605-62856739** | **none** | **1** |
| **3824** | **chr 8: 63161149-63912211** | **UG0898H09** | **2** |
| **3824** | **chr 8: 63161149-63912211** | **NKAIN3** | **2** |
| **3825** | **chr 8: 65285884-65296344** | **LOC100130155** | **1** |
| **3826** | **chr 8: 65500319-65711318** | **CYP7B1** | **3** |
| **3827** | **chr 8: 67579830-67774257** | **C8ORF44** | **1** |
| **3827** | **chr 8: 67579830-67774257** | **VCPIP1** | **1** |
| **3828** | **chr 8: 68334359-68658620** | **CPA6** | **1** |
| **3829** | **chr 8: 68864352-69149265** | **PREX2** | **1** |
| **3830** | **chr 8: 69215702-69243726** | **C8ORF34** | **1** |
| **3830** | **chr 8: 69215702-69243726** | **LOC286189** | **1** |
| **3831** | **chr 8: 69242956-69731257** | **C8ORF34** | **5** |
| **3831** | **chr 8: 69242956-69731257** | **LOC286189** | **5** |
| **3832** | **chr 8: 69824037-70016425** | **LOC100505718** | **1** |
| **3833** | **chr 8: 70378858-70573150** | **SULF1** | **1** |
| **3834** | **chr 8: 70579281-70747299** | **SLCO5A1** | **1** |
| **3835** | **chr 8: 70963885-70983928** | **PRDM14** | **1** |
| **3836** | **chr 8: 72587534-72615021** | **none** | **1** |
| **3837** | **chr 8: 72740401-73030628** | **MSC** | **1** |
| **3837** | **chr 8: 72740401-73030628** | **LOC100132891** | **1** |
| **3838** | **chr 8: 72932151-72987852** | **LOC100132891** | **1** |
| **3839** | **chr 8: 73114985-73163800** | **LOC392232** | **2** |
| **3840** | **chr 8: 74332603-74791145** | **STAU2-AS1** | **2** |
| **3840** | **chr 8: 74332603-74791145** | **STAU2** | **2** |
| **3841** | **chr 8: 74903586-74941322** | **LY96** | **2** |
| **3842** | **chr 8: 74964574-75012088** | **LY96** | **3** |
| **3843** | **chr 8: 75233364-75401107** | **JPH1** | **4** |
| **3843** | **chr 8: 75233364-75401107** | **GDAP1** | **4** |
| **3844** | **chr 8: 76135638-76236976** | **none** | **1** |
| **3845** | **chr 8: 76320148-76479078** | **HNF4G** | **1** |
| **3846** | **chr 8: 77593453-77779521** | **ZFHX4** | **1** |
| **3846** | **chr 8: 77593453-77779521** | **ZFHX4-AS1** | **1** |
| **3847** | **chr 8: 78311307-78449526** | **none** | **2** |
| **3848** | **chr 8: 79749763-79752757** | **IL7** | **2** |
| **3849** | **chr 8: 80523048-80578410** | **STMN2** | **1** |
| **3850** | **chr 8: 80830951-81143467** | **TPD52** | **1** |
| **3850** | **chr 8: 80830951-81143467** | **MRPS28** | **1** |
| **3851** | **chr 8: 81880044-82024303** | **PAG1** | **1** |
| **3852** | **chr 8: 85095021-85834079** | **RALYL** | **1** |
| **3853** | **chr 8: 86132815-86196302** | **CA13** | **1** |
| **3853** | **chr 8: 86132815-86196302** | **C8ORF59** | **1** |
| **3854** | **chr 8: 86134674-86157946** | **CA13** | **1** |
| **3855** | **chr 8: 86239836-86291243** | **CA1** | **1** |
| **3856** | **chr 8: 86285664-86361269** | **CA3** | **1** |
| **3856** | **chr 8: 86285664-86361269** | **CA1** | **1** |
| **3857** | **chr 8: 86851933-86963312** | **REXO1L2P** | **1** |
| **3858** | **chr 8: 86999551-87166457** | **ATP6V0D2** | **2** |
| **3858** | **chr 8: 86999551-87166457** | **PSKH2** | **2** |
| **3859** | **chr 8: 87060601-87100850** | **PSKH2** | **1** |
| **3860** | **chr 8: 87497058-87573726** | **CPNE3** | **1** |
| **3860** | **chr 8: 87497058-87573726** | **RMDN1** | **1** |
| **3861** | **chr 8: 87878669-88627447** | **CNBD1** | **3** |
| **3862** | **chr 8: 88554213-88767946** | **none** | **2** |
| **3863** | **chr 8: 89339064-89749363** | **MMP16** | **2** |
| **3864** | **chr 8: 91803777-91971636** | **LOC100127983** | **1** |
| **3864** | **chr 8: 91803777-91971636** | **NECAB1** | **1** |
| **3865** | **chr 8: 92555151-92919847** | **RUNX1T1** | **3** |
| **3866** | **chr 8: 92967202-93115514** | **RUNX1T1** | **2** |
| **3867** | **chr 8: 94225530-94712661** | **LINC00535** | **2** |
| **3867** | **chr 8: 94225530-94712661** | **FAM92A1** | **2** |
| **3868** | **chr 8: 95139398-95229531** | **CDH17** | **1** |
| **3869** | **chr 8: 95384187-95487337** | **RAD54B** | **1** |
| **3870** | **chr 8: 95825538-95893974** | **CCNE2** | **1** |
| **3870** | **chr 8: 95825538-95893974** | **INTS8** | **1** |
| **3871** | **chr 8: 96216683-96822364** | **C8ORF69** | **3** |
| **3871** | **chr 8: 96216683-96822364** | **C8ORF37** | **3** |
| **3872** | **chr 8: 97154561-97173020** | **GDF6** | **1** |
| **3873** | **chr 8: 98285716-98290176** | **TSPYL5** | **1** |
| **3874** | **chr 8: 98881067-99048944** | **MATN2** | **1** |
| **3875** | **chr 8: 99202060-99306621** | **NIPAL2** | **1** |
| **3876** | **chr 8: 99413630-99955055** | **STK3** | **3** |
| **3876** | **chr 8: 99413630-99955055** | **KCNS2** | **3** |
| **3877** | **chr 8: 99956630-99964332** | **OSR2** | **1** |
| **3878** | **chr 8: 100025493-100889808** | **VPS13B** | **4** |
| **3879** | **chr 8: 100973163-101143496** | **RGS22** | **1** |
| **3880** | **chr 8: 101269287-101348446** | **RNF19A** | **1** |
| **3881** | **chr 8: 102190105-102218421** | **ZNF706** | **1** |
| **3882** | **chr 8: 102424100-102464680** | **NACAP1** | **1** |
| **3882** | **chr 8: 102424100-102464680** | **GRHL2** | **1** |
| **3883** | **chr 8: 102504659-102681954** | **GRHL2** | **1** |
| **3884** | **chr 8: 102698770-103137135** | **NCALD** | **5** |
| **3885** | **chr 8: 103265239-103425069** | **UBR5** | **1** |
| **3886** | **chr 8: 103876527-103990104** | **AZIN1** | **1** |
| **3887** | **chr 8: 104152937-104242533** | **C8ORF56** | **3** |
| **3887** | **chr 8: 104152937-104242533** | **BAALC** | **3** |
| **3888** | **chr 8: 104169217-104311000** | **BAALC** | **3** |
| **3889** | **chr 8: 105342551-105479281** | **DPYS** | **2** |
| **3889** | **chr 8: 105342551-105479281** | **DCSTAMP** | **2** |
| **3890** | **chr 8: 105602960-106816760** | **ZFPM2** | **3** |
| **3891** | **chr 8: 107282371-107764922** | **OXR1** | **1** |
| **3892** | **chr 8: 108261720-108510283** | **ANGPT1** | **2** |
| **3893** | **chr 8: 108911543-109095913** | **RSPO2** | **1** |
| **3894** | **chr 8: 110586206-110704020** | **SYBU** | **1** |
| **3895** | **chr 8: 112111189-112248432** | **none** | **1** |
| **3896** | **chr 8: 113235156-114449328** | **CSMD3** | **1** |
| **3897** | **chr 8: 116420723-116821899** | **TRPS1** | **1** |
| **3898** | **chr 8: 116962735-117337297** | **LINC00536** | **5** |
| **3899** | **chr 8: 118806728-119124092** | **EXT1** | **1** |
| **3900** | **chr 8: 119201697-119634234** | **SAMD12** | **6** |
| **3901** | **chr 8: 119294377-119412844** | **SAMD12** | **3** |
| **3902** | **chr 8: 120569325-120685693** | **ENPP2** | **1** |
| **3903** | **chr 8: 123682623-123706345** | **none** | **1** |
| **3904** | **chr 8: 123793632-123986750** | **ZHX2** | **1** |
| **3905** | **chr 8: 124510128-124553446** | **FBXO32** | **1** |
| **3906** | **chr 8: 124693033-124749647** | **ANXA13** | **1** |
| **3907** | **chr 8: 124864226-125132302** | **FER1L6** | **2** |
| **3907** | **chr 8: 124864226-125132302** | **FER1L6-AS1** | **2** |
| **3908** | **chr 8: 125058313-125183763** | **FER1L6** | **2** |
| **3909** | **chr 8: 125563030-125740730** | **MTSS1** | **2** |
| **3910** | **chr 8: 126103920-126379362** | **NSMCE2** | **1** |
| **3910** | **chr 8: 126103920-126379362** | **KIAA0196** | **1** |
| **3911** | **chr 8: 126479180-126553615** | **TRIB1** | **2** |
| **3912** | **chr 8: 127570119-127725660** | **FAM84B** | **1** |
| **3913** | **chr 8: 128220110-128231333** | **none** | **1** |
| **3914** | **chr 8: 128302061-128494384** | **POU5F1B** | **1** |
| **3914** | **chr 8: 128302061-128494384** | **LOC727677** | **1** |
| **3915** | **chr 8: 128806778-129113499** | **MIR1204** | **2** |
| **3915** | **chr 8: 128806778-129113499** | **PVT1** | **2** |
| **3916** | **chr 8: 129417514-129576925** | **none** | **1** |
| **3917** | **chr 8: 130228712-130253496** | **LOC728724** | **1** |
| **3918** | **chr 8: 130363936-130695925** | **none** | **1** |
| **3919** | **chr 8: 130851838-131029375** | **FAM49B** | **3** |
| **3920** | **chr 8: 131792546-132054672** | **ADCY8** | **1** |
| **3921** | **chr 8: 133036466-133123406** | **HHLA1** | **1** |
| **3921** | **chr 8: 133036466-133123406** | **OC90** | **1** |
| **3922** | **chr 8: 133133107-133493200** | **KCNQ3** | **4** |
| **3923** | **chr 8: 133879202-134147147** | **TG** | **1** |
| **3923** | **chr 8: 133879202-134147147** | **SLA** | **1** |
| **3924** | **chr 8: 134048972-134115298** | **TG** | **1** |
| **3924** | **chr 8: 134048972-134115298** | **SLA** | **1** |
| **3925** | **chr 8: 135224107-135332690** | **none** | **2** |
| **3926** | **chr 8: 135490030-135725292** | **ZFAT** | **2** |
| **3927** | **chr 8: 136469699-136668965** | **KHDRBS3** | **1** |
| **3928** | **chr 8: 137059263-137178365** | **none** | **2** |
| **3929** | **chr 8: 137543040-137997178** | **none** | **2** |
| **3930** | **chr 8: 138821686-139095813** | **FAM135B** | **1** |
| **3931** | **chr 8: 139142265-139509065** | **FAM135B** | **2** |
| **3932** | **chr 8: 139600477-139926249** | **COL22A1** | **1** |
| **3933** | **chr 8: 140742585-141468678** | **TRAPPC9** | **4** |
| **3934** | **chr 8: 141541263-141645718** | **AGO2** | **4** |
| **3935** | **chr 8: 141667998-142012315** | **PTK2** | **2** |
| **3936** | **chr 8: 142127376-142205907** | **DENND3** | **1** |
| **3937** | **chr 8: 142443928-142517330** | **MROH5** | **2** |
| **3938** | **chr 8: 143293440-143484601** | **TSNARE1** | **2** |
| **3939** | **chr 8: 143530790-143626370** | **BAI1** | **1** |
| **3940** | **chr 8: 143808620-143818345** | **LOC100288181** | **1** |
| **3940** | **chr 8: 143808620-143818345** | **THEM6** | **1** |
| **3941** | **chr 8: 143866789-143893536** | **LY6D** | **3** |
| **3942** | **chr 8: 144661866-144681711** | **EEF1D** | **1** |
| **3943** | **chr 8: 144840322-144853992** | **SCRIB** | **1** |
| **3943** | **chr 8: 144840322-144853992** | **LOC100128338** | **1** |
| **3944** | **chr 8: 144989320-145050902** | **PLEC** | **2** |
| **3945** | **chr 8: 145162628-145173218** | **KIAA1875** | **1** |
| **3946** | **chr 8: 145202918-145316843** | **MROH1** | **1** |
| **3947** | **chr 8: 145654164-145669827** | **VPS28** | **1** |
| **3947** | **chr 8: 145654164-145669827** | **TONSL** | **1** |
| **3948** | **chr 8: 146066426-146127553** | **ZNF7** | **1** |
| **3948** | **chr 8: 146066426-146127553** | **COMMD5** | **1** |
| **3949** | **chr 9: 14510-29739** | **WASH1** | **1** |
| **3949** | **chr 9: 14510-29739** | **DDX11L5** | **1** |
| **3950** | **chr 9: 2907072-3053408** | **none** | **1** |
| **3951** | **chr 9: 3824126-4348392** | **GLIS3** | **1** |
| **3952** | **chr 9: 4792868-4885917** | **RCL1** | **1** |
| **3952** | **chr 9: 4792868-4885917** | **MIR101-2** | **1** |
| **3953** | **chr 9: 5629024-5776557** | **KIAA1432** | **1** |
| **3954** | **chr 9: 5765075-5833117** | **KIAA1432** | **2** |
| **3955** | **chr 9: 6716547-7175648** | **KDM4C** | **1** |
| **3956** | **chr 9: 8314245-10612723** | **PTPRD** | **5** |
| **3957** | **chr 9: 14081841-14398982** | **NFIB** | **2** |
| **3958** | **chr 9: 14317083-14357907** | **NFIB** | **1** |
| **3959** | **chr 9: 14734663-14910993** | **FREM1** | **2** |
| **3960** | **chr 9: 14987299-15146399** | **LOC389705** | **2** |
| **3961** | **chr 9: 15552894-16061661** | **CCDC171** | **3** |
| **3962** | **chr 9: 16409500-16870841** | **BNC2** | **2** |
| **3963** | **chr 9: 17134979-17503921** | **CNTLN** | **2** |
| **3964** | **chr 9: 19408924-19452018** | **ACER2** | **1** |
| **3965** | **chr 9: 19507449-19786926** | **SLC24A2** | **1** |
| **3966** | **chr 9: 20341662-20622542** | **MIR4473** | **3** |
| **3966** | **chr 9: 20341662-20622542** | **MLLT3** | **3** |
| **3967** | **chr 9: 21802541-22121096** | **C9ORF53** | **1** |
| **3967** | **chr 9: 21802541-22121096** | **MTAP** | **1** |
| **3968** | **chr 9: 21967750-21995300** | **C9ORF53** | **1** |
| **3968** | **chr 9: 21967750-21995300** | **CDKN2A** | **1** |
| **3969** | **chr 9: 22646198-22824212** | **FLJ35282** | **3** |
| **3970** | **chr 9: 27245681-27282791** | **LINC00032** | **1** |
| **3971** | **chr 9: 27325206-27529779** | **MOB3B** | **3** |
| **3971** | **chr 9: 27325206-27529779** | **IFNK** | **3** |
| **3972** | **chr 9: 27948075-28670283** | **LINGO2** | **2** |
| **3973** | **chr 9: 33290508-33371155** | **NFX1** | **1** |
| **3974** | **chr 9: 33785947-33818793** | **PRSS3** | **1** |
| **3975** | **chr 9: 34398181-34458568** | **C9ORF24** | **1** |
| **3975** | **chr 9: 34398181-34458568** | **FAM219A** | **1** |
| **3976** | **chr 9: 34457411-34520982** | **DNAI1** | **2** |
| **3976** | **chr 9: 34457411-34520982** | **FAM219A** | **2** |
| **3977** | **chr 9: 34621375-34628104** | **DCTN3** | **1** |
| **3977** | **chr 9: 34621375-34628104** | **ARID3C** | **1** |
| **3978** | **chr 9: 34989637-34998897** | **DNAJB5** | **1** |
| **3979** | **chr 9: 35772159-35790429** | **NPR2** | **2** |
| **3979** | **chr 9: 35772159-35790429** | **MSMP** | **2** |
| **3980** | **chr 9: 35792150-35809729** | **NPR2** | **1** |
| **3980** | **chr 9: 35792150-35809729** | **SPAG8** | **1** |
| **3981** | **chr 9: 36833271-37034103** | **PAX5** | **3** |
| **3981** | **chr 9: 36833271-37034103** | **MIR4540** | **3** |
| **3982** | **chr 9: 37120535-37358146** | **ZCCHC7** | **3** |
| **3983** | **chr 9: 37510888-38069210** | **FBXO10** | **1** |
| **3983** | **chr 9: 37510888-38069210** | **TOMM5** | **1** |
| **3984** | **chr 9: 39072763-39288312** | **CNTNAP3** | **1** |
| **3985** | **chr 9: 39384502-39405302** | **LOC653501** | **1** |
| **3985** | **chr 9: 39384502-39405302** | **SPATA31A2** | **1** |
| **3986** | **chr 9: 39722429-39817308** | **none** | **1** |
| **3987** | **chr 9: 40491621-40633282** | **none** | **1** |
| **3988** | **chr 9: 41277732-41298538** | **SPATA31A5** | **1** |
| **3989** | **chr 9: 41529582-41550381** | **LOC653501** | **1** |
| **3989** | **chr 9: 41529582-41550381** | **SPATA31A5** | **1** |
| **3990** | **chr 9: 43684901-43924049** | **CNTNAP3B** | **1** |
| **3991** | **chr 9: 44823713-44835521** | **none** | **1** |
| **3992** | **chr 9: 45034956-45047145** | **FAM27C** | **1** |
| **3993** | **chr 9: 71320574-71624092** | **PIP5K1B** | **1** |
| **3993** | **chr 9: 71320574-71624092** | **FAM122A** | **1** |
| **3994** | **chr 9: 72658496-72841886** | **LOC100507244** | **1** |
| **3994** | **chr 9: 72658496-72841886** | **MAMDC2** | **1** |
| **3995** | **chr 9: 72700731-72790804** | **LOC100507244** | **1** |
| **3995** | **chr 9: 72700731-72790804** | **MAMDC2** | **1** |
| **3996** | **chr 9: 73143978-74061820** | **TRPM3** | **6** |
| **3997** | **chr 9: 74477367-74525847** | **FAM108B1** | **1** |
| **3998** | **chr 9: 75515577-75695358** | **ALDH1A1** | **1** |
| **3999** | **chr 9: 77337410-77503010** | **TRPM6** | **1** |
| **4000** | **chr 9: 78505559-78977255** | **PCSK5** | **1** |
| **4001** | **chr 9: 80331002-80646374** | **GNAQ** | **4** |
| **4002** | **chr 9: 82186687-82341658** | **TLE4** | **1** |
| **4003** | **chr 9: 84304627-84391815** | **TLE1** | **2** |
| **4004** | **chr 9: 84887670-85179347** | **none** | **1** |
| **4005** | **chr 9: 85594499-85678092** | **RASEF** | **1** |
| **4006** | **chr 9: 85857904-86153461** | **FRMD3** | **3** |
| **4007** | **chr 9: 86274877-86323118** | **UBQLN1** | **1** |
| **4008** | **chr 9: 86890371-86955672** | **SLC28A3** | **1** |
| **4009** | **chr 9: 87283465-87638505** | **NTRK2** | **2** |
| **4010** | **chr 9: 88161454-88356944** | **AGTPBP1** | **1** |
| **4011** | **chr 9: 88902647-88969369** | **ZCCHC6** | **1** |
| **4012** | **chr 9: 90112142-90323548** | **DAPK1** | **4** |
| **4013** | **chr 9: 93881343-93945044** | **AUH** | **2** |
| **4013** | **chr 9: 93881343-93945044** | **LOC100129316** | **2** |
| **4014** | **chr 9: 93881419-93925369** | **LOC100129316** | **2** |
| **4015** | **chr 9: 93976096-94124195** | **AUH** | **1** |
| **4016** | **chr 9: 94325372-94712444** | **MIR3910-1** | **1** |
| **4016** | **chr 9: 94325372-94712444** | **MIR3910-2** | **1** |
| **4017** | **chr 9: 94903579-94922396** | **LINC00475** | **2** |
| **4018** | **chr 9: 95087765-95382815** | **NOL8** | **1** |
| **4018** | **chr 9: 95087765-95382815** | **CENPP** | **1** |
| **4019** | **chr 9: 95256364-95298937** | **CENPP** | **1** |
| **4020** | **chr 9: 95375465-95432547** | **CENPP** | **1** |
| **4021** | **chr 9: 95473644-95527094** | **BICD2** | **1** |
| **4022** | **chr 9: 95709732-95798518** | **FGD3** | **1** |
| **4023** | **chr 9: 97488982-97849441** | **C9ORF3** | **1** |
| **4024** | **chr 9: 97861335-98079991** | **FANCC** | **2** |
| **4025** | **chr 9: 98412435-98478000** | **none** | **1** |
| **4026** | **chr 9: 98782013-98790247** | **LINC00092** | **1** |
| **4027** | **chr 9: 99082987-99145992** | **SLC35D2** | **1** |
| **4028** | **chr 9: 99252522-99382112** | **HABP4** | **1** |
| **4028** | **chr 9: 99252522-99382112** | **CDC14B** | **1** |
| **4029** | **chr 9: 100000730-100140806** | **LOC100499484** | **1** |
| **4029** | **chr 9: 100000730-100140806** | **LOC100499484** | **1** |
| **4030** | **chr 9: 100174231-100258407** | **TDRD7** | **4** |
| **4031** | **chr 9: 100961310-101017915** | **TBC1D2** | **1** |
| **4032** | **chr 9: 101050390-101471479** | **GABBR2** | **3** |
| **4033** | **chr 9: 101705460-101833069** | **COL15A1** | **1** |
| **4034** | **chr 9: 102117618-102139522** | **NAMA** | **1** |
| **4035** | **chr 9: 102648607-102668883** | **STX17** | **2** |
| **4035** | **chr 9: 102648607-102668883** | **LOC441461** | **2** |
| **4036** | **chr 9: 104230720-104243784** | **TMEM246** | **1** |
| **4037** | **chr 9: 104235452-104295819** | **TMEM246** | **1** |
| **4037** | **chr 9: 104235452-104295819** | **RNF20** | **1** |
| **4038** | **chr 9: 105281917-105419796** | **none** | **1** |
| **4039** | **chr 9: 110045417-110094475** | **RAD23B** | **2** |
| **4040** | **chr 9: 111934254-112083244** | **EPB41L4B** | **3** |
| **4041** | **chr 9: 112137745-112260590** | **PTPN3** | **1** |
| **4042** | **chr 9: 112403067-112934792** | **PALM2** | **2** |
| **4043** | **chr 9: 113127530-113342160** | **SVEP1** | **1** |
| **4044** | **chr 9: 113431050-113563859** | **MUSK** | **2** |
| **4045** | **chr 9: 113635542-113800981** | **LPAR1** | **1** |
| **4046** | **chr 9: 113901525-114047116** | **OR2K2** | **1** |
| **4047** | **chr 9: 114122971-114247025** | **KIAA0368** | **1** |
| **4048** | **chr 9: 114448452-114557288** | **C9ORF84** | **3** |
| **4049** | **chr 9: 114659045-114697649** | **UGCG** | **1** |
| **4049** | **chr 9: 114659045-114697649** | **MIR4668** | **1** |
| **4050** | **chr 9: 115513117-115643951** | **SNX30** | **1** |
| **4050** | **chr 9: 115513117-115643951** | **SLC46A2** | **1** |
| **4051** | **chr 9: 116207010-116360018** | **RGS3** | **4** |
| **4052** | **chr 9: 116413235-116445135** | **none** | **4** |
| **4053** | **chr 9: 116917839-117074791** | **MIR455** | **1** |
| **4053** | **chr 9: 116917839-117074791** | **COL27A1** | **1** |
| **4054** | **chr 9: 117546914-117568406** | **TNFSF15** | **1** |
| **4055** | **chr 9: 117904096-118164923** | **DEC1** | **3** |
| **4056** | **chr 9: 118916082-119164601** | **PAPPA** | **2** |
| **4057** | **chr 9: 119048107-119080968** | **PAPPA** | **1** |
| **4058** | **chr 9: 119187503-120177348** | **ASTN2** | **6** |
| **4059** | **chr 9: 119266561-119324572** | **ASTN2** | **1** |
| **4060** | **chr 9: 120224571-120228538** | **none** | **1** |
| **4061** | **chr 9: 121915735-122131745** | **DBC1** | **3** |
| **4062** | **chr 9: 123617976-123639606** | **PHF19** | **1** |
| **4063** | **chr 9: 123714615-123812554** | **C5** | **1** |
| **4064** | **chr 9: 123970071-124095121** | **GSN** | **1** |
| **4065** | **chr 9: 124137518-124214455** | **STOM** | **1** |
| **4065** | **chr 9: 124137518-124214455** | **GGTA1P** | **1** |
| **4066** | **chr 9: 124207268-124262306** | **GGTA1P** | **1** |
| **4067** | **chr 9: 124584206-124855885** | **TTLL11** | **1** |
| **4068** | **chr 9: 125871772-126030855** | **MIR600** | **2** |
| **4068** | **chr 9: 125871772-126030855** | **MIR600HG** | **2** |
| **4069** | **chr 9: 126141932-126692431** | **DENND1A** | **6** |
| **4070** | **chr 9: 127019884-127115586** | **NEK6** | **2** |
| **4071** | **chr 9: 128003941-128019297** | **HSPA5** | **1** |
| **4072** | **chr 9: 128819858-128921892** | **none** | **2** |
| **4073** | **chr 9: 129351872-129375979** | **LMX1B** | **1** |
| **4074** | **chr 9: 129622943-129648157** | **ZBTB34** | **1** |
| **4075** | **chr 9: 129677052-129985445** | **RALGPS1** | **3** |
| **4076** | **chr 9: 129986543-130155939** | **GARNL3** | **1** |
| **4077** | **chr 9: 130159420-130170703** | **SLC2A8** | **1** |
| **4078** | **chr 9: 130500595-130541020** | **SH2D3C** | **1** |
| **4079** | **chr 9: 130801413-130819641** | **NAIF1** | **1** |
| **4080** | **chr 9: 130965657-131017527** | **CIZ1** | **2** |
| **4080** | **chr 9: 130965657-131017527** | **DNM1** | **2** |
| **4081** | **chr 9: 131018107-131038274** | **SWI5** | **1** |
| **4081** | **chr 9: 131018107-131038274** | **GOLGA2** | **1** |
| **4082** | **chr 9: 131314865-131395941** | **SPTAN1** | **1** |
| **4082** | **chr 9: 131314865-131395941** | **WDR34** | **1** |
| **4083** | **chr 9: 132083294-132087184** | **C9ORF106** | **1** |
| **4084** | **chr 9: 132192930-132213701** | **LOC100506190** | **2** |
| **4085** | **chr 9: 132245729-132275965** | **LOC100506190** | **2** |
| **4086** | **chr 9: 132427919-132484875** | **PRRX2** | **1** |
| **4087** | **chr 9: 132649465-132805473** | **FNBP1** | **1** |
| **4088** | **chr 9: 133320315-133376661** | **ASS1** | **1** |
| **4089** | **chr 9: 133454351-133513739** | **FUBP3** | **1** |
| **4089** | **chr 9: 133454351-133513739** | **LOC100272217** | **1** |
| **4090** | **chr 9: 133589332-133763062** | **ABL1** | **2** |
| **4091** | **chr 9: 134269479-134375584** | **PRRC2B** | **1** |
| **4091** | **chr 9: 134269479-134375584** | **SNORD62A** | **1** |
| **4092** | **chr 9: 134735493-134955295** | **MED27** | **4** |
| **4093** | **chr 9: 135037333-135119921** | **NTNG2** | **2** |
| **4094** | **chr 9: 135600964-135754164** | **AK8** | **2** |
| **4094** | **chr 9: 135600964-135754164** | **C9ORF9** | **2** |
| **4095** | **chr 9: 135753413-135765588** | **AK8** | **1** |
| **4095** | **chr 9: 135753413-135765588** | **C9ORF9** | **1** |
| **4096** | **chr 9: 135820931-135867083** | **GFI1B** | **2** |
| **4096** | **chr 9: 135820931-135867083** | **TSC1** | **2** |
| **4097** | **chr 9: 136379707-136393734** | **TMEM8C** | **1** |
| **4098** | **chr 9: 136528681-136605077** | **SARDH** | **2** |
| **4099** | **chr 9: 136627015-136857726** | **VAV2** | **2** |
| **4100** | **chr 9: 137208943-137332431** | **RXRA** | **1** |
| **4100** | **chr 9: 137208943-137332431** | **MIR4669** | **1** |
| **4101** | **chr 9: 138555167-138558268** | **LCN9** | **1** |
| **4102** | **chr 9: 138594030-138684992** | **KCNT1** | **1** |
| **4103** | **chr 9: 139221931-139254057** | **DKFZP434A062** | **1** |
| **4103** | **chr 9: 139221931-139254057** | **GPSM1** | **1** |
| **4104** | **chr 9: 140194082-140196703** | **NRARP** | **2** |
| **4105** | **chr 9: 140201347-140317714** | **NOXA1** | **1** |
| **4105** | **chr 9: 140201347-140317714** | **EXD3** | **1** |
| **4106** | **chr 9: 140513443-140764468** | **EHMT1** | **4** |
| **4106** | **chr 9: 140513443-140764468** | **C9ORF37** | **4** |
| **4107** | **chr 9: 140772240-141019076** | **CACNA1B** | **1** |
| **4108** | **chr X: 294697-347690** | **PPP2R3B** | **1** |
| **4109** | **chr X: 585078-620146** | **SHOX** | **1** |
| **4110** | **chr X: 1387692-1429274** | **CSF2RA** | **1** |
| **4111** | **chr X: 1455508-1501578** | **IL3RA** | **4** |
| **4112** | **chr X: 1733893-1761974** | **ASMT** | **2** |
| **4113** | **chr X: 2137556-2420846** | **ZBED1** | **4** |
| **4113** | **chr X: 2137556-2420846** | **DHRSX** | **4** |
| **4114** | **chr X: 2609219-2659350** | **CD99** | **4** |
| **4115** | **chr X: 2670090-2734539** | **XG** | **1** |
| **4115** | **chr X: 2670090-2734539** | **XGPY2** | **1** |
| **4116** | **chr X: 3226605-3264682** | **MXRA5** | **2** |
| **4117** | **chr X: 3522410-3631649** | **PRKX** | **2** |
| **4118** | **chr X: 5758677-6146904** | **NLGN4X** | **4** |
| **4119** | **chr X: 6966960-7066231** | **HDHD1** | **2** |
| **4120** | **chr X: 8496914-8700227** | **KAL1** | **4** |
| **4121** | **chr X: 9431334-9687780** | **TBL1X** | **1** |
| **4122** | **chr X: 9693385-9754337** | **SHROOM2** | **1** |
| **4122** | **chr X: 9693385-9754337** | **GPR143** | **1** |
| **4123** | **chr X: 11136238-11683821** | **HCCS** | **2** |
| **4123** | **chr X: 11136238-11683821** | **ARHGAP6** | **2** |
| **4124** | **chr X: 12156584-12742642** | **FRMPD4** | **2** |
| **4125** | **chr X: 13789149-13956757** | **GPM6B** | **1** |
| **4126** | **chr X: 14547419-14749934** | **GLRA2** | **1** |
| **4127** | **chr X: 15402920-15511687** | **FIGF** | **1** |
| **4127** | **chr X: 15402920-15511687** | **PIR-FIGF** | **1** |
| **4128** | **chr X: 15645440-15683154** | **TMEM27** | **1** |
| **4129** | **chr X: 15693054-15805747** | **CA5B** | **2** |
| **4129** | **chr X: 15693054-15805747** | **CA5BP1** | **2** |
| **4130** | **chr X: 15843928-15873054** | **AP1S2** | **1** |
| **4131** | **chr X: 16171063-16188992** | **GRPR** | **2** |
| **4132** | **chr X: 16185603-16189587** | **GRPR** | **1** |
| **4133** | **chr X: 17393542-17754114** | **MIR4768** | **4** |
| **4133** | **chr X: 17393542-17754114** | **NHS** | **4** |
| **4134** | **chr X: 18658029-18690229** | **CDKL5** | **1** |
| **4135** | **chr X: 19007426-19140755** | **GPR64** | **1** |
| **4136** | **chr X: 19378173-19533379** | **PDHA1** | **1** |
| **4136** | **chr X: 19378173-19533379** | **MAP3K15** | **1** |
| **4137** | **chr X: 19552092-19905719** | **SH3KBP1** | **2** |
| **4138** | **chr X: 19930977-19988416** | **CXORF23** | **1** |
| **4139** | **chr X: 20168028-20285523** | **RPS6KA3** | **1** |
| **4140** | **chr X: 21392535-21672813** | **CNKSR2** | **1** |
| **4141** | **chr X: 21724089-21776281** | **SMPX** | **1** |
| **4142** | **chr X: 21958690-22025798** | **SMS** | **1** |
| **4143** | **chr X: 22050558-22269427** | **PHEX-AS1** | **1** |
| **4143** | **chr X: 22050558-22269427** | **PHEX** | **1** |
| **4144** | **chr X: 22277913-23311263** | **ZNF645** | **1** |
| **4144** | **chr X: 22277913-23311263** | **LOC100873065** | **1** |
| **4145** | **chr X: 23720369-23784592** | **ACOT9** | **1** |
| **4146** | **chr X: 23851469-23926057** | **APOO** | **3** |
| **4146** | **chr X: 23851469-23926057** | **CXORF58** | **3** |
| **4147** | **chr X: 24483337-24557954** | **PDK3** | **1** |
| **4148** | **chr X: 24576203-24690794** | **PCYT1B** | **1** |
| **4149** | **chr X: 25021810-25034065** | **ARX** | **1** |
| **4150** | **chr X: 27608498-27766908** | **DCAF8L2** | **1** |
| **4151** | **chr X: 28605515-29974840** | **IL1RAPL1** | **1** |
| **4152** | **chr X: 30845558-30993201** | **TAB3** | **1** |
| **4153** | **chr X: 31115793-33357558** | **DMD** | **7** |
| **4154** | **chr X: 35937850-36008269** | **CXORF22** | **2** |
| **4155** | **chr X: 37208527-38548169** | **PRRG1** | **2** |
| **4156** | **chr X: 38008588-38080696** | **SRPX** | **1** |
| **4157** | **chr X: 38128415-38186817** | **RPGR** | **1** |
| **4158** | **chr X: 39085355-39159040** | **LOC286442** | **1** |
| **4159** | **chr X: 39909067-40036582** | **BCOR** | **2** |
| **4160** | **chr X: 40944887-41095832** | **USP9X** | **1** |
| **4161** | **chr X: 41134991-41136031** | **USP9X** | **1** |
| **4162** | **chr X: 41374186-41782716** | **GPR34** | **1** |
| **4162** | **chr X: 41374186-41782716** | **CASK** | **1** |
| **4163** | **chr X: 42111711-42558636** | **none** | **5** |
| **4164** | **chr X: 43625857-43741693** | **MAOB** | **2** |
| **4165** | **chr X: 44007127-44202918** | **EFHC2** | **1** |
| **4166** | **chr X: 44732756-44971847** | **KDM6A** | **1** |
| **4167** | **chr X: 45007618-45060146** | **CXORF36** | **1** |
| **4168** | **chr X: 45042495-45193162** | **CXORF36** | **2** |
| **4169** | **chr X: 46696374-46741793** | **RP2** | **2** |
| **4170** | **chr X: 47004267-47046212** | **RBM10** | **1** |
| **4170** | **chr X: 47004267-47046212** | **NDUFB11** | **1** |
| **4171** | **chr X: 47431302-47479252** | **ARAF** | **1** |
| **4171** | **chr X: 47431302-47479252** | **SYN1** | **1** |
| **4172** | **chr X: 47834249-47931025** | **ZNF182** | **1** |
| **4173** | **chr X: 47915698-47925971** | **ZNF630** | **1** |
| **4174** | **chr X: 48364910-48367226** | **PORCN** | **1** |
| **4175** | **chr X: 48644961-48652716** | **GATA1** | **1** |
| **4176** | **chr X: 48659783-48683392** | **HDAC6** | **1** |
| **4177** | **chr X: 48830133-48858675** | **GRIPAP1** | **1** |
| **4178** | **chr X: 49687224-49863892** | **CLCN5** | **1** |
| **4179** | **chr X: 50108407-50213737** | **DGKK** | **4** |
| **4180** | **chr X: 50334646-50557302** | **SHROOM4** | **1** |
| **4181** | **chr X: 51804922-51812368** | **MAGED4B** | **1** |
| **4181** | **chr X: 51804922-51812368** | **MAGED4** | **1** |
| **4182** | **chr X: 51927918-51935364** | **MAGED4B** | **1** |
| **4182** | **chr X: 51927918-51935364** | **MAGED4** | **1** |
| **4183** | **chr X: 52235227-52243954** | **XAGE1A** | **1** |
| **4183** | **chr X: 52235227-52243954** | **XAGE1B** | **1** |
| **4184** | **chr X: 53262057-53350522** | **IQSEC2** | **1** |
| **4185** | **chr X: 54834031-54842445** | **MAGED2** | **1** |
| **4186** | **chr X: 55026789-55035490** | **ALAS2** | **1** |
| **4186** | **chr X: 55026789-55035490** | **APEX2** | **1** |
| **4187** | **chr X: 56755691-56844813** | **LOC550643** | **1** |
| **4187** | **chr X: 56755691-56844813** | **UQCRBP1** | **1** |
| **4188** | **chr X: 62563105-62780951** | **SPIN4** | **2** |
| **4188** | **chr X: 62563105-62780951** | **LOC92249** | **2** |
| **4189** | **chr X: 63444075-63615333** | **ASB12** | **1** |
| **4189** | **chr X: 63444075-63615333** | **MTMR8** | **1** |
| **4190** | **chr X: 64808256-64961791** | **MSN** | **1** |
| **4191** | **chr X: 65815478-65859108** | **EDA2R** | **1** |
| **4192** | **chr X: 67262185-67653755** | **OPHN1** | **6** |
| **4193** | **chr X: 69509878-69640682** | **KIF4A** | **2** |
| **4193** | **chr X: 69509878-69640682** | **PDZD11** | **2** |
| **4194** | **chr X: 70145431-70150975** | **SLC7A3** | **1** |
| **4195** | **chr X: 70327253-70331958** | **CXORF65** | **1** |
| **4195** | **chr X: 70327253-70331958** | **IL2RG** | **1** |
| **4196** | **chr X: 70459473-70474996** | **BCYRN1** | **1** |
| **4197** | **chr X: 71130937-71363424** | **RPS26P11** | **6** |
| **4197** | **chr X: 71130937-71363424** | **NHSL2** | **6** |
| **4198** | **chr X: 71401202-71522776** | **PIN4** | **1** |
| **4199** | **chr X: 71475528-71497150** | **PIN4** | **1** |
| **4200** | **chr X: 79926352-80065187** | **BRWD3** | **1** |
| **4201** | **chr X: 83116153-83141705** | **CYLC1** | **1** |
| **4202** | **chr X: 85116184-85302566** | **CHM** | **1** |
| **4203** | **chr X: 85403461-86087607** | **DACH2** | **3** |
| **4204** | **chr X: 91034259-91878229** | **PCDH11X** | **2** |
| **4205** | **chr X: 95939661-96859996** | **DIAPH2** | **3** |
| **4206** | **chr X: 99546641-99665271** | **PCDH19** | **1** |
| **4207** | **chr X: 99929487-99987110** | **SYTL4** | **1** |
| **4208** | **chr X: 100264334-100349465** | **TMEM35** | **2** |
| **4208** | **chr X: 100264334-100349465** | **TRMT2B** | **2** |
| **4209** | **chr X: 101428071-101439994** | **BEX5** | **1** |
| **4210** | **chr X: 102024088-102161086** | **LINC00630** | **1** |
| **4211** | **chr X: 103028646-103047548** | **PLP1** | **1** |
| **4212** | **chr X: 103810995-105011822** | **TEX13A** | **5** |
| **4212** | **chr X: 103810995-105011822** | **IL1RAPL2** | **5** |
| **4213** | **chr X: 106366656-106449670** | **PIH1D3** | **1** |
| **4213** | **chr X: 106366656-106449670** | **NUP62CL** | **1** |
| **4214** | **chr X: 106765679-106848481** | **PRPS1** | **1** |
| **4215** | **chr X: 106956450-107020572** | **TSC22D3** | **1** |
| **4216** | **chr X: 107068984-107170423** | **MID2** | **1** |
| **4217** | **chr X: 107137826-107179210** | **MID2** | **1** |
| **4218** | **chr X: 107288199-107322414** | **VSIG1** | **1** |
| **4219** | **chr X: 107386779-107682727** | **COL4A6** | **1** |
| **4219** | **chr X: 107386779-107682727** | **ATG4A** | **1** |
| **4220** | **chr X: 107683073-107940775** | **COL4A6** | **3** |
| **4220** | **chr X: 107683073-107940775** | **COL4A5** | **3** |
| **4221** | **chr X: 109437413-109683461** | **AMMECR1** | **4** |
| **4222** | **chr X: 109602043-109699562** | **AMMECR1** | **3** |
| **4223** | **chr X: 109917083-110039286** | **CHRDL1** | **2** |
| **4224** | **chr X: 110187512-110470589** | **PAK3** | **1** |
| **4225** | **chr X: 111017542-111326004** | **TRPC5OS** | **1** |
| **4225** | **chr X: 111017542-111326004** | **TRPC5** | **1** |
| **4226** | **chr X: 111125124-111147218** | **TRPC5OS** | **1** |
| **4226** | **chr X: 111125124-111147218** | **TRPC5** | **1** |
| **4227** | **chr X: 112017730-112084043** | **AMOT** | **1** |
| **4228** | **chr X: 112859586-113181506** | **none** | **2** |
| **4229** | **chr X: 113818550-114144624** | **SNORA35** | **3** |
| **4229** | **chr X: 113818550-114144624** | **HTR2C** | **3** |
| **4230** | **chr X: 117031775-117251303** | **KLHL13** | **1** |
| **4231** | **chr X: 117629860-117820126** | **DOCK11** | **1** |
| **4232** | **chr X: 117973518-118015978** | **ZCCHC12** | **2** |
| **4233** | **chr X: 118108580-118152318** | **LONRF3** | **1** |
| **4234** | **chr X: 118212597-118284542** | **KIAA1210** | **1** |
| **4235** | **chr X: 118370215-118378429** | **PGRMC1** | **1** |
| **4236** | **chr X: 118383308-118391633** | **PGRMC1** | **1** |
| **4237** | **chr X: 122318005-122624766** | **GRIA3** | **1** |
| **4238** | **chr X: 122734411-122866906** | **THOC2** | **1** |
| **4239** | **chr X: 123094061-123556514** | **STAG2** | **5** |
| **4240** | **chr X: 123509752-124097666** | **TENM1** | **3** |
| **4241** | **chr X: 127457597-127734446** | **none** | **1** |
| **4242** | **chr X: 128872949-128903514** | **XPNPEP2** | **1** |
| **4243** | **chr X: 129115082-129192058** | **BCORL1** | **1** |
| **4244** | **chr X: 129611042-129658231** | **FAM45B** | **2** |
| **4245** | **chr X: 130115563-130192120** | **ARHGAP36** | **1** |
| **4246** | **chr X: 131760043-132095423** | **HS6ST2-AS1** | **3** |
| **4246** | **chr X: 131760043-132095423** | **HS6ST2** | **3** |
| **4247** | **chr X: 132669772-133119922** | **GPC3** | **1** |
| **4248** | **chr X: 133930818-133988640** | **FAM122B** | **1** |
| **4249** | **chr X: 134555867-134561999** | **LINC00086** | **1** |
| **4250** | **chr X: 134654583-134716435** | **DDX26B** | **1** |
| **4251** | **chr X: 135067597-135129423** | **SLC9A6** | **1** |
| **4252** | **chr X: 135229558-135293518** | **FHL1** | **1** |
| **4253** | **chr X: 135383121-135519215** | **GPR112** | **2** |
| **4254** | **chr X: 135991553-136103789** | **GPR101** | **1** |
| **4254** | **chr X: 135991553-136103789** | **SNORD61** | **1** |
| **4255** | **chr X: 137713734-138304939** | **FGF13** | **2** |
| **4256** | **chr X: 140590842-140738057** | **SPANXA2-OT1** | **2** |
| **4256** | **chr X: 140590842-140738057** | **SPANXA2** | **2** |
| **4257** | **chr X: 142372753-142604631** | **SPANXN3** | **2** |
| **4258** | **chr X: 147582138-148082193** | **AFF2** | **3** |
| **4259** | **chr X: 148558520-148622504** | **IDS** | **5** |
| **4260** | **chr X: 148621899-148632055** | **LOC100131434** | **1** |
| **4260** | **chr X: 148621899-148632055** | **CXORF40A** | **1** |
| **4261** | **chr X: 149106845-149392815** | **CXORF40B** | **5** |
| **4261** | **chr X: 149106845-149392815** | **LOC100272228** | **5** |
| **4262** | **chr X: 149529688-149682448** | **MAMLD1** | **3** |
| **4263** | **chr X: 149737068-149841795** | **MTM1** | **1** |
| **4264** | **chr X: 149934809-150067289** | **CD99L2** | **3** |
| **4265** | **chr X: 150884506-150891666** | **FATE1** | **1** |
| **4266** | **chr X: 151307354-151355329** | **MAGEA10** | **1** |
| **4266** | **chr X: 151307354-151355329** | **MAGEA5** | **1** |
| **4267** | **chr X: 151334705-151619830** | **MIR105-1** | **2** |
| **4267** | **chr X: 151334705-151619830** | **GABRA3** | **2** |
| **4268** | **chr X: 152157367-152162671** | **PNMA5** | **1** |
| **4269** | **chr X: 152599612-152625568** | **ZNF275** | **1** |
| **4270** | **chr X: 152710177-152760978** | **TREX2** | **3** |
| **4270** | **chr X: 152710177-152760978** | **HAUS7** | **3** |
| **4271** | **chr X: 152760396-152775012** | **HAUS7** | **1** |
| **4271** | **chr X: 152760396-152775012** | **BGN** | **1** |
| **4272** | **chr X: 153051220-153059978** | **SSR4** | **1** |
| **4272** | **chr X: 153051220-153059978** | **IDH3G** | **1** |
| **4273** | **chr X: 153213003-153237258** | **TMEM187** | **2** |
| **4273** | **chr X: 153213003-153237258** | **HCFC1** | **2** |
| **4274** | **chr X: 153287023-153363212** | **MECP2** | **1** |
| **4275** | **chr X: 153524023-153558700** | **TEX28** | **1** |
| **4276** | **chr X: 154695630-154841277** | **TMLHE-AS1** | **1** |
| **4276** | **chr X: 154695630-154841277** | **TMLHE** | **1** |
| **4277** | **chr X: 154719775-154899605** | **TMLHE-AS1** | **1** |
| **4277** | **chr X: 154719775-154899605** | **TMLHE** | **1** |
| **4278** | **chr Y: 244697-297690** | **PPP2R3B** | **1** |
| **4279** | **chr Y: 535078-570146** | **SHOX** | **1** |
| **4280** | **chr Y: 1337692-1379274** | **CSF2RA** | **1** |
| **4281** | **chr Y: 1405508-1451578** | **IL3RA** | **4** |
| **4282** | **chr Y: 1683893-1711974** | **ASMT** | **2** |
| **4283** | **chr Y: 2087556-2370846** | **ZBED1** | **4** |
| **4283** | **chr Y: 2087556-2370846** | **DHRSX** | **4** |
| **4284** | **chr Y: 2559219-2609350** | **CD99** | **4** |
| **4285** | **chr Y: 2709526-2800041** | **RPS4Y1** | **1** |
| **4286** | **chr Y: 2803111-2850547** | **ZFY** | **1** |
| **4287** | **chr Y: 2870952-2970313** | **LINC00278** | **2** |
| **4288** | **chr Y: 4868266-5610265** | **PCDH11Y** | **5** |
| **4289** | **chr Y: 6778726-6959724** | **TBL1Y** | **1** |
| **4290** | **chr Y: 8651350-8685423** | **TTTY11** | **1** |
| **4291** | **chr Y: 9461791-9463961** | **RBMY3AP** | **1** |
| **4292** | **chr Y: 15042074-15060090** | **DDX3Y** | **1** |
| **4293** | **chr Y: 15360258-15592553** | **UTY** | **2** |
| **4294** | **chr Y: 16634517-16957530** | **NLGN4Y** | **1** |
| **4295** | **chr Y: 17460541-17567954** | **none** | **1** |
| **4296** | **chr Y: 25525630-25538844** | **none** | **1** |
| **4297** | **chr Y: 28424069-28500565** | **none** | **1** |

**Table S1.** Nucleotide sequence location for triplex binding sites between *PARTICLE* and the human genome (hg19) as predicted by Triplex Domain Finder software (www.regulatory-genomics.org/tdf) ^1^. Regions with a second associated gene highlighted in blue.

**Reference**

1 Hanzelmann Sonja, K. C.-C., Kalwa Marie, Wagner Wolfgang, Costa Ivan G. Triplex Domain Finder: Detection of Triple Helix Binding Domains in Long Non-Coding RNAs. (2015).
